# Global, regional, and national burden of road injuries 1990–2023: a systematic analysis for the Global Burden of Disease Study 2023

**DOI:** 10.1016/S2468-2667(26)00141-6

**Published:** 2026-07-20

**Authors:** GBD 2023 Road Injuries Collaborators, Stephanie C C van der Lubbe, Madeline E Moberg, Dominique Aluquin, Yawen Zhou, Hailey Lenox, Abdullah, Bhoomadevi A, Hasan Aalruz, Ukachukwu O Abaraogu, Zahra Abbasi Dolatabadi, Hedayat Abbastabar, Siddig Ibrahim Abdelwahab, Meriem Abdoun, Zinat Abdrakhmanova, Deldar Morad Abdulah, Auwal Abdullahi, Toufik Abdul-Rahman, Tesfaye Birhanu Abebe, Armita Abedi, Hansani Madushika Abeywickrama, Olumide Abiodun, Richard Gyan Aboagye, Abdullahi Tunde Aborode, Nagah M Abourashed, Mohamed Abouzid, Teklebirhan Abrha Abrha, Dariush Abtahi, Fuad Hamdi A Abuadas, Aminu Kende Abubakar, Rana Kamal Abu-Farha, Ahmad Y Abuhelwa, Hana J Abukhadijah, Niveen ME Abu-Rmeileh, Dina Abushanab, Apurba Acharya, Kiran Acharya, Aishah Fadila Adamu, Isaac Yeboah Addo, Tajudeen Adesanmi Adebisi, Babatope Oluwadamilare Adebiyi, Kamoru Ademola Adedokun, Victor Adekanmbi, Olumide Thomas Adeleke, Adekola George Adepoju, Miracle Ayomikun Adesina, Prince Owusu Adoma, Obed Adonteng-Kissi, Clifford Afoakwah, Aanuoluwapo Adeyimika Afolabi, Ebenezer Afrifa-Yamoah, Aiman P Afsar, Mahnaz Afshari, Saira Afzal, Vijay K Aggarwal, Mahdi Aghaalikhani, Salome Wumpini Agordoh, Shreyash Agrawal, Williams Agyemang-Duah, Abdul Momin Rizwan Ahmad, Aqeel Ahmad, Danish Ahmad, Khabir Ahmad, Muayyad M Ahmad, Sajjad Ahmad, Tauseef Ahmad, Ali Ahmadi, Ali Ahmed, Ayman Ahmed, Haroon Ahmed, Mehrunnisha Sharif Ahmed, Mohamed Sherif Ali Ahmed, Muktar Beshir Ahmed, Shabbir Ahmed, Dolapo Emmanuel Ajala, Marjan Ajami, Neda Akhoundi, Salah Al Awaidy, Syed Mahfuz Al Hasan, Mohammad Ahmmad Mahmoud Al Zoubi, Sami Saleh Alabdulwahab, Khurshid Alam, Mohammad Khursheed Alam, Mohammed Fasihul Alam, Mostafa Alam, Muath A Alammar, Fahad Mashhour Alanezi, Amani N. Alansari, Fahmi Y Al-Ashwal, Hussin Albargi, Mohammed Albashtawy, Shereen M Aleidi, Fadwa Naji Alhalaiqa, Amidu Alhassan, Ashraf Alhumaidi, Dari Alhuwail, Irfan Ali, Kamran Ali, Mohammad Shahid Ali, Mohammed Usman Ali, Sheikh Mohammad Alif, Samah W Al-Jabi, Moath Saleh Aljohani, Syed Mohamed Aljunid, Zaid I Alkhatib, Hazim Alkousheh, Sameer Abdulmalik Alkubati, Khaled S. Allemailem, Mohammed Z Allouh, Wesam Taher Almagharbeh, Sabah Al-Marwani, Ahmad Almatroudi, Joseph Uy Almazan, Khalid Almugheed, Mohmmad Minwer Alnaeem, Fahad Salman Alotibi, Mushtak Talib Salih Al-Ouqaili, Jaber S Alqahtani, Saeed Ali S Alqahtani, Ahmad Alrawashdeh, Intima Alrimawi, Sahel Majed Alrousan, Mohammed A Alsabri, Mohammed Amjed Alsaegh, Bandar Alsaif, Abdullah Alshami, Ali Salman Al-Shami, Abdalkarem Fedgash Alsharari, Zaid Altaany, Malik A Althobiani, Nelson Alvis-Guzman, Nelson J Alvis-Zakzuk, Yaser Mohammed Al-Worafi, Abdulmohsen Hamdan Al-Zalabani, Junaid Amin, Tarek Tawfik Amin, Saeed Amini, Kafayat Aminu, Anandhi Deva Amirtharaj, Ganiyu Adeniyi Amusa, David B Anderson, Abhishek Anil, A.H. Sruthi Anil Kumar, Aisha Ansari, Abebe Alemu Anshebo, Boluwatife Stephen Anuoluwa, Saeid Anvari, Jalal Arabloo, Jesil Mathew Aranjani, Aleksandr Y Aravkin, Demelash Areda, Benedetta Armocida, Anton A Artamonov, Francis Arthur-Holmes, Ashokan Arumugam, Mohammad Asghari-Jafarabadi, Mohammad Ashfaque, Tahira Ashraf, Muhammad Shahzad Aslam, Imad Asmar, Ömer Ataç, Mirbahador Athari, Seyyed Shamsadin Athari, Alok Atreya, Abadi Hailay Atsbaha, Julie Alaere Atta, Avinash Aujayeb, Leticia Avila-Burgos, Faseeh Bin Awais, Sana Javaid Awan, Seyyed HamidReza Ayatizadeh, Haleh Ayatollahi, Ahmad Ayed, Damilola Jeremiah Ayowole, AKM Azad, Arian Azadnia, Abrham W Azale, Alireza Azarboo, Tomasz Bączek, Andreea Corina Badache, Ashish D Badiye, Adewale Ismahil' Badru, Youngoh Bae, Nayereh Baghcheghi, Nasser Bagheri, Khlood K Baghlaf, Ruhai Bai, Atif Amin Baig, Lovenish Bains, Shankar M Bakkannavar, Yatan Pal Singh Balhara, Jose Balmori-de-la-Miyar, Khushbu Balsara, Ovidiu Constantin Baltatu, Samuel Alemu Bamboro, Juan S Barajas-Gamboa, Suzanne Lyn Barker-Collo, Amadou Barrow, Cornelia Anne Barth, Azadeh Bashiri, Rehana Basri, Mohammad-Mahdi Bastan, Abdul-Monim Batiha, Shazia Batool, Kavita Batra, Ravi Batra, Shahrzad Bazargan-Hejazi, PhD, Neeraj Bedi, Narasimha M Beeraka, Desalegn Weldebrhan Bekru, Suleiman Ayalew Belay, Asnake Gashaw Belayneh, Enku Shiferaw Belayneh, Bashir Bello, Salaheddine Bendak, Samiun Nazrin Bente Kamal Tune, Aliyi Daba Benti, Alemshet Yirga Berhie, Mansi Sanjaykumar Bhadiyadra, Sonu Bhaskar, Arushee Bhatnagar, Sudip Bhattacharya, Jasvinder Singh Bhatti, Manpreet Singh Bhatti, Zulfiqar A Bhutta, Fengsai Bie, Corey B Bills, Abdulhakeem Binhambali, Paria Bolourinejad, Guilherme Borges, Dipan Bose, Samuel Adolf Bosoka, Alejandro Botero Carvajal, Souad Bouaoud, Soufiane Boufous, Dejana Braithwaite, Nicholas Allan Buckley, Tsion Samuel Bunare, Felix Busch, Yasser Bustanji, Senthilkumar C, Luis Alberto Cámera, Julio Cesar Campuzano Rincon, Zhong Cao, Angelo Capodici, Felix Carvalho, Ana Paula Carvalho-e-Silva, Joao Mauricio Castaldelli-Maia, Luca Cegolon, Ester Cerin, Sonia Cerrai, Sandip Chakraborty, Rama Mohan Chandika, Vijay Kumar Chattu, Anis Ahmad Chaudhary, Sirshendu Chaudhuri, Akashdeep Singh Chauhan, Akhilanand Chaurasia, Hana Chen, Patrick R Ching, Bryan Chong, Hitesh Chopra, Dinesh Kumar Choudhury, Jakir Ahmed Chowdhury, Mirza Farhana Iqbal Chowdhury, Steffan Wittrup McPhee Christensen, Isaac Sunday Chukwu, Eric Chung, Sunghyun Chung, Claudia Cosma, Stefania Curti, Omid Dadras, Hana Mahdi Dahir, Xiaochen Dai, Emanuele D'Amico, Lenards Obeng Damoah, Anh Kim Dang, Reza Darvishi Cheshmeh Soltani, Elham Davtalab Esmaeili, Bryan Gervais de Liyis, Ivan Delgado-Enciso, Andreas K Demetriades, Emina Dervišević, Aragaw Tesfaw Desale, Mitiku Desalegn, Vinoth Gnana Chellaiyan Devanbu, Devananda Devegowda, Arkadeep Dhali, Sreedhar Dharmagadda, Samath Dhamminda Dharmaratne, Bibha Dhungel, Adriana Dima, Mbekuveh Dinga-Nyoh, Vishal Diwan, Sushil Dohare, Jasmin Dönicke, Mario D'Oria, Fariba Dorostkar, Ojas Prakashbhai Doshi, Robert Kokou Dowou, Menayit Tamrat Dresse, Senbagam Duraisamy, Venkata Ravikanth Eddula, Yohannes Tekalegn Efa, Moaheda E H Eissa, Michael Ekholuenetale, Ibrahim Farahat El Bayoumy, Iman El Sayed, Mohamed Ahmed Eladl, Said El-Ashker, Iffat Elbarazi, Marwan El-Deyarbi, Hala Rashad Elhabashy, Muhammed Elhadi, Omar Abdelsadek Abdou Elmeligy, Ashraf A El-Metwally, Randa Elsheikh, Sharareh Eskandarieh, Adeniyi Francis Fagbamigbe, Ayesha Fahim, Ebenezer Gezahegn Fanta, Emerito Jose Aquino Faraon, Abdullah Farasani, Mohammad Fareed, Carla Sofia e Sá Farinha, Andre Faro, Syed Muhammad Yousaf Farooq, Nelsensius Klau Fauk, Mohammad Fayaz, Alireza Feizkhah, Ginenus Fekadu, Abdullah H Feroze, Nuno Ferreira, Aemon Berhane Fissha, Luisa S Flor, Behzad Foroutan, Matteo Foschi, Kayode Raphael Fowobaje, Richard Charles Franklin, Abdelrahman Gamil Gad, Muktar A Gadanya, Márió Gajdács, Balasubramanian Ganesh, Ezhilkugan Ganessane, Fernando Jr Barroga Garcia, Jacopo Garlasco, Zisis Gatzioufas, Ali Gerami Matin, Kalab Yigermal Gete, Mustafa Ghaderzadeh, Roya Ghafoury, Leila Ghalichi, Arin Ghamkhar, Ali Ghandili, Moein Ghasemi, Ahmad Ghashghaee, Sama Ghoba, Ali Gholamrezanezhad, Thinh Gia Gia Nguyen, Alem Abera Girmay, Alessandro Girombelli, Massimiliano Gobbo, Kimiya Gohari, Mahaveer Golechha, Pouya Goleij, Mohsen Golkar, Wei-Jie Gong, Shirisha Gooty, Shashikumar Channmgere Govindappa, Michal Grivna, Shi-Yang Guan, Giovanni Guarducci, Mohammed Ibrahim Mohialdeen Gubari, Damitha Asanga Gunawardane, Sapna Gupta, Pooja Gurnal, Reyna Alma Gutiérrez, Roberth Steven Gutiérrez-Murillo, Adrina Habibzadeh, Aryamawit Habteyesus, Shaymaa Hafedh Alkubaisy, Nguyen Hai Nam, Alazar Menbere Haile, Pritam Halder, Randah R Hamadeh, Fatimah Othman Hamarasool, Erin B Hamilton, Abdelrahman M Hamouda, Asif Hanif, Nasrin Hanifi, Mohd Anul Haq, Arief Hargono, Sara Harsini, Hamidreza Hasani, Reza Hashempour, Mohammad Hashem Hashempur, Nada Tawfig Hashim, Ibrahim Nagmeldin Hassan, Ikrama Hassan, Jeffrey J Hebert, Mohammad Heidari, Mojtaba Heydari, Matthew Hobbs, Ramesh Holla, Md Sabbir Hossain, Syed Billal Hossain, Mehdi Hosseinzadeh, Sorin Hostiuc, Junjie Huang, Ayesha Humayun, Waqar Husain, Salman Hussain, Segun Emmanuel Ibitoye, Aminu Alhassan Ibrahim, Fatma Magdi Ibrahim, Anel Ibrayeva, Olayinka Stephen Ilesanmi, Irena M Ilic, Milena D Ilic, Mustapha Immurana, Endang Indriasih, Danish Iqbal, Bilal Irfan, Lalu Muhammad Irham, Md Rabiul Islam, Md Mojahedul Islam, Md Shahinul Islam, Chidozie Declan Iwu, Oluyinka Ajibola Iyiola, Juan S Izquierdo-Condoy, Jaison Jacob, Louis Jacob, Kathryn H Jacobsen, Ali Jadidi, Mihajlo Jakovljevic, Reza Jalilzadeh Yengejeh, Jerin James, Roland Dominic G Jamora, Balamurugan Janakiraman, Rajiv Janardhanan, Saad Javed, Talha Jawaid, Sathish Kumar Jayapal, Ruwan Duminda Jayasinghe, Achala Upendra Jayatilleke, Seogsong Jeong, Bijay Mukesh Jeswani, Ravi Prakash Jha, Joseph Johnson, Jost B Jonas, Tamas Joo, Akaninyene Paul Joseph, Nitin Joseph, Bhawana Joshi, Krupal Joshi, Charity Ehimwenma Joshua, Thenmozhi K, Zubair Kabir, Dler Hussein Kadir, Bashir Kaka, Ashish Kumar Kakkar, Leila R Kalankesh, Khalil Kalavani, Rajesh Kamath, Ramat T Kamorudeen, Jiseung Kang, Kehinde Kazeem Kanmodi, Rami S Kantar, Neeti Kapoor, Sunil Kumar Kar, Mehrdad Karajizadeh, Ibraheem M Karaye, Arman Karimi Behnagh, Samad Karkhah, Mohmed Isaqali Karobari, Faizan Zaffar Kashoo, Nehemia I Kassa, Joonas H Kauppila, Neda Kaydi, Peter Njenga Keiyoro, Yousef Saleh Khader, Morteza Abdullatif Khafaie, Ajmal Khan, Hayat Ahmad Khan, Iqra Hamid Khan, Maseer Khan, Md Abdullah Saeed Khan, Muhammad Arslan Khan, Serab Khan, Sumaiya Khan, Uzma Rahim Khan, Yusuf Saleem Khan, Zahid Khan, Srijana Khanal, Sameer Uttamaro Khasbage, Haitham Khatatbeh, Moawiah Mohammad Khatatbeh, Kavin Khatri, Hamid Reza Khayat Kashani, Afshin Khazaei, Khalid A Kheirallah, Daniel Kheradmand, Mehdi Khezeli, Milad Khorasani, Samira Khoshvaght, Jagdish Khubchandani, P Ratan Khuman, Yun Jin Kim, Adnan Kisa, Marta A Kisiel, Abdul Basith KM, Shivakumar KM, Prakash Babu Kodali, Ali-Asghar Kolahi, Farzad Kompani, Hamid Reza Koohestani, Wolyu Erkano Korma, Tapos Kormoker, Ayan Hussien Korse, Arthi S Kozhumam, Kewal Krishan, Varun Krishna, Burcu Kucuk Bicer, Mohammed Kuddus, Mukhtar Kulimbet, Emmanuel Kumah, Chandan Kumar, Dewesh Kumar, Kamal Kumar, Nithin Kumar, Pawan Kumar, Pradeep Kumar, Prakash Kumar, Radha Kumar, Rakesh Kumar, Sandeep Kumar, Sumit Kumar, Tushar Kumar, Om P Kurmi, Asep Kusnali, Dian Kusuma, John Paul Kuwornu, Ville Kytö, Pallavi L C, Carlo La Vecchia, Muhammad Awwal Ladan, Lucie Laflamme, Chandrakant Lahariya, Timo Lajunen, Balzhan Lakanova, Tri Laksono, Tamás Lantos, Savita Lasrado, Mahrukh Latif, Paolo Lauriola, Caterina Ledda, Ka Yiu Lee, Munjae Lee, Seung Won Lee, Wei-Chen Lee, Yo Han Lee, Ja-Ho Leigh, Vasileios Leivaditis, Dawit Alemu Lemma, Ming-Chieh Li, Xiaopan Li, Yan Li, Zhengrui Li, Ambreen Liaqat, Xuefeng Liu, José Francisco López-Gil, Platon D Lopukhov, Surbala Devi Lourembam, Abhilash Ludhiadch, Angelina M Lutambi, Miltiadis D Lytras, Ellina Lytvyak, Zheng Feei Ma, Monika Machoy-Rakoczy, Firoozeh Madadi, Ahmed A Madfa, Hassan Magdy Abd El Razek, Mohammed Magdy Abd El Razek, Sasikumar Mahalingam, Samatar Abshir Mahamed, Preeti Maharjan, Nozad Hussein Mahmood, Azeem Majeed, Mostafa Majidnia, Igor Lima Maldonado, Deborah Carvalho Malta, Lokesh Manjani, Ramon Martinez-Piedra, J Jenifer Florence Mary, Roy Rillera Marzo, Sammer Marzouk, Sharareh Massahi, Yasith Mathangasinghe, Neeta Mathur, Khurshid A Mattoo, Steven M McPhail, Tesfahun Mekene Meto, Agezegn Asegid Mekonnen, Tesfaye Hambisa Mekonnen, Tadesse Belayneh Melkie, Osires de Medeiros Melo Neto, Walter Mendoza, Godfred Antony Menezes, Ritesh G Menezes, Emiru Ayalew Mengistie, Anusha Sultan Meo, Tuomo J Meretoja, Tomislav Mestrovic, Chamila Dinushi Kukulege Mettananda, Sachith Mettananda, Mohamed M M Metwally, Bartosz Miazgowski, Ted R Miller, Giuseppe Minervini, Long Chiau Ming, Prasanna Mithra, Chaitanya Mittal, Dhruti Modi, Lia Solomon Mogus, Mona Gamal Mohamed, Nouh Saad Mohamed, Khabab Abbasher Hussien Mohamed Ahmed, Taj Mohammad, Mokhtar Mohammadi, Abdollah Mohammadian-Hafshejani, Shafiu Mohammed, Mohammad Mohseni, Ali H Mokdad, Mohammad Ali Moni, Julio Cesar Montañez, Marie Chantel Montás, Rafael Silveira Moreira, Dimitrios Moris, Mahdis Morovvati, Shane Douglas Morrison, Mahmoud M Morsy, Md Shihab Mostafa, Rohith Motappa, Kimia Mozahheb Yousefi, Sumaira Mubarik, Shiv Kumar Mudgal, Jibran Sualeh Muhammad, Mubarak Muhammad, Sumoni Mukherjee, Francesk Mulita, Malaisamy Muniyandi, Efren Murillo-Zamora, Satta Muthu Murugan, Balaganapathy Muruganantham, Abdisalam Hassan Muse, David Musoke, Sathish Muthu, Ayoub Nafei, Ganesh R Naik, Gopal Nambi, Vinay Nangia, Shumaila Nargus, Abdulqadir J Nashwan, Mahmoud Nassar, Zuhair S Natto, Javaid Nauman, Zakira Naureen, Samidi Nirasha Kumari Navaratna, Biswa Prakash Nayak, Kinjal Nayak, Iffat Nayila, Javad Nazari, G Takop Nchanji, Nini Asfaw Negash, Ionut Negoi, Samata Nepal, Cuong Tat Nguyen, Hien Thu Nguyen, Trang Nguyen, Taxiarchis Konstantinos Nikolouzakis, Ali Nikoobar, Peishan Ning, Dina Nur Anggraini Ningrum, Vikram Niranjan, Richmond Nketia, Shuhei Nomura, Chisom Adaobi Nri-Ezedi, Jean Claude Nshimiyimana, Chimezie Igwegbe Nzoputam, Ogochukwu Janet Nzoputam, Bogdan Oancea, Kwabena Obiri - Yeboah, Fabio Massimo Oddi, Ismail A Odetokun, Felix Akpojene Ogbo, Abiola Ogunkoya, Olusegun Olatunji Ojedoyin, Hassan Okati-Aliabad, Osaretin Christabel Okonji, Oluwaseyi Isaiah Olabisi, Andrew T Olagunju, Abdulhakeem Abayomi Olorukooba, Oluseye Olalekan Oludoye, Ahmed Omar Bali, Goran Latif Omer, Michal Ordak, Hans Orru, Esteban Ortiz-Prado, Augustus Osborne, Alaa Oteir, Adrian Otoiu, Amel Ouyahia, Mayowa O Owolabi, Tope Oyelade, Mahesh P A, Kevin Pacheco-Barrios, Jagadish Rao Padubidri, Ashok Pandey, Sanjib Raj Pandey, Seithikurippu R Pandi-Perumal, Georgios D Panos, Ilias Papadimopoulos, Evangelos Pappas, Shahina Pardhan, Chulwoo Park, Eun-Cheol Park, Pragnesh Parmar, Ava Pashaei, Maja Pasovic, Roberto Passera, Bhargav Umeshchandra Patel, Hemal M Patel, Kunal Nitinkumar Patel, Niki Rut Patel, Sangram Kishor Patel, Vipin Patidar, Shankargouda Patil, Mohamed Anas Patni, Dimitrios Patoulias, Uttam Paudel, Shrikant Pawar, Hamidreza Pazoki Toroudi, Arun Mitra Peddireddy, Amy E Peden, Jarmila Pekarcikova, Veincent Christian Filipino Pepito, Prince Peprah, Daniela A Perez-Chadid, Simone Perna, Anil K Philip, Julian David Pillay, Mapa M Prabhath N Piyasena, Shiny PJ, Evgenii Plotnikov, Roman V Polibin, Reza Pourbabaki, Farzad Pourghazi, Mohsen Poursadeqiyan, Naeimeh Pourtaheri, Sergio I Prada, Harsh Priya, Muhammad Usman Qamar, Xiang Qi, Mohammad Qodrati, Nuzul Qur'aniati, Basuki Rachmat, Venkatraman Radhakrishnan, Amir Rafe, Ibrar Rafique, Yashpal Singh Raghav, Pracheth Raghuveer, Sajjad Rahimi, Vafa Rahimi-Movaghar, Firman Suryadi Rahman, Fryad Majeed Rahman, Mohammad Hifz Ur Rahman, Mosiur Rahman, Muhammad Aziz Rahman, Amir Masoud Rahmani, Amita Rai, Pramila Rai, Sunil Kumar Raina, Judah Rajendran, Shaman Rajindrajith, Pushp Lata Rajpoot, Mahmoud Mohammed Ramadan, Majed Ramadan, Kadar Ramadhan, Shakthi Kumaran Ramasamy, Balaji Ramraj, Lokesh Rana, Nemanja Rancic, Asad Gul Rao, Kumuda Rao, Mithun Rao, Sowmya J Rao, Prateek Rastogi, Devarajan Rathish, Rashita Ravi, Salman Rawaf, Karthik Rayapureddi, Ateeq Rehman, Rainer Reile, Mina Rezaei, Nazila Rezaei, Mohsen Rezaeian, Moattar Raza Rizvi, Jefferson Antonio Buendia Rodriguez, Leonardo Roever, Ravi Rohilla, Chanapong Rojanaworarit, Moustaq Karim Khan Rony, Himanshu Sekhar Rout, Bedanta Roy, Polani Rubeshkumar, Devarajulu Reddy S, Manjula S, Aly M A Saad, Siamak Sabour, Fatemeh Sadeghi-Ghyassi, Mohammad Reza Saeb, Mohd Saeed, Umar Saeed, Mehdi Safari, Sher Zaman Safi, Amene Saghazadeh, Fatemeh Saheb Sharif-Askari, Narjes Saheb Sharif-Askari, Amirhossein Sahebkar, Vaibhav Sahni, Durgesh Prasad Sahoo, Zahra Saif, Morteza Saki, Joseph W Sakshaug, Payman Salamati, Afeez Abolarinwa Salami, Samreen Saleem, Sohrab Salimi, Jayami Eshana Samaranayake, Yoseph Leonardo Samodra, Vijaya Paul Samuel, Fasineh Shekuba Samura, Abdallah M Samy, Rama Krishna Sanjeev, Milena M Santric-Milicevic, Aswini Saravanan, Yaser Sarikhani, Mohammad Sarmadi, Sachin C Sarode, Michele Sassano, Mukesh Kumar Sathya Narayanan, Maheswar Satpathy, Jennifer Saulam, Ganesh Kumar Saya, Christophe Schinckus, David C Schwebel, John W Scott, Hamidreza Seifmanesh, Siddharthan Selvaraj, Mohammad H Semreen, Subramanian Senthilkumaran, Francesco Sessa, Yashendra Sethi, Ahmed Nabil Shaaban, Shipraa Arpan Shah, Sweni Shah, Shazlin Shaharudin, Amira A Shaheen, Samiah Shahid, Moyad Jamal Shahwan, Masood Ali Shaikh, Muhammad Aaqib Shamim, Amir Hossein Shams, Mehran Shams-Beyranvand, Alfiya Shamsutdinova, Dan Shan, Mohd Shanawaz, Amin Sharifan, Javad Sharifi Rad, Prof. Fariborz Sharifian Jazi, Bhoopesh Kumar Sharma, Bunty Sharma, Jyoti Sharma, Manoj Sharma, Ravi Kumar Sharma, Saurab Sharma, Vishal Sharma, Bereket Beyene Shashamo, Shamee Shastry, Ramzi Shawahna, Samrawit Shawel, Maryam Shayan, Ali Sheidaei, Nishat Ahmed Sheikh, Rahim Ali Sheikhi, Jiabin Shen, Samendra P Sherchan, Mahabalesh Shetty, Pavanchand Shetty, Premalatha K Shetty, Suraj S Shetty, Aminu Shittu, Nathan A Shlobin, Sina Shool, Seyed Afshin Shorofi, Kerem Shuval, Md. Kaoser Bin Siddique, Gustavo Correia Basto da Silva, Padam Prasad Simkhada, Akanksha Singh, Harmanjit Singh, Paramdeep Singh, Poornima Suryanath Singh, Surendra Singh, Surjit Singh, Natia Skhvitaridze, Valentin Yurievich Skryabin, Bogdan Socea, Md Salman Sohel, Solikhah Solikhah, Yizhe Song, Lokasani Vijaya Sree, Vignes Anand Srinivasalu, Muhammad Haroon Stanikzai, Paschalis Steiropoulos, Sebastian Straube, Chen-Yang Su, Omer Subasi, Vetriselvan Subramaniyan, Agus Sudaryanto, Nathan Suga, Thitiporn Sukaew, Surajo Kamilu Sulaiman, Mark J M Sullman, Jing Sun, Suraj Sundaragiri, Katharina S Sunnerhagen, Chandan Kumar Swain, Lukasz Szarpak, Sree Sudha T Y, Rafael Tabarés-Seisdedos, Seyyed Mohammad Tabatabaei, Celine Tabche, Ramin Tabibi, Moslem Taheri Soodejani, Iman M Talaat, Mircea Tampa, Ingan Ukur Tarigan, Mengistie Kassahun Tariku, Anika Tasnim, Aigul Yelgondiyevna Tazhiyeva, Nebyu Lakew Tefera, Emre Tel, Gebremaryam Temesgen, Mohamad-Hani Temsah, Reem Temsah, Azimeraw Arega Tesfu, Enoch Teye-Kwadjo, Dejina Thapa, Rekha Thapar, Ismaeel Tharwat, Jeevarathinam Thirumalai, Benson Thomas M, Harshal R Thube, Jansje Henny Vera Ticoalu, Madi Tleshev, Santo Imanuel Tonapa, Arole Darwin Touko, Marcos Roberto Tovani-Palone, Thang Huu Tran, Nguyen Tran Minh Duc, Domenico Trico, Mike Tuffour Amirikah, Lawrence Sena Tuglo, Munkhtuya Tumurkhuu, Ermias A Turuse, Biruk Shalmeno Tusa, Irfan Ullah, Saeed Ullah, Srikanth Umakanthan, Lawan Umar, Bhaskaran Unnikrishnan, Carolyn Anne Unsworth, Selamu Amanuel Untiso, Era Upadhyay, Sheela Upendra, Jibrin Sammani Usman, Rakesh V R, Asokan Govindaraj Vaithinathan, Pascual R Valdez, Tommi Juhani Vasankari, Narayanaswamy Venketasubramanian, Akshaya Kumar Verma, Massimiliano Veroux, Georgios-Ioannis Verras, Victor E Villalobos-Daniel, Tuan Vinh, Francesco S Violante, Vasily Vlassov, Yasir Waheed, Arvinder Wander, Wanzhou Wang, Wei Wang, Yanzhong Wang, Apichai Wattanapisit, Anthony Ike Wegbom, Fei-Long Wei, Andrea Werdecker, Taweewat Wiangkham, Dakshitha Praneeth Wickramasinghe, Faustine Williams, Andrew Awuah Wireko, Wanqing Xie, Patlolla Sriram Yadav, Vikas Yadav, Musa Yahaya, Saba Yahoo, Eesha Yaqoob, Sanni Yaya, Pengpeng Ye, Ali Cem Yekdeş, Siyan Yi, Arzu Yiğit, Vahit Yiğit, S Yogeshkumar, Dong Keon Yon, Naohiro Yonemoto, Mustafa Younis, Yong Yu, Siddhesh Zadey, Mubashir Zafar, Emilia Zainal Abidin, Dilmurat Zairov, Aurora Zanghì, Iman Zare, Michael Zastrozhin, Saad Zbiri, Mohammed G M Zeariya, Haijun Zhang, Yunquan Zhang, Zhi-Jiang Zhang, Claire Chenwen Zhong, Hao Zhou, Abzal Zhumagaliuly, Yossef Teshome Zikarg, Sa'ed H Zyoud, Shaher H Zyoud, Christopher J L Murray, Simon I Hay, Marie Ng, Kanyin Liane Ong

**Affiliations:** A NUS-IHME Global Burden of Disease Research Centre, Yong Loo Lin School of Medicine, National University of Singapore, Singapore, Singapore; B Institute for Health Metrics and Evaluation, University of Washington, Seattle, WA, USA; C Medicine Department, Rawalpindi Medical University, Rawalpindi, Pakistan; D Amity Institute of Public Health and Hospital Administration, Amity University Noida, Uttar Pradesh, India; E Department of Nursing, Al Zaytoonah University of Jordan, Amman, Jordan; F School of Health and Life Sciences, University of the West of Scotland, Paisley, UK; G Department of Medical Rehabilitation, University of Nigeria Nsukka, Enugu, Nigeria; H Department of Medical-Surgical Nursing, Tehran University of Medical Sciences, Tehran, Iran; I Advanced Diagnostic and Interventional Radiology Research Center (ADIR), Tehran University of Medical Sciences, Tehran, Iran; J Health Research Centre, Jazan University, Jazan, Saudi Arabia; K Department of Medicine, University of Setif Algeria, Sétif, Algeria; L Department of Health, University of Setif Algeria, Sétif, Algeria; M Health Research Institute, Al Farabi Kazakh National University, Almaty, Kazakhstan; N Community and Maternity Nursing Unit, University of Duhok, Duhok, Iraq; O Department of Physiotherapy, Bayero University Kano, Kano, Nigeria; P Department of Physiotherapy, Federal University Wukari, Wukari, Nigeria; Q Department of Research, Toufik's World Medical Association, Sumy, Ukraine; R School of Medicine, Salale University, Fitche, Ethiopia; S Department of Emergency Medicine, Zanjan University of Medical Sciences, Zanjan, Iran; T School of Health Sciences, Niigata University, Niigata, Japan; U Department of Community Medicine, Babcock University, Ilishan-Remo, Nigeria; V Department of Family and Community Health, University of Health and Allied Sciences, Ho, Ghana; W School of Population Health, University of New South Wales, Sydney, NSW, Australia; X Department of Research and Development, Healthy Africans Platform, Ibadan, Nigeria; Y Zoology Department, Benha University, Benha, Egypt; Z Department of Physical Pharmacy and Pharmacokinetics, Poznan University of Medical Sciences, Poznan, Poland; AA College of Medicine and Health Sciences, Adigrat University, Adigrat, Ethiopia; AB Department of Anesthesiology, Shahid Beheshti University of Medical Sciences, Tehran, Iran; AC Primary Care Nursing Department, Al-Ahliyya Amman University, Amman, Jordan; AD Graduate School of Public Health, St. Luke's International University, Tokyo, Japan; AE Division of Population Data Science, National Cancer Center, Tokyo, Japan; AF Clinical Pharmacy and Therapeutics Department, Applied Science Private University, Amman, Jordan; AG Department Pharmacy Practice and Pharmacotherapeutics, University of Sharjah, Sharjah, United Arab Emirates; AH Clinical Trials Unit, Hamad Medical Corporation, Doha, Qatar; AI College of Health Sciences, Qatar University, Doha, Qatar; AJ Birzeit University, Ramallah, Palestine; AK Office of Vice President for Medical and Health Sciences, Qatar University, Doha, Qatar; AL Institute of International Health, Charité Medical University Berlin, Berlin, Germany; AM Department of Research, New ERA, Kathmandu, Nepal; AN School of Medicine, University of Health and Allied Sciences, Ho, Ghana; AO School of Medicine, University of Sydney, Sydney, NSW, Australia; AP Centre for Social Research in Health, University of New South Wales, Sydney, NSW, Australia; AQ Department of Microbiology, Ladoke Akintola University, Osogbo, Nigeria; AR NMC Healthcare, Independent Consultant, Sharjah, United Arab Emirates; AS Department of Pediatrics, University of Calgary, Calgary, AB, Canada; AT School of Public Health, University of the Western Cape, Bellville, South Africa; AU Department of Immunology, Roswell Park Comprehensive Cancer Center, Buffalo, NY, USA; AV Graduate Program Division, University at Buffalo, Buffalo, NY, USA; AW Department of Obstetrics and Gynecology, University of Texas Medical Branch, Galveston, TX, USA; AX Department of Family Medicine, Bowen University, Iwo, Nigeria; AY Department of Family Medicine, Bowen University Teaching Hospital, Ogbomoso, Nigeria; AZ Department of Health Informatics, Indiana University Indianapolis, Indianapolis, IN, USA; BA Slum and Rural Health Initiative Research Academy, Slum and Rural Health Initiative, Ibadan, Nigeria; BB Department of Physiotherapy, University of Ibadan, Ibadan, Nigeria; BC Department of Health Administration and Education, University of Education Winneba, Winneba, Ghana; BD School of Arts and Social Sciences, Edith Cowan University, Bunbury, WA, Australia; BE Australian Centre for Health Services Innovation, Queensland University of Technology, Kelvin Grove, QLD, Australia; BF Jamieson Trauma Institute, Metro North Health, Herston, QLD, Australia; BG Technical Services Directorate, MSI Nigeria Reproductive Choices, Abuja, Nigeria; BH School of Science, Edith Cowan University, Perth, WA, Australia; BI Department of Plastic Surgery, Mayo Clinic, Rochester, MN, USA; BJ Social Determinants of Health Research Center, Saveh University of Medical Sciences, Saveh, Iran; BK Department of Community Medicine, King Edward Memorial Hospital, Lahore, Pakistan; BL Department of Public Health, Public Health Institute, Lahore, Pakistan; BM University of Texas Health Science Center at San Antonio, University of Texas, San Antonio, TX, USA; BN Department of Orthopedics and Sports Medicine, Boston Children's Hospital, Boston, MA, USA; BO Harvard University, Boston, MA, USA; BP Operations Department, World Vision, Kete Krachi, Ghana; BQ Department of Anesthesia, ABVIMS and DR RAM MANOHAR LOHIA Hospital, New Delhi, India; BR Department of Public Health Sciences, Queen's University, Kingston, ON, Canada; BS Department of Human Nutrition & Dietetics, National University of Science and Technology (NUST), Islamabad, Pakistan; BT Department of Health Sciences, University of York, York, England; BU College of Medicine, Shaqra University, Shaqra, Saudi Arabia; BV School of Medicine and Psychology, Australian National University, Canberra, ACT, Australia; BW Health Research Institute, University of Canberra, Canberra, NSW, Australia; BX King Khaled Eye Specialist Hospital and Research Center, Riyadh, Saudi Arabia; BY School of Nursing, University of Jordan, Amman, Jordan; BZ Department of Health and Biological Sciences, Abasyn University, Peshawar, Pakistan; CA Department of Natural Sciences, Lebanese American University, Beirut, Lebanon; CB School of Public Health, Zhejiang University, Hangzhou, China; CC Department of Community Health Sciences, Sohail University, Karachi, Pakistan; CD Department of Epidemiology and Biostatistics, Shahrekord University of Medical Sciences, Shahrekord, Iran; CE Department of Epidemiology, Shahid Beheshti University of Medical Sciences, Tehran, Iran; CF Department of Pharmacy Practice, Riphah Institute of Pharmaceutical Sciences, Islamabad, Pakistan; CG Division of Infectious Diseases and Global Public Health (IDGPH), University of California San Diego, San Diego, CA, USA; CH Institute of Endemic Diseases, University of Khartoum, Khartoum, Sudan; CI Pan-African One Health Institute (PAOHI), Kigali, Rwanda; CJ Department of Biosciences, COMSATS Institute of Information Technology, Islamabad, Pakistan; CK College of Nursing, Majmaah University, Al Majmaah, Saudi Arabia; CL Mansoura University Hospitals, Mansoura University Hospital, Mansoura, Egypt; CM College of Medicine and Public Health, Flinders University, Adelaide, SA, Australia; CN Faculty of Public Health, Jimma University, Jimma, Ethiopia; CO Maternal and Child Health Division (MCHD), International Centre for Diarrhoeal Disease Research, Bangladesh, Dhaka, Bangladesh; CP Bowen University Hospital, Bowen University, Iwo, Nigeria; CQ Operating Theatre, Bowen University Teaching Hospital, Ogbomoso, Nigeria; CR National Nutrition and Food Technology Research Institute, Shahid Beheshti University of Medical Sciences, Tehran, Iran; CS Department of Radiology, Children's Hospital of Philadelphia, Philadelphia, PA, USA; CT Diagnostic Radiology Department, Children's Hospital of Philadelphia, Philadelphia, PA, USA; CU Department of Communicable Diseases, Ministry of Health, Muscat, Oman; CV Middle East, Eurasia, and Africa Influenza Stakeholders Network, Muscat, Oman; CW Division of Public Health Sciences, Washington University in St. Louis, St. Louis, MO, USA; CX School of Public Health, University of Texas, Houston, TX, USA; CY Health Rehabilitation Sciences Department, King Saud University, Riyadh, Saudi Arabia; CZ Murdoch Business School, Murdoch University, Perth, WA, Australia; DA Preventive Dentistry Department, Jouf University, Sakaka, Saudi Arabia; DB Public Health Department, Qatar University, Doha, Qatar; DC Department of Oral and Maxillofacial Surgery, Shahid Beheshti University of Medical Sciences, Tehran, Iran; DD Department of Family Medicine, Shaqra University, Shaqra, Saudi Arabia; DE Imam Abdulrahman Bin Faisal University, Dammam, Saudi Arabia; DF Department of Pediatric Surgery, Hamad Medical Corporation, Doha, Qatar; DG Department of Clinical Pharmacy, Al-Ayen Iraqi University, Thi-Qar, Iraq; DH Department of Clinical Pharmacy and Pharmacy Practice, University of Science and Technology, Sana'a, Yemen; DI College of Nursing and Health Sciences, Jazan University, Jazan, Saudi Arabia; DJ Department of Community and Mental Health, Al Al-Bayt University, Mafraq, Jordan; DK College of Pharmacy, University of Sharjah, Sharjah, United Arab Emirates; DL School of Pharmacy, The University of Jordan, Amman, Jordan; DM College of Nursing, Qatar University, Doha, Qatar; DN Department of Adult Health, University of Cape Coast, Cape Coast, Ghana; DO Faculty of Dentistry, Ibn Al-Nafis University for Medical Sciences, Sana'a, Yemen; DP Department of Oral and Maxillofacial Surgery, Xi'an Jiaotong University, Xi'an, China; DQ Information Science Department, Kuwait University, Sabah Alsalem University City, Kuwait; DR Health Informatics Unit and Geohealth Lab, Dasman Diabetes Institute, Dasman, Kuwait; DS Department of Statistics and Operations Research, Aligarh Muslim University, Aligarh, India; DT College of Dental Medicine, Qatar University, Doha, Qatar; DU Department of Physical Therapy, University of Hail, Hail, Saudi Arabia; DV Department of Medical Rehabilitation (Physiotherapy), University of Maiduguri, Maiduguri, Nigeria; DW Nethersole School of Nursing, The Chinese University of Hong Kong, Hong Kong, China; DX Institute of Health and Wellbeing, Federation University Australia, Melbourne, VIC, Australia; DY School of Public Health and Preventive Medicine, Monash University, Melbourne, VIC, Australia; DZ Department of Clinical and Community Pharmacy, An-Najah National University, Nablus, Palestine; EA Family and Community Medicine Department, Qassim University, Al Qassim, Saudi Arabia; EB Department of Public Health and Community Medicine, International Medical University, Kuala Lumpur, Malaysia; EC International Centre for Casemix and Clinical Coding, National University of Malaysia, Bandar Tun Razak, Malaysia; ED Department of Allied Medical Sciences, Jordan University of Science and Technology, Irbid, Jordan; EE Faculty of Medicine, The Hashemite University, Zarqa, Jordan; EF Department of Medical Surgical Nursing, University of Hail, Hail, Saudi Arabia; EG Department of Medical Laboratories, College of Applied Medical Sciences, Qassim University, Saudi Arabia, Buraydah, Saudi Arabia; EH Department of Anatomy, United Arab Emirates University, Al Ain, United Arab Emirates; EI Faculty of Medicine, Jordan University of Science and Technology, Irbid, Jordan; EJ Faculty of Nursing, University of Tabuk, Tabuk, Saudi Arabia; EK Independent Consultant, Amman, Jordan; EL Department of Medical Laboratories, College of Applied Medical Sciences, Qassim University, Buraydah, Saudi Arabia; EM Department of Medicine, Nazarbayev University, Astana, Kazakhstan; EN College of Nursing, Riyadh Elm University, Riyadh, Saudi Arabia; EO Department of Clinical Nursing, Al-Zaytoonah University of Jordan, Amman, Jordan; EP Department of Physiotherapy, Taif University, Taif, Saudi Arabia; EQ Department of Microbiology, College of Medicine, University of Anbar, Ramadi, Anbar Governorate, Iraq; ER Department of Respiratory Care, Prince Sultan Military College of Health Sciences, Dammam, Saudi Arabia; ES Prince Sultan Military College of Health Sciences, Saudi Ministry of Defence, Dhahran, Saudi Arabia; ET Department of Nursing, Georgetown University, Washington, DC, USA; EU Macro-Fiscal Policy Department, Ministry of Finance, Dubai, United Arab Emirates; EV Department of Emergency Medicine, Sana'a University, Sanaa, Yemen; EW Pediatric Emergency Medicine Department, Drexel University, Philadelphia, PA, USA; EX College of Dental Medicine, University of Sharjah, Sharjah, United Arab Emirates; EY Research Institute for Medical and Health Sciences, University of Sharjah, Sharjah, United Arab Emirates; EZ Department of Public Health, University of Hail, Hail, Saudi Arabia; FA Al Wakra Hospital, Hamad Medical Corporation, Doha, Qatar; FB College of Health and Life Science, Hamad Bin Khalifa University, Doha, Qatar; FC Department of Pharmacology, Sana'a University, Sanaa, Yemen; FD Department of Basic Medical Sciences, Universiti Malaysia Sarawak, Kota Samarahan, Malaysia; FE College of Nursing, Jouf University, Sakaka, Saudi Arabia; FF Yarmouk University, Faculty of Medicine, Irbid, Jordan; FG Higher Collage of Technology, Higher Collage of Technology, Abu Dhabi, United Arab Emirates; FH Department of Respiratory Therapy, King Abdulaziz University, Jeddah, Saudi Arabia; FI Department of Respiratory Medicine, University College London, London, England; FJ Research Group in Health Economics, Universidad De Cartagena (University of Cartagena), Cartagena, Colombia; FK Research Group in Hospital Management and Health Policies, Universidad De La Costa (University of the Coast), Barranquilla, Colombia; FL Department of Health Sciences, Universidad De La Costa, Barranquilla, Colombia; FM Department of Health Technology Assessment, ALZAK, Cartagena, Colombia; FN Fakeeh College for Medical Sciences, Fakeeh College for Medical Sciences, Jeddah, Saudi Arabia; FO College of Medical Sciences, Azal University for Human Development, Sana'a, Yemen; FP College of Medicine, Taibah University, Madinah, Saudi Arabia; FQ Physiotherapy Department, University of Hail, Hail, Saudi Arabia; FR Clinical Sciences - Orthopaedic Department, University of Science Malaysia, Kota Baharu, Malaysia; FS Kasr Alainy Faculty of Medicine, Cairo University, Cairo, Egypt; FT Bio-Medical Research Department, Armed Forces College of Medicine, Cairo, Egypt; FU Department of Health and Management Sciences, Khomein University of Medical Sciences, Khomein, Iran; FV Department of Sociology, University of Ibadan, Ibadan, Nigeria; FW College of Nursing, SULTAN QABOOS University, Muscat, Oman; FX Department of Medicine, University of Jos, Jos, Nigeria; FY Department of Internal Medicine, Jos University Teaching Hospital, Jos, Nigeria; FZ Faculty of Medicine and Health, The University of Sydney, Sydney, NSW, Australia; GA Sydney Musculoskeletal Health, The University of Sydney, Sydney, NSW, Australia; GB Department of Pharmacology, All India Institute of Medical Sciences, Bhubaneswar, India; GC Division of Medical Research, SRM Medical College Hospital and Research Centre, Sri Ramaswamy Memorial Institute of Science and Technology, Chengalpattu, India; GD Department of Midwifery, Wachemo University, Hosaena, Ethiopia; GE Department of Environmental and Occupational Health, University of Medical Sciences, Ondo, Ondo, Nigeria; GF Regenerative Medicine, Organ Procurement and Transplantation Multi-disciplinary Center, Guilan University of Medical Sciences, Rasht, Iran; GG Health Management and Economics Research Center, Iran University of Medical Sciences, Tehran, Iran; GH Department of Pharmaceutical Biotechnology, Manipal Academy of Higher Education, Manipal, India; GI Department of Applied Mathematics, University of Washington, Seattle, WA, USA; GJ Department of Health Metrics Sciences, School of Medicine, University of Washington, Seattle, WA, USA; GK College of Art and Science, Ottawa University, Surprise, AZ, USA; GL Department of Cardiovascular, Endocrine-Metabolic Diseases and Aging, Istituto Superiore Di Sanità (ISS), Rome, Italy; GM Institute of Social, Medical, and Applied Research Technology, Moscow, Russia; GN Faculty of Business and Law, University of Portsmouth, Portsmouth, UK; GO Department of Physiotherapy, University of Sharjah, Sharjah, United Arab Emirates; GP Department of Physiotherapy, Manipal Academy of Higher Education, Manipal, India; GQ Cabrini Research, Cabrini Health, Malvern, VIC, Australia; GR Department of Biosciences, Integral University, Lucknow, India; GS Department of Biostatistics, Sakarya University, Sakarya, Türkiye; GT School of Traditional Chinese Medicine, Xiamen University Malaysia, Sepang, Malaysia; GU Department of Nursing, Birzeit University, Birzeit, Palestine; GV Institute of Population and Social Research, Marmara University, Istanbul, Türkiye; GW Department of Public Health, Istanbul Medipol University, Istanbul, Türkiye; GX Keck School of Medicine, University of Southern California, Los Angeles, CA, USA; GY Department of Immunology, Zanjan University of Medical Sciences, Zanjan, Iran; GZ Department of Forensic Medicine, Lumbini Medical College, Palpa, Nepal; HA College of Medicine and Health Sciences, Adigrat University, Adigart, Ethiopia; HB Management Policy and Community Health, University of Texas, Houston, TX, USA; HC Northumbria HealthCare NHS Foundation Trust, Newcastle upon Tyne, UK; HD Center for Health Systems Research, National Institute of Public Health, Cuernavaca, Mexico; HE Rawalpindi Medical University, Rawalpindi Medical University, Rawalpindi, Pakistan; HF Institute of Molecular Biology and Biotechnology (IMBB), The University of Lahore, lahore, Pakistan; HG Trauma Research Center, Shiraz University of Medical Sciences, Shiraz, Iran; HH Department of Health Information Management, Iran University of Medical Sciences, Tehran, Iran; HI Faculty of Nursing, Arab American University Palestine, Jenin, Palestine; HJ Department of Community Medicine and Primary Care, Federal Medical Centre, Abeokuta, Nigeria; HK Department of Mathematics & Statistics, Imam Mohammad Ibn Saud Islamic University, Riyadh, Saudi Arabia; HL Social Determinants of Health Research Center, Kurdistan University of Medical Sciences, Sanandaj, Iran; HM Brown School of Public Health, Washington University in St. Louis, St.Louis, MO, USA; HN School of Medicine, Tehran University of Medical Sciences, Tehran, Iran; HO Department of Pharmaceutical Chemistry, Medical University of Gdansk, Gdansk, Poland; HP Institute of Health Sciences, Pomeranian University in Slupsk, Slupsk, Poland; HQ School of Health Sciences, Orebro University, Orebro, Sweden; HR School of Public Health, Babes-Bolyai University, Cluj Napoca, Romania; HS Department of Forensic Science, Government Institute of Forensic Science Nagpur, Nagpur, India; HT Rashtrasant Tukadoji Maharaj Nagpur University, Nagpur, India; HU Department of Anesthesia and Intensive Care, University of Ibadan, Ibadan, Nigeria; HV Department of Neurological Surgery, University of Ulsan, Seoul, South Korea; HW Department of Nursing, Saveh University of Medical Sciences, Saveh, Iran; HX Pediatric Dentistry Department, King Abdulaziz University, Jeddah, Saudi Arabia; HY Clinical Medical Research Center, Nanjing Children's Hospital, Nanjing, China; HZ School of Public Affairs, Nanjing University of Science and Technology, Nanjing, China; IA Applied College, University of Tabuk, Tabuk, Saudi Arabia; IB Surgery, Global Surgery, Emergency Medicine Department, Maulana Azad Medical College, New Delhi, India; IC WHO Collaborating Centre for Research in Surgical Care Delivery in LMICs, Mumbai, India; ID Department of Forensic Medicine and Toxicology, Manipal Academy of Higher Education, Manipal, India; IE National Drug Dependence Treatment Centre, All India Institute of Medical Sciences, New Delhi, India; IF Anahuac Business School, Universidad Anahuac Mexico, Mexico City, Mexico; IG Department of International Health, Johns Hopkins University, Baltimore, MD, USA; IH Center of Innovation, Technology, and Education (CITE), Anhembi Morumbi University, São José dos Campos, Brazil; II School of Public Health, Arba Minch University, Arba Minch, Ethiopia; IJ Digestive Disease Institute, Cleveland Clinic Abu Dhabi, Abu Dhabi, United Arab Emirates; IK Centmed, New York University Abu Dhabi, Abu Dhabi, United Arab Emirates; IL School of Psychology, University of Auckland, Auckland, New Zealand; IM Department of Public and Environmental Health, University of The Gambia, Banjul, The Gambia; IN Department of Epidemiology, University of Florida, Gainesville, FL, USA; IO School of Health Professions, Bern University of Applied Sciences, Bern, Switzerland; IP Health Information Management, Shiraz University of Medical Sciences, Shiraz, Iran; IQ College of Medicine, Jouf University, Sakaka, Saudi Arabia; IR Non-communicable Diseases Research Center, Tehran University of Medical Sciences, Tehran, Iran; IS School of Medicine, Iran University of Medical Sciences, Tehran, Iran; IT Faculty of Nursing, King Khalid University, Mahyil Asir, Saudi Arabia; IU Institute of Chemical Sciences, Bahauddin Zakariya University, Multan, Pakistan; IV Department of Medical Education, University of Nevada Las Vegas, Las Vegas, NV, USA; IW School of Public Health, University of Nevada Las Vegas, Las Vegas, NV, USA; IX IT Department, Coforge, Georgia, GA, USA; IY Department of Psychiatry, Charles R. Drew University of Medicine and Science, Los Angeles, CA, USA; IZ Department of Psychiatry and Biobehavioral Sciences, University of California Los Angeles, Los Angeles, CA, USA; JA School of Public Health, Dr. D. Y. Patil University, Mumbai, India; JB Jazan University, Jazan, Saudi Arabia; JC Department of Human Anatomy and Histology, I.M. Sechenov First Moscow State Medical University, Moscow, Russia; JD Department of Adult Health Nursing/School of Nursing, Mekelle University, Mekelle, Ethiopia; JE Department of Dermatovenereology, University of Gondar, Gondar city, Ethiopia; JF Department of Emergency and Critical Care Nursing, Bahir Dar University, Bahir Dar, Ethiopia; JG Department of General Surgery, Plastic Surgery, St. Paul's Hospital Millennium Medical College, Addis Ababa, Ethiopia; JH Bayero University Kano, Higher National School of Veterinary Medicine, Kano, Nigeria; JI North-West University, Mafikeng, South Africa; JJ Department of Industrial Engineering, Haliç University, Istanbul, Türkiye; JK BRAC James P Grant School of Public Health, BRAC University, Dhaka, Bangladesh; JL Institute of Health Sciences, Wollega University, Nekemte, Ethiopia; JM Department of Nursing, Bahir Dar University, Bahir Dar, Ethiopia; JN Ashok & Rita Patel Institute of Physiotherapy, Charotar University of Science and Technology, Changa, India; JO Global Health Neurology Lab, NSW Brain Clot Bank, Sydney, NSW, Australia; JP Division of Cerebrovascular Medicine and Neurology, National Cerebral and Cardiovascular Center, Suita, Japan; JQ Family Medicine Department, Texas Tech University, El Paso, TX, USA; JR Department of Community and Family Medicine, All India Institute of Medical Sciences, Deoghar, India; JS Department of Human Genetics and Molecular Medicine, Central University of Punjab, Bathinda, India; JT Department of Botanical and Environmental Sciences, Guru Nanak Dev University, Amritsar, India; JU Centre for Global Child Health, University of Toronto, Toronto, ON, Canada; JV Institute for Global Health & Development, Aga Khan University, Karachi, Pakistan; JW School of Public Health, University of Nevada Reno, nevada, NV, USA; JX Department of Emergency Medicine, University of Colorado, Aurora, CO, USA; JY Department of Emergency Medicine, University of California San Francisco, San Francisco, CA, USA; JZ Department of Pathobiology, Tuskegee University, Tuskegee, AL, USA; KA Isfahan University of Medical Sciences, Isfahan, Iran; KB Department of Epidemiology and Psychosocial Research, Ramón De La Fuente Muñiz National Institute of Psychiatry, Mexico City, Mexico; KC Transport and Logistics Knowledge Bank, World Bank, Washington, DC, USA; KD Disease Surveillance Department, Ghana Health Service, Ho, Ghana; KE Department of Epidemiology and Biostatistics, University of Health and Allied Sciences, Ho, Ghana; KF Facultad De Salud (Faculty of Health), Universidad Santiago De Cali, Cali, Colombia; KG Department of Medicine, University Ferhat Abbas of Setif, Sétif, Algeria; KH Department of Epidemiology and Preventive Medicine, University Hospital Saadna Abdenour, Sétif, Algeria; KI Transport and Road Safety (TARS) Research Centre, University of New South Wales, Sydney, NSW, Australia; KJ Cancer Population Sciences Program, University of Florida Health Cancer Center, Gainesville, FL, USA; KK Faculty of Medicine and Health, University of Sydney, Sydney, NSW, Australia; KL Department of Environmental Health, Bahir Dar University, Bahir Dar, Ethiopia; KM School of Medicine and Health, Technical University of Munich, Munich, Germany; KN Department of Radiology, University of Cambridge, Cambridge, England; KO Department of Basic Biomedical Sciences, University of Sharjah, Sharjah, United Arab Emirates; KP The Apollo University, Apollo Hospital, Chittor, India; KQ Department of Internal and Geriatric Medicine, Hospital Italiano De Buenos Aires (Italian Hospital of Buenos Aires), Buenos Aires, Argentina; KR Board of Directors, Argentine Society of Medicine, Buenos Aires, Argentina; KS Centro De Investigaciones En Salud Poblacional, National Institute of Public Health, Cuernavaca, Mexico; KT School of Medicine, University of the Valley of Cuernavaca, Cuernavaca, Mexico; KU Heidelberg Institute of Global Health, Heidelberg University, Heidelberg, Germany; KV Interdisciplinary Research Center for Health Science, Sant'Anna School of Advanced Studies, Pisa, Italy; KW Research Unit on Applied Molecular Biosciences (UCIBIO), University of Porto, Porto, Portugal; KX School of Health Science, University of Sydney, Sydney, NSW, Australia; KY Education Center of Australia, Health Science College, Sydney, NSW, Australia; KZ Department of Psychiatry, University of Sao Paulo, São Paulo, Brazil; LA Department of Medical, Surgical, and Health Sciences, University of Trieste, Trieste, Italy; LB Public Health Unit, University Health Agency Giuliano-Isontina (ASUGI), Trieste, Italy; LC Mary MacKillop Institute for Health Research, Australian Catholic University, Melbourne, VIC, Australia; LD School of Public Health, University of Hong Kong, Hong Kong, China; LE Institute of Clinical Physiology, Italian National Council of Research, Pisa, Italy; LF State Disease Investigation Laboratory, Animal Resources Development Department, Agartala, India; LG Clinical Nutrition Department, Jazan University, Jazan, Saudi Arabia; LH Department of Public Health and Health Sciences, Tennessee State University (TSU), Nashville, TN, USA; LI Department of Community Medicine, Datta Meghe Institute of Medical Sciences, Sawangi, India; LJ Department of Biology, Imam Mohammad Ibn Saud Islamic University, Riyadh, Saudi Arabia; LK ICMR National Institute of Health Research, Dibrugarh, Indian Council of Medical Research, Dibrugarh, India; LL Indian Institute of Public Health-Delhi, Public Health Foundation of India, Gurugram, India; LM Department of Oral Medicine and Radiology, King George's Medical University, Lucknow, India; LN Faculty of Humanities and Health Sciences, Curtin University, Miri, Malaysia; LO Division of Infectious Diseases, Virginia Commonwealth University, Richmond, VA, USA; LP Department of Medicine, National University of Singapore, Singapore, Singapore; LQ Centre for Research Impact & Outcome, Chitkara University, Rajpura, India; LR Department of Humanities and Social Sciences, Sri Sathya Sai Institute of Higher Learning, Sri Sathya Sai District, India; LS Department of Pharmaceutical Technology, University of Dhaka, Dhaka, Bangladesh; LT Department of Surgery, Johns Hopkins University, Baltimore, MD, USA; LU Department of Health Science and Technology, Aalborg University, Aalborg, Denmark; LV Department of Physiotherapy, University College of Northern Denmark, Aalborg, Denmark; LW Department of Paediatric Surgery, Federal Medical Centre, Umuahia, Nigeria; LX Department of Urology, The University of Queensland, Brisbane, QLD, Australia; LY Department of AndroUrology, AndroUrology Centre, Brisbane, QLD, Australia; LZ Department of Health Behavior, Texas A&M University, College Station, TX, USA; MA Department of Health Sciences, University of Florence, Florence, Italy; MB Department of Medical and Surgical Sciences, University of Bologna, Bologna, Italy; MC Center for Immigrant and Refugee Health, Public Health Institute, Los Angeles, CA, USA; MD School of Postgraduate Studies and Research, Amoud University, Hargiesa, Somalia; ME Department of Medical and Surgical Sciences, University of Foggia, Foggia, Italy; MF School of Public Health, University College Cork, Cork, Ireland; MG Institute for Global Health Innovations, Duy Tan University, Hanoi, Viet Nam; MH Department of Environmental Health, Arak University of Medical Sciences, Arak, Iran; MI Road Traffic Injury Research Center, Tabriz University of Medical Sciences, Tabriz, Iran; MJ Department of Neurosurgery, National Brain Center in Indonesia, Jakarta, Indonesia; MK School of Medicine, University of Colima, Colima, Mexico; ML Department of Research, State Cancerology Institute of Colima, IMSS-BIENESTAR, Colima, Mexico; MM Department of Neurosurgery, University of Edinburgh, Edinburgh, Scotland; MN Department of Neurosurgery, National Health Service (NHS) Scotland, Edinburgh, Scotland; MO Department of Forensic Medicine, University of Sarajevo, Sarajevo, Bosnia and Herzegovina; MP Department of Public Health, Debre Tabor University, Debre Tabor, Ethiopia; MQ Department of Anesthesia, Wachemo University, Hossana, Ethiopia; MR Chettinad Hospital & Research Institute, Chettinad Academy of Research and Education, Chennai, India; MS JSS Medical College, Jagadguru Sri Shivarathreeswara Academy of Health Education and Research, Mysuru, India; MT Sheffield Teaching Hospitals NHS Foundation Trust, Sheffield, UK; MU Department of Pharmaceutical Regulatory Affairs and Management, Manipal Academy of Higher Education, Manipal, India; MV Department of Community Medicine, University of Peradeniya, Peradeniya, Sri Lanka; MW Population Interventions Unit, University of Melbourne, Melbourne, VIC, Australia; MX Faculty of Management, Bucharest University of Economic Studies, Bucharest, Romania; MY Care and Health Program, Care and Health Program, Yaounde, Cameroon; MZ National Institute for Research in Environmental Health, Indian Council of Medical Research, Bhopal, India; NA Department of Global Public Health, Karolinska Institute, Stockholm, Sweden; NB Department of Public Health, Jazan University, Jazan, Saudi Arabia; NC Institute of Medical Biostatistics, Epidemiology, and Informatics, Johannes Gutenberg University Mainz, Mainz, Germany; ND Cardio-Thoraco-Vascular Department, Azienda Sanitaria Universitaria Giuliano Isontina, Trieste, Italy; NE Department of Medical Laboratory Sciences, Iran University of Medical Sciences, Tehran, Iran; NF Independent Consultant, Bridgewater, NJ, USA; NG Department of Epidemiology, University of Pittsburgh, Pittsburgh, PA, USA; NH Department of Psychiatry, University of Pittsburgh Medical Center, Pittsburgh, PA, USA; NI Faculty of Science and Humanities, SRM Institute of Science and Technology, Kattankulathur, India; NJ Department of Dermatology, Venereology, and Leprology, Apollo Institute of Medical Sciences and Research Chittoor, Chittoor, India; NK School of Public Health, University of Adelaide, Adelaide, SA, Australia; NL Biotechnology Department, Suez Canal University, Ismailia, Egypt; NM Faculty of Science and Health, University of Portsmouth, Hampshire, UK; NN Department of Public Health and Community Medicine, Tanta University, Tanta City, Egypt; NO School of Public Health, Texila American University, Guyana, Guyana; NP Biomedical Informatics and Medical Statistics Department, Alexandria University, Alexandria, Egypt; NQ Department of Basic Medical Sciences, University of Sharjah, Sharjah, United Arab Emirates; NR Department of Anatomy and Embryology, Mansoura University, Mansoura, Egypt; NS Deanship of Preparatory Year and Supporting Studies, Imam Abdulrahman Bin Faisal University, Dammam, Saudi Arabia; NT Institute of Public Health, United Arab Emirates University, Al Ain, United Arab Emirates; NU Pharmacology and Therapeutic Department, United Arab Emirates University, Al Ain, United Arab Emirates; NV Department of Neurophysiology, Cairo University, Cairo, Egypt; NW College of Medicine, Korea University, Seoul, South Korea; NX Houston Methodist Hospital, Houston, TX, USA; NY Pediatric Dentistry and Dental Public Health Department, Alexandria University, Alexandria, Egypt; NZ College of Public Health and Health Informatics, King Saud Bin Abdulaziz University for Health Sciences, Riyadh, Saudi Arabia; OA King Abdullah International Medical Research Center, Riyadh, Saudi Arabia; OB Deanery of Biomedical Sciences, University of Edinburgh, Edinburgh, Scotland; OC Multiple Sclerosis Research Center, Tehran University of Medical Sciences, Tehran, Iran; OD Department of Epidemiology and Medical Statistics, University of Ibadan, Ibadan, Nigeria; OE Research Centre for Healthcare and Community, Coventry University, Coventry, UK; OF Tanner College of Dental Medicine, University of Pikeville, Pikeville, KY, USA; OG Department of Surgery, and Clinical and Public Health Research, MyungSung Medical College, Addis Ababa, Ethiopia; OH Bloomberg School of Public Health, Johns Hopkins University, Baltimore, MD, USA; OI Department of Health Policy and Administration, University of the Philippines Manila, Manila, Philippines; OJ Department of Medical Laboratory Technology, Jazan University, Jazan, Saudi Arabia; OK College of Medicine, AlMaarefa University, Riyadh, Saudi Arabia; OL Environmental Statistics Unit, National Institute of Statistics, Lisbon, Portugal; OM Ecological Economics and Environmental Management, NOVA University of Lisbon, Lisbon, Portugal; ON Department of Psychology, Federal University of Sergipe, São Cristóvão, Brazil; OO Department of Radiography and Imaging Technology, Green International University, Lahore, Pakistan; OP Centre for Public Health, Equity and Human Flourishing, Torrens University Australia, Adelaide, SA, Australia; OQ Institute of Resource Governance and Social Change, Kupang, Indonesia; OR School of Medicine, Shahed University, Tehran, Iran; OS Department of Social Medicine and Epidemiology, Guilan University of Medical Sciences, Rasht, Iran; OT Department of Infectious Diseases and Public Health, City University of Hong Kong, Hong Kong, China; OU Department of Pharmacy, Wollega University, Nekemte, Ethiopia; OV Department of Neurosurgery, Children's National Medical Center, Washington, DC, USA; OW Department of Neurosurgery, George Washington University, Washington, DC, USA; OX Department of Social Sciences, University of Nicosia, Nicosia, Cyprus; OY Institute of Public Health, Washington University in St. Louis, St Louis, MO, USA; OZ School of Medicine, Addis Ababa University, Addis Ababa, Ethiopia; PA Department of Pharmacology, Iranshahr University of Medical Sciences, Iranshahr, Iran; PB Department of Neuroscience, Multiple Sclerosis Research Center, Ravenna, Italy; PC Department of Biotechnological and Applied Clinical Sciences, University of L'Aquila, L'Aquila, Italy; PD Division of Clinical Epidemiology (KEP), Karolinska Institute, Stockholms, Sweden; PE College of Medicine, Dentistry and Public Health, James Cook University, Townsville, QLD, Australia; PF Russell H. Morgan Department of Radiology and Radiological Science, Johns Hopkins University, Baltimore, MD, USA; PG Department of Community Medicine, Bayero University Kano, Kano, Nigeria; PH Department of Community Medicine, Aminu Kano Teaching Hospital, Kano, Nigeria; PI Department of Public Health, University of Szeged, Szeged, Hungary; PJ Laboratory Sciences Division, National Institute of Epidemiology, Chennai, India; PK Trauma and Emergency Medicine Department, All India Institute of Medical Sciences (AIIMS), Madurai, Madurai, India; PL Infectious Diseases Unit, University of Verona, Verona, Italy; PM Department of Ophthalmology, University of Basel, Basel, Switzerland; PN School of Engineering and Applied Science, George Washington University, Washington, DC, USA; PO College of Medicine and Health Science, Bahir Dar University, Bahir Dar, Ethiopia; PP Department of Health Information Technology and Management, Shahid Beheshti University of Medical Sciences, Tehran, Iran; PQ Endocrine Research Center, Institute of Endocrinology and Metabolism, Iran University of Medical Sciences, Tehran, Iran; PR Department of Applied Health Sciences, University of Birmingham, Birmingham, England; PS Research Committee of Qom University of Medical Sciences, Qom University of Medical Sciences, Qom, Iran; PT Department of Medical Biochemical Analysis, Cihan University-Erbil, Erbil, Iraq; PU Neurology Department of Imam Khomeini Hospital, Tehran University of Medical Sciences, Tehran, Iran; PV School of Public Health, Qazvin University of Medical Sciences, Qazvin, Iran; PW Department of Radiology, University of Southern California, Los Angeles, CA, USA; PX International University of Health and Welfare, International University of Health and Welfare, Narita, Japan; PY Department of Nursing, Aksum University, Aksum, Ethiopia; PZ Department of Anesthesiology and Critical Care Medicine, Ospedale SS Annunziata Savigliano, Savigliano, Italy; QA Department of Clinical and Experimental Sciences, University of Brescia, Brescia, Italy; QB IRCSS Fondazione Don Gnocchi, IRCCS Fondazione Don Gnocchi, Rovato (Brescia), Italy; QC Department of Biostatistics, Tarbiat Modares University, Tehran, Iran; QD Quantitative Department, Non-Communicable Diseases Research Center (NCDRC), Tehran, Iran; QE Department of Health Systems and Policy Research, Indian Institute of Public Health, Gandhinagar, India; QF Department of Genetics, Sana Institute of Higher Education, Sari, Iran; QG Universal Scientific Education and Research Network (USERN), Kermanshah University of Medical Sciences, Kermanshah, Iran; QH Department of Family Medicine, Shenzhen University Medical School, Shenzhen, China; QI Shenzhen Clinical Research Center for Trauma Treatment, Shenzhen University, Shenzhen, China; QJ School of Medical Sciences, University of Hyderabad, Hyderabad, India; QK College of Applied Medical Sciences, University of Hail, Hail, Saudi Arabia; QL Department of Public Health and Preventive Medicine, Charles University, Prague, Czech Republic; QM Department of Epidemiology and Biostatistics, Anhui Medical University, Hefei, China; QN Medical Directorate, Local Health Authority of Ferrara, Ferrara, Italy; QO Department of Clinical Science, University Of Sulaimani, Sulaimani, Iraq; QP Department of Community Medicine, University of Peradeniya, Kandy, Sri Lanka; QQ Department of Toxicology, Shriram Institute for Industrial Research, Delhi, India; QR Department of Anaesthesia, All India Institute of Medical Sciences, Bilaspur, India; QS Doctoral Program in Biomedical Gerontology, Pontifical Catholic University of Rio Grande Do Sul, Porto Alegre, Brazil; QT Neurosurgery Department, Fasa University of Medical Sciences, Shiraz, Iran; QU Bloomberg School of Public Health, Johns Hopkins University, Arlington, VA, USA; QV Department of Hepato-Pancreatico-Biliary Surgery, Thong Nhat Hospital, Ho Chi Minh City, Viet Nam; QW Department of Orthopedics and Trauma Surgery, Addis Ababa University, Addis Ababa, Ethiopia; QX Department of Community Medicine, Post Graduate Institute of Medical Education and Research, Chandigarh, India; QY Centre for Community Medicine, All India Institute of Medical Sciences, New Delhi, India; QZ Department of Family and Community Medicine, Arabian Gulf University, Manama, Bahrain; RA Department of Statistics and Informatics, Salahaddin University-Erbil, Erbil, Iraq; RB Department of Neurosurgery, Mayo Clinic, Rochester, USA; RC Sakarya University, Sakarya, Türkiye; RD Department of Critical Care and Emergency Nursing, Zanjan University of Medical Sciences, Zanjan, Iran; RE Department of Computer and Information Science, Majmaah University, Al Majmaah, Saudi Arabia; RF Department of Epidemiology, Biostatistics, Population Studies and Health Promotion, Faculty of Public Health, Universitas Airlangga, Surabaya, Indonesia; RG Department of Medical Imaging, University of Toronto, Toronto, ON, Canada; RH Department of Ophthalmology, Alborz University of Medical Sciences, Karaj, Iran; RI Department of Health Management, Policy and Economics, Shahid Beheshti University of Medical Sciences, Tehran, Iran; RJ Research Center for Traditional Medicine and History of Medicine, Shiraz University of Medical Sciences, Shiraz, Iran; RK Department of Periodontics, RAK Medical and Health Sciences University, Ras Al Khaimah, United Arab Emirates; RL Department of Oral Rehabilitation, University of Khartoum, Khartoum, Sudan; RM Department of Medicine, University of Khartoum, Khartoum, Sudan; RN Department of Community Medicine, Federal University Teaching Hospital, Lafia, Nigeria; RO Department of Epidemiology and Community Medicine, Federal University of Lafia, Lafia, Nigeria; RP Faculty of Kinesiology, University of New Brunswick, Fredericton, NB, Canada; RQ School of Allied Health, Murdoch University, Murdoch, WA, Australia; RR Community-Oriented Nursing Midwifery Research Center, Shahrekord University of Medical Sciences, Shahrekord, Iran; RS Poostchi Ophthalmology Research Center, Shiraz University of Medical Sciences, Shiraz, Iran; RT Faculty of Health, University of Canterbury, Christchurch, New Zealand; RU Kasturba Medical College Mangalore, Manipal Academy of Higher Education, Manipal, India; RV Department of Statistics, Shahjalal University of Science and Technology, Sylhet, Bangladesh; RW Department of Public Health, University of Science and Technology Chittagong (USTC), Chattogram, Bangladesh; RX Department of Public Health, Daffodil International University (DIU), Dhaka, Bangladesh; RY School of Engineering and Technology, Duy Tan University, Da Nang, Viet Nam; RZ International Center for Materials Sciences and Technology, Western Caspian University, Baku, Azerbaijan; SA Department of Legal Medicine and Bioethics, Carol Davila University of Medicine and Pharmacy, Bucharest, Romania; SB Department of Clinical Legal Medicine, National Institute of Legal Medicine Mina Minovici, Bucharest, Romania; SC Faculty of Medicine, The Chinese University of Hong Kong, Hong Kong, China; SD Department of Public Health and Community Medicine, Shaikh Zayed Postgraduate Medical Institute, Lahore, Pakistan; SE Department of Humanities, COMSATS University Islamabad, Islamabad, Pakistan; SF Czech National Centre for Evidence-Based Healthcare and Knowledge Translation, Masaryk University, Brno, Czech Republic; SG Institute of Biostatistics and Analyses, Masaryk University, Brno, Czech Republic; SH Department of Health Promotion and Education, University of Ibadan, Ibadan, Nigeria; SI Department of Physiotherapy, Tishk International University, Erbil, Iraq; SJ Nursing Faculty, Mansoura University, Mansoura, Egypt; SK RAK Medical and Health Science University, RAK College of Nursing, Arak University of Medical Sciences, RAK, United Arab Emirates; SL Scientific and Technological Park, Kazakh National Medical University, Almaty, Kazakhstan; SM West Africa RCC, Africa Centre for Disease Control and Prevention, Abuja, Nigeria; SN Department of Community Medicine, University College Hospital, Ibadan, Ibadan, Nigeria; SO Faculty of Medicine, University of Belgrade, Belgrade, Serbia; SP Faculty of Medical Sciences, University of Kragujevac, Kragujevac, Serbia; SQ Institute of Health Research, University of Health and Allied Sciences, Ho, Ghana; SR National Research and Innovation Agency, Jakarta, Indonesia; SS College of Nursing, Onaizah Colleges, Qassim, Saudi Arabia; ST University of Michigan, Ann Arbor, MI, USA; SU Faculty of Pharmacy, Universitas Ahmad Dahlan, Yogyakarta, Indonesia; SV School of Pharmacy, BRAC University, Dhaka, Bangladesh; SW Department of Public Health, International University of Business Agriculture and Technology, Dhaka, Bangladesh; SX 4-Green Research Society, Bangladesh, Dhaka, Bangladesh; SY School of Health Systems and Public Health, University of Washington, Seattle, WA, USA; SZ Department of Zoology, University of Ilorin, Ilorin, Nigeria; TA One Health Research Group, Universidad De Las Américas, Quito, Ecuador; TB College of Nursing, All India Institute of Medical Sciences, Bhubaneswar, India; TC Department of Physical Medicine and Rehabilitation, Université Paris Cité, Paris, France; TD Research and Development Unit, Biomedical Research Networking Center for Mental Health Network (CiberSAM), Barcelona, Spain; TE Department of Health Studies, University of Richmond, Richmond, VA, USA; TF Department of Nursing, Arak University of Medical Sciences, Arak, Iran; TG Section of Economic & Social Sciences, Humanities & Arts, The World Academy of Sciences UNESCO-TWAS, Trieste, Italy; TH Shaanxi University of Technology, Hanzhong, China; TI Department of Environmental Engineering, Islamic Azad University, Ahvaz, Iran; TJ SRM Medical College Hospital and Research Centre, Sri Ramaswamy Memorial Institute of Science and Technology, Kattankulathur, India; TK Department of Neurosciences, University of the Philippines Manila, Manila, Philippines; TL Institute for Neurosciences, St. Luke's Medical Center, Bonifacio Global City, Philippines; TM SRM College of Physiotherapy, SRM Institute of Science and Technology (SRMIST), Kattankulathur, Tamil Nadu, India; TN Division of Medical Research, Sri Ramaswamy Memorial Institute of Science and Technology, Kattankulathur, India; TO Department of Neurosurgery, Research Institute at Nationwide Children's Hospital, Rawalpindi, Pakistan; TP Department of Pharmacology, Imam Mohammad Ibn Saud Islamic University, Riyadh, Saudi Arabia; TQ The Medical City for Military and Security Services School, Muscat, Oman; TR Department of Oral Medicine and Periodontology, University of Peradeniya, Peradeniya, Sri Lanka; TS Department of Oral Medicine and Periodontology, Saveetha University, Chennai, India; TT Postgraduate Institute of Medicine, University of Colombo, Colombo, Sri Lanka; TU Faculty of Graduate Studies, Institute for Violence and Injury Prevention, Colombo, Sri Lanka; TV Department of Biomedical Informatics, Korea University, Seoul, South Korea; TW School of Medicine, Johns Hopkins University, Baltimore, MD, USA; TX Department of Community Medicine, Dr. Baba Saheb Ambedkar Medical College & Hospital, Delhi, India; TY Department of Community Medicine, Banaras Hindu University, Varanasi, India; TZ Department of Orthopedic Surgery, University of Alabama at Birmingham, Birmingham, AL, USA; UA Rothschild Foundation Hospital, Rothschild Foundation Hospital, Paris, France; UB Singapore Eye Research Institute, Singapore Eye Research Institute, Singapore, Singapore; UC Health Data Science and Artificial Intelligence Centre, Health Services Management Training Centre, Faculty of Health and Public Administration, Semmelweis University, Budapest, Hungary; UD Hungarian Health Management Association, Budapest, Hungary; UE School of Engineering and Technology, The University of New South Wales, Canberra, NSW, Australia; UF Department of Community Medicine, Manipal Academy of Higher Education, Mangalore, India; UG Faculty of Applied & Basic Sciences, SGT University of Medical Sciences, Gurugram, India; UH Institute of National Importance, All India Institute of Medical Sciences, Rajkot, India; UI Department of Economics, National Open University, Benin City, Nigeria; UJ School of Computer Science and Engineering, RV University, Bengaluru, India; UK Research Department, TobaccoFree Research Institute Ireland, Dublin, Ireland; UL Department of Statistics, Salahaddin University-Erbil, Erbil, Iraq; UM Department of Business Administrations, Cihan University-Erbil, Erbil, Iraq; UN Physiotherapy Department, Baylor University, Kano, Nigeria; UO Department of Physiotherapy, University of KwaZulu-Natal, Durban, South Africa; UP Department of Pharmacology, Post Graduate Institute of Medical Education and Research, Chandigarh, India; UQ School of Management and Medical Informatics, Tabriz University of Medical Sciences, Tabriz, Iran; UR Department of Health, Khoy Medical Sciences, Khoy, Iran; US Prasanna School of Public Health, Manipal Academy of Higher Education, Manipal, India; UT Care and Public Health Research Institute (CAPHRI), Maastricht University, Maastricht, Netherlands; UU Elderwood Rehabilitation Clinical Centre, Buffalo, NY, USA; UV Department of Public Health, South Wales University, Treforest, UK; UW School of Health and Environmental Science, Korea University, Seoul, South Korea; UX Department of Anesthesia, Critical Care and Pain Medicine, Massachusetts General Hospital, Boston, MA, USA; UY Office of the Executive Director, Cephas Health Research Initiative Inc., Ibadan, Nigeria; UZ Department of Public Health, Thomas Adewumi University, Oko, Nigeria; VA The Hansjörg Wyss Department of Plastic and Reconstructive Surgery, New York University, New York, NY, USA; VB Department of Analytical & Applied Economics, Utkal University, Bhubaneswar, India; VC School of Health Professions and Human Services, Hofstra University, Hempstead, NY, USA; VD Department of Anesthesiology, Montefiore Medical Center, Bronx, NY, USA; VE Minimally Invasive Surgery Research Center, Iran University of Medical Sciences, Tehran, Iran; VF Artificial Intelligence in Health Research Center, Iran University of Medical Sciences, Tehran, Iran; VG Department of Medical-Surgical Nursing, Guilan University of Medical Sciences, Rasht, Iran; VH Saveetha Medical College and Hospital, Saveetha University, Chennai, India; VI Department of Physical Therapy and Health Rehabilitation, Majmaah University, Al Majmaah, Saudi Arabia; VJ Department of Surgery, MyungSung Medical College, Addis Ababa, Ethiopia; VK Surgery Research Unit, University of Oulu, Oulu, Finland; VL Department of Molecular Medicine and Surgery, Karolinska Institute, Stockholm, Sweden; VM Medical Basic Sciences Research Institute, Ahvaz Jundishapur University of Medical Sciences, Ahvaz, Iran; VN Student Research Committee, Ahvaz Jundishapur University of Medical Sciences, Ahvaz, Iran; VO Open, Distance and eLearning Campus, University of Nairobi, Nairobi, Kenya; VP Department of Public Health, Jordan University of Science and Technology, Irbid, Jordan; VQ Department of Public Health, Ahvaz Jundishapur University of Medical Sciences, Ahvaz, Iran; VR Environmental Technologies Research Center, Medical Basic Sciences Research Institute, Ahvaz Jundishapur University of Medical Sciences, Ahvaz, Iran; VS Natural and Medical Sciences Research Center, University of Nizwa, Nizwa, Oman; VT Dr Sulaiman Al Habib Hospital, Sheri Kashmir Institute of Medical Sciences, Riyadh, Saudi Arabia; VU University Institute of Public Health, The University of Lahore, Lahore, Pakistan; VV Epidemiology Program, Jazan University, Jazan, Saudi Arabia; VW Department of Community Medicine, National Institute of Preventive and Social Medicine, Dhaka, Bangladesh; VX Department of Pharmacy, Minhaj University Lahore, Lahore, Pakistan; VY International Center for Chemical and Biological Sciences, International Center for Chemical and Biological Sciences, Karachi, Pakistan; VZ Centre for Interdisciplinary Research in Basic Sciences, Jamia Millia Islamia, New Delhi, India; WA Department of Emergency Medicine, Aga Khan University, Karachi, Pakistan; WB College of Medicine, University of Hail, Hail, Saudi Arabia; WC Department of Cardiology, University of South Wales, Treforest, UK; WD Department of Cardiology, University of Buckingham, Buckingham, UK; WE Central Department of Zoology, Tribhuvan University, Kathmandu, Nepal; WF Department of Pharmacology, All India Institute of Medical Sciences, Raipur, India; WG Faculty of Nursing, Yarmouk University, Irbid, Jordan; WH Department of Basic Medical Sciences, Yarmouk University, Irbid, Jordan; WI Department of Orthopaedics, Postgraduate Medical Institute, Sangrur, India; WJ Department of Neurosurgery, Shahid Beheshti University of Medical Sciences, Tehran, Iran; WK Asadabad School of Medical Sciences, Asadabad School of Medical Sciences, Asadabad, Iran; WL Department of Neurosurgery, Mashhad University of Medical Sciences, Mashhad, Iran; WM Social Development and Health Promotion Research Center, Kermanshah University of Medical Sciences, Kermanshah, Iran; WN Department of Biochemistry and Nutrition, Neyshabur University of Medical Sciences, Neyshabur, Iran; WO Institute of Research and Development, Duy Tan University, Da Nang, Viet Nam; WP School of Engineering & Technology, Duy Tan University, Da Nang, Viet Nam; WQ Department of Public Health, New Mexico State University, Las Cruces, NM, USA; WR College of Medicine and Health Sciences, United Arab Emirates University, Al Ain, United Arab Emirates; WS School of Health Sciences, Kristiania University College, Oslo, Norway; WT Department of International Health and Sustainable Development, Tulane University, New Orleans, LA, USA; WU Department of Medical Sciences, Uppsala University Hospital, Uppsala, Sweden; WV Department of Public Health Dentistry, Krishna Vishwa Vidyapeeth (Deemed to Be University), Karad, India; WW Department of Public Health and Community Medicine, Central University of Kerala, Kasaragod, India; WX Center for Tobacco Control Research and Education, University of California San Francisco, San Francisco, CA, USA; WY Social Determinants of Health Research Center, Shahid Beheshti University of Medical Sciences, Tehran, Iran; WZ Children's Medical Center, Tehran University of Medical Sciences, Tehran, Iran; XA College of Medicine and Health Science, Werabe University, Werabe, Ethiopia; XB Department of Science and Environmental Studies, The Education University of Hong Kong, Hong Kong, China; XC School of Post Graduate, Amoud University, Hargeisa, Somalia; XD Feinberg School of Medicine, Northwestern University, Chicago, IL, USA; XE Department of Anthropology, Panjab University, Chandigarh, India; XF Department of Forensic Medicine and Toxicology, Pondicherry University, Puducherry, India; XG Department of Medical Education and Informatics, Gazi University Faculty of Medicine, Ankara, Türkiye; XH Department of Biochemistry, University of Hail, Hail, Saudi Arabia; XI Department of Research Management, Research Institute of Cardiology and Internal Diseases, Almaty, Kazakhstan; XJ Department of Health Administration and Education, University of Education, Winneba, Winneba, Ghana; XK Amity Institute of Health Allied Sciences, Amity University Noida, Uttar Pradesh, Noida, India; XL Department of Community Medicine, Rajendra Institute of Medical Sciences, Ranchi, India; XM Department of Mathematics, Amity University Haryana, Gurugram, India; XN Wits Advanced Drug Delivery Platform Research Unit, University of the Witwatersrand, Johannesburg, South Africa; XO Wits Infectious Diseases and Oncology Research Institute, University of the Witwatersrand, Johannesburg, South Africa; XP Monitoring & Evaluation, IGP Health Initiatives Pvt. Ltd., Delhi, India; XQ Department of Paediatrics, Sri Ramaswamy Memorial Institute of Science and Technology, Chennai, India; XR Department of Health Management, University of Hail, Hail, Saudi Arabia; XS Faculty of Education, Shree Guru Gobind Singh Tricentenary University, Gurugram, India; XT Department of Economics, Central University of Haryana, Jant-Pali, India; XU Department of Anaesthesiology, Rajendra Institute of Medical Sciences, Ranchi, India; XV Lincoln Institute of Rural and Coastal Health, University of Lincoln, Lincoln, England; XW Department of Medicine, McMaster University, Hamilton, ON, Canada; XX National Research and Innovation Agency, Bogor, Indonesia; XY Department of Public Health and Epidemiology, Khalifa University of Science and Technology, Abu Dhabi, United Arab Emirates; XZ Faculty of Public Health, University of Indonesia, Depok, Indonesia; YA Clinical Research Center, Turku University Hospital, Turku, Finland; YB Heart Center, University of Turku and Turku University Hospital, Turku, Finland; YC Kasturba Medical College, Manipal, Manipal Academy of Higher Education, Manipal, India; YD Department of Clinical Sciences and Community Health, University of Milan, Milan, Italy; YE Department of Nursing Science, Bayero University Kano, Kano, Nigeria; YF Institute for Social and Health Sciences, University of South Africa, Pretoria, South Africa; YG Division of Evidence Synthesis, Foundation for People-centric Health Systems, New Delhi, India; YH Division of Lifestyle Medicine, Centre for Health: The Specialty Practice, New Delhi, India; YI Department of Psychology, University of Helsinki, Helsinki, Finland; YJ Department of Psychology, Norwegian University of Science and Technology, Trondheim, Norway; YK Department of Physiotherapy, Universitas Aisyiyah Yogyakarta, Yogyakarta, Indonesia; YL Institute of Allied Health Sciences, National Cheng Kung University, Tainan, Taiwan; YM Department of Medical Physics and Informatics, University of Szeged, Szeged, Hungary; YN Department of Otorhinolaryngology, Father Muller Medical College, Mangalore, India; YO University Institute of Radiological Sciences and Medical Imaging Technology, The University of Lahore, Lahore, Pakistan; YP International Society of Doctors for the Environment, Arezzo, Italy; YQ Department of Clinical and Experimental Medicine, University of Catania, Catania, Italy; YR School of Psychology, Social Work and Public Health, Oxford Brookes University, Oxford, UK; YS Brookes Epidemiological Surveillance Taskforce, Oxford, UK; YT Graduate School of Transdisciplinary Health Sciences, Yonsei University, Seoul, South Korea; YU Department of Precision Medicine, Sungkyunkwan University, Suwon-si, South Korea; YV Department of Family Medicine, University of Texas Medical Branch, Galveston, TX, USA; YW Department of Preventive Medicine, Korea University, Seoul, South Korea; YX Department of Rehabilitation Medicine, Seoul National University Hospital, Seoul, South Korea; YY Traffic Injury Rehabilitation Research Institute, National Traffic Injury Rehabilitation Hospital, Yangpyeong, South Korea; YZ Department of Cardiothoracic and Vascular Surgery, Westpfalz Klinikum, Kaiserslautern, Germany; ZA Department of Cardiothoracic Surgery, University of Patras, Patras, Greece; ZB Department of Pharmacy, Wachemo University, Hosanna, Ethiopia; ZC Department of Health Promotion and Health Education, National Taiwan Normal University, Taipei, Taiwan; ZD Department of Health Management Center, Fudan University, Shanghai, China; ZE School of Nursing, Hong Kong Polytechnic University, Hong Kong, China; ZF School of Medicine, Shanghai Jiao Tong University, Shanghai, China; ZG Clinical Sciences Department, Shaqra University, Sharqa, Saudi Arabia; ZH Lerner Research Institute, Cleveland Clinic, Cleveland, OH, USA; ZI Department of Quantitative Health Science, Case Western Reserve University, Cleveland, OH, USA; ZJ School of Medicine, Universidad Espíritu Santo, Samborondón, Ecuador; ZK Vicerrectoría De Investigación Y Postgrado, Universidad De Los Lagos, Osorno, Chile; ZL Department of Epidemiology and Evidence-Based Medicine, I.M. Sechenov First Moscow State Medical University, Moscow, Russia; ZM Ashok & Rita Patel Institute of Physiotherapy, Charotar University of Science and Technology, Anand, India; ZN Department of Community and Family Medicine, All India Institute of Medical Sciences, Bathinda, India; ZO Dodoma Medical Research Centre, National Institute for Medical Research in Tanzania, Dodoma, Tanzania; ZP College of Engineering, Effat University, Jeddah, Saudi Arabia; ZQ Management of Information Systems Department, The American College of Greece, Aghia Paraskevi, Greece; ZR Department of Medicine, University of Alberta, Edmonton, AB, Canada; ZS Tulane School of Public Health and Tropical Medicine, Tulane University, New Orleans, LA, USA; ZT Centre for Public Health and Wellbeing, University of the West of England, Bristol, England; ZU Department of Periodontology, Pomeranian Medical University, Szczecin, Poland; ZV Anesthesiology Research Center, Shahid Beheshti University of Medical Sciences, Tehran, Iran; ZW College of Dentistry, University of Hail, Hail, Saudi Arabia; ZX Thamar University, Dhamar, Yemen; ZY Mansoura Faculty of Medicine, Mansoura University, Mansoura, Egypt; ZZ Ophthalmology Department, Dr. Soliman Fakeeh Hospital, Riyadh, Saudi Arabia; AAA Department of Emergency Medicine and Trauma, Karpaga Vinayaga Educational Lore for Learning (Deemed-to-be-University), TamilNadu, India; AAB Department of One Health in Tropical Infectiousness Diseases, Jigjiga University, Jigjiga, Ethiopia; AAC College of Health Science, Amoud University, Borama, Somalia; AAD Research Center, Cihan University-Sulaimaniya, Sulaymaniyah, Iraq; AAE Department of Primary Care and Public Health, Imperial College London, London, England; AAF Kurdistan University of Medical Sciences, Kurdistan University of Medical Sciences, Sanandaj, Iran; AAG INSERM, Imaging Brain & Neuropsychiatry iBraiN, Université De Tours, Tours, France; AAH Service De Médecine Et Chirurgie Bucco-Dentaire, Tours University Hospital, Tours, France; AAI Department of Maternal-Child Nursing and Public Health, Federal University of Minas Gerais, Belo Horizonte, Brazil; AAJ Internal Medicine Department, MedStar Health, Washington, DC, USA; AAK Department of Non-communicable Diseases and Mental Health, Pan American Health Organization, Washington, DC, USA; AAL Department of Community Medicine, Sri Balaji Vidyapeeth (Deemed to Be University), Pondicherry, India; AAM Faculty of Humanities and Health Sciences, Curtin University, Sarawak, Malaysia; AAN Jeffrey Cheah School of Medicine and Health Sciences, Monash University, Subang Jaya, Malaysia; AAO Medical Scientist Training Program, Northwestern University, Chicago, IL, USA; AAP Economics Department, University of Oklahoma, Norman, OK, USA; AAQ Department of Anatomy and Developmental Biology, Monash University, Clayton, VIC, Australia; AAR Department of Anatomy, Genetics and Biomedical Informatics, University of Colombo, Colombo, Sri Lanka; AAS Department of Community Medicine, Apollo Institute of Medical Sciences and Research, Hyderabad, India; AAT Department of Prosthetic Dental Sciences, Jazan University, Jazan, Saudi Arabia; AAU Digital Health and Informatics Directorate, Queensland Health, Brisbane, QLD, Australia; AAV Department of Public Health, Arba Minch University, Arba Minch, Ethiopia; AAW College of Medicine and Health Science, Wachemo University, Hosanna, Ethiopia; AAX College of Medicine and Department of Nursing, Komar University of Science and Technology, Kurdistan Sulemaniya, Iraq; AAY General Practice, Monash University, Melbourne, VIC, Australia; AAZ School of Medicine and Institute of Public Health, University of Gondar, Gondar, Ethiopia; ABA Department of Civil Engineering, Federal University of Lavras, Lavras, Brazil; ABB Dirección General De Investigación, Desarrollo E Innovación (DGIDI), Universidad Científica Del Sur (University of the South), Lima, Peru; ABC Department of Medical Microbiology and Immunology, Trinity Medical Sciences University, St. Vincent, Saint Vincent and the Grenadines; ABD Kasturba Medical College, Manipal Academy of Higher Education, Mangalore, India; ABE Department of Pathology, Imam Abdulrahman Bin Faisal University, Dammam, Saudi Arabia; ABF Department of Adult Health Nursing, Bahir Dar University, Bahir Dar, Ethiopia; ABG NUST School of Health Sciences, National University of Science and Technology (NUST), Islamabad, Pakistan; ABH Comprehensive Cancer Center, Helsinki University Hospital, Helsinki, Finland; ABI University of Helsinki, Helsinki, Finland; ABJ University Centre Varazdin, University North, Varazdin, Croatia; ABK Department of Pharmacology, University of Kelaniya, Ragama, Sri Lanka; ABL Clinical Medicine Department, Colombo North Teaching Hospital, Ragama, Sri Lanka; ABM Department of Paediatrics, University of Kelaniya, Ragama, Sri Lanka; ABN University Paediatrics Unit, Colombo North Teaching Hospital, Ragama, Sri Lanka; ABO Department of Pathology, Zagazig University, Zagazig, Egypt; ABP Clinical Emergency Department, Pomeranian Medical University, Szczecin, Poland; ABQ Pomeranian Medical University, Szczecin, Poland; ABR College of Human Medicine, Michigan State University, Flint, MI, USA; ABS Multidisciplinary Department of Medical-Surgical and Dental Specialties, University of Campania Luigi Vanvitelli, Naples, Italy; ABT Saveetha Dental College and Hospitals, Saveetha University, Chennai, India; ABU School of Pharmacy, Sunway University, Sunway City, Malaysia; ABV Department of Forensic Medicine and Toxicology, All India Institute of Medical Sciences, Patna, India; ABW Milken Institute School of Public Health, George Washington University, Washington, DC, USA; ABX RAK College of Nursing, RAK Medical and Health Sciences University, Ras Al-Khaimah, United Arab Emirates; ABY Nursing College, Sohag University, Sohag, Egypt; ABZ Molecular Biology Unit, Sirius Training and Research Centre, Khartoum, Sudan; ACA Bio-Statistical and Molecular Biology Department, Sirius Training and Research Centre, Khartoum, Sudan; ACB Faculty of Medicine, University of Khartoum, Khartoum, Sudan; ACC Department of Pathophysiology, St.George's University, School of Medicine, St. George's University, St.George's, Grenada; ACD Department of Biophysics, All India Institute of Medical Sciences, New Delhi, India; ACE Centre For Interdisciplinary Research In Basic Sciences (CIRBSc), Jamia Millia Islamia, New Delhi, India; ACF Department of Information Technology, Lebanese French University, Erbil, Iraq; ACG Center of Excellence in Precision Medicine and Digital Health, Chulalongkorn University, Bangkok, Thailand; ACH Modeling in Health Research Center, Shahrekord University of Medical Sciences, Shahrekord, Iran; ACI Health Systems and Policy Research Unit, Ahmadu Bello University, Zaria, Nigeria; ACJ Heidelberg University Hospital and Faculty of Medicine, Heidelberg University, Heidelberg, Germany; ACK Department of Health Services Management, Iran University of Medical Sciences, Iran, Iran; ACL Department of Health Services Management, Isfahan University of Medical Sciences, Isfahan, Iran; ACM AI & Cyber Futures Institute, Charles Sturt University, Bathurst, NSW, Australia; ACN The University of Queensland, Brisbane, QLD, Australia; ACO Health Systems Research Center, National Health Research Institutes, Cuernavaca, Mexico; ACP Department of Global Health and Population, Harvard University, Boston, MA, USA; ACQ Department of Public Health, Oswaldo Cruz Foundation, Recife, Brazil; ACR Department of Public Health, Federal University of Pernambuco, Recife, Brazil; ACS Transplant Institute, Georgetown University, Washington, DC, USA; ACT Division of Plastic and Reconstructive Surgery, University of Washington Medical Center, Seattle, WA, USA; ACU American Society for Inclusion, Diversity, and Equity in Healthcare, Delaware, DE, USA; ACV Antimicrobial Resistance Research Center, Iran University of Medical Sciences, Tehran, Iran; ACW Hazrat-e Rasool General Hospital, Iran University of Medical Sciences, Tehran, Iran; ACX Unit of Pharmacotherapy, Epidemiology and Economics, University of Groningen (Rijksuniversiteit Groningen), Groningen, Netherlands; ACY Department of Epidemiology and Biostatistics, Wuhan University, Wuhan, China; ACZ College of Nursing, Dr. Ram Manohar Lohia Institute of Medical Sciences, Lucknow, India; ADA College of Medicine and Health, University of Birmingham, Birmingham, England; ADB Department of Physiotherapy, Tishk International University, Sulaimaniyah, Iraq; ADC Knowledge Management Department, Prahlad Omkarwati Foundation (POF), Mumbai, India; ADD Changescape Consulting, Changescape Consulting, New Delhi, India; ADE Department of Surgery, General University Hospital of Patras, Patras, Greece; ADF Faculty of Medicine, University of Thessaly, Larissa, Greece; ADG Department of Health Economics, National Institute for Research in Tuberculosis, Chennai, India; ADH Clinical Epidemiology Research Unit, Mexican Institute of Social Security, Villa de Alvarez, Mexico; ADI Postgraduate in Medical Sciences, Universidad De Colima, Colima, Mexico; ADJ Centre for Early Childhood Caries Research, Sri Ramachandra University, Chennai, India; ADK Santosh University, New Delhi, India; ADL Faculty of Physiotherapy, Sigma University, Anand, India; ADM Amoud University, Amoud University, Borama, Somalia; ADN School of Public Health, Makerere University, Kampala, Uganda; ADO Department of Research Methods, Orthopaedic Research Group, Coimbatore, India; ADP Central Research Laboratory, Vinayaka Mission's Research Foundation (Deemed to Be University), Puducherry, India; ADQ Elderly Health Research Center, Research and Academic Institution, Tehran, Iran; ADR Department of Computer Science and IT, Torrens University, Adelaide, SA, Australia; ADS Department of Health and Rehabilitation Sciences, Prince Sattam Bin Abdulaziz University, Al Kharj, Saudi Arabia; ADT Suraj Eye Institute, Nagpur, India; ADU Nursing & Midwifery Research Department (NMRD), Hamad Medical Corporation, Doha, Qatar; ADV Division of Endocrinology and Diabetes, University of Vermont, South Burlington, VT, USA; ADW Department of Dental Public Health, King Abdulaziz University, Jeddah, Saudi Arabia; ADX King Fahd Medical Research Center (KFMRC), Entrepreneurship & Innovation Unit, King Abdulaziz University, Jeddah, Saudi Arabia; ADY Department of Circulation and Medical Imaging, Norwegian University of Science and Technology, Trondheim, Norway; ADZ Department of Biological Sciences and Chemistry (DBSC), University of Nizwa, Nizwa, Oman; AEA Amity Institute of Forensic Sciences, Amity University, Noida, India; AEB College of Public Health, Kent State University, Kent, OH, USA; AEC Department of Pharmacy, The University of Lahore, Sargodha, Pakistan; AED Department of Molecular Biology and Chemistry, Sargodha Medical College, Sargodha, Pakistan; AEE Department of Pediatrics, Arak University of Medical Sciences, Arak, Iran; AEF TroDDIVaT Initiative, Frontline Association, Buea, Cameroon; AEG Department of Microbiology and Parasitology, University of Buea, Buea, Cameroon; AEH College of Medicine and Health Sciences, University of Gondar, Gondar, Ethiopia; AEI Department of General Surgery, Carol Davila University of Medicine and Pharmacy, Bucharest, Romania; AEJ Department of General Surgery, Emergency University Hospital Bucharest, Bucharest, Romania; AEK Department of Community Medicine, Lumbini Medical College, Palpa, Nepal; AEL Institute for Global Health Innovations, Duy Tan University, Da Nang, Viet Nam; AEM Faculty of Public Health, VNU University of Medicine and Pharmacy, Hanoi, Viet Nam; AEN Faculty of Medicine, Duy Tan University, Da Nang, Viet Nam; AEO Department of General Surgery, University Hospital of Heraklion, Heraklion, Greece; AEP Laboratory of Toxicology, University of Crete, Heraklion, Greece; AEQ Department of Epidemiology and Health Statistics, Central South University, Changsha, China; AER Public Health Department, Universitas Negeri Semarang (State University of Semarang), Kota Semarang, Indonesia; AES School of Medicine, University of Limerick, Limerick, Ireland; AET School of Health and Wellbeing, University of Glasgow, Glasgow, Scotland; AEU Department of Global Health Policy and Development, Sub-Saharan Africa Research for Sustainable Development (SSARSD) Project, Berekum, Ghana; AEV Global Health Policy Lab, Tohoku University, Miyagi, Japan; AEW Department of Health Policy and Management, Keio University, Tokyo, Japan; AEX Department of Paediatrics, Nnamdi Azikiwe University, Awka, Nigeria; AEY Global Health Department, Euclid University, Banqui, Central African Republic; AEZ Center of Excellence in Reproductive Health Innovation (CERHI), University of Benin, Benin City, Nigeria; AFA Department of Physiology, University of Benin, Edo, Nigeria; AFB Department of Physiology, Benson Idahosa University, Benin City, Nigeria; AFC Department of Applied Economics and Quantitative Analysis, University of Bucharest, Bucharest, Romania; AFD Bioinformatics Department, National Institute of Research and Development for Biological Sciences, Bucharest, Romania; AFE Department of Epidemiology, Johns Hopkins University, Baltimore, MD, USA; AFF Department of Biomedicine and Prevention, University of Rome “Tor Vergata”, Rome, Italy; AFG Department of Veterinary Public Health and Preventive Medicine, University of Ilorin, Ilorin, Nigeria; AFH Translational Health Research Institute, Western Sydney University, Sydney, NSW, Australia; AFI Department of Physiotherapy, Federal University of Health Sciences Ila Orangun Osun, Ila-Orangun, Nigeria; AFJ Health Promotion Research Center, Zahedan University of Medical Sciences, Zahedan, Iran; AFK School of Pharmacy, University of the Western Cape, Cape Town, South Africa; AFL College of Health Sciences, Bowen University, Iwo, Nigeria; AFM College of Medicine, University of Ibadan, Ibadan, Nigeria; AFN Department of Psychiatry and Behavioural Neurosciences, McMaster University, Hamilton, ON, Canada; AFO Department of Psychiatry, University of Lagos, Lagos, Nigeria; AFP Department of Community Medicine, Ahmadu Bello University, Zaria, Nigeria; AFQ School of Health and Life Sciences, Teesside University, Middlesbrough, UK; AFR Jadara Research Center, Jadara University, Irbid, Jordan; AFS Surgery Department, Sulaimani University, Sulaimani, Iraq; AFT ENT Department, Tor Vergata University of Rome, Rome, Italy; AFU Centre of Regenerative Medicine, Medical University of Bialystok, Bialystok, Poland; AFV Institute of Family Medicine and Public Health, University of Tartu, Tartu, Estonia; AFW Section of Sustainable Health, Umeå University, Umea, Sweden; AFX Department of Biological Sciences, Njala University, Freetown, Sierra Leone; AFY Department of Allied Health Sciences, Jordan University of Science and Technology, Irbid, Jordan; AFZ Department of Statistics and Econometrics, Bucharest University of Economic Studies, Bucharest, Romania; AGA Faculty of Medicine, University Ferhat Abbas of Setif, Sétif, Algeria; AGB Division of Infectious Diseases, University Hospital of Setif, Sétif, Algeria; AGC Department of Medicine, University of Ibadan, Ibadan, Nigeria; AGD Department of Medicine, University College Hospital, Ibadan, Ibadan, Nigeria; AGE Division of Medicine, University College London, London, England; AGF School of Medicine, Keele University, Keele, England; AGG Department of Respiratory Medicine, Jagadguru Sri Shivarathreeswara Academy of Health Education and Research, Mysore, India; AGH Department of Physical Medicine and Rehabilitation, Harvard University, Boston, MA, USA; AGI Universidad San Ignacio De Loyola, Lima, Peru; AGJ Department of Forensic Medicine and Toxicology, Manipal Academy of Higher Education, Mangalore, India; AGK Research Department, Nepal Health Research Council, Kathmandu, Nepal; AGL Research Department, Public Health Research Society Nepal, Kathmandu, Nepal; AGM The Royal Marsden NHS Foundation Trust, The Royal Marsden NHS Foundation Trust, London, England; AGN Faculty of Science and Engineering, School - Computing and Information Science, Anglia Ruskin University, London, England; AGO Centre for Research and Development, Chandigarh University, Punjab, India; AGP Division of Research and Development, Lovely Professional University, Phagwara, India; AGQ Division of Ophthalmology & Visual Sciences, University of Nottingham, Nottingham, England; AGR First Department of Ophthalmology, Aristotle University of Thessaloniki, Thessaloniki, Greece; AGS Department of Medicine and Surgery, University of Bologna, Bologna, Italy; AGT Medical University of Vienna, Vienna, Austria; AGU School of Health and Biomedical Sciences, Royal Melbourne Institute of Technology (RMIT) University, Melbourne, VIC, Australia; AGV University of Sydney, Sydney, NSW, Australia; AGW Vision and Eye Research Institute, Anglia Ruskin University, Cambridge, England; AGX Department of Sociology, Anthropology, and Public Health, University of Maryland Baltimore County, Baltimore, MD, USA; AGY Department of Preventive Medicine, Yonsei University, Seoul, South Korea; AGZ Institute of Health Services Research, Yonsei University, Seoul, South Korea; AHA Forensic Medicine and Toxicology, All India Institute of Medical Sciences, Hyderabad, India; AHB School of Nursing, University of British Columbia, Vancouver, BC, Canada; AHC Department of Medical Sciences, University of Torino, Torino, Italy; AHD Department of Imaging, AOU Città Della Salute E Della Scienza Di Torino (AOU City of Health and Science of Turin), Torino, Italy; AHE Department of Physiotherapy, Charotar University of Science and Technology, Anand, India; AHF Department of Cardiology, University of Kansas Medical Center, Kansas City, KS, USA; AHG Department of Cardiology, US Department of Veterans Affairs (VA), Kansas City, MO, USA; AHH Department of Research and Training, Population Council Institute, New Delhi, India; AHI College of Nursing, All India Institute of Medical Sciences, Deoghar, India; AHJ College of Dental Medicine, Roseman University of Health Sciences, South Jordan, UT, USA; AHK Department of Community Medicine, RAK Medical and Health Sciences University, Ras Al Khaimah, United Arab Emirates; AHL Second Propedeutic Department of Internal Medicine, Aristotle University of Thessaloniki, Thessaloniki, Greece; AHM Faculty of Humanities and Social Sciences, Tribhuvan University, Kathmandu, Nepal; AHN Department of Genetics, Yale University, New Haven, CT, USA; AHO Physiology Research Center, Iran University of Medical Sciences, Tehran, Iran; AHP Department of Physiology, Iran University of Medical Sciences, Tehran, Iran; AHQ Department of Community Medicine and Family Medicine, All India Institute of Medical Sciences, Hyderabad, India; AHR School of Population Health, University of New South Wales, Kensington, NSW, Australia; AHS College of Public Health, Medical, and Veterinary Sciences, James Cook University, Townsville, QLD, Australia; AHT Department of Public Health, Trnava University, Trnava, Slovakia; AHU Center for Research and Innovation, Ateneo De Manila University, Pasig City, Philippines; AHV Australian Institute of Health Innovation, Macquarie University, Sydney, NSW, Australia; AHW Fellowship, The G4 Alliance, Chicago, IL, USA; AHX Department of Food, Environmental and Nutritional Sciences, University of Milan, Milan, Italy; AHY School of Pharmacy, University of Nizwa, Nizwa, Oman; AHZ Department of Basic Medical Sciences, Durban University of Technology, Durban, South Africa; AIA Mental Health Research Institute, Tomsk National Research Medical Center, Tomsk, Russia; AIB Siberian State Medical University, Tomsk, Russia; AIC Department of Occupational Health Engineering, Sirjan School of Medical Sciences, Sirjan, Iran; AID Division of Infectious Disease, Mayo Clinic, Rochester, MN, USA; AIE Department of Occupational Health and Safety Engineering, Ardabil University of Medical Science, Ardabil, Iran; AIF Non-communicable Diseases Research Center, Bam University of Medical Sciences, Bam, Iran; AIG Centro De Investigaciones Clinicas (Clinical Research Center), Fundación Valle Del Lili (Valle Del Lili Foundation), Cali, Colombia; AIH Centro PROESA, Universidad ICESI, Cali, Colombia; AII Centre for Dental Education and Research, All India Institute of Medical Sciences, New Delhi, India; AIJ Institute of Microbiology, Government College University Faisalabad, Faisalabad, Pakistan; AIK Rory Meyers College of Nursing, New York University, New York, NY, USA; AIL College of Public Health, George Mason University, Fairfax, VA, USA; AIM Faculty of Nursing, Universitas Airlangga, Surabaya, Indonesia; AIN Research Center in Advancing Community Healthcare, Surabaya, Indonesia; AIO Research Center for Public Health and Nutrition, National Research and Innovation Agency, Jakarta, Indonesia; AIP Department of Medical Oncology, Cancer Institute (W.I.A), Chennai, India; AIQ Ingram School of Engineering, Texas State University, San Marcos, TX, USA; AIR Health Research Institute (HRI), National Institutes of Health, Islamabad, Pakistan; AIS Department of Mathematics, Jazan University, Jazan, Saudi Arabia; AIT Department of Epidemiology, National Institute of Mental Health and Neurosciences, Bengaluru, India; AIU Department of Environmental Health Engineering, Torbat Heydariyeh University of Medical Sciences, Torbat Heydariyeh, Iran; AIV Health Science Research Centre, Torbat Heydariyeh University of Medical Sciences, Torbat Heydariyeh, Iran; AIW Sina Trauma and Surgery Research Center, Tehran University of Medical Sciences, Tehran, Iran; AIX Department of Public Health, Universitas Airlangga (Airlangga University), Surabaya, Indonesia; AIY College of Health Sciences, Cihan University-Sulaimaniya, Sulaymaniyah, Iraq; AIZ Department of Biology, University of Sulaimani, Sulaymaniyah, Iraq; AJA College of Medicine and Health Sciences, National University of Science and Technology, Sohar, Oman; AJB Department of Population Science and Human Resource Development, University of Rajshahi, Rajshahi, Bangladesh; AJC Institute of Health and Wellbeing, Federation University Australia, Berwick, VIC, Australia; AJD Future Technology Research Center, National Yunlin University of Science and Technology, Yunlin, Taiwan; AJE Pharmaceutical Analysis, National Institute of Pharmaceutical Education and Research, Hajipur, India; AJF Dr. Rajendra Prasad Government Medical College, Tanda, Kangra, India; AJG Department of Cardiovascular Medicine, Cleveland Clinic, Cleveland, OH, USA; AJH Department of Paediatrics, University of Colombo, Colombo, Sri Lanka; AJI Department of Clinical Sciences, University of Sharjah, Sharjah, United Arab Emirates; AJJ Department of Cardiology, Mansoura University, Mansoura, Egypt; AJK Department of Population Health, King Saud Bin Abdulaziz University for Health Sciences, Jeddah, Saudi Arabia; AJL Department of Midwifery, Ministry of Health of the Republic of Indonesia, Palu, Indonesia; AJM Department of Radiology, Stanford University, Stanford, CA, USA; AJN National Institute for Research in Tuberculosis, Indian Council of Medical Research, Chennai, India; AJO All India Institute of Medical Sciences, Bilaspur, India; AJP Centre for Clinical Pharmacology, Military Medical Academy, Belgrade, Serbia; AJQ Division of Pediatric Surgery, Stanford University, Palo Alto, CA, USA; AJR Nitte (Deemed to Be University), AB Shetty Memorial Institute of Dental Sciences, Nitte University, Mangalore, India; AJS Department of Oral Pathology, Microbiology and Forensic Odontology, Sharavathi Dental College and Hospital, Shimogga, India; AJT Department of Family Medicine, Rajarata University of Sri Lanka, Anuradhapura, Sri Lanka; AJU Department of Public Health, Amrita Institute of Medical Sciences, Ernakulam, India; AJV Academic Public Health England, Public Health England, London, England; AJW Internal Medicine Department, Armed Forces Medical College, Pune, India; AJX Computer Science and Engineering Department, Saveetha Institute of Medical and Technical Sciences, Chennai, India; AJY Center of Excellence in Precision Medicine and Digital Health, Department of Physiology, Faculty of Dentistry, Chulalongkorn University, Bangkok, Thailand; AJZ Department for Epidemiology and Biostatistics, National Institute for Health Development, Tallinn, Estonia; AKA School of Environment, Tehran University, Tehran, Iran; AKB Department of Epidemiology and Biostatistics, Rafsanjan University of Medical Sciences, Rafsanjan, Iran; AKC Faculty of Allied Health Sciences, Santosh (Deemed to Be University), Delhi, India; AKD Department of Pharmacology and Toxicology, University of Antioquia, Medellin, Colombia; AKE Warwick Medical School, University of Warwick, Coventry, England; AKF Department of Clinical Research, University of Sao Paulo, Ribeirão Preto, Brazil; AKG Department of Community Medicine, Government Medical College, Chandigarh, India; AKH Department of Population Health, Hofstra University, Hempstead, NY, USA; AKI Miyan Research Institute, International University of Business Agriculture and Technology, Dhaka, Bangladesh; AKJ Department of Analytical and Applied Economics, Utkal University, Bhubaneswar, India; AKK RUSA Centre of Excellence in Public Policy and Governance, Utkal University, Bhubaneswar, India; AKL Faculty of Medicine, Quest International University Perak, Ipoh, Malaysia; AKM Communicable Disease Surveillance Centre, Public Health Wales, Cardiff, Wales; AKN Department of Oral and Maxillofacial Surgery, Jagadguru Sri Shivarathreeswara University, Mysore, India; AKO Cardiovascular Department, Zagazig University, Zagazig, Egypt; AKP Tabriz University of Medical Sciences, Tabriz, Iran; AKQ Department of Pharmaceutical Chemistry, International Medical University, Gdańsk, Poland; AKR Department of Biology, University of Hail, Hail, Saudi Arabia; AKS Széchenyi István University, Széchenyi István University, Győr, Hungary; AKT Irfan Suat Gunsel Operational Research Institute, Near East University, Turkey, Nicosia, Türkiye; AKU Research Institute for Health Sciences and Environment, Shahid Beheshti University of Medical Sciences, Tehran, Iran; AKV Department of Health in Disaster & Emergency, Shahid Beheshti University of Medical Sciences, Tehran, Iran; AKW Faculty of Medicine, MAHSA University, Selangor, Malaysia; AKX Research Center for Immunodeficiencies, Tehran University of Medical Sciences, Tehran, Iran; AKY Sharjah Institute of Medical Sciences, University of Sharjah, Sharjah, United Arab Emirates; AKZ Biotechnology Research Center, Mashhad University of Medical Sciences, Mashhad, Iran; ALA Centre for Research Impact and Outcome, Chitkara College of Pharmacy, Chitkara University, Rajpura, India; ALB Department of Radiation Oncology, All India Institute of Medical Sciences, New Delhi, India; ALC Community Medicine and Family Medicine, All India Institute of Medical Sciences, Bibinagar, Hyderabad, India; ALD Department of Psychiatry, Ministry of Health, Manama, Bahrain; ALE Department of Microbiology, Ahvaz Jundishapur University of Medical Sciences, Ahvaz, Iran; ALF LMU-Munich, Munich, Germany; ALG Institute for Employment Research, Nuremberg, Germany; ALH Cephas Health Research Initiative Inc, Ibadan, Nigeria; ALI Department of Oral and Maxillofacial Surgery, University College Hospital, Ibadan, Ibadan, Nigeria; ALJ Department of Human Nutrition & Dietetics, Health Services Academy, Islamabad, Pakistan; ALK Surgical Department, North Colombo Teaching Hospital, Ragama, Sri Lanka; ALL Institute of Epidemiology and Preventive Medicine, National Taiwan University, Taipei, Taiwan; ALM Benang Merah Research Center, Benang Merah Research Center (BMRC), Minahasa Utara, Indonesia; ALN Department of Anatomy, Ras Al Khaimah Medical and Health Sciences University, Ras Al Khaimah, United Arab Emirates; ALO Entomology VectorLink, Njala University, Freetown, Sierra Leone; ALP Department of Entomology, Ain Shams University, Cairo, Egypt; ALQ Medical Ain Shams Research Institute (MASRI), Ain Shams University, Cairo, Egypt; ALR Department of Pediatrics, SRM Medical College Hospital And Research Centre, Kattankulathur, India; ALS School of Public Health and Health Management, University of Belgrade, Belgrade, Serbia; ALT Department of Pharmacology, All India Institute of Medical Sciences, Jodhpur, India; ALU Indira Gandhi Medical College and Research Institute, Puducherry, India; ALV Department of Public Health, Jahrom University of Medical Sciences, Jahrom, Iran; ALW Health Policy Research Center, Shiraz University of Medical Sciences, Shiraz, Iran; ALX Faculty of Science, Queensland University of Technology, Brisbane, QLD, Australia; ALY Health Sciences Research Center, Torbat Heydariyeh University of Medical Sciences, Torbat Heydariyeh, Iran; ALZ Department of Oral Pathology and Microbiology, Dr. D. Y. Patil Vidyapeeth, Pune (Deemed to Be University), Pune, India; AMA Department of Epidemiology, National Institute for Research in Tuberculosis, Chennai, India; AMB UGC Centre of Advanced Study in Psychology, Utkal University, Bhubaneswar, India; AMC Udyam-Global Association for Sustainable Development, Bhubaneswar, India; AMD Department of Food Processing and Nutrition, Karnataka State Akkamahadevi Women University, Vijayapura, India; AME Department of Preventive and Social Medicine, Jawaharlal Institute of Postgraduate Medical Education and Research, Puducherry, India; AMF Universidad ESAN, Universidad ESAN, Lima, Peru; AMG Graduate School of Business, University of the Fraser Valley, Abbotsford, BC, Canada; AMH Office of the Vice President for Research, University of Iowa, Iowa City, IA, USA; AMI General Medicine Department, Tehran University of Medical Sciences, Tehran, Iran; AMJ Faculty of Dentistry, University of Puthisastra, Phnom Penh, Cambodia; AMK Research Institute of Medical & Health Sciences, University of Sharjah, Sharjah, United Arab Emirates; AML Emergency Department, Manian Medical Centre, Erode, India; AMM Department of Psychology and Health Sciences Faculty of Human Sciences, Education, and Sports, Pegaso Telematic University, Naples, Italy; AMN Department of Medicine, Swami Vivekanand Subharti University, Meerut, India; AMO Charotar University of Science and Technology (CHARUSAT), Ashok and Rita Patel Institute of Physiotherapy, Changa, India; AMP Rita A. Patel Institute of Physiotherapy, The Charutar Vidya Mandal(CVM) University, Anand, India; AMQ School of Health Sciences, Universiti Sains Malaysia, Kota Bharu, Malaysia; AMR National Institute of Education, Nanyang Technological University, Singapore, Singapore; AMS Faculty of Medicine and Health Science, An-Najah National University, Nablus, Palestine; AMT Institute of Molecular Biology and Biotechnology (IMBB), The University of Lahore, Lahore, Pakistan; AMU Research Centre for Health Sciences (RCHS), The University of Lahore, Lahore, Pakistan; AMV Center for Medical and Bio-Allied Health Sciences Research, Ajman University, Ajman, United Arab Emirates; AMW Independent Consultant, Karachi, Pakistan; AMX Trauma Research Center, Shiraz University of Medical Sciences, shiraz, Iran; AMY School of Medicine, Alborz University of Medical Sciences, Karaj, Iran; AMZ Department of Science, Kazakh National Medical University, Almaty, Kazakhstan; ANA Lancaster University, Lancaster, England; ANB Columbia University, New York, NY, USA; ANC Department for Evidence-based Medicine and Evaluation, University for Continuing Education Krems, Krems, Austria; AND Universidad Espíritu Santo, Samborondón, Ecuador; ANE School of Science and Technology, The University of Georgia, Tbilisi, Georgia; ANF Department of Forensic Science, Shree Guru Gobind Singh Tricentenary University, Gurugram, India; ANG Department of Biotechnology, Graphic Era (Deemed to Be University), Dehradun, India; ANH Department of Physiotherapy, Galgotas University, Greater Noida, India; ANI Department of Social and Behavioral Health, University of Nevada Las Vegas, Las Vegas, NV, USA; ANJ Department of University Institute of Biotechnology, Chandigarh University, Mohali, India; ANK Michael J. Cousins Pain Management and Research Centre, Royal North Shore Hospital, Sydney, NSW, Australia; ANL Department of Forensic Science, Panjab University, Chandigarh, India; ANM Department of Nursing, Arba Minch University, Arba Minch, Ethiopia; ANN Kasturba Medical College, Manipal Academy of Higher Education, Manipal, India; ANO Department of Physiology, Pharmacology, and Toxicology, An-Najah National University, Nablus, Palestine; ANP School of Public Health, Haramaya University, Harar, Ethiopia; ANQ Department of Ophthalmology, Harvard University, Boston, MA, USA; ANR Ophthalmic Research Center (ORC), Shahid Beheshti University of Medical Sciences, Tehran, Iran; ANS School of Public Health, Tehran University of Medical Sciences, Tehran, Iran; ANT Forensic Medicine and Toxicology, All India Institute of Medical Sciences, Deoghar, India; ANU Department of Health in Disasters and Emergencies, Shahrekord University of Medical Sciences, Shahrekord, Iran; ANV Psychology Department, University of Massachusetts Lowell, Boston, MA, USA; ANW Department of Biology, Morgan State University, Baltimore, MD, USA; ANX Department of Environmental Health Sciences, Tulane University, New Orleans, LA, USA; ANY Alva's Institute of Medical Sciences & Research Centre, Rajiv Gandhi University of Health Sciences, Moodubidire, India; ANZ KS Hegde Medical Academy, Nitte University, Mangalore, India; AOA Manipal College of Dental Sciences, Mangalore, Manipal Academy of Higher Education, Manipal, India; AOB Department of Veterinary Public Health and Preventive Medicine, Usmanu Danfodiyo University, Sokoto, Sokoto, Nigeria; AOC Department of Neurosurgery, Columbia University Medical Center, New York, NY, USA; AOD Center for Technology and Innovation in Cardiovascular Informatics, Iran University of Medical Sciences, Tehran, Iran; AOE Department of Medical-Surgical Nursing, Mazandaran University of Medical Sciences, Sari, Iran; AOF Department of Nursing and Health Sciences, Flinders University, Adelaide, SA, Australia; AOG Kenneth H. Cooper Institute, Texas Tech University Health Sciences Center, Dallas, TX, USA; AOH TMSS Nursing College, Bogura, TMSS Grand Health Sector, Bogura, Bangladesh, Bogura, Bangladesh; AOI MS Foundation Healthcare & Physiotherapy, MS Foundation Healthcare & Physiotherapy, Sherpur, Bangladesh; AOJ Faculty of Dentistry, Federal University of Minas Gerais, Belo Horizonte, Brazil; AOK Research and Innovation, University of Chester, Chester, UK; AOL Amity Institute of Public Health and Hospital Administration, Amity University, Noida, India; AOM Department of Pharmacology, Government Medical College and Hospital, Chandigarh, India; AON Department of Radiodiagnosis, All India Institute of Medical Sciences, Bathinda, India; AOO Department of Community Medicine, Veer Chandra Singh Garhwali Government Institute of Medical Science and Research, Srinagar Garhwal, India; AOP Global and European Health Education and Study Institute, University of Georgia, Tbilisi, Georgia; AOQ National Center for Disease Control and Public Health, Tbilisi, Georgia; AOR Books Committee, Royal College of Psychiatrists, London, UK; AOS Department of Surgery, “Sf. Pantelimon” Emergency Clinical Hospital Bucharest, Bucharest, Romania; AOT Department of Development Studies, Daffodil International University, Dhaka, Bangladesh; AOU Faculty of Public Health, Universitas Ahmad Dahlan, Yogyakarta, Indonesia; AOV Department of Medicine, Washington University in St. Louis, Saint Louis, MO, USA; AOW Department of Health, A.C.S. Medical College and Hospital, Hyderabad, India; AOX Department of Clinical Research, National Institute for Research in Tuberculosis, Chennai, India; AOY Department of Public Health, Kandahar University, Kandahar, Afghanistan; AOZ Department of Medicine, Democritus University of Thrace, Alexandroupolis, Greece; APA Division of Preventive Medicine, University of Alberta, Edmonton, AB, Canada; APB School of Public Health, University of Alberta, Edmonton, AB, Canada; APC McGill University, Montreal, QC, Canada; APD Department of Orthopaedics, Massachusetts General Hospital, Boston, MA, USA; APE Department of Orthopaedics, Harvard University, Cambridge, MA, USA; APF Department of Medical Sciences, Sunway University, Subang Jaya, Malaysia; APG Department of Nursing, Muhammadiyah University of Surakarta, Surakarta, Indonesia; APH Department of Medicine, MyungSung Medical College, Addis Ababa, Ethiopia; API Praboromarajchanok Institute, Ministry of Public Health, Nonthaburi, Thailand; APJ Department of Life and Health Sciences, University of Nicosia, Nicosia, Cyprus; APK Data Science Institute, University of Technology Sydney, Sydney, NSW, Australia; APL Heart Research Institute, University of Sydney, Sydney, QLD, Australia; APM Gandhi Medical College, Kaloji Narayana Rao University of Health Sciences (KNRUHS), Secunderabad, India; APN Institute of Neuroscience and Physiology, University of Gothenburg, Gothenburg, Sweden; APO Department of Rehabilitation Medicine, Sabzevar University of Medical Sciences, Gothenburg, Sweden; APP Institute of Medical Sciences, The John Paul II Catholic University of Lublin, Lublin, Poland; APQ Department of Pharmacology, All India Institute of Medical Sciences, Deoghar, India; APR Department of Medicine, University of Valencia, Valencia, Spain; APS Carlos III Health Institute, Biomedical Research Networking Center for Mental Health Network (CiberSAM), Madrid, Spain; APT Department of Medical Informatics, Mashhad University of Medical Sciences, Mashhad, Iran; APU Applied Biomedical Research Center, Mashhad University of Medical Sciences, Mashhad, Iran; APV Department of Health, Safety, and Environmental Management, Abadan University of Medical Sciences, Abadan, Iran; APW Department of Biostatistics and Epidemiology, Shahid Sadoughi University of Medical Science, Yazd, Iran; APX Department of Pathology, Alexandria University, Alexandria, Egypt; APY Department of Dermatology, Carol Davila University of Medicine and Pharmacy, Bucharest, Romania; APZ Department of Dermato-Venereology, Dr. Victor Babes Clinical Hospital of Infectious Diseases and Tropical Diseases, Bucharest, Romania; AQA Department of Public Health, Debre Markos University, Debre Markos, Ethiopia; AQB Department of Public Health and Informatics, Bangladesh Medical University, Dhaka, Bangladesh; AQC Department of Public Health, Trakya University, Faculty of Medicine, Türkiye, Edirne, Türkiye; AQD Department of Midwifery, Arba Minch University, Arba Minch, Ethiopia; AQE Pediatric Intensive Care Unit, King Saud University, Riyadh, Saudi Arabia; AQF College of Medicine, Alfaisal University, Riyadh, Saudi Arabia; AQG College of Pharmacy, Alfaisal University, Riyadh, Saudi Arabia; AQH Department of Midwifery, Bahir Dar University, Bahir Dar, Ethiopia; AQI Department of Psychology, University of Ghana, Legon, Ghana; AQJ Department of Industrial Psychology, Stellenbosch University, Cape Town, South Africa; AQK Research Department, Policy Research Institute, Kathmandu, Nepal; AQL Department of Economics, The American University in Cairo, Cairo, Egypt; AQM Department of Paediatric and Neonatal Sciences, Saveetha University, Chennai, India; AQN School of Public Health, Sri Ramaswamy Memorial Institute of Science and Technology, Chennai, India; AQO Forensic Medicine & Toxicology, All India Institute of Medical Sciences, Nagpur, India; AQP Faculty of Public Health, Universitas Sam Ratulangi (Sam Ratulangi University), Manado, Indonesia; AQQ Department of Prosthetic Dentistry, Kazakh National Medical University, Almaty, Kazakhstan; AQR College of Nursing, Kaohsiung Medical University, Taiwan, Kaohsiung, Taiwan; AQS Global Surgery Department, Data Science Center for the Study of Surgery, Injury and Equity in Africa, Buea, Cameroon; AQT Department of Internal Medicine, University of Medicine and Pharmacy at Ho Chi Minh City, Ho Chi Minh City, Viet Nam; AQU Department of Business Analytics, University of Massachusetts Dartmouth, Dartmouth, MA, USA; AQV Research and Advocacy Initiative, ALS Vietnam, Quang Ngai, Viet Nam; AQW Department of Clinical and Experimental Medicine, University of Pisa, Pisa, Italy; AQX Harvard T.H. Chan School of Public Health, Harvard University, Boston, MA, USA; AQY Infectious Disease Epidemiology Research Group, Kwame Nkrumah University of Science and Technology, Kumasi, Ghana; AQZ Department of Internal Medicine, Wake Forest University, Winston-Salem, NC, USA; ARA Department of Public Health, Wachemo University, Hossana, Ethiopia; ARB Department of Epidemiology and Biostatistics, Haramaya University, Harar, Ethiopia; ARC University Hospitals Cleveland Medical Center, Cleveland, OH, USA; ARD International Center for Chemical and Biological Sciences, University of Karachi, Karachi, Pakistan; ARE Department of Biology and Biochemistry, University of Houston, Houston, TX, USA; ARF Independent Consultant, St. Augustine, Trinidad and Tobago; ARG North Central Regional Health Authority, Champ Fleurs, Trinidad and Tobago; ARH Department of Physiotherapy, Federal Ministry of Health, Gusau, Nigeria; ARI Institute of Health and Wellbeing, Federation University Australia, Churchill, VIC, Australia; ARJ Department of Neuroscience, Monash University, Clayton, VIC, Australia; ARK Department of Nursing, Wachemo University, Hossana, Ethiopia; ARL Amity Institute of Biotechnology, Amity University Rajasthan, Jaipur, India; ARM Symbiosis College of Nursing, Symbiosis International University, Pune, India; ARN Department of Physiotherapy, Bayero University, Kano, Nigeria; ARO Department of Rehabilitation Sciences, Hong Kong Polytechnic University, Hong Kong, China; ARP College of Nursing, All India Institute of Medical Sciences, Nagpur, India; ARQ College of Health and Sport Sciences, University of Bahrain, Zallaq, Bahrain; ARR Sociedad Argentina De Medicina, Buenos Aires, Argentina; ARS Hospital Vélez Sarsfield, Buenos Aires, Argentina; ART UKK Institute, Tampere, Finland; ARU Faculty of Medicine and Health Technology, Tampere University, Tampere, Finland; ARV Raffles Neuroscience Centre, Raffles Hospital, Singapore, Singapore; ARW Yong Loo Lin School of Medicine, National University of Singapore, Singapore, Singapore; ARX Siksha ‘O’ Anusandhan (Deemed to Be University), Bhubaneswar, India; ARY Department of Medical and Surgical Sciences and Advanced Technologies “GF Ingrassia”, University of Catania, Catania, Italy; ARZ Department of Surgery, University of Southampton, Southampton, England; ASA College of Medicine and Veterinary Medicine, University of Edinburgh, Edinburgh, Scotland; ASB Centro De Investigación En Nutrición Y Salud, National Institute of Public Health, Mexico City, Mexico; ASC Health Initiative of the Americas, University of California Berkeley, Berkeley, CA, USA; ASD Radcliffe Department of Medicine, University of Oxford, Oxford, England; ASE Occupational Medicine Unit, Sant'Orsola Malpighi Hospital, Bologna, Italy; ASF Department of Health Care Administration and Economics, National Research University Higher School of Economics, Moscow, Russia; ASG Egyetem Square 1, Széchenyi István University, Győr, Hungary; ASH Irfan Suat Gunsel Operational Research Institute, Near East University, Nicosia, Türkiye; ASI Department of Paediatrics, All India Institute of Medical Sciences, Bathinda, India; ASJ National Institute of Health Data Science, Peking University, Beijing, China; ASK School of Public Health, Xuzhou Medical University, Xuzhou, China; ASL School of Life Course and Population Sciences, King's College London, London, England; ASM Department of Family and Preventive Medicine, Faculty of Medicine, Prince of Songkla University, Songkhla, Thailand; ASN Department of Public Health Sciences, Rivers State University, Port Harcourt, Nigeria; ASO Department of Orthopaedics, General Hospital of Central Theater Command, Wuhan, China; ASP Fourth Military Medical University, Xi'an, China; ASQ Demographic Change and Aging Research Area, Federal Institute for Population Research, Wiesbaden, Germany; ASR Department of Physical Therapy, Naresuan University, Phitsanulok, Thailand; ASS Department of Surgery, University of Colombo, Colombo, Sri Lanka; AST Independent Investigator, Washington, DC, USA; ASU Research Organisation, Inter-Continental Omni-Research in Medicine Collaborative, Berlin, Germany; ASV Department of Intelligent Medical Engineering, Anhui Medical University, Anhui, China; ASW Department of Surgery, The First Affiliated Hospital of Anhui Medical University, Hefei, Anhui, China; ASX School of Public Health Sciences and Technology, Malla Reddy Vishwavidyapeeth, Hyderabad, Telangana, India; ASY Department of Environmental Health and Epidemiology, National Institute for Research in Environmental Health, Bhopal, India; ASZ Zilber College of Public Health, University of Wisconsin-Milwaukee, Milwaukee, WI, USA; ATA Public Health Department, Health Services Academy, Islamabad, Pakistan; ATB The George Institute for Global Health, Imperial College London, London, England; ATC National Center for Chronic and Noncommunicable Disease Control and Prevention, Chinese Center for Disease Control and Prevention, Beijing, China; ATD The George Institute for Global Health, University of New South Wales, Sydney, NSW, Australia; ATE Department of Public Health, Trakya University, Edirne, Türkiye; ATF Saw Swee Hock School of Public Health, National University of Singapore, Singapore, Singapore; ATG KHANA Center for Population Health Research, Phnom Penh, Cambodia; ATH Department of Health Management, Süleyman Demirel Üniversitesi (Süleyman Demirel University), Isparta, Türkiye; ATI Community Medicine Department, Jawaharlal Nehru Medical College, KLE Academy of Higher Education and Research, Belagavi, India; ATJ Department of Pediatrics, Kyung Hee University, Seoul, South Korea; ATK Department of Biostatistics, University of Toyama, Toyama, Japan; ATL Department of Public Health, Juntendo University, Tokyo, Japan; ATM Department of Health Policy and Management, Jackson State University, Jackson, MS, USA; ATN School of Business & Economics, Tsinghua University, Beijing, China; ATO School of Public Health, Hubei University of Medicine, Shiyan, China; ATP Association for Socially Applicable Research (ASAR), Pune, India; ATQ Department of Emergency Medicine, Global Emergency Medicine Innovation and Implementation (GEMINI) Research Center, Durham, NC, USA; ATR Department of Family and Community Medicine, University of Hail, Hail, Saudi Arabia; ATS Department of Environmental and Occupational Health, Universiti Putra Malaysia, Serdang, Malaysia; ATT Department of Oncology and Mammology, Kazakh Russian Medical University, Almaty, Kazakhstan; ATU Research and Development Department, Sina Medical Biochemistry Technologies, Shiraz, Iran; ATV Department of Bioengineering and Therapeutical Sciences, University of California San Francisco, San Francisco, CA, USA; ATW Department of Administration, PGxAI, San Francisco, CA, USA; ATX Mohammed VI International School of Public Health, Mohammed VI University of Sciences and Health (UM6SS), Casablanca, Morocco; ATY Institute for Healthcare System Analysis (IA2S), Paris, France; ATZ Department of Zoology and Entomology, Al-Azhar University, Cairo, Egypt; AUA School of Public Health, Huazhong University of Science and Technology, Wuhan, China; AUB School of Public Health, Wuhan University of Science and Technology, Wuhan, China; AUC Hubei Province Key Laboratory of Occupational Hazard Identification and Control, Wuhan University of Science and Technology, Wuhan, China; AUD School of Public Health, Wuhan University, Wuhan, China; AUE Jockey Club School of Public Health and Primary Care, The Chinese University of Hong Kong, Hong Kong, China; AUF Department of Surgery, Zhejiang University, Zhejiang Province, China; AUG Atchabarov Scientific Research Institute of Fundamental and Applied Medicine, Kazakh National Medical University, Almaty, Kazakhstan; AUH Department of Anatomy, Addis Ababa University, Addis Ababa, Ethiopia; AUI Clinical Research Centre, An-Najah National University Hospital, Nablus, Palestine; AUJ Department of Building Engineering and Environment, Palestine Technical University (Kadoorie), Tulkarem, Palestine; AUK Civil Engineering and Sustainable Structures, Palestine Technical University (Kadoorie), Tulkarem, Palestine

## Abstract

**Background:**

Road injuries are a leading cause of mortality and morbidity worldwide. Years of international efforts have aimed to strengthen policy engagement, including the 2020 UN General Assembly's proclamation of the Second Decade of Action for Road Safety (2021–30), targeting a 50% reduction in road traffic deaths and serious injuries by 2030. The aim of this study is to provide estimates to monitor progress and identify intervention gaps.

**Methods:**

As part of the Global Burden of Diseases, Injuries, and Risk Factors Study 2023, we estimated incidence, mortality, and morbidity of road injuries for 204 countries and territories from 1990 to 2023. Four road injury types and 47 nature-of-injury categories were examined. Morbidity and mortality data from clinical records, vital registration, and police reports were harmonised using meta-analytic techniques to ensure consistency and correct for systematic bias. Incidence was modelled with the meta-regression tool Disease Modelling-Meta-Regression version 2.1 and cause-specific mortality with the Cause of Death Ensemble model, both incorporating location-specific covariates to support interpolation. Years of life lived with disability (YLDs) were estimated from the prevalence and severity of the nature of road injury, and years of life lost (YLLs) from the number of cause-specific deaths multiplied by the standard life expectancy at the age of death. Disability-adjusted life-years (DALYs) were the sum of YLLs and YLDs. All metrics were calculated with 95% uncertainty intervals (UIs).

**Findings:**

In 2023, there were 50·9 million (95% UI 46·1–56·1) road injury incident cases, 1·34 million (1·04–1·58) deaths, and 75·3 million (59·8–89·2) DALYs globally. Road injuries were the leading global cause of death among males aged 10–39 years. Between 1990 and 2023, age-standardised incidence decreased by 38·3% (95% UI 36·9–39·7) and mortality decreased by 32·3% (6·1–49·0), but progress varied widely by World Bank income group. Mortality in low-income countries (43·8 [95% UI 31·7–56·0] deaths per 100 000 population) was approximately six times higher than in high-income countries (7·5 [7·1–7·9] deaths per 100 000), despite the high-income countries showing the highest age-standardised incidence rates (858·1 [95% UI 781·9–947·1] cases per 100 000). In the past decade, many countries achieved notable reductions in road injuries, but others, including Ghana and the USA, saw increases. More severe injuries tended to occur in low-income and middle-income countries.

**Interpretation:**

Although global incidence, mortality, and DALY rates from road injuries have declined, progress remains uneven, with pronounced disparities across income groups reflecting systemic inadequacies in infrastructure, vehicle standards, enforcement, and post-crash care. Strengthening emergency response, improving road design, enforcing safety measures, and adapting policies to the evolving demographics remain essential.

**Funding:**

Gates Foundation.

## Introduction

Road injuries are a long-standing public health issue.^1^, ^2^ In the past two decades, numerous global initiatives have sought to bring road injuries into public health focus.^3^ WHO released its first global strategy for road injury prevention in 2001,^4^ followed by the UN General Assembly's inaugural road safety resolution in 2003^5^ and the creation of the UN Road Safety Collaboration in 2004. Dedicated sources of financing were established, including the Global Road Safety Facility fund in 2006^6^ and the UN Road Safety Fund in 2018. Private philanthropy has complemented these multilateral efforts, such as Bloomberg Philanthropies, who have invested more than US$865 million in road safety since 2007.^7^ Following the UN's Decade of Action for Road Safety (2011–20)^8^ and guided by the Stockholm Declaration,^9^ the Second Decade of Action commenced in 2021 with an ambitious target of halving road traffic deaths and injuries by 2030.^10^ Despite these global initiatives, investment and policy implementation lag behind what the target requires.^2^, ^11^

A key barrier to progress is the persistent paucity of reliable data.^12^ To raise awareness, monitor progress, and inform policy development, this study presents the most recent findings on road injuries from the Global Burden of Diseases, Injuries, and Risk Factors Study (GBD) 2023, a comprehensive epidemiological study that produces internally consistent and comparable estimates for 375 diseases and injuries and 88 modifiable risk factors for 204 countries and territories.^13^, ^14^ We examine estimates of incidence, mortality, years of life lost (YLLs), years of life lived with disability (YLDs), and disability-adjusted life-years (DALYs) from 1990 to 2023 by country, by 21 GBD regions, and by four World Bank income groups.^13^, ^14^ Estimates are disaggregated by sex, age, nature of injury, and five specific road user types: motor vehicle occupants, motorcyclists, cyclists, pedestrians, and other road users. This report was produced as part of the GBD Collaborator Network and in accordance with the GBD Protocol.

**Research in context**

**Evidence before this study**

The Global Burden of Diseases, Injuries, and Risk Factors Study (GBD) 2017 was the last comprehensive and globally comparable assessment of global morbidity and mortality from road injuries. To identify subsequent evidence, we restricted our search to studies published after 2017. We conducted a review of English-language articles in PubMed from Jan 1, 2017, to Dec 1, 2025, using search terms on road injuries and burden estimates (appendix 1 p 3). We included studies reporting road injury incidence, mortality, morbidity, or disability-adjusted life-years (DALYs), and excluded articles focused solely on clinical management, rehabilitation, or non-road-related injuries. We also reviewed key global sources, including WHO road safety reports and World Bank reports. The WHO Global Status Report on Road Safety (GSRRS), tracking road safety progress across more than 190 countries, estimated 1·19 million road injury deaths in 2021 in its most recent 2023 edition. The report also highlighted that road injury deaths remain the leading cause of mortality in children and young people aged 5–29 years, with 90% of all traffic deaths occurring in low-income and middle-income countries. However, the GSRRS provides only mortality estimates, without quantifying non-fatal outcomes such as years lived with disability (YLDs) or DALYs, and its most recent data extend only to 2021. Other published literature was limited to specific countries or regions, focused on particular age groups or road users, or offered only cross-sectional snapshots rather than long-term, standardised trend estimates.

**Added value of this study**

This study addresses key gaps in the existing evidence by providing the most up-to-date and comparable estimates of road injury incidence, mortality, and disability using data up to 2023 on the global, regional, and national levels. Using data from a broad range of sources, including hospital and emergency department records, vital registration and verbal autopsy data, censuses, surveys, and police records, we generated internally consistent estimates of road injury mortality, incidence, and disability by age, sex, location, and calendar year. The estimation framework accounts for under-reporting and misclassification and propagates uncertainty across all inputs. Unlike previous global assessments, this study systematically disaggregates road injuries by detailed road user categories resulting from nature-of-injury profiles, enabling comprehensive quantification of both fatal and non-fatal health loss. By generating burden estimates (years of life lost, YLDs, and DALYs) within a unified framework, this work enables direct comparison of road injury burden across populations, over time, and relative to other causes of health loss.

**Implications of all the available evidence**

Road injuries remain a major, preventable cause of premature death and disability. While international commitments, including the UN's Sustainable Development Goals and Second Decade of Action for Road Safety, signal ambition and seek to reinvigorate political will, meaningful progress demands concrete steps that galvanise cross-sector cooperation and secure sustained investment to support continuous, operational efforts.

## Methods

### Case definitions

Road injuries in GBD are defined as death or disability due to an unintentional interaction with an automobile, motorcycle, pedal cycle, or other vehicle. In the GBD four-level cause hierarchy, which orders causes from broad groupings to increasingly specific ones, road injuries are one of 176 Level 3 causes (other examples include falls and interpersonal violence) and are further disaggregated into five mutually exclusive Level 4 causes by road user types: motor vehicle road injuries, motorcyclist road injuries, bicycle road injuries, pedestrian road injuries, and other road injuries (eg, animal riders, occupants of animal-drawn vehicles, and tram occupants).^13^, ^14^ Intentional road injuries (eg, self-harm or assault) or other transport injuries (eg, aeroplane, train, and boat injuries) are excluded.

The nature of injury, referring to the specific bodily harm sustained, was also examined. GBD includes 47 nature-of-injury categories, grouped into seven broader classes: amputations, burns, fractures, head injuries, spinal injuries, minor injuries, and other injuries. Each cause of injury is mapped to a single nature-of-injury category; when multiple injuries are recorded for the same case, the category associated with the highest burden is selected.^13^

### Data sources

Road injury morbidity was estimated using data from hospital inpatient records, emergency departments, outpatient encounters, and population-representative surveys.^13^ To exclude clinically insignificant injuries (eg, scrapes), cases were restricted to injuries that required some form of health care in a system with full health-care access.^13^ Clinical data sources were mapped to road injury causes using ICD-9 and ICD-10 codes (appendix 1 p 4). Injuries that would have required care but were not treated due to financial or geographical barriers were accounted for by survey data.^13^ Road injury mortality was estimated using data from vital registrations, verbal autopsies, mortality surveillance, censuses, surveys, and police records.^14^

### Road injury incidence

Road injury incidence was estimated using Disease Modelling-Meta-Regression version 2.1 (DisMod-MR 2.1),^15^ a Bayesian meta-regression tool to synthesise epidemiological data within a compartmental disease model while ensuring internal consistency across incidence, prevalence, remission, and mortality.^13^ Input data were adjusted using standard GBD methods, to account for underascertainment, variation in care-seeking, and systematic differences in case definitions across data sources.^13^, ^16^ Country-level covariates included in the DisMod-MR 2.1 models were the age-standardised summary exposure value and vehicles per capita. Incidence estimated with DisMod-MR 2.1 corresponded to road injuries requiring inpatient care. The number of outpatient cases was derived by estimating the ratio of inpatient to outpatient cases using meta-regression—Bayesian, regularised, trimmed. Further methodological details are given in the GBD 2023 capstone appendices.^13^

### Road injury mortality

Cause-specific mortality from road injuries was estimated using the Cause of Death Ensemble model (CODEm), an ensemble framework that evaluates multiple model classes and covariate sets and combines the best-performing models into a weighted ensemble.^14^, ^17^ All cause-of-death input data underwent standard GBD processing, including the redistribution of ill-defined causes of death. In settings with sparse or no direct mortality data, CODEm generates estimates by drawing on covariates and the hierarchical mixed-effects structure of its component models, allowing information to be borrowed across countries and regions.^17^ Location-level covariates varied by road injury type and included, among others, the Socio-demographic Index,^18^ the Healthcare Access and Quality Index,^19^ and the proportion of two-wheeled vehicles. Further details are available in the GBD capstone appendices.^14^, ^20^

### Calculation of YLDs, YLLs, and DALYs

YLDs were estimated by multiplying the prevalence of each nature-of-injury sequela by its corresponding disability weight.^13^ Because injury-related disability is determined by the nature of bodily harm rather than the external cause alone, incidence estimates were first mapped to nature-of-injury categories, informed by cause–nature distributions derived from clinical data sources that report both the cause and nature of injury.^13^ Prevalence by nature of injury was then derived by multiplying these incidence estimates by nature-specific and severity-specific duration estimates for short-term sequelae, with an estimated proportion of cases progressing to long-term (permanent) health loss. Disability weights, ranging from 0 (full health) to 1 (equivalent to death), were assigned according to the injury nature and severity, with separate weights for short-term and long-term sequelae, and, where relevant, for treated and untreated cases.^13^

YLLs were calculated by multiplying the number of cause-specific deaths in each age-sex-location-year stratum by the standard life expectancy at the age of death.^14^, ^18^ DALYs were calculated as the sum of YLDs and YLLs. Further details are available in previous publications.^13^, ^14^, ^20^

For all estimates, the mean was calculated across 250 draws from the posterior distribution, and 95% uncertainty intervals (UIs) were derived from the 0·025 and 0·975 percentile values across these draws.^13^, ^14^ Age-standardised rates were computed using the GBD standard population to enable comparisons across settings with different demographic structures.^18^ Data visualisations were done with Python (version 3.11.9). This study adheres to GATHER (appendix 1 p 5).

### Role of the funding source

The funders of the study had no role in study design, data collection, data analysis, data interpretation, or the writing of the report.

## Results

In 2023, there were an estimated 50·9 million (95% UI 46·1–56·1) road injury incident cases globally, corresponding to an age-standardised incidence rate of 623·0 (95% UI 566·9–686·8) cases per 100 000 population (table 1; appendix 1 pp 7–9). In the World Bank income groups, the lowest incidence rate was observed in the lower-middle-income group (446·4 [405·3–491·1] cases per 100 000), while the highest age-standardised incidence rate was seen in the high-income group (858·1 [781·9–947·1] cases per 100 000; table 1; appendix 1 pp 7–9). Between 1990 and 2023, the global age-standardised road injury incidence declined by 38·3% (95% UI 36·9–39·7), with reductions observed across all income groups, ranging from decreases of 14·7% (12·8–16·6) in the lower-middle-income group to 49·0% (47·8–50·1) in the high-income group (appendix 1 pp 7–9, 34). Decreases in incidence were estimated in 179 of 204 countries and territories, while ten had increases in incidence (appendix 1 pp 10–12, 35).

**Table 1:** Age-standardised road injury incidence and mortality rates in 2023, and their percentage change from 1990 to 2023 globally, by World Bank income groups, and by road user type

	**Incidence**		**Mortality**	
	Age-standardised incidence rate (per 100 000), 2023	Percentage change in age-standardised incidence rate, 1990–2023	Age-standardised mortality rate (per 100 000), 2023	Percentage change in age-standardised mortality rate, 1990–2023
**Road injuries**				
Global	623·0 (566·9–686·8)	−38·3% (−39·7 to −36·9)	16·1 (12·5–19·0)	−32·3% (−49·0 to −6·1)
Low income	558·7 (525·8–595·3)	−25·6% (−27·4 to −23·9)	43·8 (31·7–56·0)	16·2% (−21·9 to 85·7)
Lower-middle income	446·4 (405·3–491·1)	−14·7% (−16·6 to −12·8)	19·1 (13·2–24·3)	−0·7% (−29·2 to 44·1)
Upper-middle income	751·3 (672·6–836·0)	−29·1% (−31·7 to −26·8)	13·2 (10·9–15·3)	−54·5% (−66·6 to −26·0)
High income	858·1 (781·9–947·1)	−49·0% (−50·1 to −47·8)	7·5 (7·1–7·9)	−61·1% (−62·7 to −58·5)
**Motor vehicles**				
Global	244·1 (216·1–279·0)	−42·3% (−44·0 to −41·0)	6·3 (5·0–7·9)	−26·4% (−48·9 to 7·2)
Low income	271·5 (252·0–294·8)	−30·2% (−32·5 to −28·1)	19·6 (13·3–27·3)	7·6% (−30·5 to 72·5)
Lower-middle income	171·9 (153·3–194·6)	−22·5% (−24·6 to −20·2)	6·2 (4·5–8·5)	−15·9% (−43·4 to 33·6)
Upper-middle income	234·4 (202·7–272·9)	−31·5% (−34·9 to −28·5)	4·8 (3·8–5·8)	−37·0% (−66·1 to 3·0)
High income	453·8 (400·0–521·7)	−48·3% (−49·6 to −46·9)	4·5 (4·2–4·7)	−54·9% (−58·1 to −51·5)
**Motorcyclists**				
Global	81·6 (69·3–96·2)	−16·0% (−18·6 to −12·5)	3·8 (2·6–5·0)	38·2% (−27·4 to 146·6)
Low income	39·0 (33·5–44·7)	−4·6% (−7·4 to −1·5)	4·5 (2·7–6·8)	106·5% (−10·0 to 381·5)
Lower-middle income	71·1 (60·8–83·7)	13·3% (10·4 to 17·4)	5·4 (3·0–7·5)	81·7% (−8·1 to 242·4)
Upper-middle income	107·0 (90·5–126·1)	−6·0% (−10·4 to −1·1)	3·6 (2·8–4·4)	9·6% (−46·8 to 107·6)
High income	80·7 (67·3–97·2)	−38·5% (−41·0 to −35·9)	1·1 (1·0–1·2)	−44·1% (−52·4 to −36·4)
**Cyclists**				
Global	122·8 (103·0–149·3)	−30·9% (−33·3 to −28·6)	1·0 (0·7–1·5)	8·4% (−50·1 to 98·3)
Low income	64·0 (54·3–77·1)	−17·9% (−20·7 to −15·1)	1·9 (1·0–3·6)	64·5% (−36·5 to 331·5)
Lower-middle income	78·9 (65·4–96·1)	−3·6% (−7·3 to 0·4)	1·2 (0·6–2·0)	54·1% (−36·1 to 233·0)
Upper-middle income	161·0 (134·6–195·1)	−15·7% (−19·9 to −11·5)	1·1 (0·7–1·5)	−6·2% (−68·5 to 105·8)
High income	183·8 (149·9–225·4)	−43·0% (−45·0 to −40·8)	0·3 (0·3–0·3)	−56·0% (−61·1 to −50·4)
**Pedestrians**				
Global	137·3 (119·1–159·1)	−45·0% (−46·9 to −43·0)	4·8 (3·5–6·4)	−57·7% (−71·9 to −35·0)
Low income	148·1 (133·5–162·1)	−23·8% (−26·0 to −21·7)	17·2 (11·1–24·8)	9·1% (−35·8 to 104·9)
Lower-middle income	96·0 (84·0–110·0)	−19·2% (−22·0 to −16·6)	6·1 (3·9–8·7)	−23·3% (−55·2 to 33·7)
Upper-middle income	198·2 (168·6–232·0)	−39·5% (−42·4 to −36·9)	3·5 (2·8–4·5)	−78·8% (−85·6 to −61·0)
High income	107·8 (91·1–125·0)	−63·5% (−65·1 to −62·0)	1·6 (1·4–1·7)	−76·0% (−78·6 to −72·7)
**Others**				
Global	37·1 (28·2–46·1)	−39·1% (−42·4 to −35·4)	0·2 (0·1–0·3)	−17·7% (−65·1 to 69·8)
Low income	36·1 (27·1–44·3)	−25·7% (−29·3 to −21·7)	0·5 (0·2–0·7)	73·2% (−37·3 to 432·0)
Lower-middle income	28·5 (21·6–35·7)	−24·9% (−28·7 to −21·5)	0·2 (0·1–0·4)	18·5% (−58·7 to 239·5)
Upper-middle income	50·8 (38·9–62·8)	−40·6% (−44·5 to −35·6)	0·2 (0·1–0·2)	−45·4% (−76·5 to 8·7)
High income	32·1 (23·2–41·6)	−42·1% (−45·2 to −38·8)	0·0 (0·0–0·1)	−63·8% (−70·8 to −56·5)

Data in parentheses are 95% uncertainty intervals. Negative percentage values indicate declines in age-standardised rates. Uncertainty intervals crossing zero indicate statistical uncertainty about the direction of change.

Of Level 4 causes of road injuries, motor vehicle injuries accounted for the largest share of road injury cases globally (19·8 million [95% UI 17·5–22·6] cases, 38·8% of the global total) in 2023, followed by pedestrian (11·6 million [10·0–13·4] cases, 22·7%), cyclist (10·1 million [8·40–12·1] cases, 19·8%), and motorcyclist (6·70 million [5·71–7·91] cases, 13·2%) injuries (appendix 1 pp 13, 36). In the high-income group, cyclist injuries accounted for 20·9% (2·38 million [1·99–2·80] cases) of all road injuries, making them the second-largest Level 4 cause. At the regional level, cyclist road injuries surpassed motor vehicle injuries to constitute the largest contributor to road injuries in high-income Asia Pacific, while in tropical Latin America, motorcyclist injuries exceeded those from motor vehicles (appendix 1 p 36). Further regional and income-group patterns are in appendix 1 (p 36). From 1990 to 2023, age-standardised incidence rates decreased for all Level 4 road injury types globally (table 1; appendix 1 p 37). Consistent decreases were observed across income groups, except for motorcyclist road injuries in the lower-middle-income group (table 1; appendix 1 p 37). At the regional level, motorcyclist injuries showed marked heterogeneity, with decreases over this period in 11 regions but increases in eight regions, particularly across Latin American regions (appendix 1 p 37).

**Figure 1:**
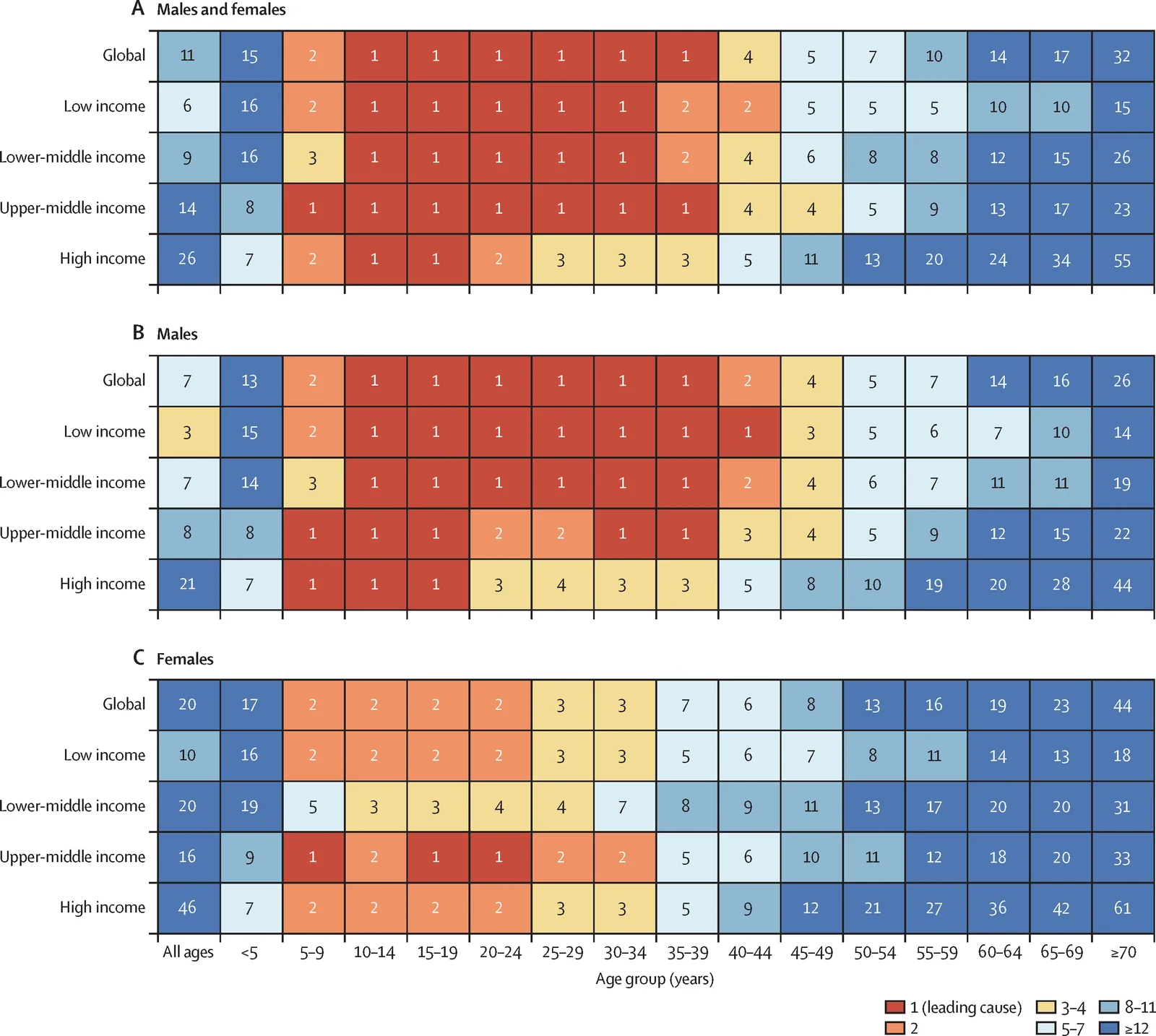
Ranking of road injury deaths relative to all Level 3 causes of death, 2023 (A) Data for males and females combined. (B) Ranking among males. (C) Ranking among females. The ranking is based on counts and is shown across age groups.

There were an estimated 1·34 million (95% UI 1·04–1·58) deaths due to road injuries in 2023, corresponding to an age-standardised mortality rate of 16·1 (95% UI 12·5–19·0) deaths per 100 000 population (table 1; appendix 1 pp 14–16). Road injuries ranked 11th of all Level 3 causes of mortality globally (figure 1). More than 90% of global road injury deaths occurred in the low-income and middle-income groups (appendix 1 p 38). The high-income group had the lowest age-standardised mortality rate, estimated at 7·5 (7·1–7·9) deaths per 100 000, followed by the upper-middle-income (13·2 [10·9–15·3] deaths per 100 000) and lower-middle-income (19·1 [13·2–24·3] deaths per 100 000) groups (table 1; appendix 1 p 39). This pattern contrasted with age-standardised incidence rates, which were highest in the high-income group. The highest mortality rate was in the low-income group, at 43·8 (31·7–56·0) deaths per 100 000—almost six times higher than in the high-income group (table 1; appendix 1 p 39).

**Figure 2:**
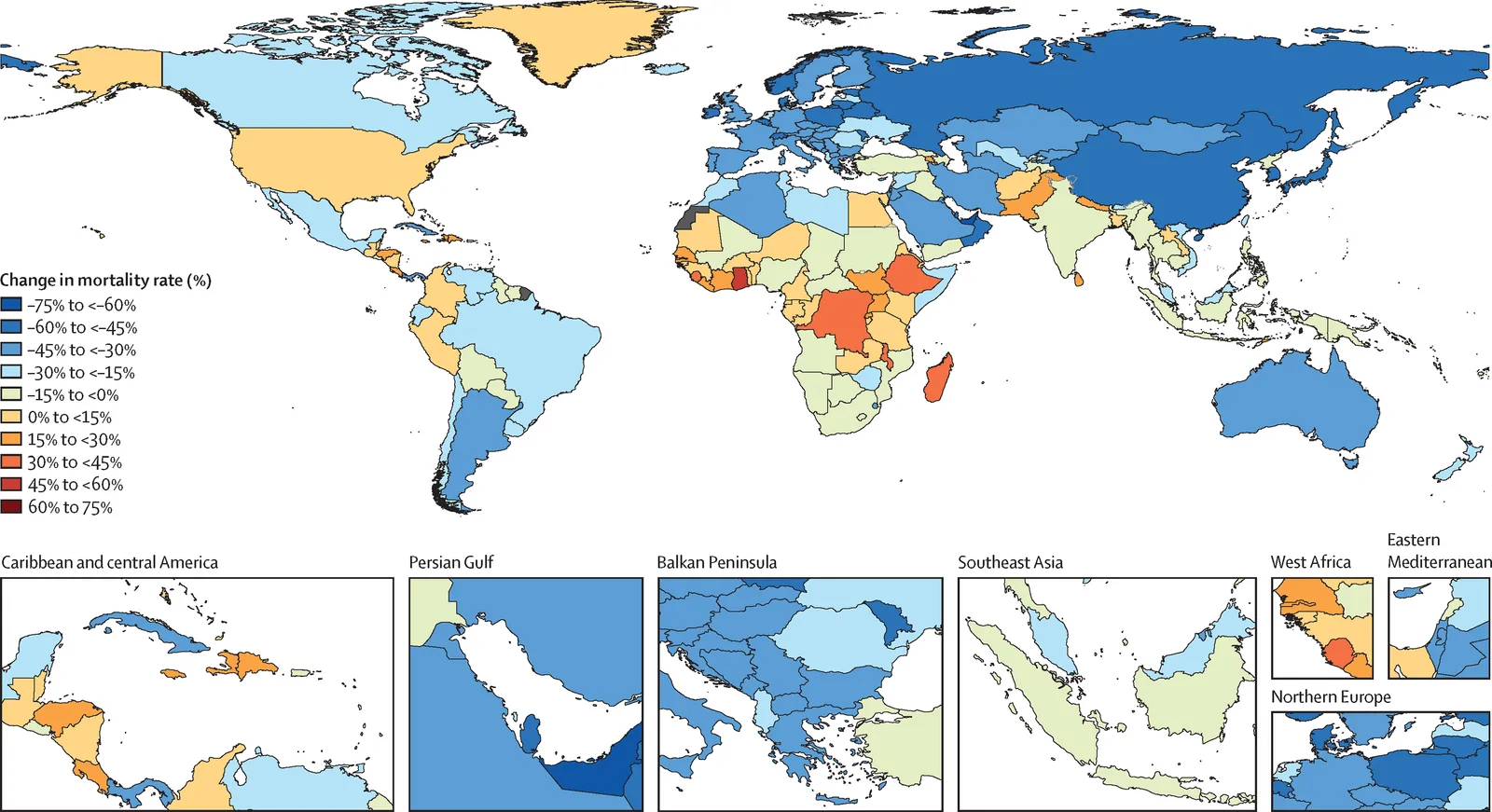
Change in age-standardised mortality rates attributable to road injuries, 2010–23

Between 1990 and 2023, the global age-standardised mortality from road injuries decreased by 32·3% (95% UI 6·1 to 49·0; table 1; appendix 1 p 39). Considerable reductions occurred in the high-income group (61·1% [58·5 to 62·7]) and the upper-middle-income group (54·5% [26·0 to 66·6]). The lower-middle-income and low-income groups had no detectable changes in age-standard mortality rates from 1990 to 2023 (table 1; appendix 1 pp 14–16, 39). Focusing on the period from 2010 to 2023, which coincides with the years of the UN Decades of Action for Road Safety, the change in global age-standardised mortality rates was –13·3% (–25·8 to 3·8). Significant reductions were observed in 80 countries and territories, 12 of which had decreases exceeding 50% (figure 2; appendix 1 pp 17–19). However, six countries had significant increases during this period, ranging from 13·2% (6·3 to 20·3) in the USA to 48·5% (8·3 to 107·5) in Ghana.

Motor vehicle road injuries accounted for the largest share of road injury deaths worldwide in 2023 (518 000 deaths [95% UI 417 000–648 000], 38·6% of the total), followed by pedestrian injuries (401 000 deaths [295 000–532 000], 29·9%; appendix 1 pp 13, 40). Although motorcyclists represented only 13·2% (6·70 million [5·71–7·91] cases) of road injury incidence, they accounted for 23·8% (319 000 deaths [217 000–416 000]) of road injury deaths. Although motor vehicle and cyclist road injury incidence rates were highest in high-income countries, the corresponding mortality rates were lowest (table 1).

Progress on addressing road injury mortality since 1990 has been uneven, with motorcyclist road injuries showing the most heterogeneous patterns, ranging from a decrease of 44·1% (95% UI 36·4 to 52·4) in the high-income group to a change of 106·5% (–10·0 to 381·5) in the low-income group (table 1; appendix 1 p 41). Despite decreases in age-standardised incidence rates, the trajectories of age-standardised mortality rates diverged between income groups for all road injury types, with the greatest reductions in the high-income group and no significant change in the low-income group (table 1).

Globally, road injuries resulted in 66·7 million (95% UI 51·5–79·4) YLLs and 8·56 million (6·25–11·2) YLDs, together accounting for 75·3 million (59·8–89·2) DALYs in 2023 (appendix 1 pp 20–28). Age-standardised YLL rates were nearly six times higher in the low-income group (2134·9 [95% UI 1546·6–2734·4] per 100 000) than in the high-income group (381·5 [362·7–401·3]) (appendix 1 pp 20–22, 42), whereas YLD rates showed a contrasting pattern, being highest in the high-income group (113·7 [82·9–152·5] per 100 000; appendix 1 pp 23–25, 43). The age-standardised DALY rate was 2229·4 (1637·2–2840·5) per 100 000 in the low-income group compared with 495·3 (457·4–541·8) per 100 000 in the high-income group (appendix 1 pp 26–28, 44). Large disparities in DALY rates were also seen at the country level (appendix 1 pp 29–31, 45). Between 1990 and 2023, global age-standardised YLL, YLD, and DALY rates all decreased significantly (appendix 1 pp 20–28), although reductions in YLL and DALY rates were confined to the high-income and upper-middle-income groups (appendix 1 p 46).

**Figure 3:**
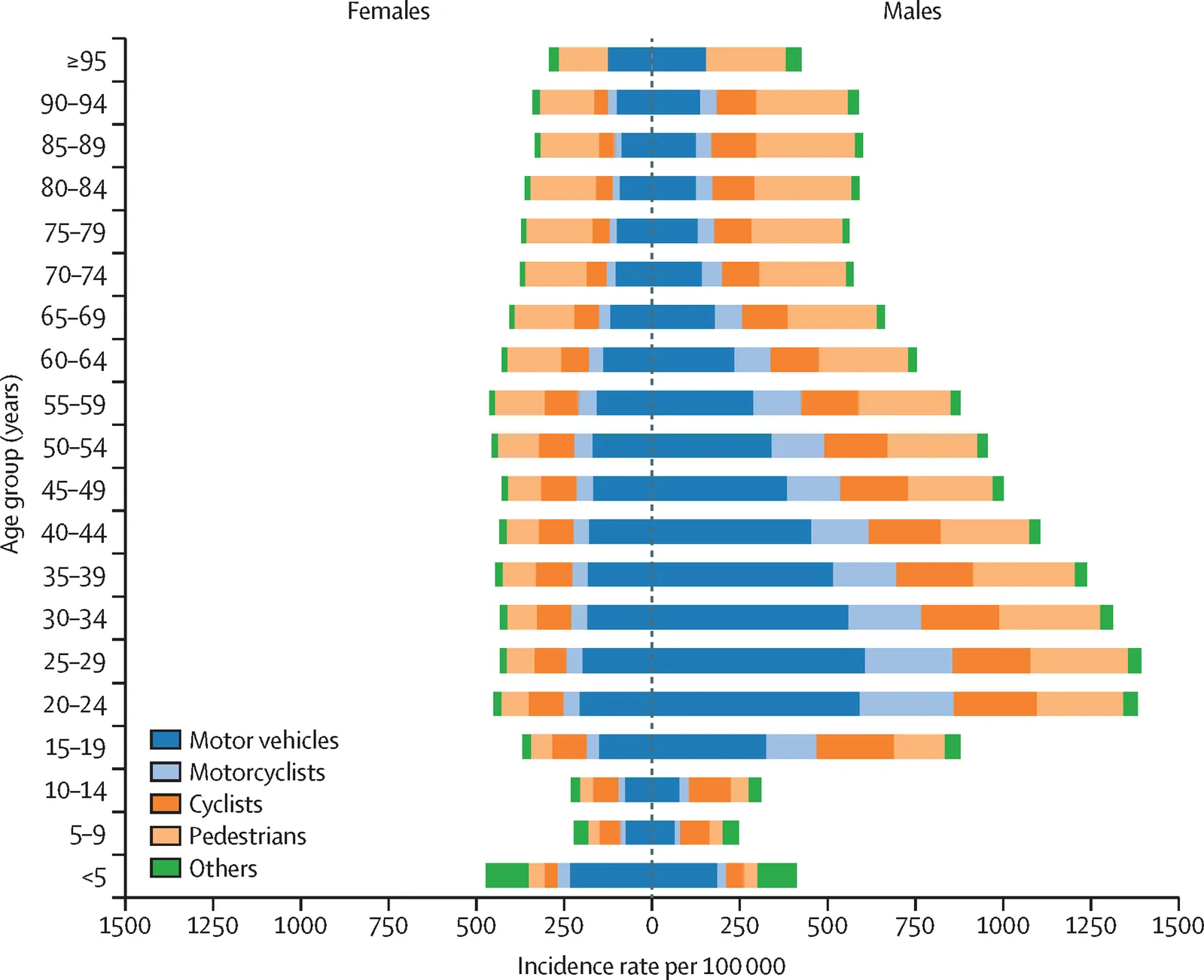
Global road injury incidence rate stratified by sex, age group, and Level 4 road injury causes, 2023

Road injury incidence rates were consistently higher in males than in females across all age groups in 2023, except among children younger than 5 years (figure 3). In males, incidence peaked in young adults aged 20–29 years, whereas in females, the highest rates were observed in young children (younger than 5 years). Age and sex patterns by road user type are shown in appendix 1 (p 47).

The greatest mortality burden was concentrated in people younger than 39 years (appendix 1 pp 48–49). Road injuries were the leading Level 3 cause of death for individuals aged 10–39 years globally and the second leading cause of death in children aged 5–9 years (figure 1). In males aged 20–24 years, road injuries accounted for 20·7% (95% UI 15·6–24·9) of all deaths globally, corresponding to 98 000 (95% UI 71 600–121 000) deaths (appendix 1 pp 32, 48).

The most common nature of injury from road injuries was fractures, constituting 38·1% (95% UI 34·6–41·9) of all non-fatal road injury cases, corresponding to 19·4 million (95% UI 16·7–22·1) cases (table 2). Minor injuries ranked second, accounting for 35·3% (95% UI 31·2–39·1) of all non-fatal road injury cases, corresponding to 18·0 million (95% UI 15·5–20·7) cases. Head injuries occurred in 10·4% (95% UI 9·0–11·8) of road injury cases, corresponding to 5·27 million (95% UI 4·43–6·30) cases. Most head injuries were classified as moderate or severe traumatic brain injuries (appendix 1 p 50), representing 7·1% (95% UI 5·9–8·3) of all non-fatal road injury cases, corresponding to 3·62 million (95% UI 2·92–4·35) cases (table 2). Low-income, lower-middle-income, and upper-middle-income groups had a disproportionately high proportion of moderate or severe traumatic brain injuries, constituting more than 70% of head injuries (appendix 1 p 50). Motorcyclist road injuries had the highest proportion of head injuries of all road user types (13·9% [95% UI 11·3–16·6]), corresponding to 930 000 cases (95% UI 730 000–1 140 000), of which 78·9% (734 000 cases [95% UI 549 000–914 000]) were moderate or severe traumatic brain injuries (appendix 1 pp 33, 51). Of the total YLDs attributable to road injuries, fractures accounted for 47·2% (95% UI 42·8–51·1), corresponding to 4·05 million (95% UI 2·80–5·60) YLDs. Head injuries accounted for 21·3% (95% UI 17·8–24·8) of road injury YLDs, corresponding to 1·82 million (95% UI 1·27–2·46) YLDs, with moderate or severe traumatic brain injuries alone resulting in 1·58 million (1·10–2·12) YLDs (table 2).

**Table 2:** Nature-of-injury incident cases and YLDs attributable to road injuries, and the share of each nature-of-injury category in total road injury incidence and YLDs, globally and across World Bank income groups, 2023

	**Incidence**		**YLDs**	
	Incident cases (thousands), 2023	Percentage of all road injury cases, 2023	YLDs (thousands), 2023	Percentage of all road injury YLDs, 2023
**Fractures**				
Global	19 400 (16 700–22 100)	38·1% (34·6–41·9)	4050 (2800–5600)	47·2% (42·8–51·1)
Low income	1370 (1210–1540)	38·0% (34·2–42·3)	187 (131–260)	42·1% (37·3–46·3)
Lower-middle income	5290 (4550–6040)	38·3% (34·7–42·6)	878 (608–1210)	43·7% (39·0–48·1)
Upper-middle income	8510 (7280–9830)	38·6% (35·0–42·9)	1840 (1260–2560)	46·7% (42·2–50·9)
High income	4200 (3630–4750)	36·9% (33·5–40·9)	1140 (789–1590)	52·4% (48·4–55·7)
**Head injuries**				
Global	5270 (4430–6300)	10·4% (9·0–11·8)	1820 (1270–2460)	21·3% (17·8–24·8)
Low income	354 (302–409)	9·8% (8·5–11·3)	85·6 (60·0–116)	19·3% (16·6–21·9)
Lower-middle income	1480 (1250–1760)	10·7% (9·2–12·2)	426 (297–578)	21·3% (18·2–24·6)
Upper-middle income	2500 (2080–3010)	11·3% (9·8–12·9)	981 (685–1320)	24·9% (20·7–29·2)
High income	940 (758–1150)	8·3% (6·9–9·9)	328 (225–441)	15·2% (12·6–17·7)
**Moderate or severe traumatic brain injury**				
Global	3620 (2920–4350)	7·1% (5·9–8·3)	1580 (1100–2120)	18·5% (15·3–21·8)
Low income	248 (206–290)	6·9% (5·7–8·1)	77·6 (54·5–105)	17·5% (14·9–20·1)
Lower-middle income	1070 (875–1280)	7·8% (6·5–9·1)	388 (271–526)	19·4% (16·5–22·5)
Upper-middle income	1860 (1500–2270)	8·4% (7·0–9·9)	892 (626–1200)	22·7% (18·7–26·8)
High income	427 (337–520)	3·8% (3·0–4·6)	218 (154–289)	10·1% (8·1–12·2)
**Minor traumatic brain injury**				
Global	1660 (1200–2270)	3·3% (2·4–4·4)	244 (168–334)	2·9% (2·5–3·3)
Low income	106 (74·4–147)	2·9% (2·1–4·1)	8·00 (5·46–11·1)	1·8% (1·6–2·1)
Lower-middle income	400 (286–549)	2·9% (2·1–4·0)	38·4 (26·1–52·9)	1·9% (1·7–2·2)
Upper-middle income	637 (453–878)	2·9% (2·1–3·9)	88·5 (59·7–121)	2·2% (1·9–2·6)
High income	513 (358–693)	4·5% (3·3–6·0)	109 (73·6–150)	5·0% (4·3–5·9)
**Spinal injury**				
Global	98·0 (68·1–137)	0·2% (0·1–0·3)	805 (576–1020)	9·5% (7·2–11·9)
Low income	5·03 (3·47–7·04)	0·1% (0·1–0·2)	32·9 (24·5–41·2)	7·5% (5·8–9·3)
Lower-middle income	21·7 (15·1–30·4)	0·2% (0·1–0·2)	154 (112–193)	7·8% (5·9–9·6)
Upper-middle income	38·5 (27·0–54·7)	0·2% (0·1–0·2)	318 (228–402)	8·2% (6·1–10·2)
High income	32·7 (22·7–45·3)	0·3% (0·2–0·4)	299 (213–386)	13·9% (10·3–17·8)
**Other injuries**				
Global	6970 (5840–8360)	13·7% (12·0–15·9)	762 (549–1020)	8·9% (7·9–10·1)
Low income	503 (434–588)	14·0% (12·1–16·3)	48·1 (33·7–65·3)	10·9% (9·3–12·7)
Lower-middle income	1930 (1600–2300)	14·0% (12·1–16·3)	197 (143–263)	9·8% (8·5–11·5)
Upper-middle income	3070 (2520–3760)	13·9% (12·1–16·3)	308 (222–417)	7·8% (6·9–9·0)
High income	1470 (1250–1750)	12·9% (11·3–15·0)	208 (148–279)	9·6% (8·7–10·6)
**Amputations**				
Global	675 (491–929)	1·3% (1·0–1·8)	572 (408–793)	6·7% (5·8–7·8)
Low income	52·2 (37·2–73·9)	1·5% (1·1–2·1)	40·5 (29·5–54·5)	9·1% (8·2–10·2)
Lower-middle income	200 (145–279)	1·4% (1·1–2·0)	174 (126–234)	8·7% (7·8–9·6)
Upper-middle income	321 (234–444)	1·5% (1·1–2·0)	275 (191–385)	7·0% (6·0–8·4)
High income	101 (69·1–145)	0·9% (0·6–1·3)	81·8 (52·8–124)	3·8% (3·0–4·8)
**Burns**				
Global	501 (302–751)	1·0% (0·6–1·5)	361 (268–466)	4·2% (3·9–4·6)
Low income	44·0 (25·6–66·5)	1·2% (0·7–1·9)	42·4 (31·1–54·0)	9·6% (8·5–10·9)
Lower-middle income	156 (93·0–231)	1·1% (0·7–1·7)	144 (105–182)	7·2% (6·5–8·0)
Upper-middle income	229 (141–336)	1·0% (0·6–1·5)	143 (106–193)	3·6% (3·3–4·1)
High income	71·2 (42·5–110)	0·6% (0·4–1·0)	31·8 (20·8–48·9)	1·5% (1·2–1·8)
**Minor injuries**				
Global	18 000 (15 500–20 700)	35·3% (31·2–39·1)	191 (91·0–366)	2·2% (1·2–3·8)
Low income	1280 (1100–1440)	35·4% (31·1–39·2)	6·63 (3·17–12·7)	1·5% (0·8–2·6)
Lower-middle income	4740 (4090–5470)	34·3% (30·1–38·1)	31·9 (15·0–60·7)	1·6% (0·8–2·7)
Upper-middle income	7380 (6280–8610)	33·5% (29·2–37·3)	71·9 (34·3–137)	1·8% (1·0–3·1)
High income	4560 (3970–5200)	40·1% (35·7–44·0)	80·1 (37·8–155)	3·7% (2·0–6·3)

Data in parentheses are 95% uncertainty intervals. Count data are presented to three significant figures. In the Global Burden of Diseases, Injuries, and Risk Factors Study framework, head injury corresponds to the traumatic brain injury category, which is disaggregated only into minor traumatic brain injury and moderate or severe traumatic brain injury. Non-traumatic brain injury head trauma is captured elsewhere: skull and facial fractures under the fractures categories, and superficial head lesions under open wounds and other injuries. YLDs=years of life lived with disability.

## Discussion

This study provides a comprehensive global assessment of road injury burden from 1990 to 2023. With nearly 51 million incident cases, 1·34 million deaths, and over 75 million DALYs estimated in 2023, road injuries remain one of the leading causes of disease burden worldwide. Although global age-standardised incidence, mortality, and DALY rates have declined, progress varied by geography. Our findings align with the WHO Global Status Report on Road Safety 2023,^2^ which estimated 1·19 million deaths globally in 2021. Although exact point estimates differ due to methodological divergence, both studies found a disproportionate mortality burden in low-income and middle-income countries (LMICs).^2^ Overall, road injuries present a heterogeneous epidemiological landscape, where progress and stagnation stand in juxtaposition.

Global reductions in road injury incidence and mortality, driven largely by trends in the high-income group, reflect the importance of strengthened regulation, improved policy, heightened awareness, and post-crash and trauma care.^2^ In high-income countries, the age-standardised incidence rate decreased by 49·0% and the mortality rate decreased by 61·1% from 1990 to 2023. These improvements have occurred alongside a shift in safety philosophy that reframes road safety as a public health issue rooted in preventable system failures rather than individual driver error.^21^ The Vision Zero principle, which holds that no road death is acceptable or inevitable, exemplifies this shift and, since its introduction in Sweden in 1997,^22^ has been adopted by many countries.^23^ Closely related is the Safe System approach, premised on designing transport systems that accommodate human vulnerability and error, and endorsed by WHO and the UN for the Second Decade of Action for Road Safety 2021–30.^10^ However, implementing these frameworks requires sustained political commitment, cross-sectoral coordination, and technical and infrastructural capacity;^23^, ^24^ prerequisites that can be difficult to realise in low-resource settings.

Profound inequity defines road injury burden, with 90% of deaths occurring in LMICs. The mortality rate in the low-income group was nearly six times higher than in the high-income group, more than three times higher than in the upper-middle-income group, and more than two times higher than in the lower-middle-income group. Despite the existence of international guidelines, progress in LMICs has been constrained by limited national capacity to operationalise them.^25^ In many LMICs, rapid motorisation and urbanisation have outpaced the development of road safety measures, leaving large proportions of road users exposed to high-risk environments.^26^ Variations in regulatory standards illustrate these gaps: in 2022, 86% of high-income countries mandated seatbelts and seatbelt anchorages, compared with 26% of low-income countries.^2^ This disparity is even more pronounced for advanced safety features such as front-impact and side-impact protection, which are mandated in less than 10% of LMICs compared with 76% of high-income countries.^2^ Road infrastructure quality similarly lags, with most road length in low-income countries rated as unsafe.^27^ These deficiencies are further compounded by underdeveloped post-crash response and data systems, hindering timely care and the ability to identify intervention priorities.^26^, ^28^, ^29^

The disparities in road safety standards across income groups coincide with an asymmetry in mortality–morbidity burden and differences in the nature of injuries. Road injuries resulted in high levels of premature mortality in LMICs, with age-standardised YLL rates three to six times higher in the low-income group than in the high-income group. By contrast, road injury burden in the high-income group manifested primarily in disability, reflected in higher YLD rates. Road injuries in low-income, lower-middle-income, and upper-middle-income groups were also more severe in nature; more than 70% of head injuries resulting from road injuries in these groups were moderate to severe traumatic brain injuries. These patterns are consistent with lower rates of helmet and seatbelt use, suboptimal speed regulation, delayed access to post-crash care, and the lack of essential trauma and surgical resources in LMICs.^2^, ^26^, ^30^, ^31^, ^32^ However, it is important to acknowledge that some of the discrepancies in mortality and morbidity patterns observed across the income groups might also reflect how road injuries are ascertained in different contexts, particularly in LMICs, where under-reporting is pervasive.^12^

Addressing these challenges demands prioritising solutions that deliver the largest reductions in road injury mortality per unit of investment.^33^ WHO-led cost-effectiveness analyses have identified scalable, context-specific intervention packages for LMICs, ranging from speed management, random breath testing, and helmet enforcement in southeast Asia to a broader package including seatbelt enforcement in eastern sub-Saharan Africa.^34^, ^35^ Strengthening health-system capacity is equally crucial. Evidence suggests that more than 200 000 lives could be saved each year through enhanced trauma care coverage in LMICs.^30^ Although policy priorities might differ by country, cross-setting analysis confirms that comprehensive strategies yield the greatest gains.^33^ India's Zero Fatality Corridor initiative exemplifies the potential of multisectoral action; combining engineering, enforcement, and emergency response, it achieved a 43% reduction in mortality within 4 years and has since expanded to more than 100 national highways across India,^36^ illustrating that meaningful progress is possible with targeted investment and multisectoral coordination.

For high-income countries, decreases in road injury burden are not assured; the USA had some of the largest increases in road injury mortality rates between 2010 and 2023. The increase, driven predominantly by pedestrian road injuries, has been associated with the uptake of larger vehicles and changes in road design.^37^ In other high-income countries, changing transport patterns are redefining the distribution of road injury burden. Cyclist injuries have surpassed pedestrian injuries to become the second leading cause of road injury DALYs in several Nordic countries, whereas in high-income Asia Pacific countries such as Japan and Singapore, they now constitute the primary cause of road injury cases. A growing ageing population also necessitates an essential reassessment of infrastructure and technology, demanding inclusive and adaptable engineering that accommodates age-related needs.^38^, ^39^ Road safety strategies must evolve alongside shifting road user profiles and transport behaviour to sustain these hard-earned gains and further advance road injury reduction.

Beyond the human toll, the economic cost of road injuries is enormous, projected at $1·8 trillion between 2015 and 2030.^40^ Yet, road safety remains underfunded. Within health-related official development assistance, less than 1% was directed to trauma care.^41^ This chronic underfunding is further compounded by a broader contraction in official development assistance in recent years.^42^ The World Bank estimated that a minimum of $200 billion of additional investment is required to meet the global road safety target of halving the number of global road injury deaths and injuries by 2030.^11^ Recasting road safety as a core sustainability concern and positioning it squarely within the environmental, social, and governance agenda is essential for mobilising the scale of resources needed to achieve these goals.^43^

This study has several limitations. First, road injury data are not routinely captured across all countries or disaggregated by age-sex groups, and empirical data on non-fatal injuries and long-term disability are often sparse. In such cases, we relied on covariates and spatiotemporal models to interpolate missing information; uncertainty intervals are consequently wide for some locations and road user types and should be interpreted with caution, and some residual uncertainty remains, particularly in data-sparse settings. These data gaps highlight the importance of strengthening road injury surveillance systems. Second, GBD disability weights capture only the direct health consequences of injury and do not capture broader social, economic, and psychological effects, which remain important for policy considerations and rehabilitation planning. Third, GBD assigns a single nature-of-injury category per case, selecting the category associated with the greatest health loss; when multiple concurrent injuries occur, total morbidity burden might be underestimated, although this applies mainly to cases with substantial multimorbidity profiles. Fourth, subnational variations are not captured in this analysis; future work could explore subnational GBD estimates. Fifth, our analysis is descriptive and does not evaluate the causal effects of specific policies or interventions. Additionally, the impact of the COVID-19 pandemic must be considered when interpreting findings, as disrupted mobility patterns and constrained access to emergency care between 2020 and 2022 might have influenced observed trends independently of road safety policy.

As we approach the midpoint of the Second Decade of Action for Road Safety, global progress in reducing road injury burden has been marked by heterogeneity. Disparities in road injury outcomes expose deep inequities in political governance, technological capacity, and access to health care. Existing investments fall short of what is needed for meaningful progress. While cost-effective measures exist, realising them demands structural reform and long-term commitment.

### GBD 2023 Road Injuries Collaborators

### Affiliations

### Contributors

### Data sharing

Input data used in these analyses are available on the Global Health Data Exchange GBD 2023 website at https://ghdx.healthdata.org/gbd-2023. All results from this study are publicly accessible. To download estimates produced in these analyses, please visit the GBD Results tool.^44^ All GBD estimates, including the different measures, metrics, causes, locations, age groups, sex, and years, are publicly available via the GBD Results tool for registered users.^44^

## Declaration of interests

Saira Afzal reports support for the present manuscript from the Institute of Public Health Lahore (study material, manuscripts, medical writings, and library resources); grants or contracts from the Dean office, Institute of Public Health Lahore (intellectual contributions and time protection); honoraria for experts, lectures, visiting speakers, and educational seminars were provided by the Dean Institute of Public Health Lahore; support for attending meetings or travel was provided by the Dean Institute of Public Health, Lahore Pakistan; participation on a data safety monitoring board or advisory board as member of the Pakistan National Bioethics Committee, member of the Institutional Review Board of Fatima Jinnah Medical University, member of the Ethical Review Board and Data Monitoring Board of Institute of Public Health Lahore Pakistan, in charge of the Clinical Research Organization of King Edward Medical University, member of the Annals of King Edward Medical University Advisory Board; leadership or fiduciary role in other board, society, committee, or advocacy group, paid or unpaid, as a member of Pakistan Higher Education Commission Research Committee, member of Pakistan Medical and Dental Commission Research and Journals Committee, member of Pakistan National Bioethics Committee, member of Pakistan Society of Internal Medicine, member of Pakistan Association of Medical Editors, member of Medical Microbiology and Infectious Diseases Society, Fellow of Leads International, Fellow of Faculty of Public Health UK, Fellow of College of Physicians and Surgeons Pakistan; received computer software and equipment from Bergen University Norway for research writing; and other financial or non-financial interests as Dean of Public Health Institute of Public Health Birdwood Lahore, member of National Bioethics Committee Pakistan, and member of Prime Minister Taskforce on HCV, HIV, and Infectious Diseases, outside the submitted work. Muhammad Shahzad Aslam reports grants or contracts from the Xiamen University Malaysia Research Fund (XMUMRF) for grant number XMUMRF/2025-C15/ITCM/0006 (“Therapeutic and toxicity evaluation of selected medicinal herbs for NAFLD: exploring the inter-organelle contact sites modulation theory,” co-investigator, January, 2025–December, 2027, ongoing), grant number XMUMRF/2023-C11/ISEM/0041 (“Children's rights education in the early years of divorce: an exploration of adolescents’ perspectives,” co-investigator, January, 2023–December, 2025, ongoing), and grant number XMUMRF/2026-C17/ITCM/0007 (“Olfactory stimulation and autonomic regulation in menstrual disorders,” co-investigator, January, 2026–December, 2028, ongoing; all internal XMUMRF research grants administered by Xiamen University Malaysia; funds disbursed to institutional research account only; no salary, honoraria, or personal payments to author), outside the submitted work. Ruhai Bai reports support for the present manuscript from the Open Research Fund of Key Laboratory of Endocrine Glucose & Lipids Metabolism and Brain Aging, Ministry of Education (number 2023JYBZDSYS012). Ovidiu Constanin Baltatu reports support for the present manuscript from the National Council for Scientific and Technological Development Fellowship (CNPq, 304224/2022-7) and from Anima Institute; leadership or fiduciary role in other board, society, committee, or advocacy group, paid or unpaid, at VividiWise Analytics as managing partner and São José dos Campos Tech Park – CITE as biotech advisory board member, outside the submitted work. Sonu Bhaskar reports grants or contracts from the Japan Society for the Promotion of Science (JSPS), Japanese Ministry of Education, Culture, Sports, Science, and Technology (MEXT), Grant-in-Aid for Scientific Research (KAKENHI; grant ID: 23KF0126), and from JSPS and the Australian Academy of Science (JSPS International Fellowship, grant ID: P23712); payment or honoraria for lectures, presentations, speakers bureaus, manuscript writing, or educational events from the Japan Stroke Society, Japan (honoraria for invited keynote presentation at the 50th Annual Meeting of the Japan Stroke Society, Osaka) and from Semey Medical University, Kazakhstan (honorarium for invited professorship and teaching course in neurocardiovascular therapy, Semey Medical University, Kazakhstan); support for attending meetings or travel from the Japan Stroke Society, Japan (travel award for invited keynote presentation at the 50th Annual Meeting of the Japan Stroke Society, Osaka) and from Asialink, The University of Melbourne (travel support for opening invited keynote at 31st Asialink Leaders Foundation Week Program 2026, Melbourne); and leadership or fiduciary role in other board, society, committee, or advocacy group, paid or unpaid, as district chair, Diversity, Equity, Inclusion, and Belonging, Rotary District 9675, Sydney, Australia; chair, founding member, and manager, Global Health and Migration Hub Community, Global Health Hub Germany, Berlin, Germany; editorial board member, PLOS One, BMC Neurology, Frontiers in Neurology, Frontiers in Stroke, Frontiers in Public Health, Journal of Aging Research, Diagnostics, and BMC Medical Research Methodology; member, College of Reviewers, Canadian Institutes of Health Research, Government of Canada; Director of Research, World Headache Society, Bengaluru, India; expert adviser or reviewer, Cariplo Foundation, Milan, Italy; visiting director, National Cerebral and Cardiovascular Center, Department of Neurology, Division of Cerebrovascular Medicine and Neurology, Suita, Osaka, Japan; member, Scientific Review Committee, Cardiff University Biobank, Cardiff, UK; chair, Rotary Reconciliation Action Plan; and health care and medical adviser, Japan Connect, Osaka, Japan, outside the submitted work. Andreas K Demetriades reports leadership or fiduciary role in other board, society, committee, or advocacy group, paid or unpaid, as a non-stipendiary role as past President, EANS (European Association of Neurosurgical Societies), non-stipendiary role as steering committee member, AO Spine Knowledge Forum, non-stipendiary role as board member, EANS Foundation, and non-stipendiary role as board member, Global Neuro Foundation, outside the submitted work. Ibrahim Farahat El Bayoumy reports leadership or fiduciary role in other board, society, committee, or advocacy group, paid or unpaid, as Editor in Chief for Recent Trends in Infectious Diseases STM Journals and editorial member in Texila American University Journals, outside the submitted work. Andre Faro reports support for the present manuscript from the National Council for Scientific and Technological Development (CNPq, Brazil; CNPq Research Fellow, Brazil) and CNPq Researcher Fellow. Richard Charles Franklin reports support for attending meetings or travel from ACTM—annual Conference 2024–25; and leadership or fiduciary role in other board, society, committee, or advocacy group, paid or unpaid, as President—Australasian College of Tropical Medicine, President—Kidsafe Australia, board member Royal Life Saving Society Australia, and board member Auschem Training, outside the submitted work. Irena M Ilic reports support for the present manuscript from the Ministry of Science, Technological Development, and Innovation of the Republic of Serbia (number 451-03-137/2025-03/200110). Joseph Johnson reports consulting fees from Stryker Inc (medical education consultant); and other financial or non-financial interests from research support provided by Ortho Xcel, outside the submitted work. Jagdish Khubchandani reports grants or contracts from Merck Sharpe Dohme, Inc (research unrelated to this study but concerning vaccine hesitancy), National Science Foundation, USA (research unrelated to this study but concerning public health message inconsistency), and NM-INBRE (NIH P20GM103451 subgrant; research unrelated to this study but concerning pain disorders in American adults); support for attending meetings or travel from NATCO: The Organization for Donation and Transplant Professionals (invited plenary speaker at annual event; paid for travel and accommodation); and leadership or fiduciary role in other board, society, committee, or advocacy group, paid or unpaid, as secretary, World Association of Medical Editors (not paid), member, Evidentiary Committee, American Public Health Association (not paid), member, US EPA Good Neighbor Environmental Board (not paid; assignment until 2025), and member, US FDA Digital Health Advisory Committee (not paid; assignment until 2025), outside the submitted work. Kewal Krishan reports other financial or non-financial interests from non-financial support from the UGC Centre of Advanced Study, CAS II, awarded to the Department of Anthropology, and the RUSA 2.0 grant awarded by the Ministry of Education to Panjab University, Chandigarh, India. Ja-Ho Leigh reports support for the present manuscript from the Ministry of Land, Infrastructure, and Transport of South Korea (NTRH RF-2026002); and grants or contracts from the Korean National Police Agency Grant for “Development of intelligent (AI) judgment algorithm for verification of high-risk drivers’ driving fitness and commercialization of VR driving simulator” (2022–24) and the Korean National Police Agency Grant for “Development of driving ability assessment system for improvement of conditional driver's license system” (2026–27; payment made to institution for both), outside the submitted work. Ming-Chieh Li reports support for the present manuscript from the National Science and Technology Council, Taiwan (NSTC 115-2410-H-003-022; 115-2621-M-003-002); and leadership or fiduciary role in other board, society, committee, or advocacy group, paid or unpaid, as technical editor, Journal of the American Heart Association, outside the submitted work. Ted R Miller reports grants or contracts from the Network of Employers for Traffic Safety and the AB InBev Foundation (payments to Ted R Miller's institution), outside the submitted work. Rafael Silveira Moreira reports grants or contracts from the CNPq Research Productivity Scholarship (Rafael Silveira Moreira is a research productivity fellow by CNPq [National Council for Scientific and Technological Development]; the scholarship registration number is 308986/2025-3), outside the submitted work. Shuhei Nomura reports support for the present manuscript from the Ministry of Education, Culture, Sports, Science, and Technology of Japan (grant 24H00663). Bogdan Oancea reports support for the present manuscript from UEFISCDI (project PN-IV-P6-6.1-CoEx-2024-0196, contract number 17/2.03.2026). Georgios D Panos reports payment or honoraria for lectures, presentations, speakers bureaus, manuscript writing, or educational events from Thea (payment to Georgios D Panos) and from Bayer and Roche (payment to Georgios D Panos through their institution); and support for attending meetings or travel from Thea, Bayer, and Roche (payment made to the agency of the meeting or congress), outside the submitted work. Roberto Passera reports being member of the data safety monitoring board for the clinical trial “Consolidation with ADCT-402 (loncastuximab tesirine) after immunochemotherapy: a phase II study in BTKi-treated/ineligible relapse/refractory mantle cell lymphoma (MCL) patients”—FIL, Fondazione Italiana Linfomi, Alessandria (Italy; no payment received); and leadership or fiduciary role in other board, society, committee, or advocacy group, paid or unpaid, as member of the EBMT Statistical Committee, European Society for Blood and Marrow Transplantation, Paris (France), member of the FIL Statistical Committee, Fondazione Italiana Linfomi, Alessandria (Italy), and past member 2020–23 (biostatistician) of the IRB/IEC Comitato Etico AO SS Antonio e Biagio Alessandria-ASL AL-VC (Italy; all no payment received), outside the submitted work. Amy E Peden reports support for the present manuscript from the Australian National Health and Medical Research Council (grant number: APP2009306). Samreen Saleem reports grants or contracts from Health Services Academy, Islamabad, Pakistan; and payment or honoraria for lectures, presentations, speakers bureaus, manuscript writing, or educational events from Health Services Academy, Islamabad, Pakistan, outside the submitted work. Yoseph Leonardo Samodra reports grants or contracts from NTU EPM & NSTC Taiwan (post-doctoral fellow contract); leadership or fiduciary role in other board, society, committee, or advocacy group, paid or unpaid, as a co-founder of Benang Merah Research Center (benangmerah.net); and other financial or non-financial interests from Jago Beasiswa (idebeasiswa.net; postgraduate scholarship mentor), outside the submitted work. Saurab Sharma reports grants or contracts from the Medical Research Future Funds (MRFF) Early/Mid-Career Research grant (as CI; paid to Neuroscience Research Australia, Sydney), a seed funding grant from the ANZ Back Pain Clinical Trials Networks, supported by the National Health and Medical Research Council (NHMRC; paid to Neuroscience Research Australia, Sydney), and an NHMRC Investigator Grant (Emerging Leadership 1/EL1) from the National Health and Medical Research Council (received 2025, commenced 2026; paid to the University of Sydney, Sydney; the funding bodies had no influence on Saurab Sharma's research or its reporting); support for attending meetings or travel from the Australian Pain Conference (2025 plenary lecture, unrelated to this paper; registration, travel, and accommodation costs reimbursed to Saurab Sharma); and leadership or fiduciary role in other board, society, committee, or advocacy group, paid or unpaid, as co-chair, IASP Global Year 2025 (“Pain management, research, and education in low/middle-income settings”); board member, IASP Global Alliance for Partners in Pain Advocacy (GAPPA); editorial board member, Pain Research Forum of the IASP; and associate editor, Journal of Orthopedic & Sports Physical Therapy and Physiotherapy (all unpaid), outside the submitted work. Vishal Sharma reports other financial or non-financial interests from DFSS (MHA)'s research project (DFSS28(1)2019/EMR/6) at the Department of Forensic Science (formerly Institute of Forensic Science & Criminology), Panjab University, Chandigarh, India; the RUSA 2.0 grant from MOE to Panjab University; and the PAIR grant (ANRF/PAIR/2025/000006/PAIR) to Panjab University from ANRF. Sebastian Straube reports leadership or fiduciary role in other board, society, committee, or advocacy group, paid or unpaid, as editorial board member, SN Comprehensive Clinical Medicine; board member, MSI Foundation (payments to Sebastian Straube); and member of the Scientific Advisory Committee—New Alberta Care First Automobile Insurance System, Ontario Tech University (payments to Sebastian Straube), outside the submitted work. Rafael Tabarés-Seisdedos reports grants or contracts from the Valencian Regional Government's Ministry of Education (PROMETEO/CIPROM/2022/58) and the Spanish Ministry of Science, Innovation and Universities (PID2021-129099OB-I00; the funders were not involved in the design of the manuscript or the decision to submit it for publication, nor will they be involved in any aspect of the study's conduct), outside the submitted work. Jansje Henny Vera Ticoalu reports leadership or fiduciary role in other board, society, committee, or advocacy group, paid or unpaid, as a co-founder of Benang Merah Research Center (benangmerah.net), outside the submitted work. Era Upadhyay reports patents planned, issued or pending for “A system and method of reusable filters for anti-pollution mask” (published), “A system and method for electricity generation through crop stubble by using microbial fuel cells” (published), “A system for disposed personal protection equipment (PPE) into biofuel through pyrolysis and method” (published), “A novel herbal pharmaceutical aid for formulation of gel and method thereof” (published), “Herbal drug formulation for treating lung tissue degenerated by particulate matter exposure” (published), “A method to transform cow dung into the wall paint by using natural materials and composition thereof” (published), “Biodegradable packaging composition and method of preparation thereof” (filed), “Eco-friendly bio-shoe polish from banana and turmeric” (filed), “Honey-based polyherbal syrup composition to treat air pollution-induced inflammation and preparation method thereof” (filed), “Process for preparing a caffeine free, antioxidant and nutrient rich beverage” (filed), “Polyherbal oral mist composition for dual relief and method for preparation thereof” (filed), and “Method for fabricating sustainable waterproof material sheets from LDPE plastic wastes” (filed); and leadership or fiduciary role in other board, society, committee, or advocacy group, paid or unpaid, as former joint secretary, executive council member, Indian Meteorological Society, Jaipur Chapter (India), and member secretary-DSTPURSE Program, outside the submitted work. Siddhesh Zadey reports leadership or fiduciary role in other board, society, committee, or advocacy group, paid or unpaid as ASAR co-founder and board member, Nivarana Board, G4 Alliance PRC and Asia WG Chair, Lancet Standing Commission, Citizen-Centred Health System for India, and State Mental Health Drafting Committee, Maharashtra, India, outside the submitted work. All other authors declare no competing interests.
