# Supplementary material for: Global, regional, and national burden of road injuries 1990–2023: a systematic analysis for the Global Burden of Disease Study 2023
Source: Lancet Public Health. 2026 Jul 20;11(9):e589–603. doi: 10.1016/S2468-2667(26)00141-6 (PMC13487794; doi:10.1016/S2468-2667(26)00141-6)
Supplement: Supplementary file 1 [file mmc1.pdf]

THE LANCET
Public Health

Supplementary appendix 1

This appendix formed part of the original submission and has been peer reviewed. We post it as supplied by the authors.

Supplement to: GBD 2023 Road Injuries Collaborators. Global, regional, and national burden of road injuries 1990–2023: a systematic analysis for the Global Burden of Disease Study 2023. *Lancet Public Health* 2026; published online July 20. https://doi.org/10.1016/S2468-2667(26)00141-6.

Appendix

Supplement to: Global, regional, and national burden of road injuries: a systematic analysis from the Global Burden of Disease Study 2023

Table of contents

Appendix 1

Search terms used in Research in Context 3

Supplementary methods 4

ICD codes mapped to cause list 4

GATHER checklist 5

Supplementary results 7

Search terms used in Research in Context

Table S1. Search terms used in PubMed literature search to identify existing evidence on global burden of road injuries

No	Search terms	Results
1	"wounds and injuries" or “burns” or “crush injuries” or "transport injuries" or "traffic injuries" or "road injuries" or “fracture*” or “bone*” or “injur*” or “accident” or “traffic”	3,065,666
2	"vehicle*" or "motorcyclist" or "motor*" or "bicycle" or "pedestrian" or "pedal"	769,616
3	"Global Burden of Disease" or "estimate" or "burden”	938,414
4	“death" or "disabilit*" or "mortality" or "morbidity" or “inpatient” or “outpatient”	3,289,140
5	1 AND 2 AND 3 AND 4	1,690
6	(1 AND 2 AND 3 AND 4) Filters: English, from 2017-2026	813

Supplementary methods

ICD codes mapped to cause list

Table S2. International Classification of Disease (ICD) codes mapped to Global Burden of Disease 2023 cause list

Cause name	ICD-10	ICD-9
Road injuries	V01-V04.99, V06-V80.929, V82-V82.9, V87.2-V87.3	E800.3, E801.3, E802.3, E803.3, E804.3, E805.3, E806.3, E807.3, E810.0-E810.6, E811.0-E811.7, E812.0-E812.7, E813.0-E813.7, E814.0-E814.7, E815.0-E815.7, E816.0-E816.7, E817.0-E817.7, E818.0-E818.7, E819.0-E819.7, E820.0-E820.6, E821.0-E821.6, E822.0-E822.7, E823.0-E823.7, E824.0-E824.7, E825.0-E825.7, E826.0-E826.1, E826.3-E826.4, E827.0, E827.3-E827.4, E828.0, E828.4, E829.0-E829.4, V03, V07.8-V07.9, V13, V13.8, V13.9, V15.2, V15.3, V15.9, V19, V42, V42.8, V42.9-V43, V47-V47.1
Motor vehicle road injuries	V30-V79.9, V87.2-V87.3	E810.0-E810.1, E811.0-E811.1, E812.0-E812.1, E813.0-E813.1, E814.0-E814.1, E815.0-E815.1, E816.0-E816.1, E817.0-E817.1, E818.0-E818.1, E819.0-E819.1, E820.0-E820.1, E821.0-E821.1, E822.0-E822.1, E823.0-E823.1, E824.0-E824.1, E825.0-E825.1, V42, V42.8, V42.9-V43, V47-V47.1
Motorcyclist road injuries	V20-V29.9	E810.2-E810.3, E811.2-E811.3, E812.2-E812.3, E813.2-E813.3, E814.2-E814.3, E815.2-E815.3, E816.2-E816.3, E817.2-E817.3, E818.2-E818.3, E819.2-E819.3, E820.2-E820.3, E821.2-E821.3, E822.2-E822.3, E823.2-E823.3, E824.2-E824.3, E825.2-E825.3
Cyclist road injuries	V10-V19.9	E800.3, E801.3, E802.3, E803.3, E804.3, E805.3, E806.3, E807.3, E810.6, E811.6, E812.6, E813.6, E814.6, E815.6, E816.6, E817.6, E818.6, E819.6, E820.6, E821.6, E822.6, E823.6, E824.6, E825.6, E826.1, V13, V13.8, V13.9, V15.2, V15.3, V15.9, V19
Pedestrian road injuries	V01-V04.99, V06-V09.9	E811.7, E812.7, E813.7, E814.7, E815.7, E816.7, E817.7, E818.7, E819.7, E822.7, E823.7, E824.7, E825.7, E826.0, E827.0, E828.0, E829.0, V03, V07.8-V07.9
Other road injuries	V80-V80.929, V82-V82.9	E810.4-E810.5, E811.4-E811.5, E812.4-E812.5, E813.4-E813.5, E814.4-E814.5, E815.4-E815.5, E816.4-E816.5, E817.4-E817.5, E818.4-E818.5, E819.4-E819.5, E820.4-E820.5, E821.4-E821.5, E822.4-E822.5, E823.4-E823.5, E824.4-E824.5, E825.4-E825.5, E826.3-E826.4, E827.3-E827.4, E828.4, E829.4

GATHER checklist

Item #	Checklist item	Reporting location
**Objectives and funding**		
1	Define the indicator(s), populations (including age, sex, and geographic entities), and time period(s) for which estimates were made.	Main text – Methods, Overview
2	List the funding sources for the work.	Main text – Summary, Funding
**Data Inputs**		
For all data inputs from multiple sources that are synthesized as part of the study:		
3	Describe how the data were identified and how the data were accessed.	Main text – Methods
4	Specify the inclusion and exclusion criteria. Identify all ad-hoc exclusions.	Main text – Methods with reference to previous publications
5	Provide information on all included data sources and their main characteristics. For each data source used, report reference information or contact name/institution, population represented, data collection method, year(s) of data collection, sex and age range, diagnostic criteria or measurement method, and sample size, as relevant.	Main text – Methods with reference to previous publications<br>Appendix – Supplementary Table 2
6	Identify and describe any categories of input data that have potentially important biases (e.g., based on characteristics listed in item 5).	Main text – Methods and Limitations
For data inputs that contribute to the analysis but were not synthesized as part of the study:		
7	Describe and give sources for any other data inputs.	Information published in GBD 2023 Capstone Papers
For all data inputs:		
8	Provide all data inputs in a file format from which data can be efficiently extracted (e.g., a spreadsheet rather than a PDF), including all relevant meta-data listed in item 5. For any data inputs that cannot be shared because of ethical or legal reasons, such as third-party ownership, provide a contact name or the name of the institution that retains the right to the data.	Data inputs in a spreadsheet format are available on the GHDx at https://sources.healthdata.org/collection/sources-2023
**Data analysis**		
9	Provide a conceptual overview of the data analysis method. A diagram may be helpful.	Main text – Methods
10	Provide a detailed description of all steps of the analysis, including mathematical formulae. This description should cover, as relevant, data cleaning, data pre-processing, data adjustments and weighting of data sources, and mathematical or statistical model(s).	Main text – Methods with reference to previous publications
11	Describe how candidate models were evaluated and how the final model(s) were selected.	Main text – Methods with reference to previous publications
12	Provide the results of an evaluation of model performance, if done, as well as the results of any relevant sensitivity analysis.	Information published in GBD 2023 Capstone Papers
13	Describe methods for calculating uncertainty of the estimates. State which sources of uncertainty were, and were not, accounted for in the uncertainty analysis.	Main text – Methods
14	State how analytic or statistical source code used to generate estimates can be accessed.	Code available on request
**Results and Discussion**		
15	Provide published estimates in a file format from which data	Main text – Data sharing

	can be efficiently extracted.	
**16**	Report a quantitative measure of the uncertainty of the estimates (e.g. uncertainty intervals).	95% Uncertainty Intervals (UIs) given for all findings, including in the text, figures, and tables in the main text and Supplemental Appendix.
**17**	Interpret results in light of existing evidence. If updating a previous set of estimates, describe the reasons for changes in estimates.	Main text – Research in context and Discussion
**18**	Discuss limitations of the estimates. Include a discussion of any modelling assumptions or data limitations that affect interpretation of the estimates.	Main text – Discussion, Limitation section

Supplementary results

Table S3. Number of road injury incident cases (in thousands) and age-standardised road injury incidence rates (per 100 000) in 2023 and 1990, and the percentage change in both metrics from 1990 to 2023, globally and across the World Bank income groups and 21 GBD regions, stratified by sex

	Incidence in 2023		Incidence in 1990		Change in incidence from 1990 to 2023	
	Number of cases, in thousands (95% UI)	Age-standardised incidence rate, per 100,000 (95% UI)	Number of cases, in thousands (95% UI)	Age-standardised incidence rate, per 100,000 (95% UI)	Percentage change in number of cases, in % (95% UI)	Percentage change in age-standardised incidence rate, in % (95% UI)
**Global**						
Total	50,900	623.0	54,900	1,008.9	-7.4	-38.3
	(46,100 to 56,100)	(566.9 to 686.8)	(49,900 to 60,400)	(915.3 to 1,106.0)	(-10.7 to -3.9)	(-39.7 to -36.9)
Male	35,000	849.7	35,500	1,293.6	-1.4	-34.3
	(31,600 to 38,600)	(767.9 to 939.6)	(32,000 to 39,300)	(1,171.7 to 1,421.4)	(-4.7 to 2.1)	(-35.8 to -32.8)
Female	15,900	393.3	19,400	720.3	-18.3	-45.4
	(14,400 to 17,500)	(357.3 to 430.6)	(17,700 to 21,300)	(652.7 to 785.1)	(-21.4 to -14.9)	(-46.7 to -44.2)
**World Bank low income**						
Total	3,600	558.7	2,340	750.7	54.1	-25.6
	(3,380 to 3,820)	(525.8 to 595.3)	(2,160 to 2,510)	(704.4 to 801.5)	(46.9 to 59.5)	(-27.4 to -23.9)
Male	2,320	734.0	1,340	913.3	73.4	-19.6
	(2,160 to 2,470)	(691.9 to 781.1)	(1,240 to 1,430)	(859.7 to 974.5)	(66.6 to 79.2)	(-21.3 to -17.8)
Female	1,280	389.7	1,000.0	597.6	28.2	-34.8
	(1,200 to 1,360)	(368.3 to 416.2)	(919 to 1,080)	(553.2 to 640.1)	(22.5 to 33.2)	(-36.6 to -33.0)
**World Bank lower-middle income**						
Total	13,800	446.4	8,910	523.3	55.0	-14.7
	(12,600 to 15,200)	(405.3 to 491.1)	(8,150 to 9,690)	(480.5 to 569.8)	(48.6 to 61.3)	(-16.6 to -12.8)
Male	9,600.0	612.4	5,590	655.9	71.5	-6.6
	(8,710 to 10,600)	(555.9 to 675.4)	(5,150 to 6,110)	(602.3 to 716.2)	(65.5 to 77.6)	(-8.6 to -4.5)
Female	4,220.0	279.8	3,320	385.0	27.2	-27.3
	(3,840 to 4,610)	(255.5 to 305.7)	(3,000 to 3,640)	(351.1 to 418.7)	(21.2 to 33.7)	(-29.2 to -25.5)
**World Bank upper-middle income**						
Total	22,000	751.3	23,600	1,060.3	-6.8	-29.1
	(19,700 to 24,700)	(672.6 to 836.0)	(21,000 to 26,400)	(948.0 to 1,171.6)	(-11.5 to -2.2)	(-31.7 to -26.8)
Male	15,500.0	1,041.1	15,300	1,348.6	1.1	-22.8
	(13,800 to 17,400)	(926.7 to 1,165.8)	(13,600 to 17,200)	(1,201.9 to 1,494.3)	(-3.9 to 6.2)	(-25.3 to -20.5)
Female	6,540.0	451.8	8,320.0	766.0	-21.4	-41.0
	(5,850 to 7,310)	(405.7 to 497.7)	(7,400 to 9,240)	(678.7 to 846.4)	(-25.7 to -16.3)	(-43.4 to -39.0)
**World Bank high income**						
Total	11,400	858.1	20,000	1,681.6	-43.1	-49.0
	(10,400 to 12,500)	(781.9 to 947.1)	(18,200 to 21,900)	(1,538.4 to 1,841.0)	(-44.5 to -41.5)	(-50.1 to -47.8)
Male	7,550	1,110.6	13,200	2,197.6	-42.8	-49.5
	(6,880 to 8,310)	(1,012.5 to 1,225.5)	(12,000 to 14,500)	(2,003.1 to 2,412.1)	(-44.4 to -41.0)	(-50.8 to -48.2)
Female	3,830	593.6	6,790	1,161.1	-43.6	-48.9
	(3,460 to 4,220)	(533.9 to 657.5)	(6,230 to 7,410)	(1,060.7 to 1,266.0)	(-44.9 to -42.1)	(-50.2 to -47.5)
**Andean Latin America**						
Total	368	548.8	281	727.6	31.0	-24.6
	(348 to 390)	(518.4 to 580.7)	(265 to 296)	(688.5 to 761.0)	(25.7 to 36.1)	(-27.2 to -22.2)
Male	258	767.6	183	975.6	41.2	-21.3
	(241 to 274)	(719.0 to 815.9)	(173 to 191)	(924.4 to 1,023.3)	(35.1 to 47.7)	(-24.3 to -18.3)
Female	111	334.0	98.8	488.2	12.1	-31.6
	(104 to 118)	(313.5 to 354.5)	(91.9 to 104)	(459.6 to 513.8)	(7.0 to 17.1)	(-34.1 to -29.5)
**Australasia**						
Total	201	668.8	338	1,650.3	-40.6	-59.5
	(189 to 216)	(624.6 to 722.1)	(318 to 358)	(1,548.0 to 1,747.8)	(-43.6 to -37.6)	(-61.3 to -57.3)
Male	126.0	831.7	211	2,041.3	-40.6	-59.3
	(118 to 136)	(769.3 to 898.7)	(197 to 224)	(1,910.1 to 2,162.6)	(-44.1 to -37.2)	(-61.8 to -56.7)
Female	75.6	504.8	127	1,256.2	-40.5	-59.8
	(70.1 to 82.7)	(459.8 to 555.4)	(118 to 137)	(1,163.0 to 1,348.5)	(-43.9 to -36.7)	(-62.2 to -56.9)
**Caribbean**						
Total	274	571.3	276	743.5	-0.6	-23.2
	(257 to 294)	(536.3 to 610.4)	(262 to 291)	(707.4 to 783.9)	(-4.4 to 4.1)	(-25.9 to -20.1)
Male	191.0	794.2	183	1,014.0	4.3	-21.7
	(179 to 205)	(742.6 to 849.3)	(174 to 193)	(965.6 to 1,064.4)	(-0.3 to 10.1)	(-25.3 to -17.7)
Female	83.4	354.5	92.7	483.9	-10.1	-26.7
	(77.2 to 90.1)	(327.4 to 383.9)	(87.4 to 99.1)	(457.2 to 513.8)	(-13.7 to -6.8)	(-29.8 to -23.9)
**Central Asia**						
Total	559	553.9	557	771.4	0.3	-28.2
	(529 to 589)	(526.2 to 583.2)	(530 to 581)	(736.7 to 803.4)	(-2.8 to 3.6)	(-30.2 to -26.3)
Male	390	775.7	384	1,095.0	1.6	-29.2
	(368 to 411)	(733.9 to 816.5)	(365 to 402)	(1,043.9 to 1,143.1)	(-2.4 to 5.7)	(-31.7 to -26.6)

Female	169	334.5	173	460.8	-2.7	-27.4
	(160 to 179)	(317.0 to 356.4)	(163 to 183)	(436.5 to 484.9)	(-5.9 to 0.6)	(-29.5 to -25.4)
**Central Europe**						
Total	678	633.1	1,590	1,279.0	-57.2	-50.5
	(630 to 730)	(589.9 to 682.7)	(1,480 to 1,700)	(1,200.5 to 1,368.5)	(-58.3 to -56.0)	(-51.7 to -49.2)
Male	478	872.7	1,100	1,765.6	-56.7	-50.6
	(446 to 514)	(813.5 to 940.4)	(1,030 to 1,190)	(1,649.2 to 1,896.2)	(-57.9 to -55.5)	(-51.8 to -49.2)
Female	201	386.4	481	792.7	-58.3	-51.2
	(186 to 217)	(355.0 to 422.7)	(451 to 516)	(739.9 to 848.4)	(-59.5 to -56.7)	(-53.0 to -49.5)
**Central Latin America**						
Total	1,900	713.0	1,770	1,059.5	7.1	-32.7
	(1,750 to 2,080)	(657.3 to 777.4)	(1,630 to 1,940)	(977.5 to 1,149.4)	(2.3 to 12.0)	(-34.5 to -31.0)
Male	1,410	1,076.3	1,260	1,557.3	12.3	-30.9
	(1,290 to 1,560)	(983.2 to 1,182.9)	(1,160 to 1,370)	(1,433.1 to 1,690.8)	(7.5 to 16.9)	(-32.9 to -29.1)
Female	487	370.4	516	588.0	-5.6	-37.0
	(449 to 531)	(339.5 to 402.7)	(464 to 567)	(535.8 to 639.3)	(-10.3 to -0.8)	(-38.8 to -35.3)
**Central sub-Saharan Africa**						
Total	1,040	734.1	549	896.4	90.1	-18.1
	(982 to 1,120)	(692.4 to 782.7)	(507 to 587)	(839.5 to 953.5)	(81.5 to 98.1)	(-20.8 to -15.6)
Male	644	925.0	283	994.2	127.4	-7.0
	(604 to 691)	(873.7 to 987.8)	(263 to 301)	(932.2 to 1,058.7)	(115.9 to 138.4)	(-10.7 to -2.7)
Female	400	546.9	266	806.2	50.4	-32.2
	(372 to 430)	(509.7 to 590.4)	(242 to 290)	(742.9 to 864.2)	(42.5 to 58.8)	(-34.9 to -29.4)
**East Asia**						
Total	12,200	775.5	11,900	949.3	1.7	-18.3
	(10,600 to 13,900)	(680.5 to 876.8)	(10,400 to 13,600)	(828.0 to 1,066.0)	(-4.5 to 9.8)	(-21.6 to -14.9)
Male	8,360	1,044.0	7,590	1,153.6	10.1	-9.5
	(7,320 to 9,600)	(910.9 to 1,186.2)	(6,590 to 8,730)	(1,005.4 to 1,302.8)	(3.5 to 18.4)	(-12.8 to -5.8)
Female	3,800	485.9	4,350	731.7	-12.8	-33.6
	(3,320 to 4,350)	(428.8 to 544.3)	(3,820 to 4,910)	(634.4 to 816.4)	(-19.1 to -5.4)	(-36.4 to -30.8)
**Eastern Europe**						
Total	1,830	962.0	3,760	1,677.1	-51.4	-42.6
	(1,590 to 2,100)	(831.6 to 1,106.2)	(3,290 to 4,280)	(1,473.8 to 1,900.5)	(-53.2 to -49.7)	(-44.8 to -40.7)
Male	1,260	1,338.0	2,610	2,347.2	-51.5	-43.0
	(1,090 to 1,460)	(1,150.7 to 1,550.7)	(2,260 to 3,000)	(2,051.1 to 2,694.0)	(-53.4 to -49.7)	(-45.2 to -40.9)
Female	564	592.2	1,150	1,029.0	-50.9	-42.4
	(497 to 636)	(520.9 to 667.6)	(1,010 to 1,280)	(908.5 to 1,150.6)	(-52.9 to -48.8)	(-44.9 to -40.3)
**Eastern sub-Saharan Africa**						
Total	1,210	303.9	819	430.1	48.2	-29.3
	(1,140 to 1,300)	(284.6 to 325.3)	(747 to 889)	(398.3 to 463.5)	(41.3 to 53.5)	(-31.0 to -27.7)
Male	805	412.3	476	529.2	68.9	-22.1
	(754 to 864)	(386.1 to 441.5)	(438 to 516)	(490.3 to 570.0)	(61.9 to 74.7)	(-24.0 to -20.3)
Female	409	201.2	343	335.4	19.4	-40.0
	(377 to 441)	(187.8 to 214.7)	(310 to 374)	(309.8 to 361.2)	(14.1 to 23.9)	(-41.6 to -38.1)
**High-income Asia Pacific**						
Total	1,020	561.7	2,940	1,700.3	-65.2	-67.0
	(942 to 1,110)	(512.0 to 617.3)	(2,710 to 3,200)	(1,566.5 to 1,837.3)	(-66.7 to -63.8)	(-68.2 to -65.7)
Male	655	722.6	1,980	2,259.4	-66.9	-68.0
	(605 to 719)	(657.9 to 795.2)	(1,820 to 2,170)	(2,076.0 to 2,462.4)	(-68.6 to -65.2)	(-69.2 to -66.7)
Female	366	394.0	956	1,133.5	-61.7	-65.2
	(336 to 400)	(351.5 to 436.9)	(884 to 1,030)	(1,046.1 to 1,224.5)	(-63.7 to -60.0)	(-67.2 to -63.2)
**High-income North America**						
Total	4,200.0	1,168.5	5,580	1,986.3	-24.7	-41.2
	(3,700 to 4,760)	(1,021.1 to 1,329.2)	(4,800 to 6,460)	(1,728.4 to 2,266.9)	(-27.7 to -21.4)	(-42.7 to -39.5)
Male	2,610.0	1,432.9	3,440	2,428.1	-23.9	-41.0
	(2,300 to 2,970)	(1,245.5 to 1,636.3)	(2,950 to 4,020)	(2,100.2 to 2,805.3)	(-27.3 to -20.0)	(-42.9 to -39.0)
Female	1,590.0	900.2	2,140	1,543.4	-26.0	-41.7
	(1,400 to 1,790)	(794.1 to 1,012.3)	(1,860 to 2,420)	(1,350.5 to 1,735.3)	(-28.5 to -23.5)	(-43.4 to -40.0)
**North Africa and Middle East**						
Total	6,960	1,076.3	6,570	1,787.9	5.9	-39.8
	(6,540 to 7,440)	(1,013.3 to 1,149.6)	(6,090 to 7,060)	(1,670.5 to 1,901.5)	(0.1 to 11.4)	(-41.9 to -37.9)
Male	4,820	1,419.9	3,970	2,188.8	21.2	-35.1
	(4,520 to 5,150)	(1,335.1 to 1,515.0)	(3,710 to 4,250)	(2,055.4 to 2,329.6)	(15.3 to 26.5)	(-37.2 to -33.3)
Female	2,140	700.4	2,600	1,366.9	-17.5	-48.8
	(2,010 to 2,300)	(656.9 to 748.8)	(2,370 to 2,820)	(1,261.8 to 1,465.9)	(-23.2 to -12.5)	(-51.0 to -46.8)
**Oceania**						
Total	86.4	587.0	42.9	659.0	101.4	-10.9
	(80.4 to 91.9)	(549.7 to 620.8)	(40.3 to 45.8)	(620.6 to 702.8)	(96.1 to 108.8)	(-13.2 to -8.1)
Male	60.6	794.6	29.7	875.3	104.3	-9.2
	(56.5 to 64.3)	(744.9 to 841.8)	(27.6 to 31.7)	(823.4 to 933.9)	(96.1 to 113.6)	(-12.6 to -5.3)
Female	25.8	366.8	13.3	428.0	94.9	-14.3
	(24.1 to 27.9)	(344.0 to 394.7)	(12.3 to 14.3)	(398.5 to 460.4)	(86.9 to 102.2)	(-17.6 to -11.3)
**South Asia**						

Total	7,540	389.9	4,110	381.8	83.2	2.1
	(6,650 to 8,600)	(343.2 to 440.5)	(3,590 to 4,660)	(334.6 to 430.4)	(73.8 to 92.0)	(-1.0 to 5.1)
Male	5,330	533.9	2,620	475.5	103.3	12.3
	(4,620 to 6,130)	(467.2 to 609.0)	(2,300 to 2,990)	(417.3 to 540.4)	(94.2 to 111.6)	(8.7 to 15.7)
Female	2,210	241.8	1,490	280.5	48.0	-13.8
	(1,950 to 2,480)	(214.7 to 270.4)	(1,290 to 1,680)	(244.5 to 312.8)	(38.8 to 57.9)	(-16.5 to -10.8)
**Southeast Asia**						
Total	3,500	475.0	4,020	820.2	-12.9	-42.1
	(3,230 to 3,810)	(438.8 to 514.9)	(3,700 to 4,370)	(758.1 to 887.5)	(-16.7 to -9.3)	(-43.7 to -40.6)
Male	2,550	686.8	2,700	1,112.6	-5.4	-38.3
	(2,350 to 2,780)	(633.7 to 748.9)	(2,480 to 2,940)	(1,023.4 to 1,206.3)	(-9.4 to -2.0)	(-40.1 to -36.5)
Female	948	260.9	1,320	538.4	-28.3	-51.5
	(870 to 1,030)	(239.8 to 282.6)	(1,200 to 1,450)	(492.8 to 583.8)	(-32.0 to -24.3)	(-53.2 to -50.1)
**Southern Latin America**						
Total	627	885.8	487	975.3	28.7	-9.2
	(594 to 667)	(837.4 to 945.2)	(462 to 512)	(925.2 to 1,023.6)	(24.0 to 34.9)	(-12.3 to -5.1)
Male	431	1,219.9	327	1,336.3	31.9	-8.7
	(407 to 460)	(1,144.5 to 1,302.5)	(310 to 343)	(1,267.1 to 1,399.7)	(25.5 to 41.0)	(-13.0 to -2.8)
Female	195	561.6	160	629.3	22.1	-10.8
	(185 to 208)	(530.1 to 599.4)	(151 to 171)	(595.5 to 669.6)	(16.6 to 27.6)	(-14.7 to -7.0)
**Southern sub-Saharan Africa**						
Total	840	901.1	696	1,356.3	20.8	-33.6
	(732 to 980)	(791.6 to 1,042.8)	(604 to 799)	(1,169.3 to 1,558.6)	(15.1 to 26.4)	(-36.0 to -31.2)
Male	589	1,286.8	415	1,725.2	41.7	-25.4
	(510 to 690)	(1,122.3 to 1,496.2)	(360 to 484)	(1,488.2 to 1,990.9)	(34.8 to 48.8)	(-28.4 to -22.7)
Female	252	537.4	280	1,018.7	-10.3	-47.3
	(220 to 290)	(477.6 to 615.1)	(242 to 321)	(878.1 to 1,172.0)	(-14.4 to -6.0)	(-49.2 to -45.0)
**Tropical Latin America**						
Total	1,860	833.4	1,850	1,182.5	0.6	-29.5
	(1,630 to 2,110)	(728.2 to 941.1)	(1,600 to 2,110)	(1,027.7 to 1,347.8)	(-4.6 to 6.4)	(-32.8 to -26.3)
Male	1,420	1,282.6	1,280	1,678.5	10.6	-23.6
	(1,240 to 1,610)	(1,120.0 to 1,452.9)	(1,110 to 1,490)	(1,459.4 to 1,929.1)	(5.3 to 16.7)	(-27.0 to -19.8)
Female	442	400.4	566	705.8	-22.0	-43.3
	(396 to 495)	(356.7 to 449.3)	(490 to 644)	(611.5 to 793.3)	(-26.9 to -17.1)	(-46.2 to -40.9)
**Western Europe**						
Total	2,440	609.9	5,980	1,583.0	-59.3	-61.5
	(2,290 to 2,610)	(565.2 to 658.8)	(5,650 to 6,360)	(1,493.8 to 1,683.9)	(-60.6 to -57.7)	(-63.0 to -59.9)
Male	1,610	798.9	4,010	2,100.9	-59.8	-62.0
	(1,520 to 1,720)	(746.9 to 857.3)	(3,760 to 4,250)	(1,973.6 to 2,231.8)	(-61.3 to -58.1)	(-63.5 to -60.3)
Female	825	417.8	1,970	1,059.4	-58.2	-60.6
	(761 to 898)	(375.3 to 464.2)	(1,860 to 2,110)	(995.3 to 1,131.1)	(-59.9 to -56.2)	(-62.7 to -58.2)
**Western sub-Saharan Africa**						
Total	1,580	322.1	766	370.8	106.4	-13.1
	(1,460 to 1,700)	(299.6 to 344.5)	(701 to 831)	(341.4 to 399.4)	(98.8 to 112.6)	(-15.1 to -11.5)
Male	995	426.0	438	442.4	127.5	-3.7
	(927 to 1,070)	(397.7 to 456.6)	(400 to 471)	(408.3 to 477.3)	(119.2 to 134.6)	(-5.8 to -1.9)
Female	586	227.2	329	299.8	78.3	-24.2
	(536 to 632)	(210.3 to 242.8)	(299 to 358)	(275.6 to 322.2)	(72.5 to 84.2)	(-25.8 to -22.9)

95% UI=95% uncertainty interval

Table S4. Number of road injury incident cases in 2023, age-standardised road injury incidence rates (per 100 000) in 2023, and the percentage change in age-standardised road injury incidence rates from 1990 to 2023, globally and across GBD regions and 204 countries and territories

	Number of incident cases in 2023 (95% UI)	Age-standardised incidence rate in 2023, per 100 000 (95% UI)	Percentage change in age-standardised incidence rate from 1990 to 2023, in % (95% UI)
**Global**	50,900,000 (46,100,000 to 56,100,000)	623.0 (566.9 to 686.8)	-38.3 (-39.7 to -36.9)
**World Bank low income**	3,600,000 (3,380,000 to 3,820,000)	558.7 (525.8 to 595.3)	-25.6 (-27.4 to -23.9)
**World Bank lower-middle income**	13,800,000 (12,600,000 to 15,200,000)	446.4 (405.3 to 491.1)	-14.7 (-16.6 to -12.8)
**World Bank upper-middle income**	22,000,000 (19,700,000 to 24,700,000)	751.3 (672.6 to 836.0)	-29.1 (-31.7 to -26.8)
**World Bank high income**	11,400,000 (10,400,000 to 12,500,000)	858.1 (781.9 to 947.1)	-49.0 (-50.1 to -47.8)
**Afghanistan**	315,000 (293,000 to 340,000)	868.3 (812.3 to 930.7)	-29.1 (-32.5 to -25.7)
**Albania**	15,400 (14,400 to 16,500)	708.0 (657.3 to 758.1)	-21.1 (-24.6 to -17.3)
**Algeria**	535,000 (503,000 to 566,000)	1,196.3 (1,128.2 to 1,267.8)	-51.1 (-52.9 to -49.1)
**American Samoa**	142 (132 to 151)	297.9 (277.7 to 317.6)	-21.0 (-24.4 to -17.7)
**Andorra**	812 (764 to 864)	946.4 (887.7 to 1,012.9)	-30.2 (-33.7 to -27.1)
**Angola**	253,000 (238,000 to 270,000)	713.5 (673.7 to 760.4)	-21.8 (-25.1 to -18.7)
**Antigua and Barbuda**	221 (207 to 236)	246.6 (229.3 to 265.3)	-40.5 (-43.1 to -37.9)
**Argentina**	448,000 (421,000 to 481,000)	931.8 (877.6 to 998.4)	-1.7 (-6.3 to 3.8)
**Armenia**	12,600 (11,900 to 13,400)	437.1 (410.6 to 464.7)	-32.4 (-35.7 to -29.6)
**Australia**	155,000 (146,000 to 166,000)	615.8 (574.5 to 661.4)	-59.6 (-62.4 to -56.5)
**Austria**	46,200 (43,400 to 49,300)	578.5 (532.8 to 628.2)	-67.2 (-69.8 to -64.7)
**Azerbaijan**	36,300 (33,600 to 38,800)	345.2 (320.6 to 368.9)	-43.9 (-46.6 to -41.2)
**Bahamas**	2,270 (2,130 to 2,430)	519.8 (487.4 to 554.9)	-33.0 (-36.2 to -29.7)
**Bahrain**	10,300 (9,530 to 11,000)	641.6 (600.4 to 684.0)	-42.4 (-44.8 to -40.0)
**Bangladesh**	300,000 (278,000 to 325,000)	169.4 (158.0 to 182.9)	-26.7 (-29.8 to -23.5)
**Barbados**	976 (918 to 1,040)	339.7 (315.8 to 364.5)	-29.1 (-32.3 to -25.5)
**Belarus**	58,800 (55,100 to 63,100)	684.5 (639.0 to 738.2)	-52.4 (-55.3 to -49.4)
**Belgium**	80,700 (75,900 to 85,700)	762.8 (710.6 to 813.0)	-60.7 (-63.1 to -57.6)
**Belize**	2,930 (2,760 to 3,100)	676.3 (638.9 to 717.4)	-16.3 (-19.8 to -12.5)
**Benin**	48,100 (44,900 to 51,000)	358.2 (338.6 to 377.3)	-18.7 (-21.4 to -16.0)
**Bermuda**	182 (172 to 194)	317.4 (298.0 to 341.6)	-51.4 (-54.1 to -48.5)
**Bhutan**	1,770 (1,650 to 1,910)	229.3 (215.5 to 245.0)	-28.9 (-32.1 to -25.9)
**Bolivia**	61,800 (58,300 to 66,000)	517.8 (489.5 to 551.6)	-42.4 (-44.7 to -40.3)
**Bosnia and Herzegovina**	12,200 (11,400 to 13,000)	423.5 (393.1 to 459.5)	-21.6 (-25.4 to -17.6)
**Botswana**	16,300 (15,200 to 17,600)	661.8 (618.8 to 708.3)	6.6 (2.3 to 11.6)
**Brazil**	1,810,000 (1,580,000 to 2,060,000)	834.8 (726.8 to 945.3)	-30.5 (-33.6 to -27.3)
**Brunei**	6,520 (6,110 to 6,950)	1,346.7 (1,265.2 to 1,432.7)	-47.9 (-50.4 to -45.6)
**Bulgaria**	44,600 (42,100 to 47,000)	774.7 (727.6 to 821.5)	-34.8 (-37.9 to -31.0)
**Burkina Faso**	114,000 (106,000 to 122,000)	482.2 (454.9 to 511.4)	-8.3 (-11.4 to -4.7)
**Burundi**	48,600 (45,400 to 51,600)	386.0 (364.3 to 407.3)	-28.1 (-30.4 to -25.4)
**Cabo Verde**	907 (849 to 971)	186.0 (174.2 to 198.9)	-14.7 (-18.4 to -11.3)
**Cambodia**	81,100 (76,300 to 86,600)	466.3 (439.7 to 496.7)	-34.4 (-37.3 to -31.4)
**Cameroon**	151,000 (140,000 to 161,000)	484.4 (455.6 to 513.5)	-5.5 (-9.2 to -2.0)
**Canada**	225,000 (211,000 to 239,000)	653.3 (607.4 to 700.9)	-58.5 (-61.1 to -55.5)
**Central African Republic**	65,000 (60,900 to 69,600)	984.7 (929.1 to 1,052.0)	7.4 (3.4 to 11.2)
**Chad**	72,500 (67,400 to 77,700)	379.0 (359.1 to 402.7)	4.3 (1.0 to 8.3)
**Chile**	147,000 (140,000 to 157,000)	756.7 (717.2 to 805.3)	-27.6 (-31.5 to -24.2)
**China**	11,700,000 (10,200,000 to 13,500,000)	773.3 (675.8 to 877.3)	-18.1 (-21.5 to -14.7)
**Colombia**	344,000 (321,000 to 367,000)	636.5 (595.6 to 678.8)	-29.5 (-33.5 to -25.7)
**Comoros**	3,160 (2,960 to 3,360)	388.1 (365.1 to 411.3)	-27.8 (-30.4 to -25.1)
**Congo**	35,600 (33,200 to 38,200)	608.5 (570.6 to 650.7)	-24.3 (-27.2 to -21.5)
**Cook Islands**	74.7 (69.6 to 79.6)	538.1 (496.1 to 580.0)	-42.2 (-44.9 to -38.8)
**Costa Rica**	37,100 (34,900 to 39,500)	681.4 (640.2 to 723.3)	-10.0 (-15.5 to -4.7)
**Croatia**	22,100 (20,900 to 23,300)	670.8 (629.4 to 718.0)	-53.9 (-56.5 to -50.9)
**Cuba**	34,700 (32,800 to 37,000)	320.9 (301.7 to 345.3)	-59.2 (-61.6 to -56.6)
**Cyprus**	12,500 (11,800 to 13,200)	965.7 (905.7 to 1,018.7)	-53.2 (-55.2 to -51.3)
**Czechia**	52,800 (49,800 to 55,700)	555.4 (514.7 to 593.7)	-52.1 (-54.9 to -48.9)
**Côte d'Ivoire**	108,000 (100,000 to 114,000)	344.8 (324.2 to 364.1)	-10.6 (-13.4 to -7.5)
**Democratic Republic of the Congo**	670,000 (630,000 to 719,000)	737.1 (692.2 to 787.8)	-18.2 (-21.7 to -14.7)
**Denmark**	28,600 (27,100 to 30,600)	540.3 (504.0 to 583.1)	-59.5 (-61.9 to -56.7)
**Djibouti**	4,100 (3,850 to 4,380)	318.6 (300.5 to 338.6)	-9.5 (-12.6 to -6.6)
**Dominica**	385 (359 to 411)	555.1 (516.3 to 591.7)	-15.4 (-19.2 to -11.6)
**Dominican Republic**	87,400 (81,000 to 93,800)	750.3 (697.2 to 804.8)	-1.1 (-6.1 to 4.7)
**Ecuador**	148,000 (139,000 to 157,000)	811.9 (763.7 to 862.7)	-2.7 (-8.2 to 1.8)
**Egypt**	1,530,000 (1,450,000 to 1,640,000)	1,433.0 (1,351.9 to 1,524.5)	-39.4 (-42.7 to -36.8)
**El Salvador**	42,300 (39,200 to 45,400)	661.4 (615.4 to 705.3)	-36.6 (-39.3 to -33.8)
**Equatorial Guinea**	8,650 (8,000 to 9,390)	543.4 (506.5 to 586.5)	-31.5 (-34.5 to -28.4)
**Eritrea**	25,600 (23,900 to 27,600)	405.5 (379.6 to 436.1)	-15.4 (-19.0 to -12.3)
**Estonia**	5,220 (4,900 to 5,550)	455.3 (420.0 to 489.8)	-73.0 (-75.0 to -71.0)
**Eswatini**	11,200 (10,300 to 12,100)	939.9 (878.1 to 1,012.6)	21.5 (16.6 to 27.2)
**Ethiopia**	223,000 (197,000 to 250,000)	225.4 (201.7 to 250.3)	-55.0 (-56.7 to -53.4)
**Fiji**	2,860 (2,670 to 3,050)	303.2 (282.9 to 323.0)	-23.0 (-25.6 to -20.0)
**Finland**	24,600 (23,100 to 26,300)	512.5 (470.9 to 554.1)	-58.8 (-61.3 to -55.8)

**France**	435,000 (410,000 to 466,000)	709.6 (661.2 to 762.5)	-62.5 (-65.0 to -59.6)
**Gabon**	11,700 (10,900 to 12,500)	626.9 (589.2 to 668.5)	-15.4 (-18.8 to -11.9)
**Gambia**	6,170 (5,790 to 6,620)	287.7 (270.3 to 308.0)	2.2 (-1.3 to 6.2)
**Georgia**	26,800 (25,500 to 28,200)	788.1 (744.1 to 832.5)	-2.8 (-7.9 to 2.1)
**Germany**	404,000 (380,000 to 430,000)	562.2 (522.2 to 605.7)	-62.7 (-65.3 to -59.9)
**Ghana**	130,000 (122,000 to 140,000)	404.8 (378.5 to 432.8)	-1.9 (-5.5 to 1.5)
**Greece**	108,000 (102,000 to 114,000)	1,132.5 (1,068.4 to 1,206.5)	-42.4 (-45.4 to -38.7)
**Greenland**	252 (235 to 274)	463.8 (425.4 to 503.9)	-48.9 (-51.4 to -46.3)
**Grenada**	340 (319 to 361)	300.7 (280.4 to 319.1)	-43.0 (-45.5 to -40.4)
**Guam**	551 (517 to 583)	355.3 (333.1 to 377.5)	-22.7 (-26.2 to -19.4)
**Guatemala**	108,000 (100,000 to 115,000)	640.1 (598.0 to 679.0)	14.3 (9.3 to 19.7)
**Guinea**	54,500 (51,000 to 57,900)	399.5 (377.8 to 424.7)	-7.6 (-11.5 to -3.7)
**Guinea-Bissau**	10,900 (10,100 to 11,700)	529.9 (495.7 to 563.9)	-28.2 (-30.9 to -25.4)
**Guyana**	5,140 (4,780 to 5,460)	600.0 (559.8 to 635.3)	-3.7 (-8.2 to 0.8)
**Haiti**	100,000 (93,300 to 108,000)	693.7 (647.3 to 755.2)	-19.3 (-22.1 to -16.5)
**Honduras**	62,600 (58,600 to 66,600)	609.9 (572.1 to 647.3)	-28.1 (-31.2 to -24.5)
**Hungary**	48,500 (45,900 to 51,200)	532.1 (496.7 to 563.0)	-60.0 (-62.4 to -57.5)
**Iceland**	1,510 (1,390 to 1,640)	427.8 (390.6 to 471.0)	-63.0 (-65.8 to -60.0)
**India**	6,340,000 (5,550,000 to 7,280,000)	422.0 (370.2 to 479.9)	2.3 (-1.4 to 5.8)
**Indonesia**	1,170,000 (1,020,000 to 1,320,000)	400.0 (353.8 to 448.7)	-54.1 (-56.9 to -51.6)
**Iran**	1,130,000 (987,000 to 1,290,000)	1,277.7 (1,121.5 to 1,441.5)	-53.9 (-56.7 to -51.5)
**Iraq**	272,000 (254,000 to 290,000)	615.8 (577.5 to 654.4)	-42.9 (-45.0 to -40.8)
**Ireland**	22,100 (20,400 to 23,900)	462.0 (421.5 to 506.9)	-65.8 (-68.4 to -62.8)
**Israel**	62,900 (59,000 to 66,800)	632.9 (591.5 to 674.1)	-45.8 (-48.6 to -42.5)
**Italy**	435,000 (393,000 to 484,000)	807.7 (715.7 to 899.7)	-54.9 (-57.2 to -52.1)
**Jamaica**	7,410 (6,940 to 7,880)	263.8 (246.8 to 282.6)	-0.6 (-4.3 to 3.5)
**Japan**	508,000 (454,000 to 573,000)	433.2 (379.5 to 488.9)	-63.8 (-64.9 to -62.6)
**Jordan**	79,000 (74,600 to 83,900)	595.0 (564.6 to 627.1)	-43.3 (-45.2 to -41.2)
**Kazakhstan**	129,000 (122,000 to 136,000)	650.5 (611.5 to 687.4)	-31.7 (-34.5 to -28.3)
**Kenya**	123,000 (109,000 to 137,000)	275.5 (248.0 to 307.8)	-4.1 (-6.7 to -1.5)
**Kiribati**	597 (549 to 649)	451.5 (417.3 to 487.3)	-4.4 (-8.8 to 0.3)
**Kuwait**	31,600 (29,900 to 33,500)	663.6 (629.1 to 700.3)	-45.6 (-48.4 to -42.6)
**Kyrgyzstan**	47,900 (45,700 to 50,600)	656.9 (624.9 to 694.4)	-31.2 (-34.1 to -27.4)
**Laos**	34,300 (32,100 to 36,800)	443.9 (417.9 to 474.8)	-43.1 (-45.5 to -40.4)
**Latvia**	10,400 (9,820 to 11,100)	641.2 (598.6 to 681.5)	-67.8 (-69.8 to -65.5)
**Lebanon**	26,600 (24,800 to 28,600)	464.9 (434.2 to 498.7)	-23.4 (-26.4 to -19.5)
**Lesotho**	19,200 (17,800 to 20,600)	1,009.3 (945.9 to 1,083.8)	55.5 (47.7 to 62.4)
**Liberia**	16,300 (15,300 to 17,500)	294.7 (277.0 to 314.0)	-22.0 (-25.5 to -18.5)
**Libya**	97,000 (89,900 to 104,000)	1,247.0 (1,167.2 to 1,331.9)	-30.7 (-33.7 to -27.8)
**Lithuania**	14,400 (13,600 to 15,200)	579.8 (542.6 to 617.6)	-67.0 (-68.9 to -64.9)
**Luxembourg**	3,900 (3,670 to 4,140)	647.0 (599.6 to 694.3)	-66.8 (-68.8 to -64.9)
**Madagascar**	78,700 (73,900 to 83,300)	285.9 (270.2 to 302.0)	-31.9 (-35.0 to -28.9)
**Malawi**	56,500 (52,900 to 60,300)	300.6 (282.1 to 317.6)	-22.2 (-25.9 to -18.8)
**Malaysia**	238,000 (222,000 to 253,000)	649.0 (609.1 to 690.6)	-16.6 (-20.9 to -11.9)
**Maldives**	1,020 (938 to 1,100)	177.2 (163.2 to 190.7)	-53.2 (-55.6 to -50.8)
**Mali**	64,500 (59,900 to 68,800)	263.8 (249.0 to 280.4)	-26.4 (-28.8 to -23.9)
**Malta**	2,270 (2,110 to 2,460)	485.6 (445.4 to 535.0)	-42.7 (-46.2 to -39.0)
**Marshall Islands**	217 (200 to 233)	526.5 (489.9 to 564.0)	-14.3 (-18.2 to -9.4)
**Mauritania**	22,200 (20,700 to 23,800)	520.7 (490.0 to 555.2)	-38.8 (-40.4 to -36.7)
**Mauritius**	4,460 (4,230 to 4,720)	339.8 (321.2 to 360.4)	-19.3 (-23.7 to -15.4)
**Mexico**	1,030,000 (898,000 to 1,170,000)	757.3 (664.8 to 860.4)	-36.1 (-38.4 to -34.0)
**Micronesia (Federated States of)**	680 (629 to 741)	642.7 (596.8 to 700.9)	-13.7 (-18.0 to -9.8)
**Monaco**	194 (181 to 207)	647.7 (596.5 to 700.3)	-34.4 (-37.9 to -30.5)
**Mongolia**	26,800 (24,800 to 28,800)	762.1 (705.5 to 817.3)	11.4 (6.6 to 16.8)
**Montenegro**	4,070 (3,830 to 4,320)	649.1 (604.2 to 691.4)	-22.8 (-26.5 to -19.3)
**Morocco**	374,000 (350,000 to 397,000)	987.3 (926.3 to 1,046.6)	-41.0 (-43.5 to -38.4)
**Mozambique**	119,000 (111,000 to 126,000)	420.5 (396.2 to 446.0)	-5.3 (-9.8 to -1.1)
**Myanmar**	238,000 (220,000 to 256,000)	401.1 (372.6 to 431.3)	-48.5 (-50.8 to -46.5)
**Namibia**	19,500 (18,100 to 20,900)	669.5 (627.3 to 715.3)	-4.9 (-8.0 to -1.7)
**Nauru**	109 (101 to 117)	850.4 (790.4 to 912.4)	-4.6 (-9.3 to 0.1)
**Nepal**	66,100 (61,400 to 71,200)	211.7 (197.3 to 227.5)	-29.2 (-32.3 to -26.0)
**Netherlands**	76,600 (71,800 to 82,000)	468.4 (431.3 to 508.0)	-57.6 (-60.6 to -54.8)
**New Zealand**	45,800 (40,300 to 52,100)	942.5 (822.3 to 1,068.2)	-58.2 (-60.7 to -55.9)
**Nicaragua**	31,300 (29,300 to 33,300)	458.6 (430.6 to 484.9)	-29.1 (-32.0 to -26.2)
**Niger**	71,600 (66,500 to 76,300)	274.8 (260.2 to 288.6)	-24.3 (-27.2 to -21.2)
**Nigeria**	590,000 (522,000 to 660,000)	255.6 (227.1 to 283.1)	-16.1 (-18.9 to -13.8)
**Niue**	7.02 (6.55 to 7.58)	432.7 (399.6 to 468.8)	-25.4 (-28.4 to -22.0)
**North Korea**	309,000 (289,000 to 332,000)	1,055.3 (982.1 to 1,133.6)	3.6 (-1.2 to 8.4)
**North Macedonia**	9,190 (8,640 to 9,810)	532.6 (497.7 to 574.3)	-26.7 (-30.2 to -23.4)
**Northern Mariana Islands**	230 (215 to 247)	474.6 (442.9 to 506.8)	-29.5 (-33.5 to -25.9)
**Norway**	22,000 (19,400 to 24,600)	458.6 (399.6 to 516.8)	-63.2 (-64.9 to -61.3)
**Oman**	94,000 (89,300 to 98,700)	1,823.1 (1,745.9 to 1,907.4)	-54.1 (-56.0 to -52.1)
**Pakistan**	830,000 (726,000 to 941,000)	346.3 (304.1 to 392.5)	17.1 (13.4 to 21.6)
**Palau**	116 (108 to 126)	626.6 (579.3 to 681.3)	-16.4 (-19.9 to -12.4)
**Palestine**	24,000 (22,500 to 25,800)	436.2 (410.6 to 465.0)	-34.3 (-36.7 to -31.6)
**Panama**	22,300 (20,900 to 23,900)	526.5 (494.1 to 564.3)	-40.7 (-44.0 to -37.3)

**Papua New Guinea**	68,100 (63,200 to 72,400)	617.3 (577.2 to 654.4)	-15.2 (-17.9 to -11.6)
**Paraguay**	52,200 (48,400 to 56,000)	789.2 (735.3 to 845.5)	59.1 (50.4 to 67.9)
**Peru**	159,000 (150,000 to 169,000)	431.7 (406.9 to 458.8)	-31.0 (-33.8 to -28.0)
**Philippines**	397,000 (345,000 to 459,000)	337.6 (296.7 to 387.2)	-25.9 (-27.8 to -24.0)
**Poland**	242,000 (212,000 to 276,000)	653.3 (564.2 to 745.8)	-54.7 (-56.0 to -53.6)
**Portugal**	73,400 (69,600 to 77,700)	742.8 (698.6 to 798.1)	-71.7 (-73.4 to -69.7)
**Puerto Rico**	11,600 (10,900 to 12,200)	390.0 (364.0 to 416.6)	-44.5 (-47.2 to -41.1)
**Qatar**	30,100 (28,100 to 32,100)	940.6 (890.4 to 996.2)	-46.1 (-48.5 to -43.8)
**Republic of Moldova**	20,400 (19,200 to 21,600)	649.0 (606.5 to 687.5)	-59.3 (-61.8 to -56.9)
**Romania**	129,000 (122,000 to 136,000)	720.3 (677.8 to 762.7)	-44.8 (-48.0 to -41.6)
**Russia**	1,370,000 (1,180,000 to 1,580,000)	987.5 (845.6 to 1,144.3)	-43.8 (-45.9 to -41.8)
**Rwanda**	52,400 (49,100 to 55,800)	415.9 (392.2 to 439.5)	-42.4 (-44.4 to -40.2)
**Saint Kitts and Nevis**	156 (144 to 169)	317.3 (293.8 to 344.3)	-52.4 (-55.1 to -49.4)
**Saint Lucia**	844 (794 to 903)	468.3 (440.6 to 502.4)	-35.7 (-39.0 to -32.5)
**Saint Vincent and the Grenadines**	339 (319 to 362)	298.2 (279.9 to 319.7)	-32.9 (-35.5 to -29.8)
**Samoa**	985 (911 to 1,060)	491.6 (455.1 to 531.2)	-13.7 (-17.2 to -10.4)
**San Marino**	253 (239 to 269)	821.7 (770.6 to 883.8)	-37.0 (-40.0 to -33.8)
**São Tomé and Príncipe**	512 (479 to 547)	248.7 (235.1 to 263.3)	-8.9 (-13.3 to -4.8)
**Saudi Arabia**	797,000 (740,000 to 859,000)	2,100.3 (1,974.5 to 2,245.2)	5.2 (0.1 to 9.6)
**Senegal**	49,100 (45,900 to 52,500)	291.5 (274.7 to 310.5)	-15.7 (-19.0 to -12.2)
**Serbia**	49,100 (46,100 to 52,200)	564.3 (527.5 to 604.0)	-52.7 (-55.0 to -49.9)
**Seychelles**	469 (440 to 501)	429.3 (402.2 to 461.0)	4.7 (-0.4 to 9.7)
**Sierra Leone**	32,100 (29,800 to 34,100)	402.7 (377.8 to 425.5)	-11.3 (-14.8 to -7.8)
**Singapore**	22,300 (20,500 to 24,500)	457.3 (410.9 to 507.7)	-54.8 (-58.1 to -50.6)
**Slovakia**	28,900 (27,200 to 30,700)	579.9 (544.1 to 626.9)	-54.0 (-56.4 to -51.3)
**Slovenia**	9,800 (9,180 to 10,400)	564.8 (523.6 to 604.2)	-67.4 (-69.6 to -65.2)
**Solomon Islands**	5,670 (5,260 to 6,120)	673.6 (629.3 to 727.5)	-6.2 (-10.0 to -2.3)
**Somalia**	75,000 (70,300 to 80,300)	389.1 (368.2 to 416.2)	-18.5 (-21.2 to -16.0)
**South Africa**	699,000 (597,000 to 832,000)	995.5 (863.2 to 1,171.2)	-38.7 (-41.1 to -36.3)
**South Korea**	485,000 (458,000 to 516,000)	863.1 (813.2 to 919.1)	-72.0 (-73.7 to -70.4)
**South Sudan**	25,900 (24,200 to 27,600)	285.4 (268.6 to 303.1)	-23.5 (-26.3 to -20.2)
**Spain**	252,000 (239,000 to 269,000)	599.2 (561.2 to 644.2)	-68.8 (-70.8 to -66.7)
**Sri Lanka**	92,400 (87,000 to 99,000)	394.0 (370.4 to 423.2)	-29.1 (-32.2 to -25.9)
**Sudan**	333,000 (312,000 to 357,000)	915.8 (861.7 to 978.5)	-33.5 (-35.8 to -30.9)
**Suriname**	3,610 (3,390 to 3,860)	608.1 (570.0 to 649.6)	-16.8 (-20.6 to -12.5)
**Sweden**	40,300 (35,700 to 45,100)	421.4 (368.9 to 477.6)	-62.0 (-63.5 to -60.3)
**Switzerland**	36,400 (34,000 to 39,400)	470.6 (429.1 to 518.0)	-63.9 (-66.7 to -60.7)
**Syria**	98,700 (93,000 to 105,000)	557.8 (525.4 to 591.2)	-32.7 (-35.0 to -30.1)
**Taiwan**	132,000 (125,000 to 139,000)	543.2 (505.5 to 576.9)	-53.2 (-55.7 to -50.4)
**Tajikistan**	40,400 (37,500 to 43,600)	378.1 (352.5 to 403.6)	-37.4 (-39.9 to -34.8)
**Tanzania**	169,000 (158,000 to 180,000)	278.8 (263.0 to 293.0)	-18.3 (-21.4 to -15.4)
**Thailand**	498,000 (467,000 to 533,000)	799.9 (750.3 to 861.2)	-38.0 (-41.2 to -34.9)
**Timor-Leste**	5,490 (5,080 to 5,880)	383.8 (358.8 to 411.6)	-25.8 (-29.4 to -21.8)
**Togo**	38,900 (36,300 to 41,800)	463.3 (434.2 to 495.3)	-10.5 (-13.7 to -7.3)
**Tokelau**	5.67 (5.24 to 6.17)	347.0 (320.7 to 377.3)	-32.0 (-34.7 to -29.4)
**Tonga**	299 (276 to 321)	293.3 (273.2 to 314.3)	-8.8 (-12.0 to -5.5)
**Trinidad and Tobago**	6,180 (5,790 to 6,630)	460.4 (430.7 to 495.2)	-24.9 (-29.0 to -20.3)
**Tunisia**	109,000 (102,000 to 118,000)	896.7 (841.3 to 961.3)	-38.4 (-40.7 to -35.7)
**Turkmenistan**	17,800 (16,700 to 19,200)	308.7 (289.0 to 330.8)	-57.9 (-60.5 to -55.4)
**Tuvalu**	67.6 (62.7 to 72.8)	607.2 (566.1 to 651.2)	-26.8 (-29.8 to -22.8)
**Türkiye**	362,000 (341,000 to 386,000)	426.1 (402.0 to 451.4)	-44.6 (-47.4 to -41.9)
**Uganda**	152,000 (141,000 to 162,000)	381.8 (361.7 to 406.3)	-3.2 (-6.7 to 0.5)
**UK**	265,000 (236,000 to 294,000)	419.6 (371.9 to 470.2)	-57.7 (-58.8 to -56.4)
**Ukraine**	350,000 (304,000 to 405,000)	1,024.1 (879.6 to 1,178.5)	-30.5 (-33.9 to -27.2)
**United Arab Emirates**	175,000 (159,000 to 194,000)	1,448.6 (1,355.5 to 1,569.8)	-27.2 (-30.2 to -24.0)
**Uruguay**	31,400 (29,500 to 33,200)	927.6 (867.2 to 985.3)	-8.4 (-13.2 to -4.5)
**USA**	3,970,000 (3,480,000 to 4,530,000)	1,226.4 (1,063.2 to 1,403.0)	-39.6 (-41.5 to -37.6)
**Uzbekistan**	221,000 (208,000 to 234,000)	609.7 (575.7 to 644.5)	-13.2 (-17.1 to -8.8)
**Vanuatu**	2,170 (2,000 to 2,340)	664.7 (610.8 to 715.3)	-2.5 (-7.3 to 2.0)
**Venezuela**	226,000 (213,000 to 240,000)	887.4 (837.8 to 944.1)	-24.9 (-29.1 to -20.0)
**Viet Nam**	734,000 (688,000 to 791,000)	689.7 (647.6 to 743.5)	-5.6 (-10.1 to -1.2)
**Virgin Islands**	326 (306 to 345)	386.7 (362.2 to 413.2)	-41.0 (-43.8 to -38.7)
**Yemen**	527,000 (491,000 to 563,000)	1,508.2 (1,413.9 to 1,616.5)	-29.7 (-32.6 to -26.5)
**Zambia**	56,700 (53,000 to 60,400)	319.9 (302.6 to 339.2)	-24.1 (-27.7 to -20.9)
**Zimbabwe**	75,400 (70,200 to 81,700)	512.2 (476.9 to 554.3)	-17.8 (-21.2 to -13.8)

95% UI=95% uncertainty interval

Table S5. Number of incident cases and deaths (in thousands) for each Level 4 road injury cause, together with the percentage each cause contributes to the total number of road injury cases and deaths, for both sexes and all ages combined, globally and by World Bank income group, in 2023.

		Incidence		Mortality	
		Number of incident cases in 2023, in thousands (95% UI)	Percentage of all road injury incident cases in 2023 (95% UI)	Number of deaths in 2023, in thousands (95% UI)	Percentage of all road injury deaths in 2023 (95% UI)
**Global**	**Motor vehicles**	19,800 (17,500 to 22,600)	38.8	518 (417 to 648)	38.6
	**Motorcyclists**	6,700 (5,710 to 7,910)	13.2	319 (217 to 416)	23.8
	**Cyclists**	10,100 (8,400 to 12,100)	19.8	88.8 (61.4 to 127)	6.6
	**Pedestrians**	11,600 (10,000 to 13,400)	22.7	401 (295 to 532)	29.9
	**Others**	2,790 (2,120 to 3,450)	5.5	15.4 (10.0 to 22.6)	1.1
**Low-income**	**Motor vehicles**	1,780 (1,650 to 1,940)	49.5	116 (78.9 to 162)	46.9
	**Motorcyclists**	253 (215 to 297)	7.0	27.5 (15.8 to 41.7)	11.1
	**Cyclists**	431 (356 to 529)	12.0	8.93 (4.52 to 17.7)	3.6
	**Pedestrians**	824 (732 to 903)	22.9	92.3 (58.7 to 136)	37.3
	**Others**	309 (228 to 389)	8.6	2.89 (1.50 to 4.65)	1.2
**Lower-middle income**	**Motor vehicles**	5,340 (4,750 to 6,060)	38.7	185 (134 to 253)	33.4
	**Motorcyclists**	2,230 (1,890 to 2,650)	16.1	164 (91.7 to 228)	29.4
	**Cyclists**	2,510 (2,050 to 3,090)	18.2	34.4 (18.3 to 55.1)	6.2
	**Pedestrians**	2,840 (2,460 to 3,270)	20.6	166 (105 to 239)	29.8
	**Others**	892 (673 to 1,130)	6.5	6.42 (3.60 to 10.8)	1.2
**Upper-middle income**	**Motor vehicles**	6,710 (5,830 to 7,860)	30.4	147 (118 to 179)	35.3
	**Motorcyclists**	3,100 (2,640 to 3,680)	14.1	112 (85.5 to 135)	26.8
	**Cyclists**	4,730 (3,970 to 5,570)	21.5	39.3 (23.8 to 52.1)	9.4
	**Pedestrians**	6,270 (5,340 to 7,380)	28.4	114 (91.1 to 147)	27.2
	**Others**	1,220 (946 to 1,510)	5.6	5.20 (3.83 to 6.96)	1.2
**High-income**	**Motor vehicles**	5,900 (5,290 to 6,800)	51.9	69.1 (65.1 to 72.9)	57.0
	**Motorcyclists**	1,110 (948 to 1,330)	9.8	15.9 (14.5 to 17.4)	13.1
	**Cyclists**	2,380 (1,990 to 2,800)	20.9	6.08 (5.49 to 6.75)	5.0
	**Pedestrians**	1,620 (1,400 to 1,870)	14.2	29.2 (26.8 to 32.3)	24.1
	**Others**	364 (264 to 466)	3.2	0.84 (0.74 to 0.95)	0.7

95% UI=95% uncertainty interval

Table S6. Number of road injury deaths (in thousands) and age-standardised road injury mortality rates (per 100 000) in 2023 and 1990, and the percentage change in both metrics from 1990 to 2023, globally and across the World Bank income groups and 21 GBD regions, stratified by sex

	Deaths in 2023		Deaths in 1990		Change in mortality from 1990 to 2023	
	Number of deaths, in thousands (95% UI)	Age-standardised mortality rate, per 100 000 (95% UI)	Number of deaths, in thousands (95% UI)	Age-standardised mortality rate, per 100 000 (95% UI)	Percentage change in number of deaths, in % (95% UI)	Percentage change in age-standardised mortality rate, in % (95% UI)
**Global**						
Total	1,340	16.1	1,210	23.7	11.1	-32.3
	(1,040 to 1,580)	(12.5 to 19.0)	(962 to 1,440)	(19.0 to 28.4)	(-16.0 to 54.7)	(-49.0 to -6.1)
Male	991	23.9	881	35.0	12.5	-31.9
	(718 to 1,200)	(17.3 to 29.0)	(643 to 1,130)	(25.6 to 45.3)	(-18.0 to 68.2)	(-50.7 to 2.4)
Female	352	8.4	328	12.8	7.2	-34.6
	(216 to 445)	(5.1 to 10.7)	(230 to 439)	(9.1 to 17.1)	(-25.6 to 67.5)	(-54.4 to 2.3)
**World Bank low income**						
Total	248	43.8	95.9	37.7	158.2	16.2
	(179 to 318)	(31.7 to 56.0)	(63.9 to 135)	(25.2 to 52.4)	(75.1 to 311.1)	(-21.9 to 85.7)
Male	170	62.1	64.3	53.0	165.1	17.2
	(121 to 224)	(43.4 to 82.5)	(34.6 to 101)	(29.0 to 81.9)	(57.6 to 439.7)	(-30.2 to 138.5)
Female	77.2	26.7	31.6	23.4	144.2	13.8
	(44.3 to 120)	(15.2 to 41.0)	(15.9 to 56.4)	(11.9 to 41.2)	(38.7 to 412.2)	(-34.8 to 130.2)
**World Bank lower-middle income**						
Total	556	19.1	278	19.3	100.3	-0.7
	(386 to 703)	(13.2 to 24.3)	(204 to 360)	(14.2 to 25.0)	(45.4 to 192.5)	(-29.2 to 44.1)
Male	418	28.7	195	26.8	114.3	7.4
	(262 to 544)	(17.8 to 37.5)	(130 to 265)	(18.0 to 36.1)	(45.5 to 238.8)	(-28.7 to 68.1)
Female	138	9.7	82.6	11.5	67.5	-15.6
	(71.1 to 189)	(4.9 to 13.3)	(50.2 to 116)	(7.1 to 15.9)	(1.4 to 188.4)	(-48.1 to 44.5)
**World Bank upper-middle income**						
Total	417	13.2	591	29.0	-29.4	-54.5
	(341 to 484)	(10.9 to 15.3)	(412 to 766)	(20.0 to 38.1)	(-48.2 to 14.0)	(-66.6 to -26.0)
Male	312	19.8	442	43.4	-29.4	-54.5
	(246 to 369)	(15.6 to 23.2)	(277 to 606)	(26.9 to 60.3)	(-49.6 to 25.1)	(-67.7 to -19.1)
Female	105	6.6	150	14.9	-29.4	-55.4
	(73.5 to 131)	(4.6 to 8.2)	(90.2 to 222)	(9.0 to 22.1)	(-53.0 to 28.4)	(-70.4 to -19.9)
**World Bank high income**						
Total	121	7.5	243	19.1	-50.2	-61.1
	(115 to 129)	(7.1 to 7.9)	(235 to 252)	(18.4 to 19.8)	(-52.3 to -46.9)	(-62.7 to -58.5)
Male	90.8	11.4	179	29.2	-49.4	-61.2
	(85.7 to 96.3)	(10.7 to 12.0)	(171 to 188)	(27.9 to 30.6)	(-52.0 to -45.5)	(-63.3 to -58.3)
Female	30.4	3.5	63.9	9.5	-52.5	-63.0
	(27.9 to 32.9)	(3.3 to 3.8)	(60.6 to 67.4)	(9.0 to 10.1)	(-56.4 to -48.3)	(-65.7 to -59.8)
**Andean Latin America**						
Total	14.2	21.3	7.12	21.9	100.0	-2.6
	(12.5 to 17.0)	(18.6 to 25.4)	(6.00 to 8.31)	(18.5 to 25.7)	(70.8 to 158.5)	(-16.7 to 25.3)
Male	10.8	32.9	5.29	33.5	105.1	-1.6
	(9.12 to 13.8)	(27.7 to 42.1)	(4.18 to 6.56)	(26.7 to 41.8)	(71.1 to 193.6)	(-17.4 to 41.0)
Female	3.40	10.2	1.83	10.8	85.4	-5.7
	(2.67 to 4.44)	(8.0 to 13.3)	(1.42 to 2.31)	(8.4 to 13.6)	(47.6 to 176.9)	(-25.0 to 37.9)
**Australasia**						
Total	1.79	4.9	3.50	16.5	-49.0	-70.5
	(1.63 to 1.94)	(4.5 to 5.3)	(3.38 to 3.63)	(15.9 to 17.1)	(-53.7 to -44.6)	(-73.1 to -68.0)
Male	1.31	7.3	2.44	23.5	-46.2	-68.9
	(1.17 to 1.44)	(6.5 to 8.0)	(2.35 to 2.54)	(22.6 to 24.4)	(-51.9 to -40.7)	(-72.1 to -65.7)
Female	0.47	2.5	1.06	9.8	-55.5	-74.4
	(0.41 to 0.53)	(2.2 to 2.8)	(1 to 1.13)	(9.1 to 10.4)	(-61.2 to -49.6)	(-77.6 to -71.1)
**Caribbean**						
Total	11.5	23.0	8.69	25.0	32.4	-7.9
	(8.95 to 14.5)	(17.8 to 29.3)	(7.12 to 10.4)	(20.7 to 29.6)	(10.7 to 56.8)	(-23.4 to 9.5)
Male	8.86	35.9	6.41	38.1	38.3	-5.8
	(6.74 to 11.5)	(27.2 to 46.8)	(5.17 to 8.09)	(31.2 to 47.8)	(13.3 to 69.5)	(-22.7 to 15.8)
Female	2.66	10.8	2.29	12.6	16.1	-14.2
	(1.59 to 3.99)	(6.3 to 16.4)	(1.52 to 3.25)	(8.5 to 17.7)	(-10.0 to 59.3)	(-35.0 to 19.7)
**Central Asia**						
Total	9.51	9.6	10.7	16.6	-11.1	-42.2
	(8.57 to 10.6)	(8.6 to 10.7)	(9.66 to 11.8)	(14.9 to 18.4)	(-21.4 to 1.9)	(-48.9 to -33.7)
Male	7.35	15.2	8.15	26.6	-9.9	-42.6
	(6.55 to 8.22)	(13.5 to 17.1)	(7.08 to 9.30)	(23.0 to 30.4)	(-22.1 to 6.8)	(-50.4 to -31.8)
Female	2.17	4.3	2.55	7.5	-15.1	-43.1
	(1.85 to 2.55)	(3.7 to 5.1)	(2.15 to 2.97)	(6.4 to 8.7)	(-30.9 to 8.1)	(-53.6 to -27.6)
**Central Europe**						
Total	8.16	5.8	24.9	18.7	-67.2	-69.1
	(7.62 to 8.87)	(5.4 to 6.3)	(23.5 to 26.3)	(17.6 to 19.8)	(-69.1 to -64.2)	(-70.8 to -66.4)
Male	6.22	9.2	19.2	29.9	-67.6	-69.3
	(5.72 to 6.84)	(8.5 to 10.1)	(17.8 to 20.5)	(27.7 to 32.0)	(-69.8 to -63.7)	(-71.4 to -65.8)
Female	1.95	2.5	5.75	8.2	-66.1	-69.6
	(1.75 to 2.16)	(2.3 to 2.8)	(5.33 to 6.14)	(7.6 to 8.8)	(-69.1 to -61.0)	(-72.3 to -65.3)
**Central Latin America**						
Total	40.3	14.9	35.9	26.3	12.2	-43.5
	(37.4 to 43.8)	(13.8 to 16.2)	(33.2 to 38.5)	(24.3 to 28.4)	(1.5 to 27.8)	(-48.8 to -35.5)
Male	32.0	24.5	27.8	42.0	15.1	-41.6

	(29.3 to 35.4)	(22.4 to 27.1)	(25.3 to 30.4)	(38.1 to 46.1)	(2.1 to 34.6)	(-48.3 to -31.7)
Female	8.25	6.0	8.05	11.6	2.4	-48.5
	(7.53 to 9.12)	(5.5 to 6.6)	(7.20 to 8.85)	(10.4 to 12.8)	(-10.9 to 20.1)	(-55.3 to -40.2)
**Central sub-Saharan Africa**						
Total	107	84.1	31.2	60.9	244.1	38.0
	(72.8 to 140)	(55.4 to 110.2)	(16.5 to 46.4)	(32.2 to 89.3)	(132.3 to 431.8)	(-6.3 to 113.2)
Male	69.2	111.4	20.3	82.8	240.3	34.6
	(44.3 to 94.7)	(70.7 to 156.0)	(10.2 to 33.8)	(41.2 to 138.1)	(109.3 to 501.8)	(-16.7 to 143.1)
Female	38.2	58.2	10.9	40.2	251.2	44.9
	(18.6 to 64.2)	(27.8 to 99.9)	(3.75 to 20.7)	(14.0 to 77.2)	(100.6 to 598.5)	(-15.9 to 182.3)
**East Asia**						
Total	173	9.2	383	32.2	-54.7	-71.4
	(122 to 213)	(6.8 to 11.2)	(232 to 533)	(19.3 to 45.4)	(-71.9 to -11.3)	(-81.6 to -43.1)
Male	123	13.0	284	46.8	-56.7	-72.2
	(80.2 to 165)	(8.7 to 17.1)	(141 to 419)	(22.8 to 69.9)	(-76.0 to 5.0)	(-84.2 to -31.9)
Female	50.0	5.3	98.2	17.2	-49.0	-69.4
	(31.6 to 64.7)	(3.4 to 6.8)	(50.1 to 160)	(8.7 to 28.2)	(-70.9 to 20.0)	(-82.4 to -27.9)
**Eastern Europe**						
Total	18.8	8.3	55.3	23.2	-66.0	-64.2
	(17.0 to 21.0)	(7.5 to 9.2)	(50.3 to 60.5)	(21.1 to 25.3)	(-70.9 to -60.0)	(-69.2 to -58.1)
Male	14.5	13.4	41.9	37.5	-65.3	-64.3
	(12.9 to 16.5)	(11.9 to 15.1)	(37.1 to 46.2)	(33.2 to 41.5)	(-71.0 to -58.6)	(-70.1 to -57.6)
Female	4.27	3.6	13.4	10.2	-68.2	-65.0
	(3.64 to 4.86)	(3.1 to 4.1)	(11.5 to 15.6)	(8.7 to 11.9)	(-74.8 to -59.9)	(-72.2 to -56.0)
**Eastern sub-Saharan Africa**						
Total	143	42.2	58.1	37.3	146.0	13.0
	(103 to 189)	(30.6 to 56.3)	(40.3 to 79.9)	(25.9 to 50.5)	(61.3 to 296.0)	(-25.9 to 84.6)
Male	105	63.9	39.8	53.3	164.9	19.8
	(72.2 to 149)	(43.5 to 90.1)	(21.5 to 61.3)	(29.4 to 81.7)	(55.6 to 424.0)	(-28.1 to 137.0)
Female	37.6	22.0	18.3	22.3	105.0	-1.5
	(21.4 to 56.4)	(12.6 to 32.9)	(10.1 to 28.4)	(12.5 to 33.8)	(6.1 to 330.7)	(-48.1 to 98.7)
**High-income Asia Pacific**						
Total	9.11	2.9	32.1	17.7	-71.6	-83.4
	(7.92 to 11.5)	(2.5 to 3.8)	(28.4 to 35.1)	(15.6 to 19.4)	(-75.5 to -60.2)	(-86.0 to -75.9)
Male	6.23	4.4	23.5	26.6	-73.5	-83.4
	(5.37 to 8.42)	(3.7 to 6.0)	(19.7 to 27.4)	(22.4 to 31.0)	(-77.8 to -57.6)	(-86.1 to -72.9)
Female	2.88	1.5	8.60	9.1	-66.5	-83.9
	(2.27 to 3.83)	(1.2 to 2.0)	(7.19 to 10.1)	(7.4 to 10.6)	(-74.6 to -50.1)	(-87.3 to -74.7)
**High-income North America**						
Total	50.7	12.0	54.0	18.2	-6.2	-34.3
	(47.5 to 54.0)	(11.2 to 12.7)	(52.5 to 55.5)	(17.7 to 18.7)	(-12.6 to 0.4)	(-38.9 to -29.1)
Male	36.4	17.3	37.2	25.9	-2.1	-33.0
	(33.7 to 38.9)	(15.9 to 18.7)	(36.0 to 38.2)	(25.1 to 26.6)	(-9.7 to 5.6)	(-38.2 to -27.5)
Female	14.2	6.6	16.8	11.0	-15.3	-39.4
	(12.8 to 15.7)	(6.0 to 7.3)	(15.7 to 18.0)	(10.4 to 11.6)	(-25.0 to -3.5)	(-46.1 to -31.5)
**North Africa and Middle East**						
Total	105	17.3	89.4	30.3	17.3	-42.9
	(80.1 to 129)	(13.3 to 21.5)	(56.3 to 124)	(19.0 to 41.8)	(-17.4 to 91.4)	(-59.9 to -4.0)
Male	80.8	25.9	64.6	44.0	25.1	-41.1
	(55.6 to 102)	(17.9 to 33.2)	(34.5 to 97.2)	(23.4 to 67.3)	(-17.4 to 139.6)	(-62.1 to 15.5)
Female	24.1	8.3	24.8	16.2	-2.9	-48.6
	(15.5 to 32.7)	(5.4 to 11.3)	(10.8 to 40.1)	(7.1 to 26.0)	(-44.1 to 123.3)	(-70.5 to 16.5)
**Oceania**						
Total	2.27	17.3	1.03	18.4	120.5	-5.8
	(1.62 to 3.05)	(12.1 to 22.9)	(0.73 to 1.38)	(13.0 to 24.4)	(50.2 to 240.9)	(-36.4 to 47.1)
Male	1.50	22.6	0.71	25.1	112.1	-9.9
	(1.01 to 2.23)	(15.6 to 32.3)	(0.41 to 1.08)	(15.2 to 37.2)	(29.8 to 266.2)	(-44.6 to 54.8)
Female	0.78	11.8	0.32	11.4	138.8	4.1
	(0.44 to 1.13)	(6.8 to 17.4)	(0.18 to 0.54)	(6.4 to 18.5)	(32.0 to 323.2)	(-42.3 to 84.1)
**South Asia**						
Total	295	16.1	144	15.6	105.4	3.0
	(185 to 383)	(10.1 to 20.9)	(111 to 183)	(12.1 to 20.0)	(36.4 to 195.9)	(-33.1 to 50.4)
Male	230	24.7	104	21.8	122.1	13.5
	(135 to 306)	(14.1 to 32.9)	(71.2 to 137)	(15.1 to 29.1)	(36.6 to 245.9)	(-31.9 to 77.8)
Female	64.6	7.4	39.9	9.0	61.9	-17.5
	(32.3 to 92.5)	(3.7 to 10.7)	(26.9 to 55.7)	(6.2 to 12.4)	(1.8 to 188.4)	(-48.8 to 43.3)
**Southeast Asia**						
Total	135	18.1	92.9	22.1	45.6	-18.0
	(102 to 169)	(13.6 to 22.5)	(64.8 to 118)	(15.5 to 27.9)	(7.9 to 122.2)	(-38.6 to 25.0)
Male	100	27.0	69.0	33.5	44.3	-19.5
	(73.4 to 129)	(20.0 to 35.2)	(42.0 to 94.3)	(20.6 to 47.1)	(1.2 to 160.0)	(-42.6 to 45.5)
Female	35.8	9.5	23.9	11.4	49.6	-16.6
	(21.0 to 48.0)	(5.5 to 12.8)	(12.9 to 35.6)	(6.3 to 16.7)	(-8.5 to 194.0)	(-48.5 to 60.4)
**Southern Latin America**						
Total	7.28	9.4	6.59	13.6	10.5	-31.0
	(6.82 to 7.76)	(8.8 to 10.0)	(6.19 to 7.03)	(12.8 to 14.5)	(3.2 to 18.8)	(-35.8 to -25.8)
Male	5.71	15.4	4.99	21.6	14.4	-28.9
	(5.31 to 6.12)	(14.3 to 16.4)	(4.61 to 5.41)	(19.9 to 23.4)	(5.5 to 23.3)	(-34.5 to -23.1)
Female	1.57	3.8	1.59	6.3	-1.7	-40.0
	(1.44 to 1.70)	(3.5 to 4.1)	(1.47 to 1.73)	(5.8 to 6.8)	(-9.6 to 8.7)	(-45.0 to -33.5)
**Southern sub-Saharan Africa**						
Total	30.6	34.2	18.6	40.4	64.1	-15.4
	(23.8 to 39.2)	(26.6 to 43.9)	(14.5 to 22.6)	(31.6 to 49.3)	(18.6 to 126.6)	(-38.5 to 16.2)
Male	21.5	50.0	13.1	60.4	64.5	-17.2
	(15.3 to 29.4)	(35.4 to 68.3)	(9.10 to 16.7)	(42.1 to 77.6)	(14.2 to 131.6)	(-42.8 to 17.5)
Female	9.08	19.8	5.56	22.8	63.2	-13.2

	(6.01 to 13.2)	(13.1 to 28.7)	(3.68 to 8.01)	(15.2 to 32.9)	(-2.4 to 178.7)	(-48.0 to 47.5)
**Tropical Latin America**						
Total	40.7	16.9	38.9	28.7	4.6	-41.2
	(39.6 to 42.0)	(16.4 to 17.4)	(35.4 to 42.7)	(26.0 to 31.7)	(-5.0 to 14.7)	(-46.9 to -35.3)
Male	33.5	28.8	30.1	45.7	11.2	-37.1
	(32.5 to 34.6)	(27.8 to 29.7)	(26.9 to 33.8)	(40.6 to 51.6)	(-1.3 to 24.6)	(-44.6 to -28.9)
Female	7.21	5.9	8.78	12.8	-17.9	-54.2
	(6.81 to 7.57)	(5.5 to 6.1)	(7.78 to 10.0)	(11.3 to 14.7)	(-27.8 to -6.7)	(-59.9 to -48.0)
**Western Europe**						
Total	19.1	3.4	66.6	15.7	-71.3	-78.5
	(17.4 to 20.9)	(3.1 to 3.7)	(64.2 to 68.8)	(15.2 to 16.2)	(-73.8 to -68.9)	(-80.2 to -76.7)
Male	14.5	5.4	48.8	24.1	-70.3	-77.4
	(13.0 to 16.0)	(4.9 to 6.0)	(47.0 to 50.8)	(23.2 to 25.1)	(-73.0 to -67.3)	(-79.4 to -75.3)
Female	4.57	1.4	17.8	7.7	-74.2	-82.2
	(3.89 to 5.24)	(1.2 to 1.6)	(16.6 to 18.8)	(7.2 to 8.1)	(-78.1 to -70.6)	(-84.7 to -79.8)
**Western sub-Saharan Africa**						
Total	120	29.8	47.3	28.0	154.6	6.2
	(80.1 to 162)	(19.8 to 39.6)	(29.8 to 67.3)	(18.1 to 39.6)	(68.1 to 297.6)	(-28.7 to 62.0)
Male	82.5	43.1	29.8	36.7	177.3	17.5
	(53.4 to 115)	(27.4 to 59.7)	(16.6 to 47.8)	(20.7 to 57.4)	(70.9 to 417.9)	(-26.0 to 114.3)
Female	37.8	17.6	17.5	19.4	115.9	-9.4
	(18.9 to 56.0)	(8.7 to 25.6)	(8.89 to 29.8)	(10.4 to 32.3)	(18.1 to 331.6)	(-49.7 to 74.6)

95% UI=95% uncertainty interval

Table S7. Age-standardised road injury mortality rates (per 100 000) in 2023, and percentage changes in age-standardised road injury mortality rates from 1990 to 2023 and from 2010 to 2023, globally and across GBD regions and 204 countries and territories

	Age-standardised mortality rate in 2023, per 100 000 (95% UI)	Percentage change in age-standardised mortality rate from 1990 to 2023, in % (95% UI)	Percentage change in age-standardised mortality rate from 2010 to 2023, in % (95% UI)
**Global**	16.1 (12.5 to 19.0)	-32.3 (-49.0 to -6.1)	-13.3 (-25.8 to 3.8)
**World Bank low income**	43.8 (31.7 to 56.0)	16.2 (-21.9 to 85.7)	23.9 (-12.1 to 75.9)
**World Bank lower-middle income**	19.1 (13.2 to 24.3)	-0.7 (-29.2 to 44.1)	-5.6 (-25.3 to 21.6)
**World Bank upper-middle income**	13.2 (10.9 to 15.3)	-54.5 (-66.6 to -26.0)	-34.8 (-42.4 to -22.4)
**World Bank high income**	7.5 (7.1 to 7.9)	-61.1 (-62.7 to -58.5)	-26.1 (-29.9 to -21.8)
**Afghanistan**	21.6 (14.1 to 32.3)	21.1 (-27.5 to 119.1)	4.3 (-30.3 to 54.5)
**Albania**	5.9 (4.0 to 8.3)	-52.1 (-65.8 to -25.3)	-28.5 (-45.0 to -4.6)
**Algeria**	17.4 (11.6 to 24.5)	-51.8 (-69.7 to -14.2)	-32.2 (-56.3 to 12.7)
**American Samoa**	8.7 (6.2 to 11.7)	-34.8 (-59.2 to 0.2)	-0.3 (-30.7 to 46.1)
**Andorra**	9.6 (7.6 to 12.0)	-32.9 (-56.2 to 24.6)	-0.3 (-25.1 to 30.2)
**Angola**	68.2 (43.1 to 93.0)	-0.8 (-32.8 to 59.9)	-8.6 (-33.5 to 27.3)
**Antigua and Barbuda**	4.0 (3.3 to 4.7)	-58.6 (-66.7 to -48.3)	-30.5 (-45.3 to -15.0)
**Argentina**	8.9 (8.0 to 9.9)	-31.8 (-40.3 to -23.5)	-37.1 (-44.5 to -28.7)
**Armenia**	9.4 (8.0 to 10.8)	-14.0 (-33.1 to 17.5)	21.6 (1.3 to 46.3)
**Australia**	4.4 (4.0 to 4.9)	-71.0 (-73.8 to -68.0)	-34.0 (-40.3 to -27.3)
**Austria**	3.8 (3.4 to 4.3)	-78.0 (-80.4 to -75.2)	-36.4 (-44.6 to -28.0)
**Azerbaijan**	6.1 (3.8 to 10.5)	-24.0 (-52.4 to 40.9)	-3.8 (-39.9 to 79.8)
**Bahamas**	16.3 (14.0 to 18.9)	-29.0 (-41.4 to -11.0)	9.0 (-7.6 to 29.6)
**Bahrain**	11.7 (8.3 to 18.9)	-47.3 (-65.9 to -0.0)	-24.4 (-49.8 to 26.1)
**Bangladesh**	6.9 (4.5 to 11.4)	-8.7 (-46.0 to 75.3)	2.2 (-32.7 to 49.9)
**Barbados**	7.3 (6.2 to 8.6)	-40.0 (-50.8 to -26.2)	-11.7 (-27.4 to 7.4)
**Belarus**	6.7 (5.9 to 7.5)	-64.4 (-70.5 to -55.3)	-53.7 (-60.4 to -45.6)
**Belgium**	4.1 (3.7 to 4.7)	-77.9 (-80.6 to -74.8)	-48.2 (-55.0 to -41.0)
**Belize**	19.9 (16.9 to 23.4)	-3.4 (-21.6 to 22.4)	4.9 (-14.0 to 26.9)
**Benin**	40.3 (26.5 to 54.4)	-17.0 (-47.3 to 53.2)	1.5 (-32.2 to 49.3)
**Bermuda**	10.4 (8.9 to 12.1)	-44.5 (-54.0 to -31.7)	9.1 (-7.5 to 27.0)
**Bhutan**	8.3 (3.8 to 13.4)	-22.7 (-56.5 to 26.6)	-27.1 (-52.8 to 19.8)
**Bolivia**	29.9 (21.0 to 39.7)	-14.1 (-42.7 to 50.9)	-12.4 (-40.3 to 31.9)
**Bosnia and Herzegovina**	3.2 (2.4 to 4.1)	-61.3 (-70.6 to -42.1)	-34.7 (-51.6 to -6.5)
**Botswana**	20.5 (14.1 to 29.0)	-52.9 (-70.6 to -21.7)	-8.4 (-38.4 to 45.8)
**Brazil**	16.7 (16.2 to 17.2)	-42.5 (-48.0 to -36.6)	-26.8 (-29.4 to -24.0)
**Brunei**	8.3 (6.1 to 11.3)	-66.5 (-77.5 to -38.2)	-34.9 (-53.3 to -12.2)
**Bulgaria**	5.8 (5.1 to 6.6)	-63.7 (-68.1 to -58.7)	-37.0 (-45.3 to -28.3)
**Burkina Faso**	27.1 (16.6 to 38.0)	-11.9 (-49.5 to 43.3)	7.9 (-31.8 to 61.0)
**Burundi**	61.2 (39.9 to 84.9)	7.7 (-35.3 to 85.2)	5.2 (-25.8 to 53.3)
**Cabo Verde**	7.6 (5.2 to 11.1)	0.6 (-38.2 to 57.0)	2.7 (-32.7 to 54.3)
**Cambodia**	19.4 (12.8 to 26.1)	-50.6 (-68.0 to -7.8)	-13.3 (-40.7 to 38.0)
**Cameroon**	43.4 (29.4 to 57.1)	6.7 (-36.2 to 68.9)	0.3 (-32.5 to 47.8)
**Canada**	4.9 (4.4 to 5.4)	-67.1 (-70.4 to -62.8)	-26.5 (-34.2 to -17.9)
**Central African Republic**	53.6 (26.7 to 79.3)	-27.0 (-50.9 to 11.7)	-8.7 (-35.4 to 30.2)
**Chad**	31.5 (19.1 to 42.9)	5.8 (-31.6 to 56.1)	-5.0 (-32.0 to 38.3)
**Chile**	10.0 (9.3 to 11.0)	-34.6 (-41.5 to -26.0)	-16.7 (-24.6 to -7.2)
**China**	9.0 (6.4 to 11.1)	-72.1 (-82.6 to -42.8)	-54.3 (-64.2 to -41.6)
**Colombia**	15.7 (14.9 to 16.6)	-32.8 (-38.1 to -26.8)	7.7 (-0.1 to 16.3)
**Comoros**	15.9 (10.6 to 23.5)	-9.3 (-44.0 to 55.0)	25.3 (-18.3 to 97.1)
**Congo**	44.1 (25.3 to 66.1)	-17.3 (-45.4 to 39.7)	8.5 (-27.0 to 52.2)
**Cook Islands**	25.0 (18.5 to 32.3)	-36.8 (-56.8 to 3.3)	-10.6 (-38.0 to 21.0)
**Costa Rica**	16.5 (15.0 to 18.1)	-20.3 (-28.6 to -11.3)	15.7 (3.6 to 28.6)
**Croatia**	5.8 (5.3 to 6.4)	-67.8 (-71.5 to -63.7)	-37.1 (-45.1 to -28.5)
**Cuba**	5.4 (4.7 to 6.2)	-74.2 (-77.7 to -70.4)	-31.9 (-40.5 to -21.8)
**Cyprus**	6.3 (4.9 to 8.1)	-65.0 (-77.4 to -37.1)	-38.5 (-55.9 to -14.9)
**Czechia**	4.7 (4.4 to 5.0)	-64.0 (-67.6 to -60.2)	-34.3 (-42.1 to -26.5)
**Côte d'Ivoire**	23.4 (15.6 to 32.0)	8.8 (-29.3 to 76.4)	20.8 (-16.9 to 78.1)
**Democratic Republic of the Congo**	96.3 (63.5 to 128.9)	63.2 (12.4 to 149.0)	42.6 (-0.5 to 112.4)
**Denmark**	2.0 (1.7 to 2.2)	-84.3 (-86.4 to -81.8)	-57.4 (-63.1 to -51.1)
**Djibouti**	27.2 (19.3 to 37.7)	-5.6 (-37.5 to 45.3)	8.5 (-21.9 to 62.6)
**Dominica**	12.1 (8.6 to 16.6)	-23.7 (-45.4 to 10.2)	-11.3 (-36.7 to 29.4)
**Dominican Republic**	33.7 (25.5 to 40.2)	13.2 (-15.3 to 43.9)	27.2 (3.6 to 59.2)
**Ecuador**	25.1 (23.2 to 27.0)	-21.6 (-27.8 to -15.3)	-20.2 (-27.4 to -13.4)
**Egypt**	14.4 (10.0 to 19.2)	2.8 (-31.4 to 60.2)	5.4 (-28.3 to 72.8)
**El Salvador**	21.4 (16.1 to 30.2)	-37.6 (-53.8 to 0.4)	-6.8 (-28.2 to 14.3)
**Equatorial Guinea**	61.0 (30.8 to 99.5)	8.6 (-41.7 to 194.2)	11.0 (-34.4 to 65.5)
**Eritrea**	44.4 (31.3 to 58.7)	3.3 (-36.5 to 76.0)	14.7 (-18.2 to 64.0)
**Estonia**	3.3 (2.9 to 3.8)	-86.0 (-88.3 to -83.4)	-55.6 (-62.6 to -49.3)
**Eswatini**	41.3 (27.8 to 56.6)	-57.5 (-73.7 to -31.1)	-34.6 (-54.0 to -6.1)
**Ethiopia**	40.6 (28.5 to 56.6)	13.5 (-31.3 to 115.7)	32.4 (-11.9 to 94.9)
**Fiji**	9.5 (6.9 to 12.9)	-17.7 (-43.8 to 22.6)	-7.4 (-36.8 to 34.1)
**Finland**	2.7 (2.4 to 3.1)	-79.0 (-82.0 to -75.7)	-38.2 (-47.9 to -26.9)
**France**	4.0 (3.5 to 4.5)	-78.5 (-81.1 to -75.5)	-39.8 (-46.9 to -32.9)
**Gabon**	30.6 (17.4 to 47.0)	-23.8 (-51.3 to 24.3)	0.1 (-32.4 to 40.8)
**Gambia**	21.1 (14.4 to 28.4)	8.7 (-30.3 to 71.7)	25.9 (-11.7 to 80.7)
**Georgia**	14.5 (12.4 to 16.8)	30.9 (-0.5 to 77.8)	-9.9 (-26.0 to 10.8)
**Germany**	2.6 (2.3 to 2.9)	-81.5 (-83.6 to -79.1)	-43.6 (-50.2 to -37.2)
**Ghana**	30.8 (20.7 to 43.4)	38.6 (-14.7 to 127.1)	48.5 (8.3 to 107.5)
**Greece**	6.6 (5.9 to 7.3)	-67.3 (-70.7 to -63.6)	-42.1 (-48.5 to -35.9)
**Greenland**	6.4 (3.7 to 10.4)	-52.9 (-78.9 to 11.1)	0.2 (-36.8 to 51.7)
**Grenada**	5.6 (4.6 to 6.7)	-58.4 (-66.8 to -45.3)	-33.1 (-47.4 to -17.7)
**Guam**	8.7 (7.4 to 10.1)	-54.2 (-63.1 to -42.1)	-18.0 (-32.5 to -2.6)

**Guatemala**	16.4 (13.8 to 19.2)	6.1 (-12.5 to 27.6)	5.0 (-15.3 to 27.8)
**Guinea**	29.0 (18.9 to 39.4)	-20.1 (-49.2 to 38.8)	5.8 (-25.6 to 54.6)
**Guinea-Bissau**	19.6 (13.4 to 26.5)	-20.3 (-51.4 to 42.4)	3.1 (-29.5 to 59.3)
**Guyana**	15.3 (12.6 to 19.9)	-14.0 (-34.2 to 24.1)	-9.8 (-27.5 to 20.1)
**Haiti**	41.0 (21.7 to 61.6)	-5.5 (-37.2 to 63.0)	24.2 (-12.4 to 85.2)
**Honduras**	22.4 (15.3 to 32.4)	14.2 (-20.1 to 67.4)	19.0 (-12.0 to 60.0)
**Hungary**	5.3 (4.8 to 6.0)	-75.3 (-78.2 to -72.1)	-31.7 (-39.2 to -22.6)
**Iceland**	2.6 (2.2 to 3.0)	-77.1 (-80.9 to -72.2)	-24.7 (-38.0 to -11.2)
**India**	17.1 (10.7 to 22.5)	-1.3 (-36.1 to 43.7)	-11.6 (-29.8 to 10.4)
**Indonesia**	16.9 (12.5 to 22.9)	14.4 (-27.2 to 100.2)	-8.4 (-36.1 to 32.3)
**Iran**	18.3 (13.6 to 23.9)	-63.0 (-75.5 to -27.8)	-40.7 (-58.8 to 2.7)
**Iraq**	18.7 (11.4 to 28.3)	-10.7 (-49.6 to 80.6)	-12.6 (-43.0 to 34.9)
**Ireland**	2.0 (1.7 to 2.3)	-84.7 (-86.7 to -82.3)	-55.9 (-61.2 to -49.5)
**Israel**	4.0 (3.5 to 4.5)	-65.2 (-69.5 to -59.9)	-31.8 (-39.5 to -22.1)
**Italy**	4.8 (4.2 to 5.3)	-71.1 (-74.4 to -66.8)	-35.7 (-43.3 to -25.7)
**Jamaica**	5.5 (4.6 to 6.9)	50.0 (17.6 to 96.2)	29.4 (4.8 to 62.5)
**Japan**	1.9 (1.7 to 2.2)	-83.2 (-85.5 to -80.9)	-49.1 (-53.0 to -44.2)
**Jordan**	11.6 (8.9 to 15.5)	-60.3 (-72.5 to -32.5)	-31.0 (-45.5 to -11.8)
**Kazakhstan**	12.7 (11.7 to 13.7)	-55.0 (-60.1 to -48.8)	-37.8 (-44.9 to -30.5)
**Kenya**	31.8 (20.9 to 45.7)	25.9 (-20.9 to 82.0)	8.9 (-24.6 to 51.5)
**Kiribati**	7.6 (4.9 to 11.2)	6.0 (-35.5 to 68.9)	-4.3 (-37.7 to 43.5)
**Kuwait**	10.0 (9.1 to 11.0)	-63.2 (-67.2 to -58.1)	-41.5 (-47.5 to -35.0)
**Kyrgyzstan**	12.0 (10.7 to 13.8)	-8.1 (-23.0 to 11.6)	-39.3 (-47.6 to -28.2)
**Laos**	23.9 (15.4 to 32.2)	16.1 (-27.2 to 152.7)	5.3 (-28.0 to 70.4)
**Latvia**	7.3 (6.6 to 8.1)	-68.6 (-73.8 to -62.7)	-28.6 (-39.3 to -17.7)
**Lebanon**	9.2 (6.7 to 11.8)	-13.1 (-46.9 to 101.5)	-4.5 (-33.6 to 52.0)
**Lesotho**	36.8 (24.7 to 48.1)	-33.9 (-60.7 to 5.1)	-10.3 (-39.1 to 32.3)
**Liberia**	29.6 (20.7 to 41.0)	10.3 (-28.3 to 81.8)	30.0 (-12.4 to 92.7)
**Libya**	17.0 (11.8 to 22.9)	-46.7 (-66.5 to -15.2)	-21.4 (-47.3 to 16.4)
**Lithuania**	5.7 (5.1 to 6.3)	-77.8 (-80.8 to -74.1)	-52.5 (-59.4 to -46.3)
**Luxembourg**	3.1 (2.7 to 3.5)	-84.5 (-86.4 to -81.8)	-43.4 (-50.5 to -33.9)
**Madagascar**	51.4 (37.4 to 66.2)	15.8 (-19.6 to 84.4)	39.9 (-1.6 to 101.1)
**Malawi**	52.0 (34.2 to 75.9)	7.0 (-35.1 to 75.2)	37.8 (-7.0 to 103.6)
**Malaysia**	17.3 (14.2 to 21.3)	-42.1 (-57.8 to -12.7)	-26.9 (-41.0 to -3.3)
**Maldives**	3.8 (2.6 to 5.3)	-28.8 (-57.6 to 88.6)	-22.4 (-45.9 to 24.1)
**Mali**	34.4 (22.4 to 47.1)	2.8 (-34.7 to 69.5)	-0.2 (-31.2 to 41.3)
**Malta**	1.6 (1.3 to 1.8)	-77.3 (-81.4 to -72.2)	-50.6 (-58.6 to -40.4)
**Marshall Islands**	18.4 (12.3 to 25.4)	-8.4 (-39.2 to 49.2)	2.5 (-25.3 to 46.3)
**Mauritania**	16.9 (9.8 to 26.5)	-13.1 (-45.9 to 57.7)	8.3 (-27.3 to 60.8)
**Mauritius**	12.0 (10.7 to 13.4)	-36.4 (-46.1 to -25.1)	-9.4 (-21.1 to 5.0)
**Mexico**	13.1 (11.7 to 14.7)	-53.2 (-60.2 to -44.7)	-29.8 (-38.7 to -18.5)
**Micronesia (Federated States of)**	9.7 (7.0 to 13.1)	-31.5 (-56.7 to 11.9)	-18.3 (-43.7 to 15.0)
**Monaco**	5.2 (3.7 to 6.9)	-51.0 (-71.9 to -12.0)	-11.9 (-39.7 to 25.3)
**Mongolia**	14.1 (9.7 to 17.5)	-31.5 (-63.5 to 12.7)	-39.2 (-58.6 to -10.2)
**Montenegro**	5.7 (4.2 to 7.4)	-48.9 (-65.5 to -22.8)	-32.4 (-52.8 to -5.7)
**Morocco**	25.2 (17.1 to 34.0)	-40.9 (-61.9 to 13.4)	-18.7 (-46.5 to 35.5)
**Mozambique**	20.0 (14.0 to 27.4)	-25.9 (-50.2 to 20.3)	-5.0 (-38.0 to 43.5)
**Myanmar**	19.6 (11.5 to 28.3)	-18.1 (-50.3 to 46.8)	-4.1 (-36.6 to 48.7)
**Namibia**	41.1 (30.4 to 55.3)	-43.4 (-62.0 to -11.4)	-9.2 (-35.3 to 33.8)
**Nauru**	68.1 (35.4 to 96.8)	-7.8 (-41.0 to 67.8)	-15.8 (-40.0 to 20.2)
**Nepal**	10.0 (6.9 to 13.7)	9.8 (-33.7 to 96.6)	15.7 (-21.7 to 73.0)
**Netherlands**	3.2 (3.0 to 3.6)	-68.2 (-71.2 to -64.4)	-20.2 (-27.8 to -10.2)
**New Zealand**	7.1 (6.6 to 7.9)	-68.1 (-70.9 to -64.6)	-18.1 (-25.6 to -7.5)
**Nicaragua**	16.5 (13.0 to 23.6)	-28.4 (-46.6 to 17.1)	0.1 (-21.8 to 26.8)
**Niger**	32.3 (20.4 to 45.7)	24.8 (-20.9 to 109.1)	8.8 (-25.4 to 65.7)
**Nigeria**	27.5 (15.6 to 39.9)	1.3 (-37.0 to 66.3)	-9.5 (-41.6 to 33.4)
**Niue**	10.9 (6.4 to 15.5)	-37.3 (-62.3 to -3.2)	-15.7 (-42.6 to 20.1)
**North Korea**	19.0 (13.8 to 24.9)	-17.3 (-44.5 to 37.0)	-1.1 (-29.1 to 43.3)
**North Macedonia**	4.5 (3.4 to 6.1)	-52.5 (-64.3 to -28.2)	-30.3 (-47.9 to -9.9)
**Northern Mariana Islands**	14.6 (10.8 to 19.2)	-47.3 (-67.4 to -16.9)	-9.2 (-35.5 to 29.8)
**Norway**	2.1 (1.8 to 2.4)	-77.8 (-81.5 to -73.1)	-52.1 (-59.6 to -40.8)
**Oman**	24.7 (17.1 to 38.0)	-64.3 (-76.2 to -37.5)	-55.7 (-69.2 to -25.5)
**Pakistan**	16.9 (9.0 to 23.9)	54.6 (0.5 to 133.5)	15.5 (-20.0 to 65.2)
**Palau**	15.7 (11.7 to 19.7)	-26.4 (-52.1 to 30.5)	4.1 (-32.0 to 60.1)
**Palestine**	7.0 (5.3 to 9.9)	-56.4 (-71.7 to -24.7)	-33.3 (-52.2 to -9.0)
**Panama**	10.0 (9.1 to 11.2)	-55.9 (-61.3 to -48.8)	-34.7 (-41.3 to -25.6)
**Papua New Guinea**	19.1 (13.4 to 25.6)	-6.5 (-37.0 to 47.1)	-3.8 (-31.7 to 37.3)
**Paraguay**	23.9 (18.9 to 29.1)	45.7 (24.0 to 71.4)	-7.4 (-19.9 to 12.1)
**Peru**	17.1 (13.6 to 23.7)	24.5 (-0.7 to 90.5)	10.2 (-11.9 to 36.8)
**Philippines**	11.2 (8.3 to 14.0)	11.5 (-19.0 to 60.3)	-3.4 (-30.4 to 34.2)
**Poland**	5.6 (4.9 to 6.3)	-75.3 (-78.6 to -70.8)	-48.2 (-54.4 to -41.0)
**Portugal**	5.6 (4.9 to 6.4)	-82.0 (-84.3 to -79.3)	-35.7 (-44.2 to -24.8)
**Puerto Rico**	8.3 (7.4 to 9.2)	-55.5 (-60.4 to -49.4)	-13.5 (-21.9 to -3.5)
**Qatar**	9.9 (7.3 to 14.4)	-71.7 (-80.7 to -44.3)	-58.6 (-71.8 to -33.2)
**Republic of Moldova**	6.6 (5.7 to 7.8)	-75.2 (-79.4 to -69.5)	-48.3 (-57.0 to -35.8)
**Romania**	8.1 (7.2 to 9.3)	-61.2 (-66.4 to -55.6)	-27.2 (-36.3 to -18.4)
**Russia**	8.4 (7.5 to 9.3)	-67.5 (-71.6 to -62.9)	-50.8 (-58.0 to -43.4)
**Rwanda**	76.4 (49.1 to 114.0)	-17.1 (-49.3 to 48.8)	21.9 (-15.2 to 83.0)
**Saint Kitts and Nevis**	8.3 (7.1 to 9.9)	-49.6 (-58.5 to -37.7)	-13.9 (-30.5 to 4.9)
**Saint Lucia**	9.2 (7.8 to 10.9)	-57.6 (-65.7 to -46.7)	-27.8 (-38.4 to -14.6)
**Saint Vincent and the Grenadines**	4.7 (3.9 to 5.4)	-53.6 (-63.3 to -41.4)	-38.2 (-50.7 to -25.0)
**Samoa**	11.7 (8.2 to 15.7)	-20.8 (-50.0 to 25.5)	-7.3 (-35.3 to 35.6)
**San Marino**	6.1 (4.6 to 8.1)	-55.4 (-72.1 to -22.7)	-12.6 (-36.6 to 12.7)
**São Tomé and Príncipe**	31.2 (18.8 to 51.5)	51.5 (-10.0 to 151.6)	-19.9 (-48.5 to 21.1)
**Saudi Arabia**	32.4 (23.6 to 40.5)	-45.7 (-62.2 to -14.3)	-38.2 (-51.8 to -19.3)
**Senegal**	21.7 (15.2 to 29.0)	-2.2 (-38.2 to 62.0)	16.7 (-20.4 to 68.8)

**Serbia**	5.3 (4.0 to 6.6)	-60.2 (-71.3 to -40.4)	-35.4 (-49.5 to -21.6)
**Seychelles**	10.3 (6.6 to 15.0)	-18.9 (-51.7 to 40.0)	-21.0 (-43.7 to 19.4)
**Sierra Leone**	35.5 (25.3 to 47.2)	52.9 (2.7 to 136.4)	37.6 (-7.1 to 92.5)
**Singapore**	1.9 (1.7 to 2.1)	-82.3 (-84.9 to -79.6)	-48.8 (-53.6 to -42.3)
**Slovakia**	5.5 (4.3 to 7.2)	-67.4 (-75.1 to -48.9)	-35.1 (-46.6 to -17.7)
**Slovenia**	4.1 (3.7 to 4.6)	-80.1 (-82.5 to -76.6)	-40.6 (-49.3 to -31.9)
**Solomon Islands**	16.7 (10.6 to 23.1)	-2.2 (-41.5 to 56.4)	13.2 (-26.0 to 66.0)
**Somalia**	17.1 (10.2 to 23.4)	0.0 (-40.9 to 67.2)	-15.2 (-42.2 to 31.5)
**South Africa**	36.7 (28.4 to 46.3)	-7.1 (-33.9 to 26.3)	-14.5 (-34.2 to 12.9)
**South Korea**	5.6 (4.4 to 8.6)	-85.2 (-88.9 to -70.7)	-51.0 (-61.2 to -34.8)
**South Sudan**	59.4 (39.7 to 81.8)	17.3 (-22.0 to 84.3)	24.9 (-9.2 to 76.9)
**Spain**	3.4 (3.1 to 3.7)	-84.9 (-86.1 to -83.6)	-35.2 (-41.7 to -28.2)
**Sri Lanka**	14.4 (10.6 to 18.2)	-18.8 (-41.5 to 30.3)	16.7 (-7.5 to 49.2)
**Sudan**	28.5 (17.4 to 40.1)	-29.8 (-56.3 to 29.5)	-4.8 (-34.4 to 46.1)
**Suriname**	16.4 (13.1 to 21.0)	-27.5 (-44.5 to -0.9)	-11.1 (-25.9 to 3.4)
**Sweden**	2.0 (1.8 to 2.2)	-78.7 (-81.4 to -75.7)	-35.2 (-42.7 to -27.4)
**Switzerland**	1.8 (1.6 to 2.0)	-87.1 (-88.9 to -85.0)	-49.5 (-56.9 to -41.5)
**Syria**	9.0 (5.7 to 13.2)	-46.1 (-69.6 to 7.0)	-28.3 (-48.6 to 3.7)
**Taiwan**	10.2 (8.6 to 13.1)	-73.2 (-77.4 to -65.6)	-29.1 (-40.1 to -8.4)
**Tajikistan**	5.7 (4.0 to 8.0)	-60.2 (-73.3 to -27.8)	-4.6 (-36.4 to 53.7)
**Thailand**	26.3 (17.2 to 30.8)	-30.4 (-53.5 to 0.7)	-15.0 (-31.1 to 4.8)
**Timor-Leste**	19.9 (13.9 to 26.5)	-4.9 (-36.9 to 74.4)	27.0 (-10.9 to 91.9)
**Togo**	44.7 (32.5 to 59.4)	64.9 (9.6 to 148.4)	13.1 (-20.5 to 68.2)
**Tokelau**	7.6 (5.0 to 10.7)	-30.1 (-54.8 to 9.5)	-11.4 (-38.3 to 27.8)
**Tonga**	10.7 (6.9 to 15.5)	-30.5 (-57.0 to 5.1)	-10.8 (-40.9 to 27.3)
**Trinidad and Tobago**	11.1 (9.2 to 13.8)	-37.7 (-48.3 to -20.7)	-22.1 (-34.0 to -4.2)
**Tunisia**	17.0 (12.9 to 21.5)	-41.7 (-61.3 to 24.8)	-26.9 (-49.8 to 25.0)
**Turkmenistan**	5.1 (4.4 to 6.1)	-75.6 (-80.1 to -70.7)	-36.5 (-45.3 to -26.5)
**Tuvalu**	34.7 (25.0 to 46.6)	-22.8 (-49.0 to 29.0)	-33.3 (-51.8 to -3.8)
**Türkiye**	9.3 (6.9 to 12.3)	-50.1 (-69.0 to -5.6)	-0.9 (-24.1 to 33.6)
**Uganda**	63.7 (44.8 to 85.5)	92.6 (19.2 to 201.8)	26.9 (-12.5 to 82.3)
**Ukraine**	8.7 (7.3 to 11.1)	-44.0 (-56.1 to -21.5)	-22.5 (-37.0 to -1.3)
**United Arab Emirates**	7.7 (5.6 to 10.5)	-82.0 (-89.9 to -37.1)	-72.2 (-88.7 to 1.4)
**UK**	2.1 (2.0 to 2.3)	-77.9 (-79.5 to -76.2)	-40.9 (-45.7 to -35.5)
**Tanzania**	37.3 (27.2 to 49.8)	15.5 (-22.3 to 78.7)	12.2 (-18.9 to 58.8)
**Virgin Islands**	11.9 (9.7 to 14.4)	-45.7 (-58.9 to -28.8)	-6.6 (-25.6 to 19.7)
**USA**	12.8 (11.9 to 13.6)	-31.3 (-36.5 to -26.1)	13.2 (6.3 to 20.3)
**Uruguay**	11.8 (10.4 to 13.3)	-12.2 (-24.4 to 2.0)	-24.0 (-35.1 to -11.3)
**Uzbekistan**	9.6 (8.2 to 11.3)	-22.5 (-34.8 to -6.3)	-29.1 (-41.7 to -15.7)
**Vanuatu**	11.5 (6.9 to 16.5)	-1.4 (-35.3 to 51.3)	1.6 (-29.0 to 43.0)
**Venezuela**	17.6 (14.9 to 20.2)	-41.1 (-50.9 to -29.6)	-23.7 (-35.8 to -12.0)
**Viet Nam**	23.4 (13.8 to 38.7)	-29.9 (-57.3 to 26.0)	-26.6 (-48.5 to 19.2)
**Yemen**	23.9 (12.3 to 34.4)	-13.6 (-50.3 to 75.2)	-9.1 (-35.4 to 32.5)
**Zambia**	44.2 (32.5 to 60.0)	-8.7 (-41.0 to 57.3)	8.2 (-27.3 to 57.8)
**Zimbabwe**	20.1 (14.0 to 27.9)	-33.1 (-56.9 to -5.8)	-29.0 (-50.1 to 3.4)

95% UI=95% uncertainty interval

Table S8. Number of road injury YLLs (in thousands) and age-standardised road injury YLL rates (per 100 000) in 2023 and 1990, and the percentage change in both metrics from 1990 to 2023, globally and across the World Bank income groups and 21 GBD regions, stratified by sex. YLLs=years of life lost.

	YLLs in 2023		YLLs in 1990		Change in YLLs from 1990 to 2023	
	Number of YLLs, in thousands (95% UI)	Age-standardised YLL rates, per 100 000 (95% UI)	Number of YLLs, in thousands (95% UI)	Age-standardised YLL rates, per 100 000 (95% UI)	Percentage change in number of YLLs, in % (95% UI)	Percentage change in age-standardised YLL rate, in % (95% UI)
**Global**						
Total	66,700 (51,500 to 79,400)	826.6 (636.5 to 986.9)	67,000 (52,900 to 79,600)	1,225.7 (971.1 to 1,458.1)	-0.4 (-24.6 to 38.1)	-32.6 (-49.0 to -7.1)
Male	49,500 (36,100 to 61,100)	1,212.8 (883.2 to 1,503.4)	48,700 (35,400 to 61,900)	1,774.6 (1,295.5 to 2,266.9)	1.8 (-23.9 to 51.3)	-31.7 (-49.4 to 1.9)
Female	17,200 (10,300 to 22,500)	434.0 (259.6 to 573.6)	18,300 (12,500 to 24,600)	672.1 (462.5 to 903.1)	-6.3 (-36.0 to 49.5)	-35.4 (-55.7 to 3.3)
**World Bank low income**						
Total	14,400 (10,300 to 18,700)	2,134.9 (1,546.6 to 2,734.4)	6,020 (3,980 to 8,650)	1,979.1 (1,326.1 to 2,776.1)	138.5 (63.1 to 282.7)	7.9 (-26.8 to 71.4)
Male	9,840 (6,890 to 13,000)	2,991.5 (2,123.5 to 3,928.8)	3,970 (2,110 to 6,290)	2,750.4 (1,491.7 to 4,282.3)	148.1 (46.4 to 406.2)	8.8 (-34.7 to 119.7)
Female	4,520 (2,530 to 7,010)	1,309.1 (752.6 to 2,030.8)	2,050 (1,020 to 3,700)	1,253.0 (631.0 to 2,231.3)	119.9 (24.1 to 367.6)	4.5 (-40.7 to 118.2)
**World Bank lower-middle income**						
Total	28,300 (19,600 to 35,500)	905.7 (628.2 to 1,139.4)	16,200 (11,800 to 21,100)	961.9 (705.8 to 1,246.2)	74.7 (28.0 to 156.0)	-5.8 (-32.0 to 37.4)
Male	21,400 (13,600 to 27,900)	1,360.2 (857.7 to 1,771.7)	11,200 (7,360 to 15,300)	1,326.6 (882.2 to 1,806.1)	91.2 (30.5 to 203.8)	2.5 (-30.5 to 61.5)
Female	6,880 (3,640 to 9,520)	449.5 (235.3 to 622.6)	5,000 (2,980 to 7,160)	582.0 (352.9 to 815.9)	37.6 (-16.4 to 140.4)	-22.8 (-53.5 to 34.1)
**World Bank upper-middle income**						
Total	18,800 (15,600 to 21,600)	653.2 (545.6 to 750.8)	32,500 (22,900 to 41,300)	1,455.2 (1,019.0 to 1,864.6)	-42.0 (-55.7 to -7.8)	-55.1 (-65.8 to -28.7)
Male	14,300 (11,500 to 16,800)	968.6 (780.0 to 1,147.4)	24,200 (15,500 to 32,200)	2,141.6 (1,350.5 to 2,904.9)	-41.0 (-56.2 to 1.9)	-54.8 (-66.6 to -21.7)
Female	4,560 (3,190 to 5,640)	327.2 (229.6 to 403.6)	8,300 (4,980 to 12,400)	758.1 (456.8 to 1,128.5)	-45.1 (-63.1 to -0.3)	-56.8 (-71.1 to -22.5)
**World Bank high income**						
Total	5,190 (4,930 to 5,460)	381.5 (362.7 to 401.3)	12,300 (11,800 to 12,700)	1,022.2 (981.4 to 1,059.7)	-57.7 (-59.6 to -54.9)	-62.7 (-64.4 to -60.1)
Male	3,990 (3,760 to 4,210)	574.7 (540.1 to 608.4)	9,320 (8,900 to 9,800)	1,542.6 (1,470.9 to 1,622.7)	-57.2 (-59.6 to -53.9)	-62.7 (-65.0 to -59.7)
Female	1,200 (1,120 to 1,300)	180.2 (167.5 to 193.8)	2,960 (2,810 to 3,110)	499.8 (473.8 to 526.9)	-59.5 (-62.5 to -55.8)	-63.9 (-66.6 to -60.6)
**Andean Latin America**						
Total	707 (618 to 850)	1,055.7 (923.9 to 1,270.3)	409 (341 to 478)	1,078.4 (905.1 to 1,254.0)	72.7 (47.2 to 124.1)	-2.1 (-16.7 to 26.5)
Male	537 (453 to 685)	1,603.6 (1,353.0 to 2,048.4)	299 (234 to 371)	1,628.1 (1,282.9 to 2,020.6)	79.9 (48.2 to 158.4)	-1.5 (-18.2 to 41.5)
Female	170 (133 to 218)	518.8 (409.4 to 667.2)	111 (83.2 to 140)	546.8 (418.7 to 690.1)	53.3 (23.2 to 135.5)	-5.1 (-23.8 to 43.4)
**Australasia**						
Total	76.8 (70.1 to 84.0)	247.6 (227.5 to 270.6)	190 (184 to 197)	921.2 (892.6 to 953.4)	-59.5 (-63.1 to -55.7)	-73.1 (-75.5 to -70.5)
Male	57.3 (51.4 to 63.3)	366.8 (330.2 to 405.4)	136 (131 to 142)	1,311.9 (1,263.5 to 1,365.1)	-58.0 (-62.4 to -53.4)	-72.0 (-75.0 to -68.9)
Female	19.5 (17.3 to 22.1)	128.2 (112.6 to 145.4)	53.5 (50.3 to 56.9)	528.4 (497.8 to 562.9)	-63.5 (-68.0 to -58.5)	-75.7 (-78.8 to -72.4)
**Caribbean**						
Total	611 (462 to 786)	1,259.9 (949.0 to 1,628.9)	502 (400 to 616)	1,356.1 (1,092.2 to 1,647.1)	21.7 (-2.0 to 47.1)	-7.1 (-25.6 to 12.6)
Male	460 (342 to 609)	1,898.4 (1,406.3 to 2,522.9)	360 (283 to 465)	2,004.6 (1,595.5 to 2,562.9)	27.7 (1.2 to 60.3)	-5.3 (-24.4 to 18.8)
Female	151 (83.1 to 233)	638.0 (345.7 to 997.6)	141 (88.3 to 206)	734.9 (466.7 to 1,062.9)	6.5 (-21.3 to 50.5)	-13.2 (-37.2 to 24.2)
**Central Asia**						
Total	484 (434 to 539)	479.9 (429.9 to 534.7)	613 (551 to 677)	870.7 (784.8 to 962.7)	-21.1 (-30.2 to -9.4)	-44.9 (-51.2 to -36.6)
Male	373 (330 to 418)	745.0 (658.3 to 835.9)	466 (404 to 532)	1,365.5 (1,184.6 to 1,560.6)	-20.0 (-31.3 to -5.0)	-45.4 (-53.1 to -35.3)
Female	111 (95.4 to 130)	219.8 (189.5 to 258.1)	147 (123 to 172)	398.9 (334.8 to 464.3)	-24.7 (-39.4 to -3.6)	-44.9 (-55.4 to -29.3)
**Central Europe**						
Total	328 (306 to 356)	283.0 (263.7 to 307.1)	1,200 (1,130 to 1,270)	946.9 (889.2 to 1,002.3)	-72.7 (-74.3 to -70.2)	-70.1 (-71.8 to -67.6)
Male	258 (239 to 283)	440.8 (408.2 to 480.9)	941 (872 to 1,010)	1,484.6 (1,375.7 to 1,590.7)	-72.6 (-74.5 to -69.4)	-70.3 (-72.4 to -67.0)
Female	70.1 (63.2 to 77.6)	122.3 (110.6 to 135.6)	260 (241 to 278)	414.9 (385.1 to 443.6)	-73.1 (-75.5 to -69.2)	-70.5 (-73.4 to -66.4)
**Central Latin America**						
Total	1,990 (1,850 to 2,160)	740.8 (688.3 to 801.9)	2,020 (1,880 to 2,170)	1,259.1 (1,163.7 to 1,353.4)	-1.5 (-10.8 to 11.5)	-41.2 (-46.7 to -33.4)
Male	1,600 (1,460 to 1,760)	1,210.0 (1,107.8 to 1,331.2)	1,560 (1,410 to 1,700)	2,008.3 (1,825.5 to 2,197.5)	2.4 (-9.1 to 19.1)	-39.8 (-46.6 to -30.1)

Female	396	297.3	463	549.5	-14.6	-45.9
	(364 to 436)	(273.6 to 327.7)	(416 to 512)	(493.7 to 604.7)	(-25.3 to 0.5)	(-52.8 to -36.5)
**Central sub-Saharan Africa**						
Total	6,510	4,480.1	2,020	3,503.6	222.2	27.9
	(4,430 to 8,550)	(3,027.3 to 5,841.9)	(1,070 to 3,040)	(1,853.1 to 5,174.0)	(115.7 to 396.5)	(-13.4 to 97.6)
Male	4,160	5,847.8	1,290	4,688.1	221.8	24.7
	(2,660 to 5,700)	(3,735.7 to 8,003.4)	(647 to 2,160)	(2,345.2 to 7,769.1)	(95.3 to 463.8)	(-22.7 to 120.6)
Female	2,350	3,139.7	728	2,361.0	222.9	33.0
	(1,160 to 3,950)	(1,527.4 to 5,266.9)	(251 to 1,380)	(816.0 to 4,479.4)	(83.0 to 557.8)	(-23.8 to 163.0)
**East Asia**						
Total	6,580	408.7	20,500	1,593.5	-67.9	-74.3
	(4,900 to 8,160)	(318.2 to 497.8)	(12,600 to 28,200)	(964.8 to 2,214.6)	(-78.9 to -38.3)	(-82.7 to -50.1)
Male	4,780	573.6	15,200	2,288.6	-68.5	-74.9
	(3,250 to 6,290)	(397.5 to 745.5)	(7,630 to 22,000)	(1,135.2 to 3,361.0)	(-81.3 to -24.9)	(-84.7 to -39.3)
Female	1,790	233.6	5,260	857.4	-65.9	-72.8
	(1,150 to 2,300)	(152.4 to 308.2)	(2,710 to 8,600)	(440.2 to 1,407.0)	(-79.8 to -20.4)	(-83.6 to -36.3)
**Eastern Europe**						
Total	883	441.3	2,830	1,245.5	-68.9	-64.6
	(802 to 982)	(401.5 to 488.0)	(2,570 to 3,090)	(1,130.3 to 1,355.9)	(-73.2 to -63.4)	(-69.4 to -58.8)
Male	693	697.2	2,220	1,982.9	-68.7	-64.8
	(616 to 784)	(619.8 to 786.3)	(1,970 to 2,440)	(1,758.8 to 2,187.8)	(-73.8 to -62.9)	(-70.6 to -58.4)
Female	190	193.3	619	536.4	-69.3	-64.0
	(162 to 217)	(165.3 to 220.8)	(528 to 718)	(457.3 to 619.9)	(-75.7 to -61.2)	(-71.2 to -54.6)
**Eastern sub-Saharan Africa**						
Total	7,960	1,884.3	3,620	1,883.3	120.0	0.1
	(5,730 to 10,500)	(1,359.7 to 2,502.2)	(2,510 to 5,050)	(1,304.8 to 2,565.4)	(44.0 to 253.7)	(-34.2 to 61.7)
Male	5,870	2,851.8	2,440	2,680.6	141.1	6.4
	(3,950 to 8,220)	(1,951.6 to 4,026.0)	(1,300 to 3,740)	(1,462.2 to 4,074.7)	(39.4 to 381.5)	(-36.7 to 108.5)
Female	2,090	966.4	1,180	1,134.6	76.5	-14.8
	(1,190 to 3,150)	(552.3 to 1,455.8)	(650 to 1,870)	(627.3 to 1,746.1)	(-9.0 to 278.7)	(-55.6 to 79.0)
**High-income Asia Pacific**						
Total	259	122.4	1,570	915.5	-83.5	-86.6
	(223 to 332)	(104.4 to 161.1)	(1,380 to 1,730)	(803.3 to 1,012.3)	(-86.0 to -75.8)	(-88.7 to -80.2)
Male	192	186.8	1,200	1,375.0	-83.9	-86.4
	(161 to 265)	(155.7 to 253.9)	(998 to 1,400)	(1,138.4 to 1,620.9)	(-86.6 to -73.4)	(-88.8 to -77.7)
Female	66.6	56.4	376	452.2	-82.3	-87.5
	(54.2 to 90.7)	(47.0 to 79.0)	(305 to 438)	(358.3 to 523.6)	(-86.0 to -71.9)	(-90.4 to -79.0)
**High-income North America**						
Total	2,240	607.0	2,830	1,002.3	-20.8	-39.4
	(2,100 to 2,390)	(565.4 to 648.3)	(2,760 to 2,900)	(976.6 to 1,026.3)	(-26.6 to -14.2)	(-44.0 to -34.1)
Male	1,620	866.9	2,000	1,407.2	-18.8	-38.4
	(1,490 to 1,750)	(793.2 to 936.2)	(1,940 to 2,050)	(1,360.5 to 1,446.7)	(-25.4 to -11.5)	(-43.8 to -32.7)
Female	618	343.7	834	598.2	-25.9	-42.5
	(561 to 677)	(310.8 to 376.0)	(795 to 877)	(569.3 to 627.4)	(-33.8 to -16.5)	(-48.6 to -35.7)
**North Africa and Middle East**						
Total	5,550	862.1	5,440	1,532.1	2.1	-43.7
	(4,250 to 6,750)	(662.5 to 1,047.0)	(3,390 to 7,620)	(967.5 to 2,125.6)	(-28.4 to 66.4)	(-60.4 to -7.9)
Male	4,300	1,281.0	3,830	2,171.9	12.2	-41.0
	(2,980 to 5,340)	(892.2 to 1,599.8)	(2,030 to 5,790)	(1,163.2 to 3,254.4)	(-25.9 to 111.1)	(-60.6 to 10.2)
Female	1,260	410.3	1,610	862.4	-22.0	-52.4
	(810 to 1,710)	(264.3 to 559.9)	(694 to 2,580)	(375.2 to 1,391.7)	(-56.1 to 79.9)	(-73.2 to 10.2)
**Oceania**						
Total	127	869.3	61.3	930.9	107.6	-6.6
	(91.2 to 172)	(622.2 to 1,168.9)	(43.3 to 83.5)	(660.1 to 1,246.5)	(39.8 to 222.8)	(-37.0 to 44.3)
Male	82.1	1,106.6	41.0	1,240.0	100.2	-10.8
	(55.1 to 126)	(741.0 to 1,666.8)	(23.1 to 64.3)	(723.3 to 1,895.4)	(19.9 to 249.9)	(-45.6 to 53.7)
Female	45.1	621.2	20.2	603.1	122.7	3.0
	(25.1 to 65.1)	(348.8 to 902.3)	(11.0 to 34.3)	(330.7 to 1,008.2)	(21.9 to 300.5)	(-43.4 to 83.2)
**South Asia**						
Total	14,000	722.1	8,100	759.6	73.2	-4.9
	(8,960 to 18,200)	(459.5 to 935.4)	(6,200 to 10,300)	(585.2 to 967.8)	(17.8 to 146.5)	(-36.3 to 36.0)
Male	11,200	1,126.8	5,770	1,055.3	94.4	6.8
	(6,760 to 14,900)	(677.5 to 1,498.4)	(3,880 to 7,650)	(724.7 to 1,398.4)	(24.3 to 200.5)	(-32.6 to 63.7)
Female	2,820	306.0	2,330	439.4	20.7	-30.3
	(1,460 to 4,010)	(157.5 to 437.0)	(1,520 to 3,320)	(294.4 to 615.2)	(-25.4 to 120.5)	(-56.6 to 24.5)
**Southeast Asia**						
Total	6,660	896.0	5,360	1,116.9	24.3	-19.8
	(5,010 to 8,200)	(670.7 to 1,103.4)	(3,670 to 6,900)	(774.7 to 1,431.8)	(-7.5 to 91.3)	(-39.7 to 21.0)
Male	4,940	1,320.2	3,990	1,687.5	23.9	-21.8
	(3,640 to 6,360)	(972.2 to 1,705.1)	(2,400 to 5,480)	(1,024.6 to 2,303.1)	(-13.6 to 125.1)	(-45.2 to 42.3)
Female	1,720	468.4	1,370	569.6	25.6	-17.8
	(991 to 2,340)	(267.8 to 640.0)	(724 to 2,090)	(306.3 to 855.7)	(-24.4 to 150.5)	(-50.2 to 62.2)
**Southern Latin America**						
Total	342	471.0	327	658.5	4.8	-28.5
	(323 to 364)	(443.7 to 501.2)	(307 to 349)	(619.2 to 703.2)	(-2.8 to 13.0)	(-33.9 to -22.8)
Male	275	765.8	249	1,029.0	10.7	-25.6
	(257 to 295)	(713.5 to 820.3)	(230 to 270)	(952.8 to 1,115.3)	(1.0 to 20.1)	(-32.1 to -19.1)
Female	67.0	185.0	77.8	305.8	-13.9	-39.5
	(62.0 to 71.9)	(171.0 to 199.1)	(71.9 to 83.9)	(282.3 to 329.6)	(-21.4 to -4.5)	(-44.5 to -32.9)
**Southern sub-Saharan Africa**						
Total	1,640	1,758.8	1,090	2,075.4	50.9	-15.3
	(1,270 to 2,100)	(1,367.6 to 2,254.3)	(842 to 1,320)	(1,615.9 to 2,510.5)	(9.4 to 103.9)	(-39.0 to 15.5)
Male	1,160	2,533.9	757	3,050.7	52.7	-16.9
	(822 to 1,570)	(1,799.1 to 3,452.6)	(528 to 965)	(2,120.5 to 3,911.8)	(6.4 to 116.5)	(-42.5 to 18.2)
Female	485	1,030.4	330	1,186.5	46.8	-13.2
	(320 to 709)	(682.3 to 1,500.5)	(222 to 474)	(787.3 to 1,709.7)	(-12.7 to 152.0)	(-48.2 to 47.9)

**Tropical Latin America**						
Total	1,950	847.4	2,130	1,399.1	-8.6	-39.4
	(1,890 to 2,010)	(822.2 to 877.5)	(1,950 to 2,340)	(1,276.3 to 1,535.6)	(-16.5 to 0.1)	(-45.0 to -33.6)
Male	1,610	1,422.3	1,640	2,207.6	-2.0	-35.6
	(1,550 to 1,670)	(1,369.6 to 1,475.6)	(1,470 to 1,830)	(1,976.1 to 2,472.0)	(-12.9 to 8.9)	(-43.0 to -28.1)
Female	337	297.5	488	624.4	-30.9	-52.3
	(319 to 353)	(280.2 to 313.7)	(431 to 553)	(552.3 to 710.5)	(-39.7 to -20.9)	(-58.3 to -45.6)
**Western Europe**						
Total	715	164.3	3,230	840.1	-77.9	-80.4
	(656 to 777)	(151.2 to 178.7)	(3,130 to 3,340)	(813.6 to 866.7)	(-79.6 to -75.9)	(-82.0 to -78.8)
Male	572	263.6	2,470	1,279.4	-76.9	-79.4
	(517 to 630)	(240.2 to 291.6)	(2,390 to 2,570)	(1,234.7 to 1,327.5)	(-78.9 to -74.6)	(-81.2 to -77.4)
Female	144	63.9	761	397.6	-81.1	-83.9
	(124 to 166)	(55.3 to 73.1)	(720 to 799)	(375.5 to 419.1)	(-83.7 to -78.4)	(-86.1 to -81.6)
**Western sub-Saharan Africa**						
Total	7,060	1,408.9	2,990	1,460.4	135.9	-3.5
	(4,680 to 9,660)	(940.3 to 1,890.9)	(1,840 to 4,290)	(930.2 to 2,075.8)	(53.5 to 278.3)	(-35.9 to 49.1)
Male	4,780	2,015.3	1,830	1,882.0	161.0	7.1
	(3,100 to 6,780)	(1,308.9 to 2,790.5)	(1,000 to 3,010)	(1,061.8 to 2,984.9)	(57.9 to 398.1)	(-33.5 to 97.6)
Female	2,280	850.4	1,160	1,042.4	96.2	-18.4
	(1,140 to 3,450)	(423.6 to 1,258.3)	(570 to 2,000)	(537.5 to 1,765.2)	(3.1 to 304.2)	(-55.0 to 61.9)

95% UI=95% uncertainty interval

Table S9. Number of road injury YLDs (in thousands) and age-standardised road injury YLD rates (per 100 000) in 2023 and 1990, and the percentage change in both metrics from 1990 to 2023, globally and across the World Bank income groups and 21 GBD regions, stratified by sex. YLDs=years lived with disability.

	YLDs in 2023		YLDs in 1990		Change in YLDs from 1990 to 2023	
	Number of YLDs, in thousands (95% UI)	Age-standardised YLD rates, per 100 000 (95% UI)	Number of YLDs, in thousands (95% UI)	Age-standardised YLD rates, per 100 000 (95% UI)	Percentage change in number of YLDs, in % (95% UI)	Percentage change in age-standardised YLD rate, in % (95% UI)
**Global**						
Total	8,560	97.4	7,890	168.2	8.5	-42.1
	(6,250 to 11,200)	(71.2 to 127.6)	(5,830 to 10,400)	(124.7 to 221.5)	(5.4 to 11.2)	(-43.3 to -41.0)
Male	5,830	135.1	5,010	217.6	16.3	-37.9
	(4,250 to 7,620)	(98.4 to 176.7)	(3,680 to 6,570)	(160.7 to 285.6)	(13.3 to 19.1)	(-39.2 to -36.8)
Female	2,730	60.8	2,880	120.6	-5.1	-49.5
	(2,000 to 3,590)	(44.7 to 80.1)	(2,130 to 3,810)	(89.3 to 159.3)	(-7.9 to -2.5)	(-50.8 to -48.4)
**World Bank low income**						
Total	443	94.5	260	126.6	70.4	-25.3
	(327 to 578)	(69.8 to 123.0)	(190 to 339)	(93.1 to 164.7)	(66.0 to 74.1)	(-26.7 to -24.0)
Male	284	124.1	151	153.7	88.6	-19.3
	(210 to 371)	(91.4 to 161.6)	(110 to 197)	(112.7 to 200.2)	(84.0 to 92.8)	(-20.8 to -17.8)
Female	159	66.4	109	101.2	45.3	-34.3
	(117 to 206)	(49.2 to 86.2)	(79.9 to 142)	(74.8 to 131.3)	(41.5 to 48.9)	(-35.8 to -33.0)
**World Bank lower-middle income**						
Total	2,000	72.2	1,160	91.3	73.2	-20.9
	(1,470 to 2,620)	(52.8 to 94.8)	(848 to 1,500)	(67.3 to 118.1)	(67.4 to 77.9)	(-23.1 to -19.2)
Male	1,390	100.6	733	114.3	89.3	-12.0
	(1,020 to 1,820)	(73.3 to 132.0)	(541 to 952)	(84.1 to 147.9)	(83.4 to 93.9)	(-14.4 to -10.2)
Female	616	44.5	424	67.2	45.5	-33.8
	(453 to 808)	(32.6 to 58.5)	(312 to 550)	(49.9 to 86.9)	(40.2 to 50.1)	(-35.8 to -32.1)
**World Bank upper-middle income**						
Total	3,940	111.1	3,300	174.1	19.3	-36.2
	(2,880 to 5,160)	(81.2 to 146.0)	(2,430 to 4,320)	(129.0 to 227.4)	(14.4 to 24.1)	(-38.4 to -34.2)
Male	2,750	157.2	2,110	223.6	30.3	-29.7
	(2,000 to 3,590)	(114.5 to 206.0)	(1,550 to 2,750)	(165.8 to 292.2)	(25.4 to 35.1)	(-31.9 to -27.8)
Female	1,190	66.2	1,200	125.5	0.0	-47.2
	(869 to 1,570)	(48.4 to 87.4)	(882 to 1,570)	(93.0 to 164.1)	(-5.0 to 5.1)	(-49.5 to -45.1)
**World Bank high income**						
Total	2,170	113.7	3,160	233.8	-31.5	-51.3
	(1,580 to 2,890)	(82.9 to 152.5)	(2,300 to 4,220)	(170.0 to 311.6)	(-33.2 to -29.8)	(-52.4 to -50.5)
Male	1,410	151.8	2,020	314.8	-30.1	-51.8
	(1,030 to 1,870)	(110.6 to 202.4)	(1,470 to 2,680)	(229.5 to 417.6)	(-32.2 to -28.4)	(-53.0 to -51.0)
Female	759	76.3	1,150	159.3	-33.8	-52.1
	(548 to 1,010)	(55.5 to 102.4)	(833 to 1,530)	(116.0 to 212.5)	(-35.3 to -32.4)	(-53.0 to -51.2)
**Andean Latin America**						
Total	56.8	84.4	36.3	126.1	56.7	-33.1
	(41.7 to 74.2)	(61.8 to 110.1)	(26.9 to 47.0)	(93.9 to 163.1)	(49.9 to 62.9)	(-35.7 to -30.9)
Male	39.5	120.3	23.5	169.7	67.7	-29.1
	(29.0 to 51.4)	(88.3 to 156.6)	(17.5 to 30.5)	(126.3 to 219.6)	(61.0 to 74.4)	(-31.6 to -26.6)
Female	17.4	50.4	12.7	84.4	36.3	-40.3
	(12.7 to 22.9)	(36.8 to 66.6)	(9.38 to 16.5)	(62.2 to 109.0)	(29.2 to 42.3)	(-43.0 to -38.0)
**Australasia**						
Total	38.2	92.1	49.0	221.4	-22.1	-58.4
	(27.8 to 50.9)	(67.2 to 123.2)	(35.3 to 65.7)	(159.1 to 297.0)	(-25.0 to -19.2)	(-59.7 to -57.0)
Male	23.8	118.1	30.3	280.2	-21.5	-57.8
	(17.4 to 31.7)	(86.2 to 157.6)	(21.8 to 40.2)	(201.6 to 372.1)	(-24.4 to -18.6)	(-59.4 to -56.3)
Female	14.4	67.0	18.7	164.0	-23.1	-59.1
	(10.5 to 19.2)	(48.9 to 90.6)	(13.4 to 25.4)	(117.7 to 222.9)	(-26.2 to -19.9)	(-60.7 to -57.6)
**Caribbean**						
Total	44.7	85.6	38.0	121.9	17.5	-29.8
	(32.9 to 58.3)	(63.0 to 111.5)	(28.1 to 49.5)	(90.3 to 158.9)	(14.7 to 20.4)	(-31.3 to -28.3)
Male	30.4	119.6	25.0	166.4	21.6	-28.1
	(22.3 to 39.6)	(87.6 to 156.1)	(18.5 to 32.7)	(123.0 to 216.6)	(18.7 to 25.0)	(-29.7 to -26.3)
Female	14.2	53.7	13.0	79.7	9.6	-32.6
	(10.6 to 18.7)	(39.9 to 70.5)	(9.6 to 16.9)	(59.2 to 103.8)	(6.5 to 12.6)	(-34.3 to -31.1)
**Central Asia**						
Total	82.0	82.2	70.1	120.8	17.0	-32.0
	(59.9 to 108)	(59.9 to 108.5)	(51.4 to 91.9)	(88.5 to 158.3)	(13.6 to 20.0)	(-33.8 to -30.5)
Male	56.1	117.5	46.9	174.9	19.6	-32.9
	(41.1 to 74.0)	(85.8 to 155.1)	(34.3 to 61.4)	(127.9 to 228.8)	(16.2 to 22.5)	(-34.6 to -31.5)
Female	25.9	50.3	23.2	74.6	11.7	-32.5
	(18.8 to 34.4)	(36.5 to 66.9)	(17.1 to 30.5)	(54.9 to 98.0)	(7.9 to 15.3)	(-34.6 to -30.6)
**Central Europe**						

Total	139	86.9	269	192.2	-48.3	-54.8
	(99.9 to 185)	(62.8 to 116.2)	(199 to 352)	(142.3 to 252.2)	(-49.9 to -46.6)	(-56.0 to -53.5)
Male	94.6	123.2	180	269.5	-47.3	-54.3
	(67.8 to 126)	(88.6 to 164.0)	(133 to 234)	(199.3 to 352.5)	(-48.9 to -45.6)	(-55.5 to -53.0)
Female	44.5	52.6	89.2	122.1	-50.1	-56.9
	(31.9 to 59.5)	(38.1 to 71.0)	(66.1 to 117)	(90.9 to 160.6)	(-52.1 to -48.2)	(-58.5 to -55.3)
**Central Latin America**						
Total	301	109.0	218	178.2	38.0	-38.8
	(222 to 393)	(80.6 to 142.8)	(160 to 284)	(132.0 to 231.1)	(32.5 to 43.3)	(-40.7 to -36.9)
Male	219	167.7	154	263.0	42.6	-36.2
	(162 to 286)	(124.1 to 218.9)	(114 to 200)	(194.7 to 340.4)	(36.8 to 48.0)	(-38.2 to -34.2)
Female	81.3	56.5	64.0	98.6	26.9	-42.7
	(60.0 to 107)	(41.7 to 74.4)	(47.1 to 83.9)	(73.0 to 128.6)	(21.4 to 32.1)	(-44.7 to -40.5)
**Central sub-Saharan Africa**						
Total	124	123.9	59.5	152.7	108.3	-18.9
	(91.1 to 161)	(91.2 to 160.7)	(43.7 to 77.6)	(112.0 to 197.3)	(100.4 to 115.2)	(-21.3 to -16.4)
Male	75.6	156.0	30.7	167.7	146.2	-6.9
	(55.2 to 98.1)	(114.3 to 203.1)	(22.3 to 39.8)	(122.6 to 216.6)	(136.9 to 154.2)	(-9.9 to -4.2)
Female	48.4	93.9	28.8	139.4	67.9	-32.6
	(35.8 to 63.2)	(69.7 to 121.0)	(21.2 to 37.8)	(102.4 to 180.9)	(61.1 to 74.5)	(-34.8 to -30.4)
**East Asia**						
Total	2,320	112.1	1,800	161.4	28.9	-30.6
	(1,680 to 3,040)	(81.5 to 147.5)	(1,320 to 2,340)	(118.9 to 210.5)	(22.3 to 35.2)	(-33.7 to -27.8)
Male	1,580	154.0	1,120	196.7	41.7	-21.7
	(1,150 to 2,080)	(112.4 to 202.4)	(819 to 1,460)	(144.8 to 256.5)	(35.0 to 48.0)	(-25.0 to -18.9)
Female	731	69.6	678	124.9	7.9	-44.3
	(528 to 973)	(50.7 to 92.3)	(499 to 885)	(92.1 to 163.0)	(1.1 to 14.0)	(-47.2 to -41.6)
**Eastern Europe**						
Total	369	131.6	624	243.0	-40.9	-45.9
	(264 to 489)	(94.5 to 175.5)	(461 to 820)	(179.9 to 320.8)	(-42.9 to -38.6)	(-47.6 to -44.0)
Male	239	189.0	400	351.9	-40.2	-46.3
	(171 to 316)	(135.4 to 250.7)	(296 to 525)	(260.3 to 463.0)	(-42.2 to -37.7)	(-47.9 to -44.5)
Female	129	82.7	224	155.8	-42.1	-46.9
	(92.9 to 173)	(59.4 to 111.8)	(165 to 296)	(115.0 to 207.5)	(-44.2 to -39.8)	(-48.8 to -45.0)
**Eastern sub-Saharan Africa**						
Total	145	49.8	89.4	72.1	61.8	-31.0
	(107 to 188)	(36.7 to 65.1)	(65.5 to 116)	(53.3 to 92.7)	(58.2 to 65.4)	(-32.0 to -29.8)
Male	95.2	67.8	52.5	88.2	81.3	-23.1
	(70.0 to 124)	(49.8 to 88.6)	(38.5 to 68.0)	(65.1 to 113.1)	(77.3 to 85.2)	(-24.2 to -21.9)
Female	49.5	33.0	36.9	56.7	34.2	-41.8
	(36.6 to 64.5)	(24.2 to 43.2)	(27.0 to 47.5)	(41.9 to 73.1)	(30.0 to 37.8)	(-43.1 to -40.5)
**High-income Asia Pacific**						
Total	224	77.2	449	231.2	-50.1	-66.6
	(163 to 296)	(56.0 to 102.8)	(328 to 596)	(169.0 to 307.1)	(-51.8 to -48.2)	(-67.5 to -65.7)
Male	143	103.1	295	316.0	-51.6	-67.4
	(105 to 189)	(75.1 to 136.3)	(216 to 390)	(231.6 to 417.0)	(-53.4 to -49.9)	(-68.5 to -66.5)
Female	81.0	51.5	153	151.7	-47.2	-66.1
	(58.4 to 111)	(37.3 to 70.1)	(112 to 205)	(110.1 to 203.1)	(-49.0 to -45.2)	(-67.2 to -65.0)
**High-income North America**						
Total	778	158.1	839	267.4	-7.3	-40.9
	(568 to 1,040)	(115.6 to 211.8)	(603 to 1,130)	(192.3 to 359.9)	(-10.3 to -4.5)	(-42.4 to -39.4)
Male	480	200.1	503	337.5	-4.6	-40.7
	(354 to 636)	(147.2 to 267.1)	(364 to 674)	(244.3 to 452.6)	(-8.0 to -1.3)	(-42.3 to -39.2)
Female	298	117.5	336	202.4	-11.4	-42.0
	(214 to 399)	(84.7 to 157.9)	(240 to 453)	(144.6 to 273.4)	(-13.7 to -9.1)	(-43.2 to -40.7)
**North Africa and Middle East**						
Total	929	152.6	732	281.8	27.0	-45.9
	(681 to 1,230)	(111.7 to 200.9)	(541 to 960)	(209.4 to 368.4)	(20.6 to 31.7)	(-48.0 to -44.3)
Male	650	204.6	454	349.9	43.2	-41.5
	(475 to 859)	(149.5 to 269.8)	(334 to 594)	(258.7 to 457.8)	(36.8 to 47.9)	(-43.7 to -40.0)
Female	280	96.6	278	210.8	0.6	-54.2
	(205 to 369)	(70.5 to 127.2)	(206 to 366)	(157.3 to 275.4)	(-5.3 to 5.0)	(-56.4 to -52.6)
**Oceania**						
Total	11.8	99.8	5.37	114.5	118.9	-12.8
	(8.65 to 15.3)	(73.1 to 130.8)	(3.95 to 6.97)	(84.3 to 148.9)	(112.6 to 125.1)	(-14.9 to -10.7)
Male	8.16	133.7	3.67	149.9	122.6	-10.8
	(6.01 to 10.7)	(98.2 to 174.7)	(2.7 to 4.77)	(110.8 to 195.7)	(115.9 to 129.5)	(-13.3 to -8.3)
Female	3.60	64.0	1.71	76.8	110.9	-16.7
	(2.63 to 4.69)	(46.6 to 83.9)	(1.25 to 2.2)	(56.1 to 99.3)	(103.6 to 118.8)	(-19.2 to -13.8)
**South Asia**						
Total	1,160	64.6	576	69.4	100.8	-6.8
	(850 to 1,510)	(47.4 to 84.6)	(422 to 742)	(51.2 to 89.2)	(94.2 to 106.4)	(-9.3 to -4.8)

Male	813	89.7	373	86.7	118.1	3.6
	(594 to 1,060)	(65.7 to 117.2)	(274 to 480)	(63.6 to 111.5)	(111.2 to 123.9)	(0.9 to 5.8)
Female	344	39.2	204	50.3	69.2	-22.1
	(254 to 451)	(28.8 to 51.5)	(151 to 262)	(37.5 to 64.7)	(62.3 to 74.5)	(-24.6 to -20.2)
**Southeast Asia**						
Total	593	77.8	533	141.5	11.2	-45.0
	(430 to 782)	(56.6 to 102.6)	(389 to 699)	(103.2 to 185.3)	(7.5 to 14.6)	(-46.5 to -43.7)
Male	425	114.2	350	192.0	21.4	-40.5
	(308 to 560)	(82.8 to 150.5)	(257 to 459)	(140.0 to 251.3)	(17.8 to 24.8)	(-42.0 to -39.2)
Female	168	43.3	183	94.4	-8.4	-54.2
	(123 to 222)	(31.7 to 57.2)	(134 to 240)	(69.6 to 123.4)	(-11.8 to -4.8)	(-55.7 to -52.9)
**Southern Latin America**						
Total	107	130.5	75.7	158.2	41.9	-17.5
	(78.7 to 141)	(95.7 to 171.6)	(55.6 to 99.3)	(116.2 to 207.3)	(35.8 to 46.9)	(-20.8 to -14.7)
Male	72.3	186.0	50.1	221.0	44.3	-15.9
	(52.8 to 94.6)	(135.7 to 243.2)	(36.8 to 65.3)	(162.4 to 287.6)	(38.0 to 49.7)	(-19.3 to -12.9)
Female	35.1	80.5	25.6	101.6	37.1	-20.7
	(25.5 to 46.5)	(58.4 to 106.7)	(18.6 to 33.4)	(74.0 to 132.9)	(31.0 to 42.2)	(-24.1 to -17.7)
**Southern sub-Saharan Africa**						
Total	114	137.3	88.6	232.8	29.0	-41.0
	(83.0 to 149)	(100.0 to 178.9)	(64.0 to 115)	(168.3 to 301.0)	(25.4 to 32.9)	(-42.6 to -39.5)
Male	76.9	199.2	51.0	291.3	50.9	-31.6
	(55.8 to 101)	(145.2 to 259.7)	(36.8 to 66.6)	(211.2 to 376.8)	(46.9 to 55.0)	(-33.3 to -30.0)
Female	37.4	84.8	37.7	183.2	-0.6	-53.7
	(27.2 to 48.8)	(61.7 to 110.4)	(27.1 to 48.9)	(132.0 to 235.8)	(-3.7 to 3.2)	(-55.0 to -52.1)
**Tropical Latin America**						
Total	330	128.6	248	197.6	32.9	-34.9
	(241 to 433)	(93.7 to 169.1)	(182 to 326)	(145.9 to 258.1)	(26.2 to 39.4)	(-37.7 to -31.8)
Male	247	202.6	170	281.4	45.7	-28.0
	(180 to 324)	(148.0 to 265.6)	(125 to 222)	(207.6 to 367.1)	(38.4 to 52.9)	(-31.2 to -24.6)
Female	83.3	62.2	78.9	119.8	5.6	-48.1
	(60.8 to 109)	(45.4 to 81.2)	(58.4 to 103)	(88.6 to 155.5)	(0.4 to 11.9)	(-50.1 to -45.6)
**Western Europe**						
Total	523	84.5	1,010	218.4	-48.3	-61.3
	(378 to 691)	(61.6 to 112.2)	(732 to 1,340)	(157.9 to 289.8)	(-49.8 to -46.6)	(-62.4 to -60.1)
Male	344	115.1	655	299.3	-47.5	-61.5
	(249 to 456)	(84.0 to 153.3)	(474 to 868)	(217.2 to 396.4)	(-49.4 to -45.5)	(-62.9 to -60.3)
Female	179	55.2	356	143.1	-49.8	-61.5
	(128 to 241)	(40.1 to 74.0)	(258 to 475)	(103.6 to 191.0)	(-51.3 to -48.1)	(-62.5 to -60.2)
**Western sub-Saharan Africa**						
Total	181	53.2	84.4	62.9	114.1	-15.4
	(133 to 235)	(39.1 to 69.2)	(61.7 to 109)	(46.1 to 81.3)	(110.0 to 117.7)	(-17.0 to -14.2)
Male	115	71.1	49.7	74.9	130.2	-5.1
	(84.1 to 150)	(52.3 to 92.9)	(36.4 to 64.3)	(55.0 to 97.3)	(125.9 to 133.8)	(-6.7 to -3.9)
Female	66.2	36.9	34.6	50.3	91.1	-26.7
	(48.7 to 85.7)	(27.0 to 48.1)	(25.5 to 44.7)	(37.1 to 64.6)	(86.5 to 94.5)	(-28.3 to -25.4)

95% UI=95% uncertainty interval

Table S10. Number of road injury DALYs (in thousands) and age-standardised road injury DALY rates (per 100 000) in 2023 and 1990, and the percentage change in both metrics from 1990 to 2023, globally and across the World Bank income groups and 21 GBD regions, stratified by sex. DALYs=disability-adjusted life years.

	DALYs in 2023		DALYs in 1990		Change in DALYs from 1990 to 2023	
	Number of DALYs, in thousands (95% UI)	Age-standardised DALY rates, per 100 000 (95% UI)	Number of DALY, in thousands (95% UI)	Age-standardised DALY rates, per 100 000 (95% UI)	Percentage change in number of DALYs, in % (95% UI)	Percentage change in age-standardised DALY rate, in % (95% UI)
**Global**						
Total	75,300	924.1	74,900	1,393.9	0.5	-33.7
	(59,800 to 89,200)	(731.7 to 1,094.2)	(60,300 to 87,800)	(1,122.6 to 1,627.5)	(-21.3 to 34.5)	(-48.3 to -12.1)
Male	55,400	1,348.0	53,700	1,992.2	3.1	-32.3
	(41,200 to 66,400)	(999.9 to 1,623.5)	(40,600 to 67,200)	(1,516.2 to 2,491.8)	(-20.6 to 47.8)	(-48.2 to -3.4)
Female	19,900	494.9	21,200	792.7	-6.1	-37.6
	(13,100 to 25,400)	(321.3 to 638.4)	(15,100 to 27,700)	(571.5 to 1,029.2)	(-32.9 to 40.6)	(-55.0 to -6.8)
**World Bank low income**						
Total	14,800	2,229.4	6,280	2,105.7	135.6	5.9
	(10,700 to 19,100)	(1,637.2 to 2,840.5)	(4,200 to 8,890)	(1,442.6 to 2,911.2)	(63.3 to 268.8)	(-26.7 to 62.7)
Male	10,100	3,115.6	4,120	2,904.1	145.9	7.3
	(7,170 to 13,300)	(2,233.6 to 4,074.7)	(2,250 to 6,410)	(1,631.8 to 4,423.0)	(47.3 to 383.5)	(-34.2 to 105.7)
Female	4,680	1,375.5	2,160	1,354.1	116.1	1.6
	(2,670 to 7,170)	(808.7 to 2,095.3)	(1,130 to 3,810)	(727.3 to 2,335.5)	(24.7 to 344.4)	(-40.4 to 98.7)
**World Bank lower-middle income**						
Total	30,300	978.0	17,300	1,053.2	74.6	-7.1
	(21,800 to 37,800)	(706.9 to 1,221.1)	(13,000 to 22,200)	(800.3 to 1,330.6)	(31.1 to 148.5)	(-31.2 to 30.6)
Male	22,800.0	1,460.8	11,900	1,440.9	91.1	1.4
	(14,900 to 29,400)	(953.1 to 1,880.1)	(8,000 to 16,000)	(993.9 to 1,900.3)	(33.2 to 194.7)	(-29.4 to 54.1)
Female	7,500.0	494.0	5,420.0	649.2	38.2	-23.9
	(4,220 to 10,200)	(278.1 to 668.2)	(3,370 to 7,600)	(413.2 to 896.0)	(-12.3 to 131.3)	(-51.7 to 24.9)
**World Bank upper-middle income**						
Total	22,800	764.4	35,800	1,629.3	-36.4	-53.1
	(19,500 to 26,200)	(653.4 to 877.8)	(26,100 to 44,900)	(1,196.1 to 2,055.0)	(-50.4 to -4.5)	(-63.4 to -29.7)
Male	17,000	1,125.8	26,300	2,365.2	-35.3	-52.4
	(13,800 to 19,800)	(912.8 to 1,310.4)	(17,500 to 34,300)	(1,568.1 to 3,111.5)	(-50.5 to 4.9)	(-63.7 to -22.6)
Female	5,750	393.4	9,490	883.6	-39.4	-55.5
	(4,370 to 6,910)	(294.4 to 471.8)	(6,030 to 13,500)	(565.9 to 1,243.7)	(-57.8 to -0.8)	(-68.6 to -28.5)
**World Bank high income**						
Total	7,360	495.3	15,400	1,255.9	-52.4	-60.6
	(6,700 to 8,180)	(457.4 to 541.8)	(14,400 to 16,500)	(1,177.9 to 1,336.9)	(-54.5 to -49.5)	(-62.2 to -58.4)
Male	5,400	726.5	11,300	1,857.5	-52.4	-60.9
	(4,940 to 5,930)	(670.8 to 788.4)	(10,700 to 12,100)	(1,753.3 to 1,980.8)	(-54.9 to -49.1)	(-63.0 to -58.1)
Female	1,960	256.6	4,100	659.1	-52.3	-61.1
	(1,750 to 2,230)	(233.6 to 285.4)	(3,730 to 4,480)	(603.9 to 713.7)	(-55.0 to -48.9)	(-63.2 to -58.3)
**Andean Latin America**						
Total	764	1,140.1	445	1,204.5	71.4	-5.4
	(675 to 902)	(1,008.0 to 1,347.4)	(378 to 514)	(1,029.6 to 1,387.4)	(48.0 to 118.2)	(-18.0 to 20.0)
Male	577	1,723.9	322	1,797.9	79.0	-4.1
	(489 to 724)	(1,461.2 to 2,169.5)	(258 to 397)	(1,456.0 to 2,203.6)	(49.3 to 150.8)	(-19.0 to 33.7)
Female	187	569.2	123	631.2	51.6	-9.8
	(153 to 236)	(463.5 to 719.0)	(95.5 to 152)	(497.1 to 768.3)	(24.5 to 122.3)	(-25.8 to 29.7)
**Australasia**						
Total	115	339.7	239	1,142.6	-51.8	-70.3
	(103 to 128)	(305.7 to 373.8)	(223 to 253)	(1,072.8 to 1,210.8)	(-55.1 to -48.5)	(-72.3 to -68.4)
Male	81.0	484.9	167	1,592.1	-51.3	-69.5
	(72.5 to 89.6)	(431.9 to 533.7)	(156 to 177)	(1,497.5 to 1,689.7)	(-55.4 to -47.4)	(-72.1 to -66.9)
Female	33.9	195.2	72.2	692.4	-53.0	-71.8
	(29.3 to 39.4)	(174.0 to 221.7)	(65.4 to 78.5)	(633.1 to 749.0)	(-57.4 to -48.4)	(-74.3 to -68.9)
**Caribbean**						
Total	655	1,345.5	540	1,478.0	21.4	-9.0
	(508 to 830)	(1,037.8 to 1,713.9)	(437 to 655)	(1,207.8 to 1,773.0)	(-1.0 to 45.2)	(-26.0 to 9.5)
Male	490	2,018.0	385	2,171.0	27.3	-7.0
	(369 to 638)	(1,509.4 to 2,638.8)	(310 to 492)	(1,769.7 to 2,741.4)	(2.7 to 57.0)	(-24.8 to 15.5)
Female	165	691.8	154	814.6	6.8	-15.1
	(97.0 to 245)	(397.9 to 1,042.0)	(101 to 219)	(545.7 to 1,138.2)	(-18.2 to 47.2)	(-36.8 to 19.6)
**Central Asia**						
Total	566	562.1	683	991.6	-17.2	-43.3
	(508 to 624)	(504.4 to 620.7)	(613 to 755)	(889.8 to 1,097.5)	(-26.0 to -6.4)	(-49.2 to -36.0)
Male	429	862.5	513	1,540.4	-16.4	-44.0
	(384 to 475)	(772.8 to 954.4)	(451 to 580)	(1,360.2 to 1,744.2)	(-27.5 to -2.6)	(-51.3 to -35.0)
Female	137	270.1	170	473.5	-19.7	-42.9
	(119 to 157)	(236.3 to 310.1)	(146 to 195)	(407.6 to 542.4)	(-32.8 to -2.4)	(-52.0 to -30.2)
**Central Europe**						
Total	467	369.9	1,470	1,139.1	-68.2	-67.5
	(423 to 522)	(339.8 to 404.3)	(1,380 to 1,570)	(1,066.9 to 1,215.3)	(-70.1 to -66.0)	(-69.2 to -65.3)
Male	353	564.0	1,120	1,754.2	-68.5	-67.8
	(319 to 389)	(516.4 to 617.4)	(1,040 to 1,200)	(1,631.0 to 1,872.6)	(-70.5 to -65.7)	(-69.8 to -65.0)
Female	115	174.9	349	537.0	-67.2	-67.4
	(101 to 130)	(158.6 to 195.6)	(320 to 384)	(494.9 to 587.8)	(-69.9 to -64.0)	(-69.8 to -63.9)
**Central Latin America**						
Total	2,290	849.8	2,240	1,437.3	2.4	-40.9

	(2,140 to 2,490)	(792.2 to 923.4)	(2,090 to 2,390)	(1,336.5 to 1,535.7)	(-6.1 to 14.5)	(-45.7 to -34.1)
Male	1,820	1,377.7	1,710	2,271.3	6.1	-39.3
	(1,670 to 1,980)	(1,271.6 to 1,505.5)	(1,570 to 1,840)	(2,082.4 to 2,451.0)	(-4.3 to 21.6)	(-45.2 to -30.7)
Female	477	353.9	527	648.1	-9.5	-45.4
	(437 to 521)	(323.9 to 385.5)	(472 to 578)	(578.8 to 711.2)	(-19.5 to 4.2)	(-51.4 to -37.8)
**Central sub-Saharan Africa**						
Total	6,630	4,604.0	2,080	3,656.3	218.9	25.9
	(4,560 to 8,680)	(3,141.8 to 5,975.7)	(1,120 to 3,100)	(1,988.6 to 5,326.5)	(115.4 to 385.4)	(-13.5 to 91.0)
Male	4,230	6,003.8	1,320	4,855.7	220.0	23.6
	(2,740 to 5,780)	(3,888.5 to 8,182.6)	(680 to 2,190)	(2,525.6 to 7,933.0)	(96.2 to 451.9)	(-22.3 to 112.7)
Female	2,400	3,233.6	757	2,500.4	217.0	29.3
	(1,200 to 4,000)	(1,610.2 to 5,374.0)	(277 to 1,410)	(943.8 to 4,633.5)	(82.7 to 524.5)	(-24.2 to 146.0)
**East Asia**						
Total	8,890	520.8	22,300	1,754.9	-60.0	-70.3
	(7,140 to 10,700)	(424.9 to 626.7)	(14,400 to 29,900)	(1,136.4 to 2,375.1)	(-72.3 to -28.7)	(-79.3 to -46.9)
Male	6,370	727.6	16,300	2,485.3	-61.0	-70.7
	(4,690 to 8,050)	(544.7 to 918.9)	(8,510 to 23,100)	(1,291.0 to 3,556.8)	(-74.7 to -16.4)	(-80.6 to -36.8)
Female	2,530	303.2	5,940	982.4	-57.5	-69.1
	(1,900 to 3,100)	(225.9 to 370.2)	(3,310 to 9,230)	(549.9 to 1,516.9)	(-73.9 to -15.4)	(-80.7 to -38.2)
**Eastern Europe**						
Total	1,250	572.8	3,460	1,488.5	-63.8	-61.5
	(1,100 to 1,400)	(510.0 to 637.3)	(3,170 to 3,790)	(1,359.6 to 1,632.0)	(-68.2 to -58.4)	(-65.8 to -56.1)
Male	932	886.2	2,620	2,334.8	-64.4	-62.0
	(819 to 1,050)	(783.0 to 998.5)	(2,360 to 2,890)	(2,105.3 to 2,574.8)	(-69.2 to -58.7)	(-67.2 to -56.2)
Female	319	275.9	842	692.2	-62.1	-60.1
	(268 to 376)	(233.8 to 320.0)	(724 to 976)	(595.0 to 800.0)	(-67.8 to -56.0)	(-66.4 to -52.9)
**Eastern sub-Saharan Africa**						
Total	8,110	1,934.1	3,710	1,955.4	118.6	-1.1
	(5,870 to 10,600)	(1,408.5 to 2,559.6)	(2,610 to 5,130)	(1,377.8 to 2,644.5)	(44.4 to 247.4)	(-34.1 to 57.6)
Male	5,970	2,919.6	2,490	2,768.7	139.9	5.4
	(4,060 to 8,320)	(2,015.1 to 4,085.3)	(1,350 to 3,800)	(1,554.2 to 4,187.0)	(40.0 to 369.3)	(-36.4 to 100.8)
Female	2,140	999.4	1,220	1,191.3	75.2	-16.1
	(1,240 to 3,200)	(583.7 to 1,490.7)	(687 to 1,910)	(685.6 to 1,813.7)	(-8.1 to 268.3)	(-55.1 to 70.6)
**High-income Asia Pacific**						
Total	483	199.6	2,020	1,146.7	-76.1	-82.6
	(412 to 572)	(172.1 to 240.8)	(1,810 to 2,230)	(1,031.8 to 1,264.8)	(-78.9 to -69.8)	(-84.5 to -77.3)
Male	335	289.9	1,490	1,691.0	-77.5	-82.9
	(286 to 407)	(247.9 to 359.9)	(1,280 to 1,690)	(1,447.7 to 1,932.6)	(-80.5 to -68.5)	(-85.1 to -75.2)
Female	148	107.9	530	603.9	-72.1	-82.1
	(121 to 180)	(88.4 to 134.3)	(459 to 600)	(514.5 to 682.4)	(-76.1 to -63.7)	(-84.9 to -74.9)
**High-income North America**						
Total	3,020	765.1	3,670	1,269.7	-17.8	-39.7
	(2,720 to 3,330)	(696.4 to 831.7)	(3,430 to 3,940)	(1,190.6 to 1,356.1)	(-22.8 to -12.6)	(-43.5 to -35.6)
Male	2,100	1,067.0	2,500	1,744.7	-15.9	-38.8
	(1,900 to 2,330)	(963.2 to 1,166.2)	(2,350 to 2,670)	(1,643.0 to 1,860.7)	(-22.1 to -10.3)	(-43.4 to -34.4)
Female	916	461.2	1,170	800.7	-21.7	-42.4
	(822 to 1,040)	(421.0 to 512.0)	(1,050 to 1,280)	(726.6 to 870.2)	(-27.5 to -14.5)	(-47.0 to -37.1)
**North Africa and Middle East**						
Total	6,480	1,014.7	6,170	1,813.9	5.0	-44.1
	(5,080 to 7,860)	(794.0 to 1,231.4)	(4,130 to 8,350)	(1,250.6 to 2,376.4)	(-22.6 to 59.8)	(-58.4 to -15.9)
Male	4,950	1,485.6	4,280	2,521.8	15.4	-41.1
	(3,560 to 6,110)	(1,074.5 to 1,838.9)	(2,510 to 6,210)	(1,534.1 to 3,597.9)	(-21.1 to 98.7)	(-58.8 to -1.3)
Female	1,540	506.9	1,890	1,073.1	-18.7	-52.8
	(1,080 to 1,970)	(359.3 to 648.4)	(960 to 2,870)	(577.8 to 1,606.2)	(-48.8 to 64.0)	(-70.0 to -8.2)
**Oceania**						
Total	139	969.1	66.7	1,045.4	108.5	-7.3
	(102 to 184)	(716.4 to 1,276.4)	(48.6 to 87.7)	(774.4 to 1,344.4)	(46.8 to 211.5)	(-34.1 to 36.0)
Male	90.3	1,240.4	44.7	1,389.9	102.0	-10.8
	(62.0 to 135)	(856.9 to 1,805.2)	(27.2 to 67.7)	(885.7 to 2,033.5)	(26.0 to 235.7)	(-42.8 to 44.7)
Female	48.7	685.2	22.0	679.8	121.8	0.8
	(29.5 to 69.1)	(427.0 to 967.4)	(12.6 to 36.0)	(400.9 to 1,097.1)	(27.7 to 281.4)	(-41.0 to 67.5)
**South Asia**						
Total	15,200	786.7	8,680	829.0	75.0	-5.1
	(10,100 to 19,500)	(524.0 to 1,007.0)	(6,750 to 11,000)	(646.0 to 1,041.7)	(23.7 to 143.5)	(-33.7 to 32.3)
Male	12,000	1,216.5	6,140	1,141.9	95.8	6.5
	(7,640 to 15,800)	(773.0 to 1,588.3)	(4,230 to 8,040)	(806.1 to 1,493.7)	(29.7 to 193.8)	(-30.1 to 58.0)
Female	3,160	345.3	2,540	489.7	24.6	-29.5
	(1,800 to 4,390)	(196.0 to 476.5)	(1,710 to 3,520)	(338.9 to 669.9)	(-19.7 to 115.4)	(-53.9 to 18.8)
**Southeast Asia**						
Total	7,260	973.7	5,890	1,258.4	23.1	-22.6
	(5,520 to 8,820)	(737.6 to 1,180.5)	(4,130 to 7,450)	(897.4 to 1,580.7)	(-6.3 to 82.1)	(-40.1 to 12.2)
Male	5,370	1,434.4	4,340	1,879.5	23.7	-23.7
	(4,100 to 6,820)	(1,096.0 to 1,828.5)	(2,830 to 5,820)	(1,260.8 to 2,512.8)	(-11.7 to 109.9)	(-44.8 to 29.4)
Female	1,890	511.7	1,550	664.0	21.5	-22.9
	(1,160 to 2,490)	(310.7 to 676.4)	(910 to 2,250)	(404.4 to 952.2)	(-22.7 to 127.1)	(-50.7 to 42.1)
**Southern Latin America**						
Total	450	601.5	402	816.7	11.8	-26.4
	(411 to 490)	(552.2 to 651.5)	(376 to 430)	(761.5 to 872.7)	(5.2 to 19.0)	(-31.0 to -21.7)
Male	348	951.8	299	1,250.0	16.3	-23.9
	(319 to 377)	(875.1 to 1,030.4)	(278 to 321)	(1,160.0 to 1,345.1)	(7.5 to 24.3)	(-29.7 to -18.6)
Female	102	265.5	103	407.4	-1.3	-34.8
	(91.0 to 114)	(238.6 to 295.4)	(94.5 to 113)	(372.2 to 443.8)	(-8.0 to 7.4)	(-39.0 to -29.6)
**Southern sub-Saharan Africa**						
Total	1,760	1,896.2	1,180	2,308.3	49.2	-17.9
	(1,380 to 2,210)	(1,492.5 to 2,389.6)	(933 to 1,400)	(1,842.4 to 2,764.3)	(10.6 to 98.4)	(-39.2 to 9.6)
Male	1,230	2,733.1	808	3,342.0	52.6	-18.2

	(891 to 1,640)	(1,974.4 to 3,648.8)	(575 to 1,020)	(2,392.1 to 4,184.2)	(9.1 to 111.3)	(-41.7 to 12.7)
Female	522	1,115.3	368	1,369.7	41.9	-18.6
	(357 to 744)	(769.3 to 1,582.2)	(257 to 506)	(970.8 to 1,898.4)	(-11.7 to 131.0)	(-48.9 to 30.2)
**Tropical Latin America**						
Total	2,280	976.0	2,380	1,596.6	-4.3	-38.9
	(2,170 to 2,390)	(932.6 to 1,022.3)	(2,190 to 2,580)	(1,469.3 to 1,733.8)	(-12.4 to 3.8)	(-44.1 to -33.7)
Male	1,860	1,624.9	1,810	2,489.0	2.4	-34.7
	(1,770 to 1,950)	(1,545.4 to 1,700.1)	(1,640 to 1,980)	(2,251.6 to 2,728.4)	(-7.9 to 12.7)	(-41.4 to -28.1)
Female	420	359.7	566	744.2	-25.8	-51.7
	(391 to 451)	(334.6 to 385.9)	(509 to 640)	(663.6 to 838.8)	(-34.0 to -16.8)	(-56.9 to -46.0)
**Western Europe**						
Total	1,240	248.8	4,250	1,058.4	-70.8	-76.5
	(1,080 to 1,420)	(223.7 to 279.7)	(3,930 to 4,560)	(988.8 to 1,128.1)	(-73.0 to -68.7)	(-78.0 to -74.9)
Male	916	378.7	3,130	1,578.8	-70.7	-76.0
	(809 to 1,030)	(338.7 to 420.1)	(2,930 to 3,340)	(1,488.0 to 1,677.4)	(-73.0 to -68.6)	(-77.8 to -74.3)
Female	323	119.1	1,120	540.8	-71.1	-78.0
	(270 to 388)	(101.4 to 139.3)	(1,010 to 1,240)	(496.1 to 588.6)	(-73.9 to -68.7)	(-80.2 to -76.0)
**Western sub-Saharan Africa**						
Total	7,240	1,462.0	3,080	1,523.3	135.3	-4.0
	(4,860 to 9,840)	(993.8 to 1,951.0)	(1,920 to 4,370)	(993.0 to 2,133.7)	(54.6 to 273.5)	(-35.3 to 46.5)
Male	4,900	2,086.4	1,880	1,956.9	160.2	6.6
	(3,210 to 6,890)	(1,377.8 to 2,858.6)	(1,060 to 3,060)	(1,143.2 to 3,051.2)	(58.8 to 384.6)	(-32.9 to 90.3)
Female	2,340	887.3	1,190	1,092.7	96.1	-18.8
	(1,210 to 3,530)	(460.2 to 1,297.6)	(609 to 2,040)	(582.9 to 1,818.9)	(4.9 to 295.3)	(-54.2 to 56.1)

95% UI=95% uncertainty interval

Table S11. Number of road injury DALYs (in thousands) and age-standardised road injury DALY rates (per 100 000) in 2023, and percentage changes in age-standardised road injury DALY rates from 1990 to 2023 and from 2010 to 2023, across 204 countries and territories. DALYs=disability-adjusted life-years.

	Number of DALYs (in thousands) in 2023 (95% UI)	Age-standardised DALY rates in 2023, per 100 000 (95% UI)	Percentage change in age-standardised DALY rates from 1990 to 2023, in % (95% UI)
**Afghanistan**	434 (288 to 646)	1,214.3 (819.7 to 1,755.7)	17.7 (-23.4 to 92.4)
**Albania**	9.95 (7.45 to 12.6)	384.3 (286.8 to 493.5)	-52.3 (-63.4 to -32.2)
**Algeria**	446 (323 to 598)	1,003.8 (727.0 to 1,347.0)	-55.1 (-69.3 to -29.4)
**American Samoa**	0.24 (0.18 to 0.31)	494.7 (363.9 to 651.5)	-36.0 (-57.7 to -5.8)
**Andorra**	0.61 (0.49 to 0.72)	680.2 (548.9 to 813.1)	-32.2 (-50.8 to 9.1)
**Angola**	1,390 (888 to 1,920)	3,853.6 (2,486.4 to 5,242.6)	-6.3 (-36.2 to 46.1)
**Antigua and Barbuda**	0.23 (0.2 to 0.26)	229.7 (199.1 to 265.0)	-55.8 (-63.6 to -47.4)
**Argentina**	300 (266 to 333)	595.5 (530.4 to 658.2)	-25.0 (-32.0 to -17.8)
**Armenia**	16.6 (14.4 to 19.1)	555.9 (479.5 to 643.4)	-18.8 (-35.0 to 6.2)
**Australia**	88.2 (78.2 to 98.3)	309.2 (277.4 to 342.4)	-70.4 (-72.9 to -68.1)
**Austria**	26.1 (23.0 to 29.8)	266.0 (236.2 to 299.4)	-77.0 (-79.0 to -74.7)
**Azerbaijan**	40.2 (26.4 to 65.9)	397.8 (258.0 to 644.5)	-23.4 (-49.5 to 35.8)
**Bahamas**	4.12 (3.57 to 4.74)	910.4 (788.8 to 1,048.8)	-29.1 (-40.8 to -12.7)
**Bahrain**	10.3 (7.52 to 15.3)	601.4 (441.8 to 888.7)	-47.3 (-62.5 to -13.8)
**Bangladesh**	539 (343 to 924)	316.8 (203.0 to 533.8)	-20.9 (-50.9 to 36.8)
**Barbados**	1.31 (1.13 to 1.51)	401.2 (344.5 to 462.2)	-41.0 (-50.4 to -28.3)
**Belarus**	43.1 (38.2 to 48.5)	407.7 (362.4 to 458.5)	-66.4 (-71.4 to -59.8)
**Belgium**	39.5 (34.7 to 45.0)	301.5 (267.7 to 338.7)	-76.2 (-78.5 to -73.9)
**Belize**	4.82 (4.17 to 5.60)	1,121.4 (967.5 to 1,306.4)	-7.5 (-23.7 to 14.0)
**Benin**	270 (173 to 373)	1,994.1 (1,319.1 to 2,699.0)	-22.8 (-49.0 to 41.2)
**Bermuda**	0.35 (0.31 to 0.4)	562.7 (488.9 to 635.8)	-43.5 (-52.4 to -31.8)
**Bhutan**	3.60 (1.78 to 5.64)	447.4 (222.4 to 697.4)	-25.2 (-54.5 to 16.5)
**Bolivia**	170 (123 to 219)	1,412.1 (1,025.9 to 1,828.9)	-25.4 (-48.4 to 20.3)
**Bosnia and Herzegovina**	7.51 (5.96 to 9.37)	209.2 (166.7 to 260.9)	-56.3 (-65.2 to -39.6)
**Botswana**	30.6 (21.9 to 41.6)	1,213.6 (867.4 to 1,656.9)	-57.3 (-72.5 to -32.1)
**Brazil**	2,190 (2,090 to 2,300)	965.1 (922.2 to 1,011.4)	-40.2 (-45.2 to -35.3)
**Brunei**	2.89 (2.33 to 3.71)	576.1 (465.4 to 734.7)	-64.3 (-72.7 to -45.8)
**Bulgaria**	29.0 (25.7 to 33.1)	418.4 (376.0 to 469.8)	-59.3 (-63.5 to -54.5)
**Burkina Faso**	297 (182 to 430)	1,288.1 (811.0 to 1,808.8)	-18.5 (-52.1 to 29.2)
**Burundi**	441 (288 to 611)	3,085.5 (2,025.7 to 4,248.5)	-6.4 (-43.0 to 58.6)
**Cabo Verde**	1.87 (1.30 to 2.68)	371.3 (260.7 to 529.9)	-5.4 (-37.6 to 43.6)
**Cambodia**	190 (132 to 254)	1,079.8 (748.9 to 1,441.2)	-56.9 (-71.2 to -22.6)
**Cameroon**	657 (458 to 870)	2,090.7 (1,445.6 to 2,707.8)	-1.4 (-40.5 to 51.7)
**Canada**	136 (122 to 155)	337.5 (303.8 to 376.9)	-67.0 (-69.9 to -63.6)
**Central African Republic**	184 (96.9 to 272)	2,979.6 (1,588.1 to 4,365.0)	-28.8 (-51.9 to 7.8)
**Chad**	261 (164 to 359)	1,537.0 (980.9 to 2,071.0)	-3.8 (-37.5 to 40.3)
**Chile**	124 (114 to 136)	597.8 (550.1 to 649.4)	-32.2 (-38.6 to -25.8)
**China**	8,450 (6,660 to 10,200)	510.3 (408.6 to 617.9)	-71.0 (-79.9 to -46.9)
**Colombia**	481 (452 to 512)	860.1 (809.6 to 915.8)	-30.7 (-35.5 to -25.5)
**Comoros**	6.63 (4.39 to 9.95)	778.5 (518.8 to 1,146.0)	-16.3 (-47.6 to 36.2)
**Congo**	138 (79.9 to 202)	2,306.6 (1,358.9 to 3,386.3)	-26.0 (-50.5 to 20.6)
**Cook Islands**	0.19 (0.14 to 0.24)	1,278.0 (979.8 to 1,632.4)	-45.1 (-61.6 to -14.2)
**Costa Rica**	51.1 (46.7 to 55.7)	895.6 (819.9 to 974.6)	-14.6 (-22.2 to -6.1)
**Croatia**	15.6 (13.8 to 17.4)	371.4 (336.1 to 411.3)	-66.0 (-69.2 to -62.6)
**Cuba**	37.2 (32.1 to 41.9)	294.4 (254.2 to 332.0)	-75.6 (-78.8 to -72.4)
**Cyprus**	6.42 (5.20 to 7.82)	443.9 (358.2 to 541.2)	-63.8 (-73.5 to -44.9)
**Czechia**	35.3 (31.7 to 39.7)	299.7 (272.5 to 332.0)	-63.6 (-66.5 to -60.4)
**Côte d'Ivoire**	412 (282 to 564)	1,219.0 (839.8 to 1,643.3)	4.3 (-31.8 to 63.4)
**Democratic Republic of the Congo**	4,840 (3,310 to 6,350)	5,232.6 (3,565.1 to 6,871.7)	46.7 (1.7 to 118.9)
**Denmark**	11.3 (9.60 to 13.2)	166.9 (145.5 to 192.1)	-79.7 (-82.0 to -77.5)
**Djibouti**	16.5 (11.7 to 23.1)	1,246.2 (893.6 to 1,722.7)	-10.7 (-40.6 to 35.1)
**Dominica**	0.51 (0.38 to 0.66)	686.7 (511.6 to 898.8)	-26.3 (-44.3 to 1.3)
**Dominican Republic**	199 (157 to 234)	1,713.6 (1,352.4 to 2,016.3)	5.5 (-18.5 to 28.7)
**Ecuador**	261 (241 to 281)	1,438.0 (1,330.3 to 1,545.5)	-12.5 (-19.4 to -5.4)
**Egypt**	993 (737 to 1,260)	932.7 (703.8 to 1,182.5)	-16.8 (-38.2 to 11.2)
**El Salvador**	63.1 (48.4 to 88.3)	996.4 (764.5 to 1,385.0)	-45.7 (-59.6 to -14.9)
**Equatorial Guinea**	52.3 (27.2 to 83.3)	3,195.8 (1,656.9 to 5,118.8)	-4.3 (-48.5 to 144.5)
**Eritrea**	179 (128 to 241)	2,442.9 (1,740.1 to 3,243.9)	-1.0 (-39.0 to 66.8)
**Estonia**	3.23 (2.83 to 3.72)	217.2 (192.6 to 246.3)	-85.6 (-87.5 to -83.3)
**Eswatini**	31.9 (22.1 to 43.5)	2,519.0 (1,761.5 to 3,409.2)	-55.1 (-71.7 to -29.0)
**Ethiopia**	1,970 (1,330 to 2,750)	1,846.5 (1,270.3 to 2,571.3)	-7.5 (-43.8 to 67.8)
**Fiji**	5.12 (3.84 to 6.78)	542.3 (407.0 to 717.2)	-19.7 (-43.3 to 14.7)
**Finland**	12.6 (10.9 to 14.3)	197.3 (173.4 to 225.6)	-76.2 (-78.6 to -73.2)
**France**	223 (197 to 253)	303.9 (270.5 to 340.0)	-75.8 (-78.1 to -73.5)
**Gabon**	30.9 (17.5 to 45.6)	1,646.9 (955.8 to 2,442.1)	-28.7 (-52.9 to 13.2)
**Gambia**	25.6 (18.0 to 34.9)	1,046.9 (746.6 to 1,407.3)	-2.2 (-36.5 to 52.6)
**Georgia**	30.5 (26.6 to 34.8)	832.3 (721.6 to 952.1)	19.1 (-5.7 to 55.2)
**Germany**	197 (171 to 230)	205.9 (181.6 to 234.9)	-79.4 (-81.3 to -77.6)
**Ghana**	469 (313 to 650)	1,493.1 (1,007.1 to 2,081.6)	24.5 (-23.6 to 102.2)
**Greece**	57.6 (50.0 to 66.1)	491.0 (435.8 to 549.7)	-62.3 (-65.6 to -59.4)
**Greenland**	0.22 (0.14 to 0.35)	433.6 (266.9 to 693.3)	-52.1 (-76.0 to -3.2)
**Grenada**	0.39 (0.33 to 0.46)	324.3 (274.7 to 383.5)	-57.3 (-64.5 to -45.6)
**Guam**	0.8 (0.7 to 0.92)	505.5 (436.6 to 580.7)	-51.2 (-60.2 to -39.8)
**Guatemala**	159 (134 to 185)	939.7 (801.8 to 1,082.2)	12.3 (-5.5 to 32.4)
**Guinea**	184 (120 to 248)	1,413.0 (939.4 to 1,881.3)	-27.1 (-53.0 to 23.9)
**Guinea-Bissau**	19.8 (13.7 to 26.6)	996.4 (706.2 to 1,327.6)	-28.5 (-55.0 to 17.6)
**Guyana**	7.44 (6.23 to 9.31)	865.2 (726.2 to 1,081.5)	-12.3 (-31.0 to 20.7)
**Haiti**	336 (190 to 488)	2,379.7 (1,342.1 to 3,463.0)	-13.2 (-40.6 to 46.1)

**Honduras**	116 (81.9 to 160)	1,109.4 (785.4 to 1,524.8)	-3.3 (-30.8 to 33.9)
**Hungary**	35.1 (31.4 to 39.3)	321.7 (288.5 to 358.9)	-74.5 (-76.9 to -71.6)
**Iceland**	0.77 (0.67 to 0.89)	187.7 (163.1 to 214.6)	-75.0 (-78.2 to -71.4)
**India**	12,500 (8,280 to 16,100)	831.1 (549.7 to 1,071.9)	-9.0 (-36.3 to 26.5)
**Indonesia**	2,830 (2,130 to 3,700)	947.6 (713.6 to 1,233.5)	1.4 (-32.7 to 63.3)
**Iran**	978 (746 to 1,220)	1,128.1 (855.3 to 1,411.4)	-62.3 (-73.1 to -35.6)
**Iraq**	452 (292 to 665)	1,024.6 (663.4 to 1,502.9)	-17.1 (-50.0 to 49.7)
**Ireland**	9.05 (7.81 to 10.4)	151.3 (132.3 to 174.0)	-82.5 (-84.3 to -80.5)
**Israel**	27.8 (24.7 to 31.2)	273.4 (243.4 to 304.6)	-62.7 (-66.2 to -58.9)
**Italy**	233 (200 to 267)	336.4 (294.6 to 380.9)	-69.0 (-71.9 to -66.0)
**Jamaica**	10.0 (8.35 to 12.3)	321.4 (269.1 to 394.7)	43.6 (15.3 to 84.2)
**Japan**	243 (210 to 286)	143.5 (125.7 to 165.1)	-80.9 (-82.7 to -79.1)
**Jordan**	81.5 (64.0 to 101)	625.5 (491.3 to 778.5)	-61.8 (-72.7 to -40.3)
**Kazakhstan**	147 (135 to 161)	730.3 (667.6 to 794.8)	-55.4 (-60.1 to -49.5)
**Kenya**	654 (443 to 948)	1,371.2 (923.2 to 1,982.1)	17.9 (-25.7 to 70.1)
**Kiribati**	0.67 (0.46 to 0.93)	509.5 (348.9 to 696.6)	0.6 (-34.4 to 49.8)
**Kuwait**	27.3 (25.1 to 29.6)	544.5 (502.0 to 589.6)	-59.3 (-62.2 to -55.7)
**Kyrgyzstan**	49.8 (44.6 to 56.1)	696.9 (623.9 to 787.3)	-15.7 (-27.9 to -0.9)
**Laos**	95.1 (63.7 to 124)	1,238.0 (834.4 to 1,615.9)	-1.6 (-35.1 to 93.1)
**Latvia**	8.91 (7.93 to 9.94)	429.8 (385.9 to 477.4)	-71.7 (-75.6 to -67.4)
**Lebanon**	30.2 (22.4 to 38.4)	524.1 (386.5 to 665.9)	-19.6 (-47.9 to 54.7)
**Lesotho**	37.4 (25.7 to 48.5)	1,971.1 (1,354.9 to 2,553.2)	-38.2 (-63.1 to -4.0)
**Liberia**	85.2 (60.9 to 117)	1,485.7 (1,063.4 to 2,043.5)	2.9 (-33.5 to 67.5)
**Libya**	78.2 (58.1 to 101)	1,028.3 (762.0 to 1,323.1)	-46.1 (-63.7 to -20.9)
**Lithuania**	11.0 (9.74 to 12.2)	354.1 (316.7 to 391.3)	-77.6 (-80.3 to -74.4)
**Luxembourg**	1.73 (1.48 to 2.01)	236.6 (207.0 to 272.2)	-82.3 (-84.0 to -80.2)
**Madagascar**	579 (408 to 748)	2,134.7 (1,521.0 to 2,755.8)	3.0 (-28.5 to 62.8)
**Malawi**	413 (270 to 592)	2,272.0 (1,500.1 to 3,244.9)	-5.5 (-43.2 to 52.8)
**Malaysia**	316 (265 to 379)	864.7 (726.3 to 1,037.3)	-41.7 (-55.5 to -16.7)
**Maldives**	0.99 (0.7 to 1.36)	175.7 (124.6 to 236.7)	-48.1 (-66.7 to 8.1)
**Mali**	362 (238 to 505)	1,662.9 (1,107.9 to 2,259.8)	-7.4 (-41.0 to 50.9)
**Malta**	0.9 (0.74 to 1.07)	147.2 (124.4 to 171.2)	-69.2 (-73.3 to -64.7)
**Marshall Islands**	0.37 (0.26 to 0.5)	924.0 (656.0 to 1,251.7)	-16.2 (-42.2 to 26.5)
**Mauritania**	35.0 (21.1 to 54.4)	840.7 (507.9 to 1,295.7)	-24.2 (-50.3 to 30.0)
**Mauritius**	8.65 (7.70 to 9.56)	607.5 (538.4 to 673.4)	-34.3 (-43.4 to -23.7)
**Mexico**	1,080 (974 to 1,200)	786.7 (711.7 to 874.4)	-49.0 (-55.6 to -41.1)
**Micronesia (Federated States of)**	0.62 (0.46 to 0.82)	595.2 (445.9 to 779.8)	-29.9 (-52.7 to 2.1)
**Monaco**	0.14 (0.11 to 0.17)	365.1 (277.9 to 451.7)	-49.4 (-67.3 to -23.0)
**Mongolia**	28.4 (21.3 to 34.8)	813.3 (611.3 to 991.9)	-37.0 (-61.4 to -5.0)
**Montenegro**	2.55 (1.93 to 3.19)	358.7 (269.4 to 452.2)	-48.5 (-62.3 to -27.9)
**Morocco**	514 (374 to 683)	1,356.2 (981.0 to 1,795.6)	-42.3 (-61.1 to -4.5)
**Mozambique**	286 (201 to 395)	934.0 (664.4 to 1,289.6)	-36.5 (-57.6 to 1.0)
**Myanmar**	629 (377 to 918)	1,064.2 (638.0 to 1,556.0)	-25.4 (-52.7 to 22.8)
**Namibia**	75.9 (56.6 to 102)	2,409.9 (1,799.3 to 3,212.8)	-44.6 (-62.3 to -14.6)
**Nauru**	0.44 (0.24 to 0.62)	3,577.6 (1,925.2 to 5,038.9)	-12.4 (-43.4 to 53.1)
**Nepal**	174 (124 to 239)	544.2 (389.2 to 743.6)	2.7 (-35.4 to 74.7)
**Netherlands**	42.3 (37.6 to 47.7)	209.2 (190.3 to 235.0)	-69.2 (-71.7 to -66.6)
**New Zealand**	26.7 (23.7 to 29.6)	498.4 (448.4 to 545.7)	-69.2 (-71.3 to -66.7)
**Nicaragua**	60.7 (47.6 to 83.5)	865.2 (684.1 to 1,193.2)	-33.4 (-49.0 to 6.0)
**Niger**	506 (304 to 733)	1,688.9 (1,065.9 to 2,414.3)	19.7 (-25.2 to 98.3)
**Nigeria**	3,140 (1,780 to 4,650)	1,337.9 (776.9 to 1,933.9)	-10.9 (-44.5 to 44.7)
**Niue**	0.01 (0.01 to 0.01)	644.4 (406.1 to 887.7)	-40.3 (-61.4 to -12.5)
**North Korea**	310 (239 to 391)	1,043.8 (806.4 to 1,333.7)	-18.9 (-43.0 to 26.1)
**North Macedonia**	5.85 (4.72 to 7.40)	293.2 (233.2 to 372.6)	-51.1 (-60.6 to -33.5)
**Northern Mariana Islands**	0.38 (0.29 to 0.49)	799.9 (611.3 to 1,017.5)	-46.4 (-65.0 to -20.4)
**Norway**	9.92 (8.52 to 11.5)	167.5 (146.4 to 193.8)	-75.3 (-78.6 to -71.8)
**Oman**	70.6 (52.1 to 98.5)	1,376.9 (1,022.1 to 1,910.4)	-65.3 (-74.9 to -46.6)
**Pakistan**	1,940 (1,130 to 2,660)	862.7 (503.6 to 1,188.8)	38.6 (-5.3 to 101.4)
**Palau**	0.17 (0.12 to 0.21)	923.1 (685.7 to 1,153.2)	-28.4 (-51.5 to 19.9)
**Palestine**	18.3 (14.4 to 24.2)	367.8 (289.0 to 490.7)	-62.6 (-75.0 to -39.4)
**Panama**	23.7 (21.5 to 26.0)	550.6 (501.6 to 603.3)	-55.6 (-60.4 to -49.5)
**Papua New Guinea**	114 (81.5 to 153)	1,059.8 (772.1 to 1,417.5)	-8.2 (-35.0 to 37.6)
**Paraguay**	86.2 (69.6 to 102)	1,312.9 (1,063.9 to 1,554.5)	47.5 (26.9 to 72.0)
**Peru**	333 (262 to 461)	913.6 (720.6 to 1,266.5)	13.6 (-8.9 to 71.4)
**Philippines**	707 (525 to 880)	605.4 (453.2 to 753.6)	1.3 (-25.1 to 40.0)
**Poland**	157 (141 to 176)	370.9 (333.5 to 413.5)	-72.8 (-75.8 to -69.1)
**Portugal**	44.5 (39.1 to 50.5)	369.2 (326.7 to 416.9)	-81.9 (-83.8 to -80.1)
**Puerto Rico**	15.4 (14.1 to 17.2)	474.8 (432.1 to 529.5)	-53.7 (-58.1 to -47.8)
**Qatar**	17.5 (13.8 to 22.9)	582.9 (457.7 to 787.1)	-65.7 (-74.7 to -43.0)
**Republic of Moldova**	16.1 (13.9 to 18.5)	431.5 (372.2 to 499.8)	-74.1 (-77.9 to -69.1)
**Romania**	103 (90.4 to 115)	492.2 (436.3 to 549.8)	-61.0 (-65.3 to -56.4)
**Russia**	920 (815 to 1,030)	585.3 (521.1 to 649.0)	-64.3 (-68.4 to -60.2)
**Rwanda**	482 (313 to 703)	3,575.6 (2,322.7 to 5,311.6)	-32.4 (-58.2 to 18.4)
**Saint Kitts and Nevis**	0.23 (0.2 to 0.27)	454.1 (394.1 to 528.9)	-49.8 (-57.0 to -39.8)
**Saint Lucia**	1.05 (0.91 to 1.23)	553.9 (473.2 to 645.8)	-53.9 (-62.0 to -43.2)
**Saint Vincent and the Grenadines**	0.34 (0.29 to 0.39)	279.1 (241.4 to 320.2)	-51.6 (-61.1 to -40.3)
**Samoa**	1.40 (1.01 to 1.82)	684.8 (497.7 to 892.2)	-25.7 (-51.2 to 9.5)
**San Marino**	0.16 (0.13 to 0.19)	462.0 (364.2 to 572.3)	-49.8 (-65.4 to -22.4)
**Saudi Arabia**	610 (463 to 735)	1,808.0 (1,381.3 to 2,162.2)	-43.2 (-58.5 to -17.9)
**Senegal**	197 (142 to 265)	1,091.6 (770.0 to 1,456.7)	-15.4 (-46.4 to 36.1)
**Serbia**	32.9 (27.3 to 39.6)	321.1 (264.2 to 388.0)	-63.7 (-72.1 to -50.5)
**Seychelles**	0.63 (0.43 to 0.89)	548.4 (372.3 to 776.7)	-19.1 (-47.5 to 30.3)
**Sierra Leone**	134 (96.0 to 180)	1,719.2 (1,247.9 to 2,247.4)	42.1 (-4.2 to 118.9)
**Singapore**	9.68 (8.31 to 11.3)	156.0 (137.2 to 179.2)	-76.0 (-79.0 to -73.4)
**Slovakia**	20.3 (16.3 to 25.0)	345.4 (277.5 to 427.3)	-66.2 (-73.2 to -51.7)
**Slovenia**	6.28 (5.49 to 7.17)	262.0 (229.2 to 296.7)	-79.3 (-81.6 to -76.7)

**Solomon Islands**	6.50 (4.47 to 8.78)	853.3 (590.8 to 1,139.6)	-2.8 (-36.0 to 40.7)
**Somalia**	185 (108 to 254)	878.9 (536.0 to 1,187.5)	-5.9 (-42.8 to 46.9)
**South Africa**	1,410 (1,100 to 1,740)	2,025.9 (1,599.8 to 2,504.0)	-9.2 (-33.8 to 18.9)
**South Korea**	227 (186 to 301)	332.5 (270.1 to 463.2)	-85.5 (-88.2 to -75.4)
**South Sudan**	275 (187 to 377)	2,858.7 (1,945.7 to 3,904.6)	13.3 (-23.2 to 75.2)
**Spain**	135 (118 to 154)	251.8 (226.0 to 283.0)	-82.9 (-84.2 to -81.6)
**Sri Lanka**	172 (132 to 215)	710.9 (542.9 to 887.6)	-21.9 (-42.1 to 17.2)
**Sudan**	562 (368 to 765)	1,506.0 (979.1 to 2,049.0)	-35.5 (-58.1 to 8.9)
**Suriname**	5.86 (4.76 to 7.25)	982.9 (793.0 to 1,213.2)	-21.5 (-39.1 to 3.8)
**Sweden**	18.2 (15.8 to 21.1)	150.2 (133.4 to 171.8)	-76.4 (-78.7 to -74.2)
**Switzerland**	15.2 (13.0 to 17.9)	144.7 (124.6 to 167.4)	-84.2 (-85.9 to -82.2)
**Syria**	80.9 (57.2 to 110)	451.6 (321.5 to 609.2)	-53.4 (-71.0 to -18.6)
**São Tomé and Príncipe**	3.38 (2.08 to 5.36)	1,595.2 (973.1 to 2,574.4)	44.5 (-12.0 to 138.9)
**Taiwan**	136 (117 to 168)	514.2 (444.8 to 631.9)	-73.3 (-76.8 to -67.1)
**Tajikistan**	34.4 (25.0 to 45.4)	335.2 (246.3 to 436.5)	-60.5 (-72.0 to -34.5)
**Tanzania**	1,010 (695 to 1,350)	1,682.0 (1,179.8 to 2,228.0)	1.5 (-31.4 to 59.2)
**Thailand**	992 (664 to 1,160)	1,490.0 (956.7 to 1,764.0)	-29.4 (-50.5 to -2.3)
**Timor-Leste**	15.4 (10.8 to 20.5)	1,074.8 (764.5 to 1,420.6)	-14.1 (-41.9 to 45.5)
**Togo**	181 (128 to 238)	2,200.5 (1,589.8 to 2,883.5)	47.8 (-3.4 to 118.3)
**Tokelau**	0.01 (0 to 0.01)	447.7 (303.0 to 608.9)	-36.4 (-57.1 to -6.3)
**Tonga**	0.56 (0.37 to 0.79)	562.9 (379.5 to 797.8)	-34.8 (-58.1 to -5.6)
**Trinidad and Tobago**	8.97 (7.65 to 10.9)	613.7 (520.7 to 746.8)	-35.8 (-45.9 to -20.9)
**Tunisia**	104 (81.5 to 133)	835.9 (656.5 to 1,063.2)	-40.8 (-57.8 to 5.6)
**Turkmenistan**	17.1 (14.8 to 20.4)	302.7 (262.0 to 359.5)	-75.8 (-79.6 to -71.4)
**Tuvalu**	0.21 (0.15 to 0.27)	1,894.7 (1,396.6 to 2,471.4)	-25.1 (-49.0 to 18.3)
**Türkiye**	449 (345 to 569)	506.8 (387.8 to 638.7)	-49.3 (-66.1 to -12.6)
**UK**	125 (110 to 143)	163.8 (146.5 to 183.1)	-75.4 (-77.3 to -73.5)
**USA**	2,880 (2,590 to 3,170)	813.5 (740.8 to 883.4)	-37.3 (-41.4 to -32.9)
**Uganda**	1,250 (872 to 1,650)	2,985.0 (2,102.7 to 3,973.7)	87.4 (15.8 to 195.4)
**Ukraine**	249 (211 to 304)	615.1 (523.0 to 749.3)	-42.7 (-53.1 to -25.6)
**United Arab Emirates**	57.0 (44.8 to 70.7)	552.3 (432.9 to 679.0)	-74.0 (-83.6 to -38.1)
**Uruguay**	25.6 (22.7 to 28.6)	688.4 (609.4 to 762.2)	-15.9 (-25.4 to -5.0)
**Uzbekistan**	201 (175 to 234)	562.6 (489.3 to 652.5)	-23.9 (-34.5 to -10.2)
**Vanuatu**	1.92 (1.30 to 2.60)	645.2 (443.2 to 868.1)	-4.5 (-32.9 to 36.3)
**Venezuela**	262 (224 to 299)	997.7 (857.3 to 1,140.7)	-38.4 (-48.0 to -28.4)
**Viet Nam**	1,290 (811 to 2,020)	1,178.6 (743.4 to 1,848.8)	-31.6 (-56.1 to 14.7)
**Virgin Islands**	0.53 (0.44 to 0.63)	689.3 (558.9 to 832.4)	-41.4 (-55.6 to -24.4)
**Yemen**	459 (280 to 629)	1,356.7 (841.1 to 1,843.6)	-16.3 (-47.5 to 35.7)
**Zambia**	353 (257 to 481)	1,979.5 (1,455.2 to 2,666.5)	-16.9 (-46.0 to 40.2)
**Zimbabwe**	174 (121 to 244)	1,129.8 (796.8 to 1,571.1)	-34.2 (-56.4 to -8.6)

95% UI=95% uncertainty interval

Table S12. Number of road injury deaths and their percentage of all-cause mortality by age group and sex, globally in 2023.

Age	Both sexes		Males		Females	
	Number of deaths in 2023 (95% UI)	Road injury deaths as a percentage of all-cause mortality in 2023 (95% UI)	Number of deaths in 2023 (95% UI)	Road injury deaths as a percentage of all-cause mortality in 2023 (95% UI)	Number of deaths in 2023 (95% UI)	Road injury deaths as a percentage of all-cause mortality in 2023 (95% UI)
**<5 years**	41,600 (27,900 to 59,300)	0.9 (0.6 to 1.3)	25,700 (15,200 to 38,400)	1.0 (0.6 to 1.5)	15,900 (8,610 to 24,000)	0.8 (0.4 to 1.1)
**5-9 years**	41,600 (30,200 to 56,000)	8.9 (6.5 to 11.9)	23,500 (15,200 to 33,500)	9.4 (6.2 to 13.1)	18,100 (10,400 to 26,900)	8.3 (4.9 to 12.0)
**10-14 years**	39,300 (29,600 to 49,200)	9.7 (7.4 to 12.3)	25,300 (17,800 to 33,200)	11.5 (8.1 to 15.0)	14,000 (8,340 to 19,200)	7.6 (4.7 to 10.3)
**15-19 years**	97,600 (77,700 to 117,000)	14.6 (11.8 to 17.6)	72,800 (54,400 to 91,600)	18.9 (14.3 to 23.8)	24,800 (16,000 to 33,500)	8.7 (5.8 to 11.7)
**20-24 years**	123,000 (98,500 to 146,000)	15.4 (12.1 to 18.2)	98,000 (71,600 to 121,000)	20.7 (15.6 to 24.9)	24,900 (16,400 to 32,500)	7.7 (5.0 to 9.9)
**25-29 years**	117,000 (94,000 to 139,000)	13.6 (11.1 to 15.8)	92,800 (71,000 to 112,000)	18.3 (14.5 to 21.9)	24,600 (16,400 to 32,400)	6.9 (4.5 to 9.0)
**30-34 years**	119,000 (96,400 to 143,000)	10.9 (8.7 to 12.8)	95,400 (68,900 to 117,000)	14.9 (10.8 to 17.9)	23,900 (15,300 to 31,000)	5.3 (3.4 to 6.8)
**35-39 years**	112,000 (86,100 to 133,000)	8.9 (6.8 to 10.5)	88,400 (63,900 to 108,000)	11.6 (8.4 to 14.0)	23,700 (15,600 to 30,800)	4.7 (3.1 to 5.9)
**40-44 years**	102,000 (79,100 to 121,000)	6.5 (5.1 to 7.6)	79,000 (57,000 to 96,600)	8.3 (6.0 to 10.0)	23,100 (14,800 to 28,900)	3.8 (2.4 to 4.7)
**45-49 years**	88,500 (67,800 to 107,000)	4.7 (3.7 to 5.6)	67,400 (47,900 to 86,200)	5.8 (4.1 to 7.3)	21,100 (13,500 to 27,100)	2.9 (1.9 to 3.7)
**50-54 years**	89,300 (67,700 to 108,000)	3.5 (2.7 to 4.1)	66,800 (47,200 to 83,100)	4.2 (2.9 to 5.2)	22,500 (13,900 to 29,100)	2.3 (1.4 to 2.9)
**55-59 years**	83,800 (61,500 to 99,300)	2.5 (1.9 to 2.9)	60,900 (41,600 to 76,100)	2.9 (1.9 to 3.7)	22,900 (15,400 to 29,600)	1.8 (1.1 to 2.2)
**60-64 years**	74,700 (56,800 to 90,300)	1.7 (1.3 to 2.0)	53,700 (36,300 to 66,000)	2.0 (1.4 to 2.5)	20,900 (12,800 to 27,500)	1.2 (0.8 to 1.6)
**65-69 years**	70,100 (52,800 to 84,200)	1.3 (1.0 to 1.6)	48,700 (31,900 to 61,600)	1.5 (1.0 to 1.9)	21,400 (12,800 to 27,800)	1.0 (0.6 to 1.3)
**70-74 years**	52,700 (39,800 to 64,200)	0.8 (0.6 to 1.0)	35,100 (24,100 to 44,600)	1.0 (0.7 to 1.2)	17,500 (11,500 to 22,400)	0.7 (0.4 to 0.8)
**75-79 years**	38,900 (30,600 to 46,300)	0.6 (0.5 to 0.7)	25,200 (17,400 to 32,400)	0.7 (0.5 to 0.9)	13,600 (8,970 to 17,600)	0.5 (0.3 to 0.6)
**80-84 years**	26,600 (20,800 to 31,800)	0.4 (0.3 to 0.5)	17,800 (12,200 to 22,000)	0.5 (0.4 to 0.7)	8,740 (6,270 to 11,300)	0.3 (0.2 to 0.3)
**85-89 years**	16,000 (12,600 to 19,400)	0.3 (0.2 to 0.3)	9,830 (6,780 to 12,300)	0.4 (0.3 to 0.5)	6,150 (4,300 to 7,970)	0.2 (0.1 to 0.2)
**90-94 years**	6,490 (4,950 to 8,080)	0.2 (0.1 to 0.2)	3,930 (2,800 to 5,140)	0.3 (0.2 to 0.3)	2,560 (1,760 to 3,470)	0.1 (0.1 to 0.1)
**95+ years**	2,280 (1,700 to 2,910)	0.1 (0.1 to 0.2)	1,140 (820 to 1,500)	0.2 (0.2 to 0.3)	1,140 (765 to 1,590)	0.1 (0.1 to 0.1)

95% UI=95% uncertainty interval

Table S13. Age-standardised nature-of-injury incidence rates (per 100 000 population) and the number of nature-of-injury incident cases and YLDs (in thousands) attributable to each Level 4 road injury cause, and the share of each nature-of-injury category within the corresponding Level 4 road injury cause, globally in 2023. YLDs=years lived with disability.

		Incidence			YLDs	
		Age-standardised incidence rates in 2023, per 100,000 (95% UI)	Number of incident cases in 2023, in thousands (95% UI)	Percentage of all road injury cases in 2023 (95% UI)	Number of YLDs in 2023, in thousands (95% UI)	Percentage of all road injury YLDs in 2023 (95% UI)
**Amputations**	**Motor vehicles**	2.3 (1.5 to 3.5)	188 (117 to 281)	0.9 (0.6 to 1.4)	161 (105 to 223)	5.4 (4.4 to 6.4)
	**Motorcyclists**	1.5 (1.0 to 2.2)	120 (79.8 to 177)	1.8 (1.3 to 2.6)	127 (84.8 to 174)	7.4 (6.4 to 8.4)
	**Cyclists**	1.4 (0.8 to 2.5)	116 (61.1 to 198)	1.2 (0.6 to 1.9)	71.3 (43.0 to 118)	5.5 (4.1 to 7.3)
	**Pedestrians**	2.4 (1.6 to 3.5)	208 (136 to 296)	1.8 (1.2 to 2.6)	172 (118 to 249)	8.0 (7.1 to 9.1)
	**Others**	0.6 (0.3 to 1.0)	43.0 (22.6 to 74.0)	1.5 (0.9 to 2.6)	41.3 (28.1 to 61.1)	9.8 (7.9 to 12.1)
**Burns**	**Motor vehicles**	1.6 (0.9 to 2.9)	129 (69.5 to 228)	0.7 (0.4 to 1.2)	75.1 (54.1 to 102)	2.5 (2.2 to 2.9)
	**Motorcyclists**	1.4 (0.8 to 2.2)	110 (66.1 to 174)	1.6 (1.0 to 2.5)	108 (75.0 to 143)	6.4 (5.5 to 7.4)
	**Cyclists**	1.0 (0.5 to 1.9)	83.0 (39.7 to 147)	0.8 (0.4 to 1.5)	63.4 (46.9 to 83.4)	4.9 (4.1 to 5.8)
	**Pedestrians**	1.9 (1.1 to 3.2)	157 (88.4 to 255)	1.4 (0.8 to 2.1)	99.4 (70.6 to 132)	4.6 (4.0 to 5.3)
	**Others**	0.3 (0.1 to 0.6)	21.5 (8.11 to 42.4)	0.8 (0.3 to 1.5)	15.2 (10.5 to 20.3)	3.6 (3.2 to 4.1)
**Fractures**	**Motor vehicles**	81.2 (67.3 to 96.6)	6,590 (5,510 to 7,780)	33.4 (29.2 to 37.5)	1,300 (859 to 1,840)	43.5 (38.4 to 48.4)
	**Motorcyclists**	33.4 (27.7 to 41.3)	2,750 (2,280 to 3,380)	41.0 (36.5 to 46.2)	738 (494 to 1,030)	43.3 (37.4 to 48.6)
	**Cyclists**	50.7 (40.7 to 61.1)	4,170 (3,340 to 5,020)	41.5 (35.8 to 48.0)	699 (464 to 1,020)	53.7 (47.9 to 59.0)
	**Pedestrians**	55.1 (45.4 to 65.7)	4,670 (3,880 to 5,550)	40.4 (35.7 to 45.9)	1,130 (758 to 1,610)	52.0 (47.1 to 56.7)
	**Others**	16.0 (11.8 to 20.7)	1,210 (886 to 1,560)	43.3 (36.5 to 50.9)	184 (125 to 263)	43.5 (37.3 to 48.4)
**Head Injuries**	**Motor vehicles**	24.7 (20.0 to 31.1)	2,040 (1,650 to 2,560)	10.3 (8.5 to 12.4)	675 (456 to 899)	22.7 (19.1 to 26.4)
	**Motorcyclists**	11.2 (8.8 to 13.7)	930 (730 to 1,140)	13.9 (11.3 to 16.6)	374 (268 to 515)	22.0 (17.9 to 26.4)
	**Cyclists**	9.9 (7.1 to 13.4)	824 (594 to 1,110)	8.2 (6.3 to 10.5)	260 (181 to 353)	20.1 (15.7 to 24.9)
	**Pedestrians**	14.5 (11.2 to 18.0)	1,240 (952 to 1,520)	10.7 (8.8 to 12.8)	424 (305 to 589)	19.7 (15.4 to 23.8)
	**Others**	3.1 (2.0 to 4.4)	240 (156 to 339)	8.6 (6.5 to 11.0)	89.5 (61.3 to 124)	21.2 (17.0 to 26.1)
**Moderate/ Severe TBI**	**Motor vehicles**	16.2 (12.6 to 20.2)	1,360 (1,050 to 1,690)	6.9 (5.6 to 8.4)	572 (387 to 770)	19.2 (15.7 to 23.0)
	**Motorcyclists**	8.8 (6.6 to 11.0)	734 (549 to 914)	11.0 (8.6 to 13.8)	336 (242 to 466)	19.8 (16.0 to 23.9)
	**Cyclists**	5.3 (3.7 to 7.2)	451 (318 to 603)	4.5 (3.2 to 5.7)	213 (145 to 297)	16.5 (12.4 to 20.9)
	**Pedestrians**	10.7 (8.0 to 13.5)	916 (688 to 1,150)	7.9 (6.5 to 9.6)	377 (268 to 522)	17.5 (13.5 to 21.5)
	**Others**	2.0 (1.2 to 2.7)	159 (99.5 to 219)	5.7 (4.1 to 7.3)	79.7 (53.6 to 112)	18.9 (14.9 to 23.7)
**Minor TBI**	**Motor vehicles**	8.5 (5.6 to 12.1)	685 (450 to 982)	3.5 (2.3 to 5.0)	103 (65.8 to 141)	3.4 (2.9 to 4.0)
	**Motorcyclists**	2.4 (1.5 to 3.7)	196 (121 to 302)	2.9 (1.9 to 4.2)	37.4 (25.3 to 52.4)	2.2 (1.8 to 2.6)
	**Cyclists**	4.6 (2.5 to 7.9)	373 (203 to 640)	3.7 (2.1 to 5.8)	47.1 (31.5 to 65.1)	3.6 (3.0 to 4.3)
	**Pedestrians**	3.8 (2.4 to 5.7)	322 (202 to 470)	2.8 (1.7 to 4.0)	47.2 (31.4 to 64.7)	2.2 (1.8 to 2.6)
	**Others**	1.1 (0.5 to 2.0)	81.5 (36.9 to 149)	2.9 (1.5 to 4.9)	9.82 (6.81 to 14.0)	2.3 (2.0 to 2.8)
**Minor Injuries**	**Motor vehicles**	98.9 (81.2 to 119.4)	7,950 (6,510 to 9,590)	40.2 (35.0 to 44.5)	70.0 (32.8 to 133)	2.3 (1.2 to 4.1)
	**Motorcyclists**	23.7 (17.8 to 30.7)	1,930 (1,450 to 2,490)	28.7 (23.9 to 34.0)	44.1 (20.0 to 85.5)	2.6 (1.4 to 4.4)
	**Cyclists**	45.0 (33.8 to 58.9)	3,670 (2,750 to 4,770)	36.4 (29.9 to 43.0)	28.3 (12.9 to 53.0)	2.2 (1.2 to 3.7)
	**Pedestrians**	41.9 (32.4 to 51.6)	3,500 (2,710 to 4,310)	30.2 (25.1 to 35.6)	41.7 (19.5 to 77.9)	1.9 (1.0 to 3.2)
	**Others**	12.5 (8.5 to 16.9)	929 (637 to 1,260)	33.2 (25.9 to 40.2)	6.52 (3.06 to 12.7)	1.5 (0.8 to 2.6)
**Other Injuries**	**Motor vehicles**	34.8 (28.3 to 43.2)	2,810 (2,290 to 3,490)	14.2 (12.0 to 17.0)	300 (208 to 404)	10.1 (8.9 to 11.6)
	**Motorcyclists**	10.3 (7.9 to 13.1)	848 (651 to 1,070)	12.7 (10.5 to 15.3)	166 (116 to 222)	9.8 (8.6 to 10.9)
	**Cyclists**	14.6 (11.1 to 19.6)	1,190 (908 to 1,600)	11.8 (9.3 to 14.4)	90.2 (63.2 to 125)	7.0 (5.9 to 8.5)
	**Pedestrians**	21.2 (16.9 to 26.3)	1,780 (1,420 to 2,210)	15.4 (13.0 to 18.2)	176 (125 to 235)	8.1 (6.9 to 9.3)
	**Others**	4.6 (3.2 to 6.2)	346 (241 to 469)	12.4 (9.8 to 15.6)	30.6 (21.2 to 41.3)	7.3 (6.2 to 8.6)
**Spinal Injuries**	**Motor vehicles**	0.6 (0.4 to 0.9)	49.8 (33.9 to 72.3)	0.3 (0.2 to 0.4)	398 (282 to 519)	13.5 (10.3 to 16.9)
	**Motorcyclists**	0.2 (0.1 to 0.3)	16.4 (8.13 to 27.9)	0.2 (0.1 to 0.4)	144 (102 to 193)	8.5 (6.3 to 11.2)
	**Cyclists**	0.1 (0.1 to 0.2)	10.1 (4.76 to 18.4)	0.1 (0.0 to 0.2)	85.0 (58.6 to 111)	6.7 (4.6 to 9.0)
	**Pedestrians**	0.2 (0.1 to 0.3)	15.8 (8.41 to 27.7)	0.1 (0.1 to 0.2)	123 (85.0 to 157)	5.8 (4.1 to 7.6)
	**Others**	0.1 (0.0 to 0.1)	5.86 (2.71 to 11.7)	0.2 (0.1 to 0.4)	54.5 (37.6 to 69.3)	13.0 (9.6 to 17.2)

95% UI=95% uncertainty interval

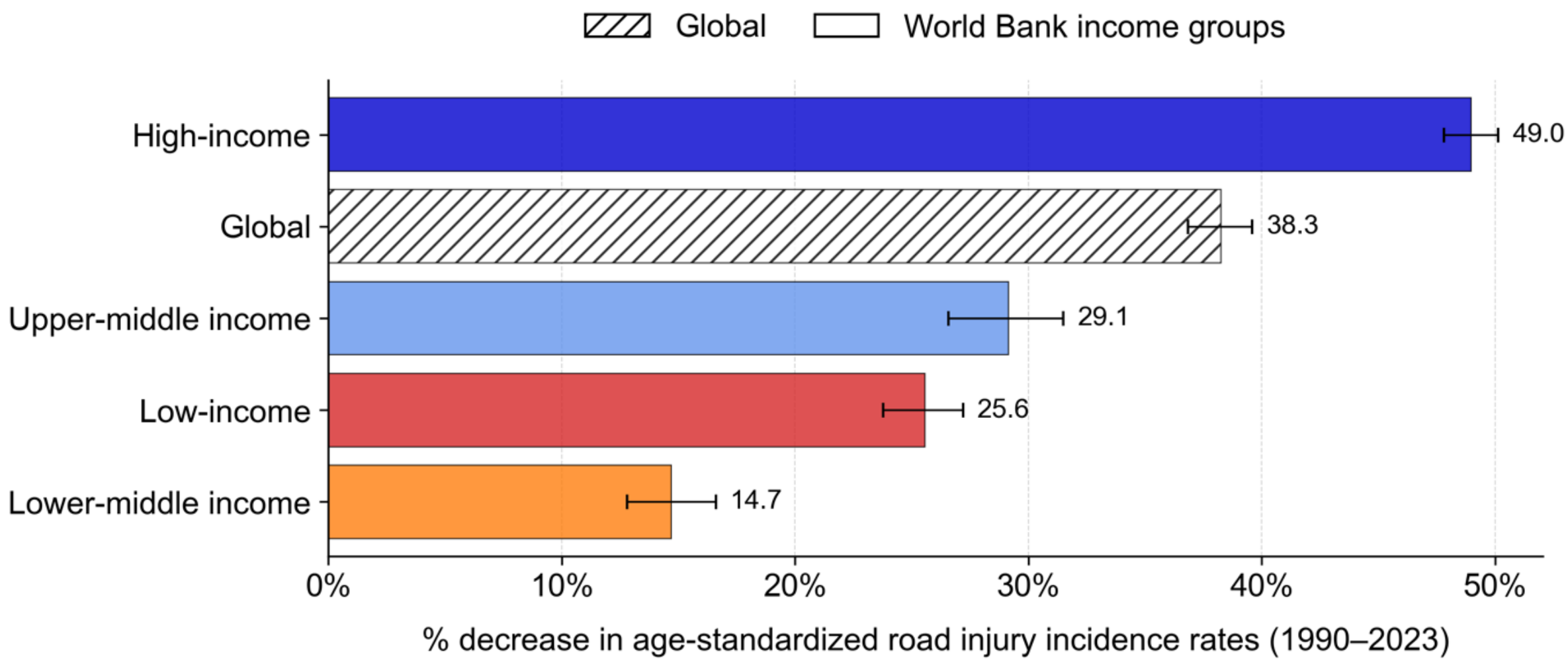

Figure S1. Percentage decrease in age-standardised road injury incidence rates from 1990 to 2023, globally and across the World Bank income groups. Values denote the percentage decrease, and error bars represent the 95% uncertainty interval.

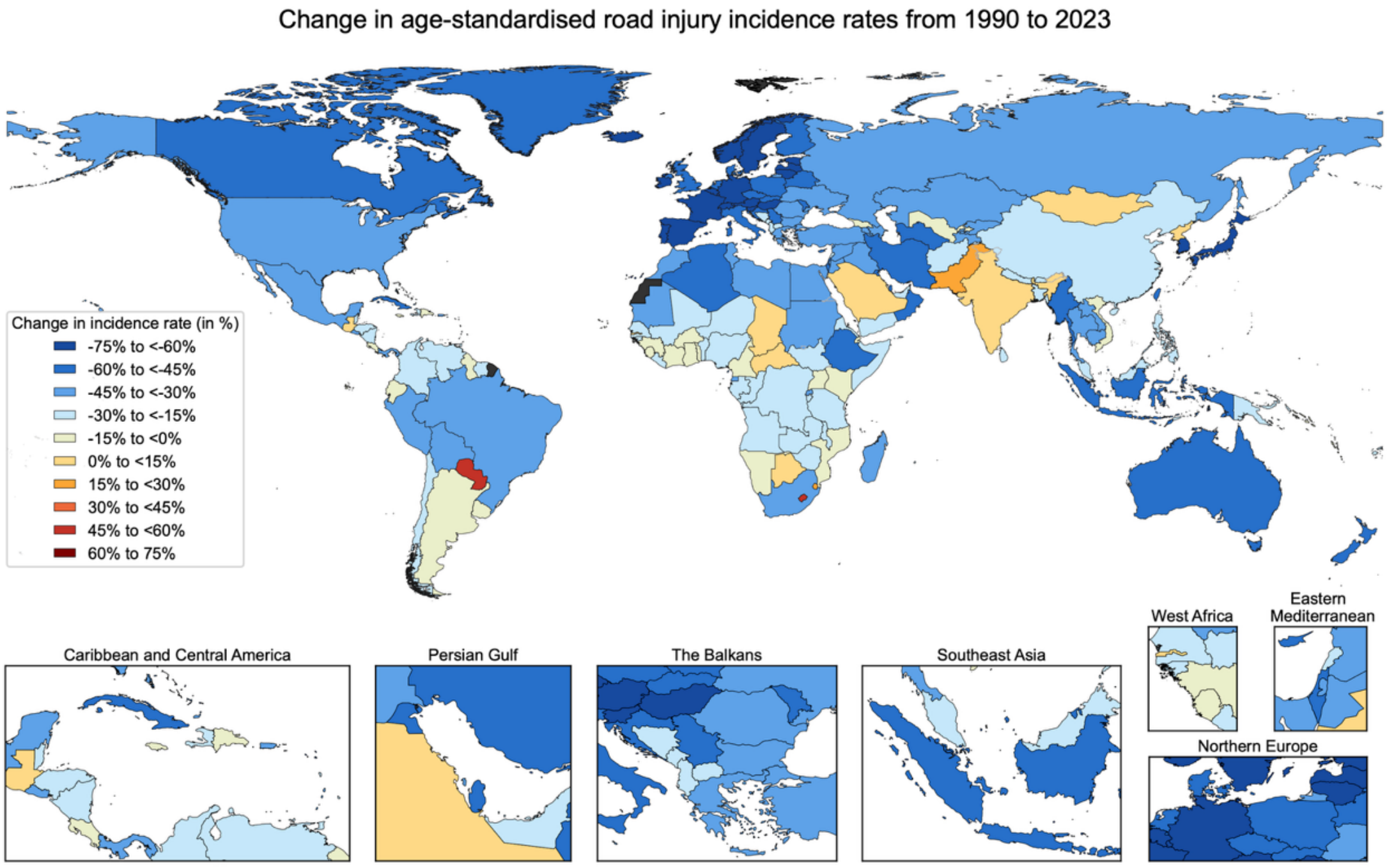

Figure S2. Change in age-standardised incidence rates attributable to road injuries from 1990 to 2023

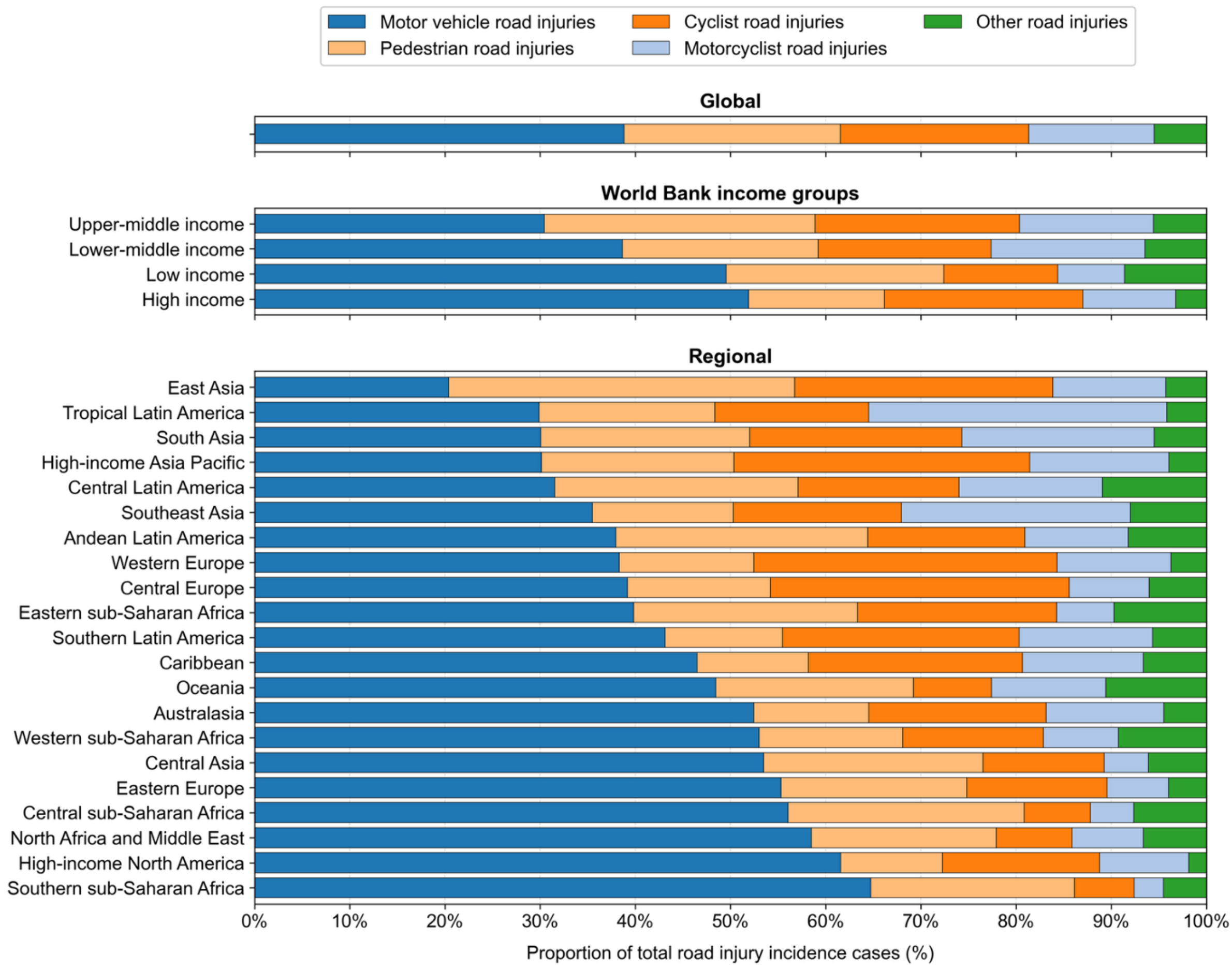

Figure S3. Proportion (%) of Level 4 road injury cases to the total road injury cases in 2023, globally and across the World Bank income groups and 21 GBD regions

Percentage change in age-standardised road injury incidence rates from 1990 to 2023

(Colour scale: −100, −75, −50, −25, 0, 25, 50, 75, 100)

	Motor vehicles	Motorcyclists	Cyclists	Pedestrians	Others
Global	-42.3 (-44.0 to -41.0)	-16.0 (-18.6 to -12.5)	-30.9 (-33.3 to -28.6)	-45.0 (-46.9 to -43.0)	-39.1 (-42.4 to -35.4)
World Bank low income	-30.2 (-32.5 to -28.1)	-4.6 (-7.4 to -1.5)	-17.9 (-20.7 to -15.1)	-23.8 (-26.0 to -21.7)	-25.7 (-29.3 to -21.7)
World Bank lower-middle income	-22.5 (-24.6 to -20.2)	13.3 (10.4 to 17.4)	-3.6 (-7.3 to 0.4)	-19.2 (-22.0 to -16.6)	-24.9 (-28.7 to -21.5)
World Bank upper-middle income	-31.5 (-34.9 to -28.5)	-6.0 (-10.4 to -1.1)	-15.7 (-19.9 to -11.5)	-39.5 (-42.4 to -36.9)	-40.6 (-44.5 to -35.6)
World Bank high income	-48.3 (-49.6 to -46.9)	-38.5 (-41.0 to -35.9)	-43.0 (-45.0 to -40.8)	-63.5 (-65.1 to -62.0)	-42.1 (-45.2 to -38.8)
Andean Latin America	-30.9 (-33.7 to -27.9)	97.9 (83.9 to 113.5)	5.0 (-2.1 to 12.6)	-41.5 (-43.8 to -39.3)	-26.4 (-30.6 to -21.9)
Australasia	-66.0 (-68.2 to -63.6)	-46.8 (-51.1 to -42.4)	-45.1 (-49.9 to -41.1)	-59.3 (-63.3 to -55.3)	-28.4 (-34.1 to -21.9)
Caribbean	-19.2 (-22.7 to -15.6)	72.5 (62.1 to 82.3)	-38.6 (-42.2 to -34.7)	-44.1 (-46.7 to -41.5)	-14.0 (-17.8 to -10.0)
Central Asia	-28.1 (-30.5 to -25.7)	-38.6 (-41.8 to -35.7)	-11.6 (-16.0 to -7.5)	-33.4 (-35.9 to -30.7)	-26.7 (-30.7 to -23.0)
Central Europe	-48.1 (-50.0 to -46.3)	-39.2 (-41.7 to -36.7)	-35.3 (-37.8 to -32.9)	-71.9 (-73.1 to -70.3)	-57.9 (-61.2 to -55.1)
Central Latin America	-29.1 (-31.4 to -26.8)	75.5 (62.3 to 90.3)	-21.7 (-26.1 to -17.5)	-53.7 (-55.6 to -52.0)	-41.6 (-44.7 to -38.6)
Central sub-Saharan Africa	-20.3 (-23.7 to -17.3)	23.6 (16.4 to 31.6)	-15.3 (-19.7 to -10.6)	-16.4 (-19.4 to -13.1)	-27.4 (-32.6 to -22.1)
East Asia	-22.7 (-27.6 to -18.0)	-11.9 (-16.0 to -6.4)	-4.2 (-9.8 to 1.5)	-24.1 (-28.5 to -20.3)	-32.0 (-38.2 to -24.4)
Eastern Europe	-30.2 (-33.3 to -26.5)	-37.8 (-41.4 to -34.0)	-31.9 (-35.6 to -28.2)	-67.5 (-69.2 to -66.0)	-38.7 (-43.8 to -34.0)
Eastern sub-Saharan Africa	-34.8 (-36.9 to -32.8)	-16.1 (-19.0 to -13.1)	-17.2 (-20.2 to -14.6)	-31.5 (-33.7 to -29.0)	-27.1 (-30.9 to -23.3)
High-income Asia Pacific	-69.3 (-71.3 to -67.5)	-66.3 (-68.7 to -63.9)	-62.3 (-65.0 to -59.9)	-72.4 (-74.5 to -70.3)	-53.9 (-57.9 to -50.3)
High-income North America	-45.6 (-47.3 to -43.9)	-8.7 (-16.6 to 0.6)	-36.5 (-40.7 to -31.7)	-41.7 (-44.1 to -38.6)	-16.1 (-22.5 to -7.9)
North Africa and Middle East	-43.6 (-45.8 to -41.4)	-25.3 (-28.0 to -22.3)	-18.3 (-23.4 to -14.0)	-38.5 (-41.6 to -35.7)	-40.2 (-43.6 to -37.0)
Oceania	-14.9 (-18.0 to -11.9)	7.3 (1.5 to 13.9)	-11.7 (-15.0 to -7.7)	-11.3 (-14.4 to -7.8)	-6.7 (-10.5 to -1.7)
South Asia	-3.1 (-6.7 to 0.6)	33.8 (28.6 to 39.5)	1.4 (-2.9 to 5.7)	-2.3 (-6.7 to 2.2)	-23.2 (-28.1 to -18.8)
Southeast Asia	-45.3 (-47.4 to -43.2)	-35.4 (-37.8 to -33.3)	-26.4 (-30.2 to -22.5)	-53.0 (-54.9 to -51.1)	-46.4 (-50.3 to -42.3)
Southern Latin America	-10.0 (-13.8 to -6.0)	82.0 (67.5 to 102.9)	-4.6 (-9.0 to 1.5)	-42.5 (-45.4 to -39.1)	-22.2 (-26.5 to -17.5)
Southern sub-Saharan Africa	-33.6 (-36.4 to -30.7)	-28.9 (-32.6 to -25.3)	-17.8 (-21.9 to -12.8)	-39.1 (-41.7 to -36.6)	-23.3 (-27.3 to -19.0)
Tropical Latin America	-17.1 (-22.3 to -10.0)	92.0 (75.0 to 114.2)	-2.3 (-11.7 to 10.4)	-70.4 (-72.3 to -68.6)	-64.6 (-68.1 to -61.0)
Western Europe	-69.8 (-71.7 to -68.1)	-42.2 (-45.1 to -39.0)	-43.0 (-45.5 to -40.4)	-71.8 (-74.1 to -69.2)	-42.2 (-46.2 to -38.0)
Western sub-Saharan Africa	-14.9 (-17.0 to -13.1)	1.4 (-1.3 to 4.0)	-9.8 (-13.3 to -6.3)	-13.6 (-15.8 to -11.9)	-18.1 (-21.1 to -15.3)

Figure S4. Percentage change (95% UI) in age-standardised road injury incidence rates from 1990 to 2023, globally and across the World Bank income groups and 21 GBD regions

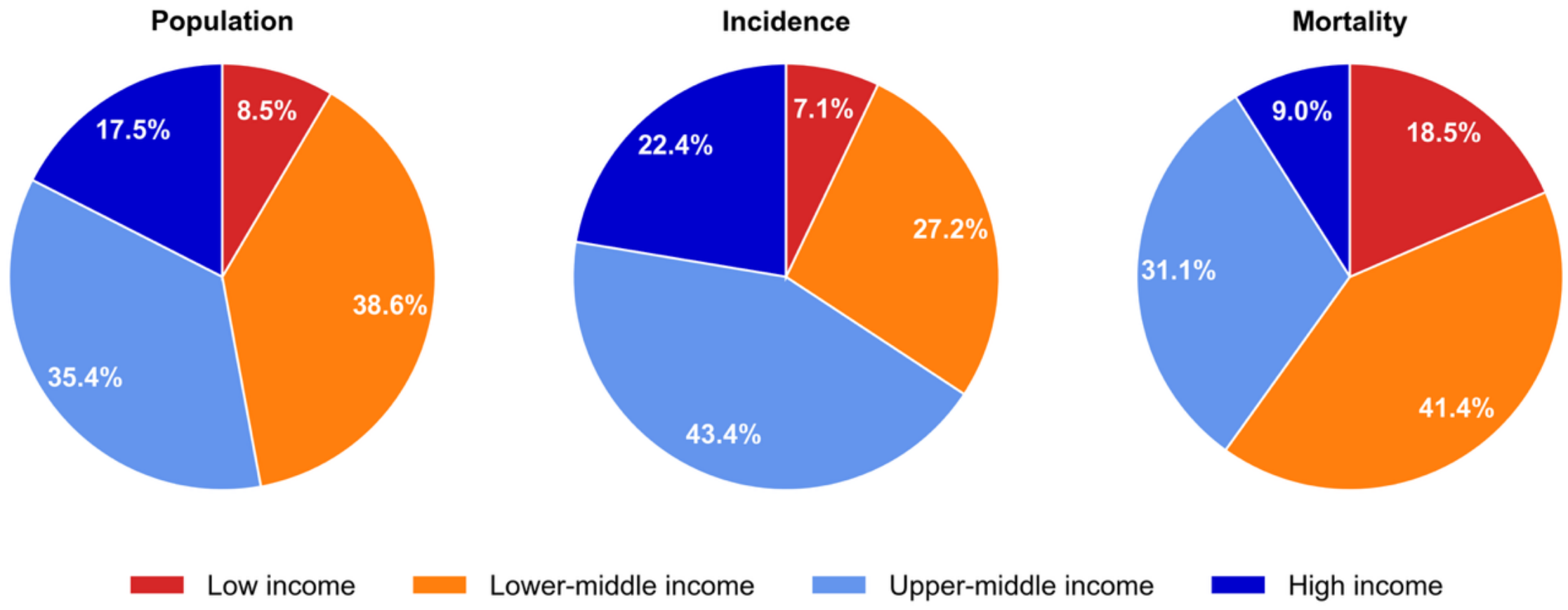

Figure S5. Proportion of the global population, road injury incident cases, and road injury deaths accounted for by each World Bank income group in 2023

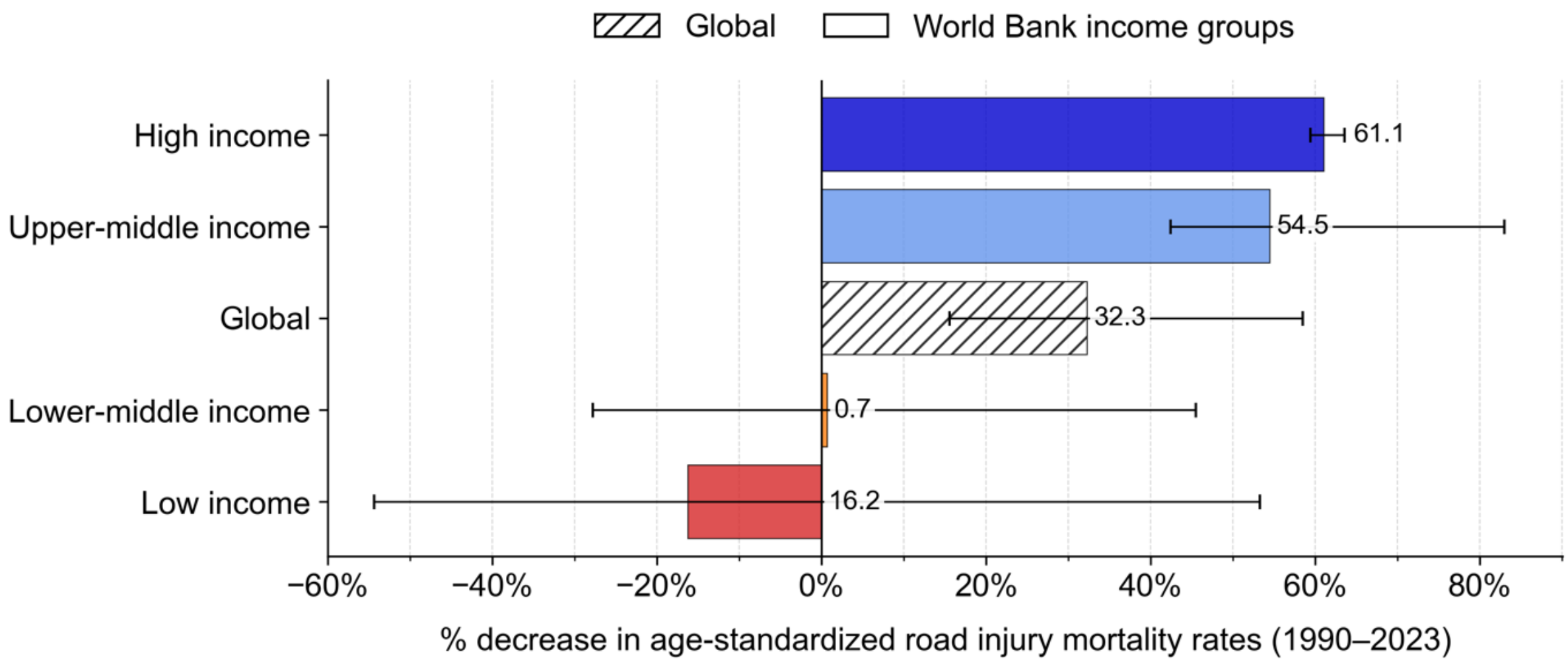

Figure S6. Percentage decrease in age-standardised road injury mortality rates from 1990 to 2023, globally and across the World Bank income groups. Values denote the percentage decrease, and error bars represent the 95% uncertainty interval.

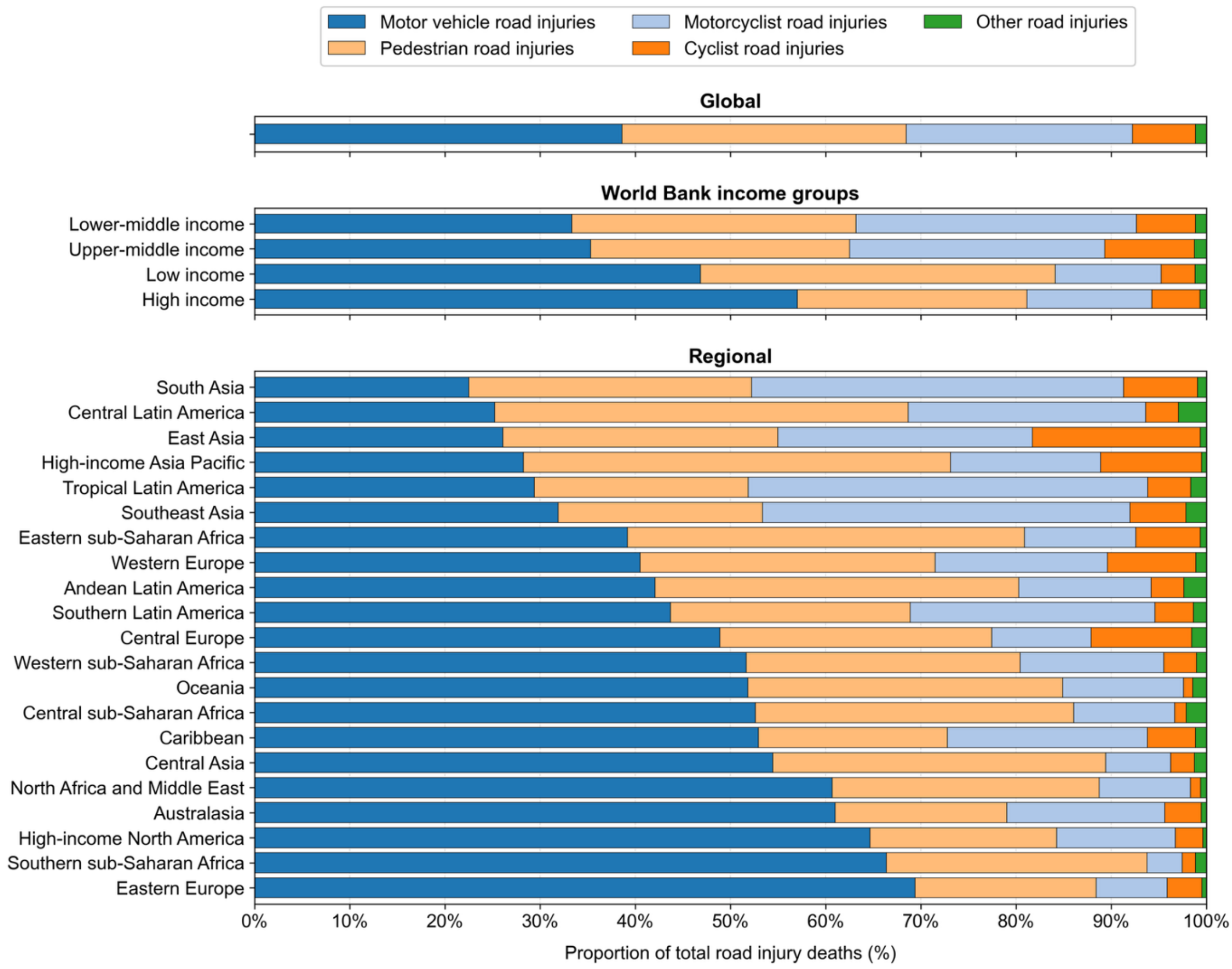

Figure S7. Percentage (%) of Level 4 road injury deaths to the total road deaths in 2023, globally and across the World Bank income groups and 21 GBD regions

Percentage change in age-standardised road injury mortality rates from 1990 to 2023

−100 −75 −50 −25 0 25 50 75 100

	Motor vehicles	Motorcyclists	Cyclists	Pedestrians	Others
Global	-26.4 (-48.9 to 7.2)	38.2 (-27.4 to 146.6)	8.4 (-50.1 to 98.3)	-57.7 (-71.9 to -35.0)	-17.7 (-65.1 to 69.8)
World Bank low income	7.6 (-30.5 to 72.5)	106.5 (-10.0 to 381.5)	64.5 (-36.5 to 331.5)	9.1 (-35.8 to 104.9)	73.2 (-37.3 to 432.0)
World Bank lower-middle income	-15.9 (-43.4 to 33.6)	81.7 (-8.1 to 242.4)	54.1 (-36.1 to 233.0)	-23.3 (-55.2 to 33.7)	18.5 (-58.7 to 239.5)
World Bank upper-middle income	-37.0 (-66.1 to 3.0)	9.6 (-46.8 to 107.6)	-6.2 (-68.5 to 105.8)	-78.8 (-85.6 to -61.0)	-45.4 (-76.5 to 8.7)
World Bank high income	-54.9 (-58.1 to -51.5)	-44.1 (-52.4 to -36.4)	-56.0 (-61.1 to -50.4)	-76.0 (-78.6 to -72.7)	-63.8 (-70.8 to -56.5)
Andean Latin America	6.8 (-17.7 to 42.1)	139.7 (52.7 to 252.4)	114.1 (28.0 to 261.7)	-29.2 (-45.0 to -1.9)	69.7 (-18.2 to 208.9)
Australasia	-72.1 (-74.7 to -69.3)	-57.3 (-63.4 to -50.0)	-64.8 (-73.5 to -54.5)	-75.6 (-79.2 to -71.6)	2.3 (-22.5 to 38.1)
Caribbean	-2.7 (-19.1 to 21.1)	105.6 (38.4 to 210.6)	-51.7 (-66.0 to -23.0)	-39.1 (-56.1 to -19.3)	4.9 (-27.9 to 61.9)
Central Asia	-35.5 (-50.3 to -18.4)	-52.3 (-67.1 to -25.2)	-15.8 (-39.6 to 20.7)	-48.7 (-61.3 to -24.3)	-58.6 (-73.2 to -35.6)
Central Europe	-57.5 (-61.3 to -52.8)	-48.1 (-56.3 to -38.5)	-42.5 (-51.9 to -30.5)	-84.1 (-86.1 to -80.8)	-81.4 (-85.1 to -75.8)
Central Latin America	-35.3 (-44.6 to -23.9)	79.8 (55.2 to 115.5)	-16.2 (-32.4 to 3.8)	-61.6 (-66.7 to -53.7)	-48.6 (-62.6 to -28.1)
Central sub-Saharan Africa	23.6 (-22.7 to 97.4)	217.9 (17.2 to 679.1)	138.2 (-31.1 to 718.4)	35.4 (-25.3 to 156.0)	99.6 (-29.2 to 523.5)
East Asia	-63.1 (-87.1 to -6.3)	-22.2 (-72.0 to 109.0)	-9.6 (-75.8 to 154.9)	-86.9 (-92.1 to -69.7)	-65.8 (-93.0 to 69.7)
Eastern Europe	-32.2 (-44.0 to -17.3)	-61.1 (-71.9 to -45.0)	-55.0 (-67.9 to -35.6)	-88.6 (-91.2 to -84.2)	-80.1 (-86.4 to -69.9)
Eastern sub-Saharan Africa	3.9 (-38.3 to 81.0)	116.1 (-10.2 to 448.2)	58.3 (-39.7 to 333.9)	2.1 (-40.6 to 88.4)	69.9 (-40.1 to 581.2)
High-income Asia Pacific	-83.9 (-87.3 to -74.0)	-83.3 (-90.0 to -74.9)	-83.0 (-87.7 to -76.7)	-83.2 (-87.3 to -73.1)	-84.9 (-94.3 to -34.5)
High-income North America	-41.4 (-45.6 to -36.9)	23.5 (4.7 to 42.1)	-29.5 (-43.6 to -12.2)	-27.2 (-36.9 to -16.8)	-56.3 (-65.5 to -44.0)
North Africa and Middle East	-43.4 (-62.6 to -8.4)	-5.7 (-62.7 to 132.5)	72.7 (-47.6 to 433.3)	-48.6 (-74.2 to 18.0)	-66.5 (-93.1 to 58.0)
Oceania	-9.4 (-42.7 to 32.6)	53.0 (-23.8 to 237.5)	39.0 (-24.4 to 174.7)	-13.3 (-50.1 to 60.4)	15.8 (-42.7 to 231.5)
South Asia	-22.3 (-54.1 to 45.4)	96.6 (-4.0 to 264.2)	53.7 (-42.0 to 253.7)	-27.3 (-58.3 to 30.1)	24.6 (-64.8 to 281.1)
Southeast Asia	-26.7 (-53.2 to 23.7)	17.8 (-33.8 to 119.7)	61.1 (-17.2 to 236.5)	-44.9 (-69.6 to 5.4)	-7.8 (-66.8 to 160.8)
Southern Latin America	-38.7 (-46.4 to -29.2)	232.2 (161.1 to 322.9)	-21.9 (-39.6 to 8.6)	-58.7 (-65.8 to -46.2)	-59.7 (-71.1 to -42.1)
Southern sub-Saharan Africa	-4.7 (-33.7 to 33.7)	-1.7 (-56.7 to 106.0)	-11.6 (-54.4 to 80.9)	-33.2 (-63.7 to 9.5)	-30.8 (-62.8 to 41.3)
Tropical Latin America	11.3 (-2.6 to 26.6)	117.7 (85.8 to 150.0)	80.8 (59.3 to 106.9)	-81.6 (-83.6 to -79.1)	-48.6 (-62.9 to -26.0)
Western Europe	-82.1 (-83.6 to -80.1)	-57.4 (-61.7 to -52.4)	-59.3 (-64.4 to -53.0)	-83.3 (-85.3 to -81.1)	-55.9 (-62.1 to -49.5)
Western sub-Saharan Africa	-7.2 (-40.2 to 43.6)	109.1 (-1.1 to 390.6)	68.0 (-23.9 to 308.5)	1.7 (-41.8 to 87.5)	29.8 (-50.8 to 248.4)

Figure S8. Percentage change (95% UI) in age-standardised road injury mortality rates from 1990 to 2023, globally and across the World Bank income groups and 21 GBD regions

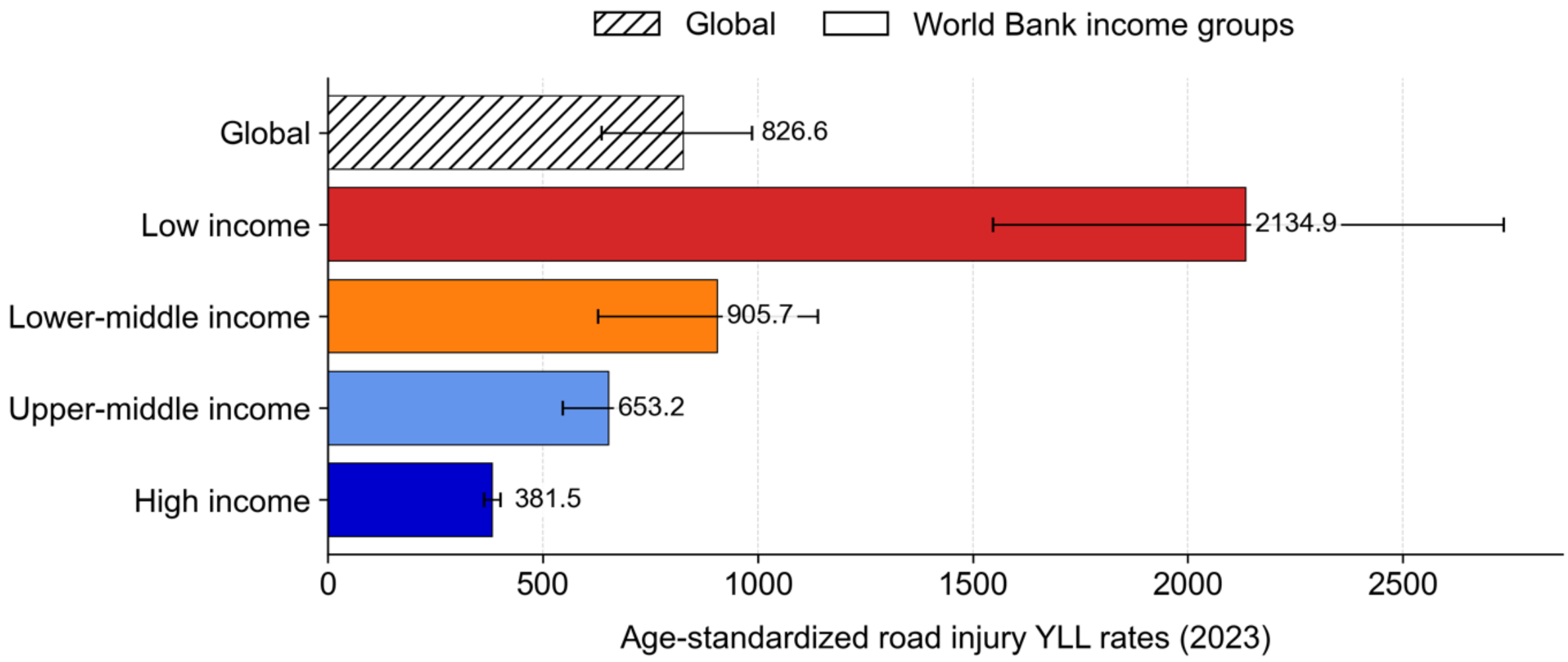

Figure S9. Age-standardised road injury YLL rates for both sexes combined, 2023, globally and across the World Bank income groups. Error bars represent the 95% uncertainty interval. YLLs=years of life lost.

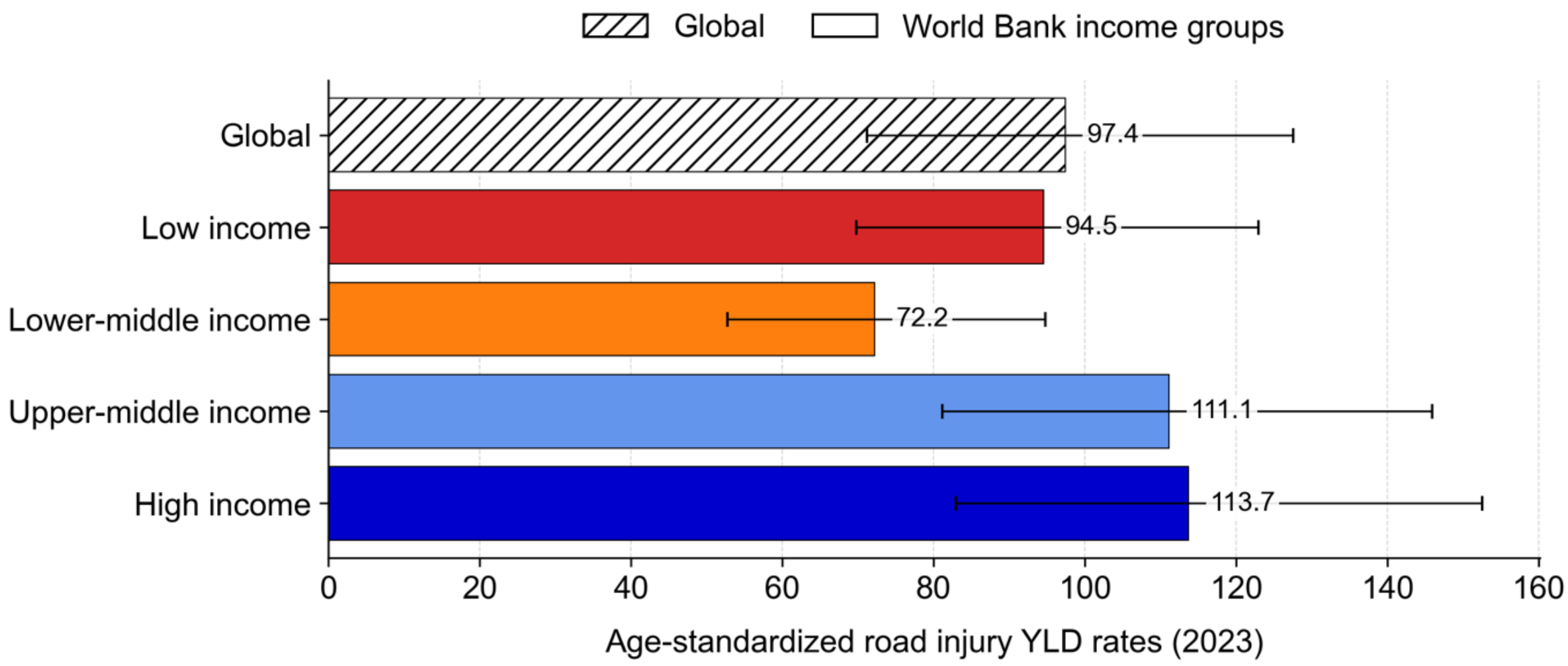

Figure S10. Age-standardised road injury YLD rates for both sexes combined, 2023, globally and across the World Bank income groups. Error bars represent the 95% uncertainty interval. YLDs=years lived with disability.

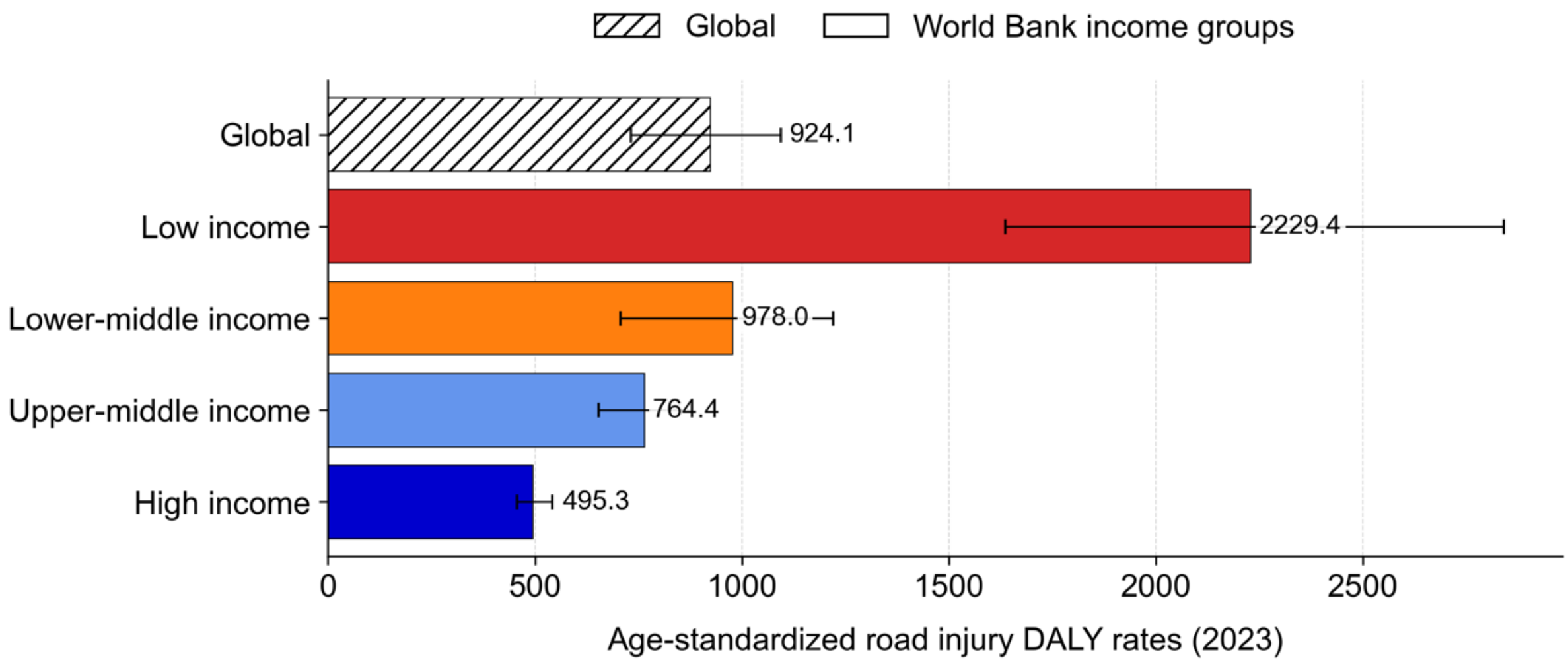

Figure S11. Age-standardised road injury DALY rates for both sexes combined, 2023, globally and across the World Bank income groups. Error bars represent the 95% uncertainty interval. DALYs=disability-adjusted life-years.

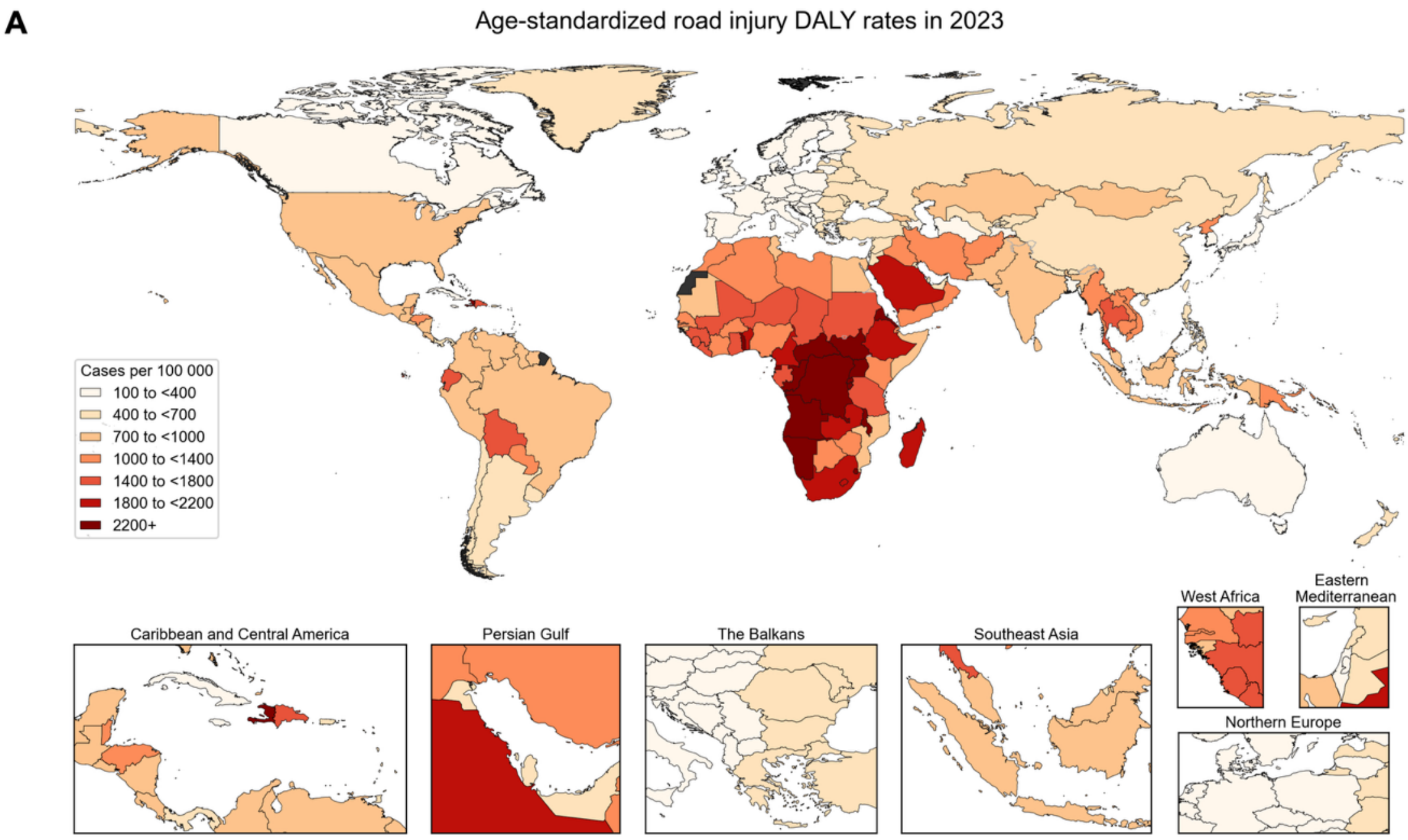

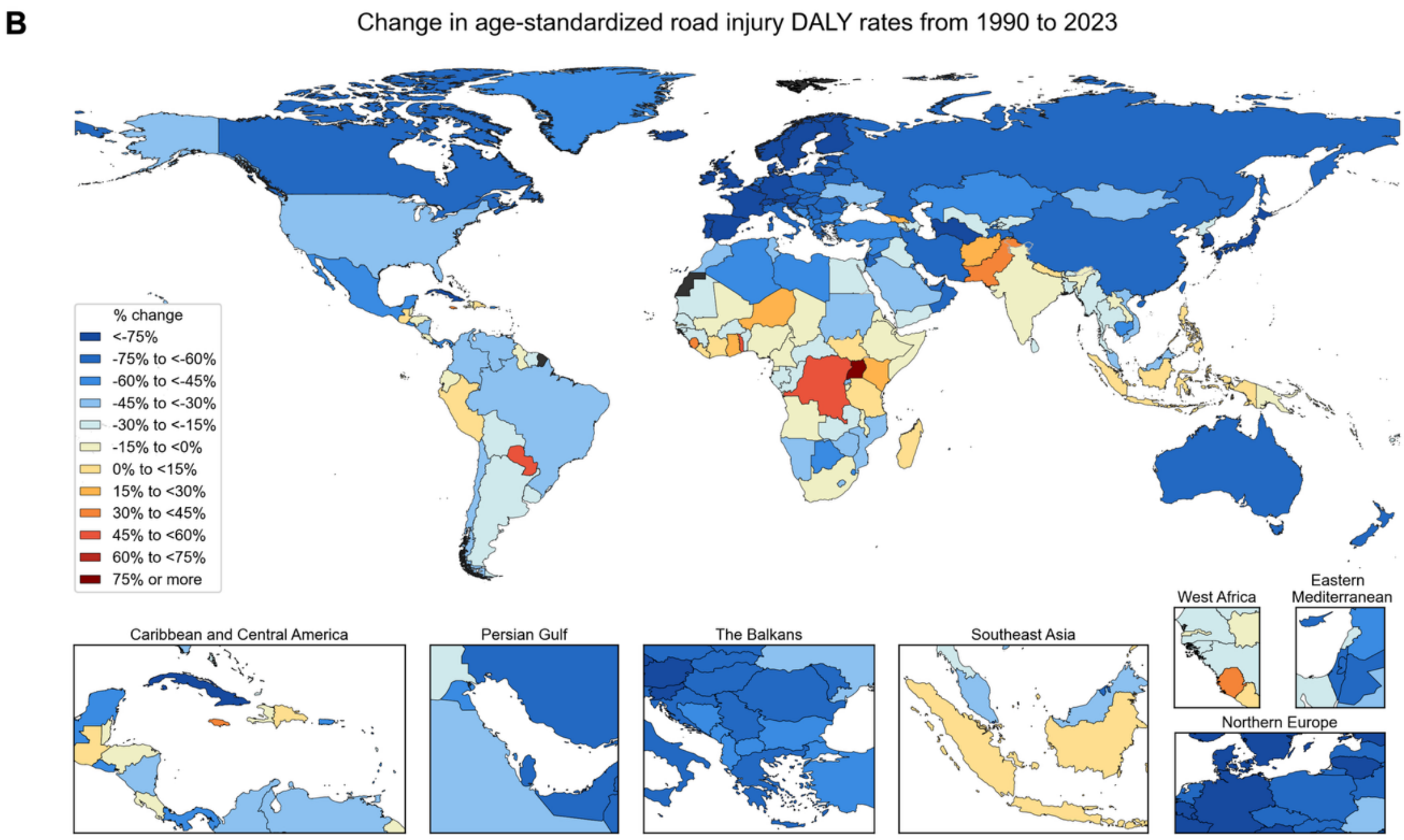

Figure S12. Age-standardised DALY rates (per 100 000 population) attributable to road injuries in 2023. **B**: Change in age-standardised DALY rates attributable to road injuries from 1990 to 2023. DALYs=disability-adjusted life-years.

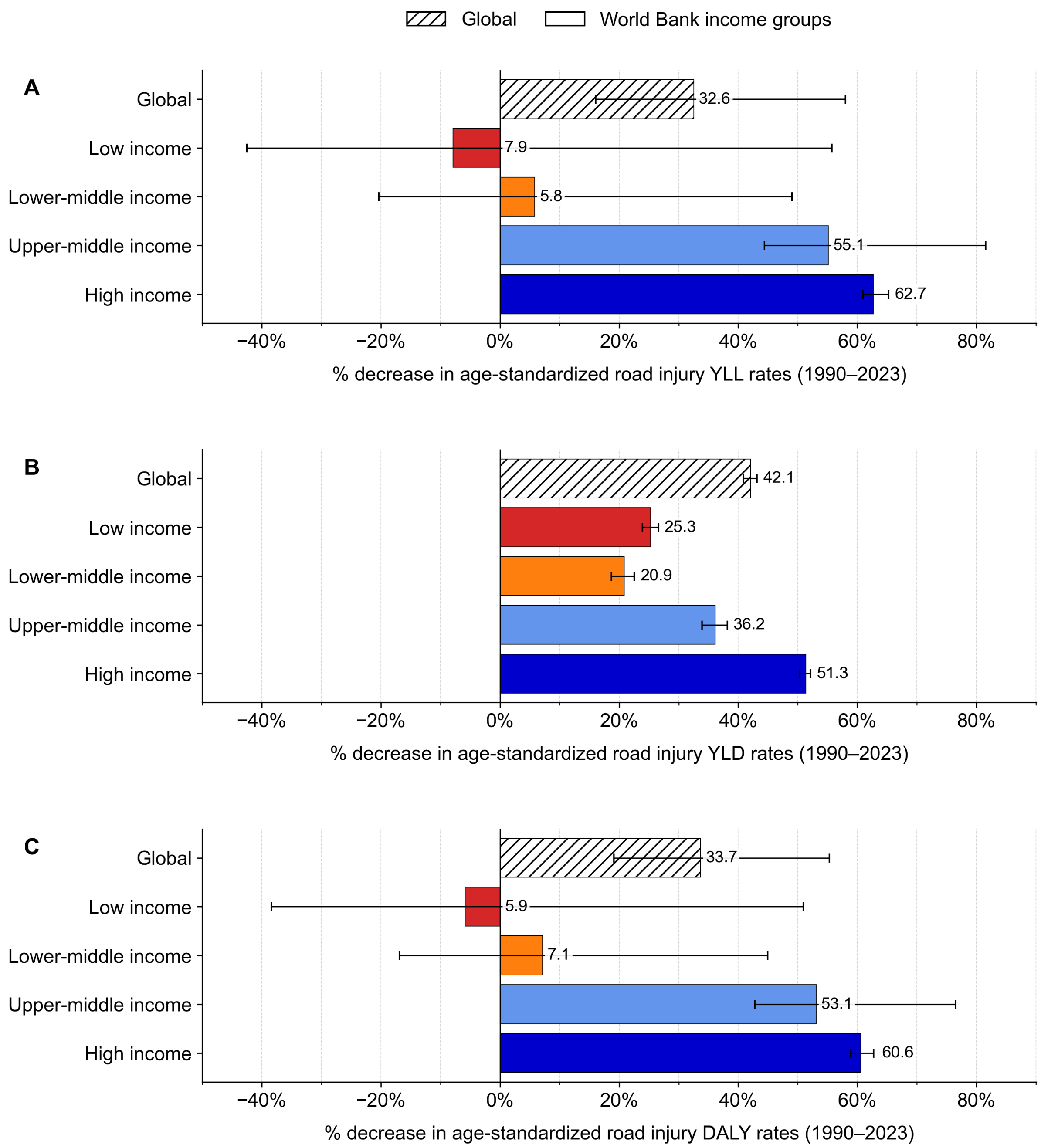

Figure S13. Percentage decrease in age-standardised road injury burden from 1990 to 2023, globally and across the World Bank income groups. **Panel A**: YLL rates. **Panel B**: YLD rates. **Panel C:** DALY rates. Values denote the percentage decrease, and error bars represent the 95% uncertainty interval. YLLs=years of life lost. YLDs=years lived with disability. DALYs=disability-adjusted life-years.

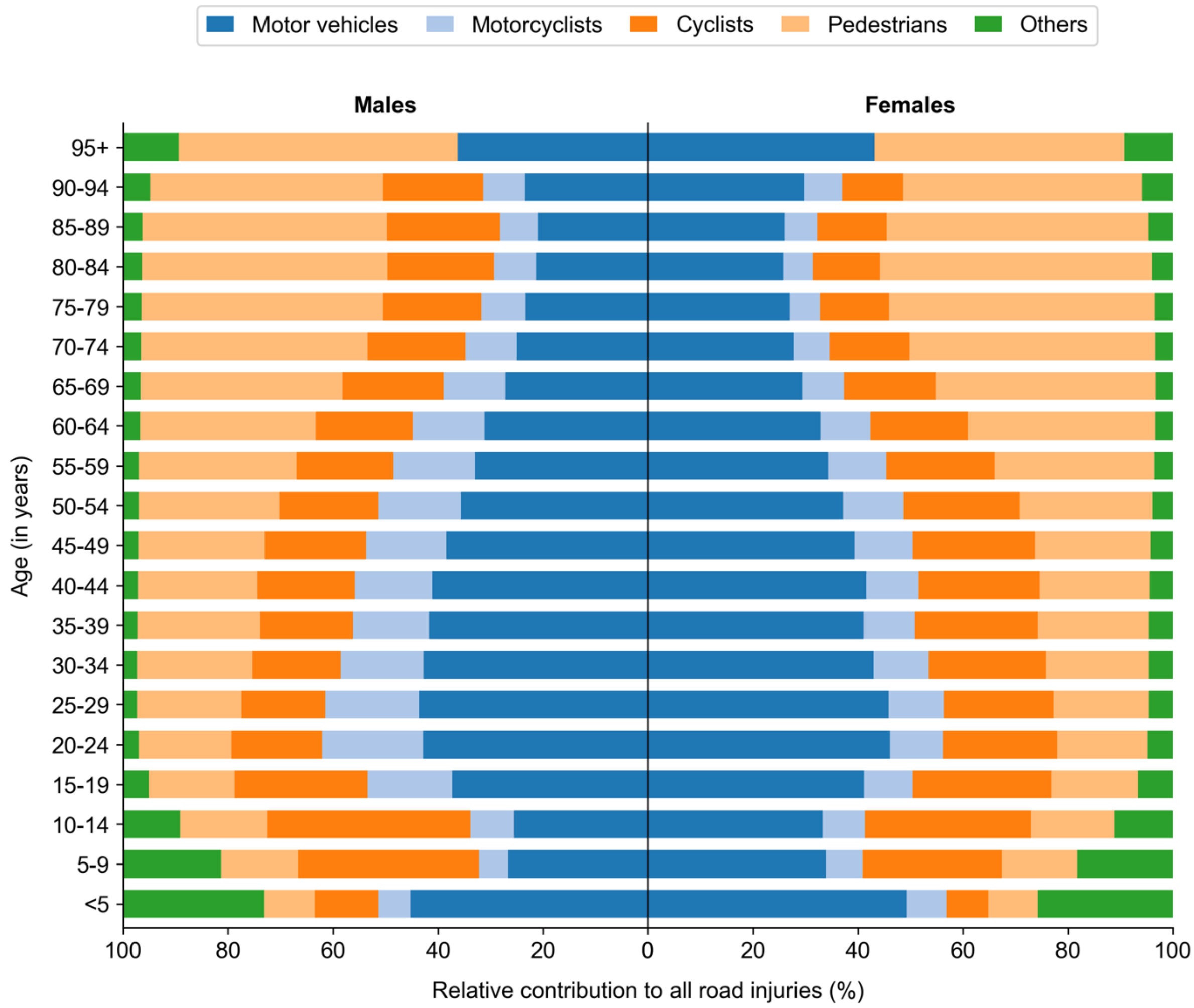

Figure S14. Relative contribution (%) of Level 4 road injury incidence rates to all road injuries by sex and age group, globally in 2023

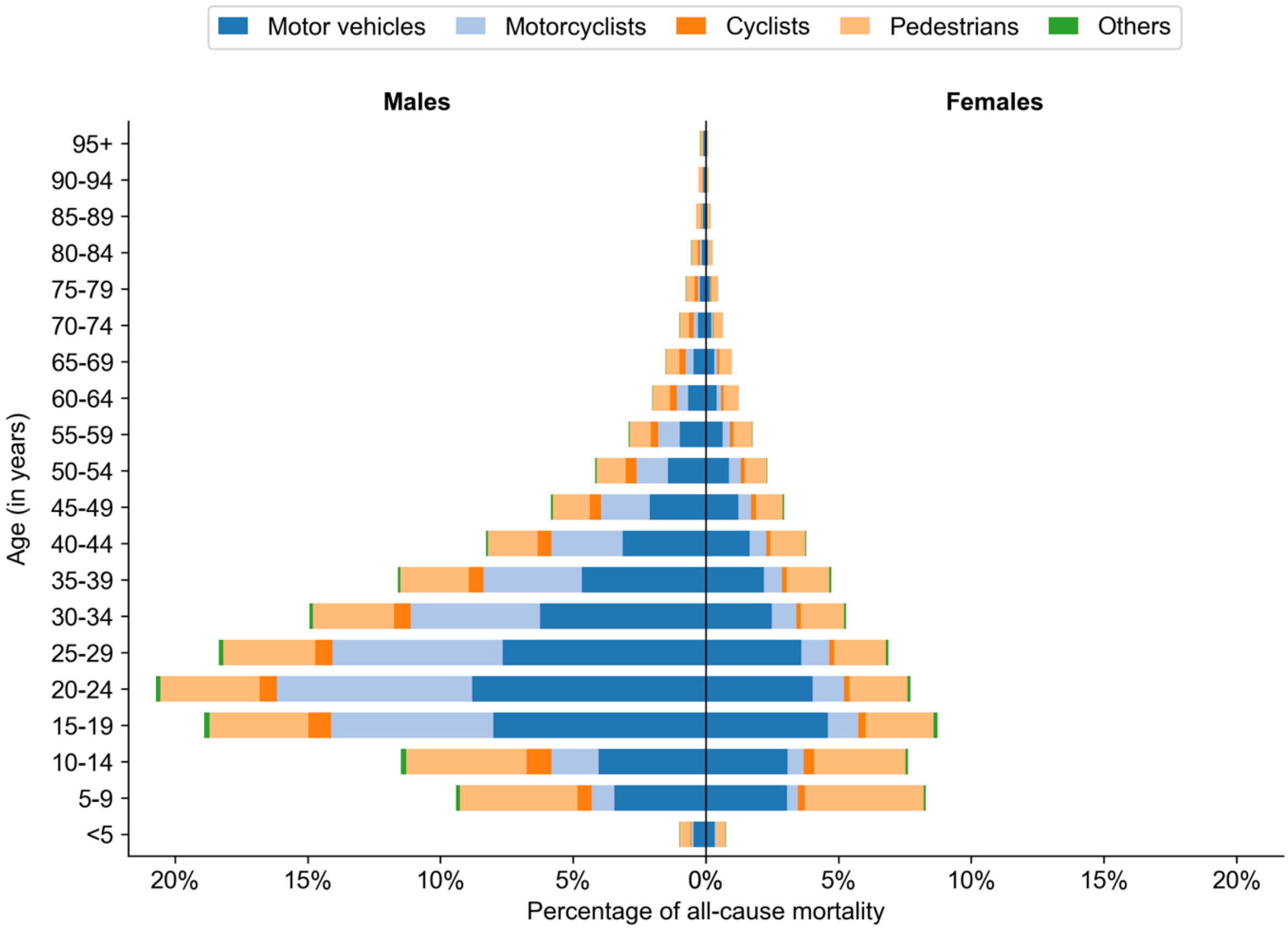

Figure S15. Road injury deaths as a percentage of all-cause mortality, stratified by sex, age group, and Level 4 road injury cause, globally in 2023

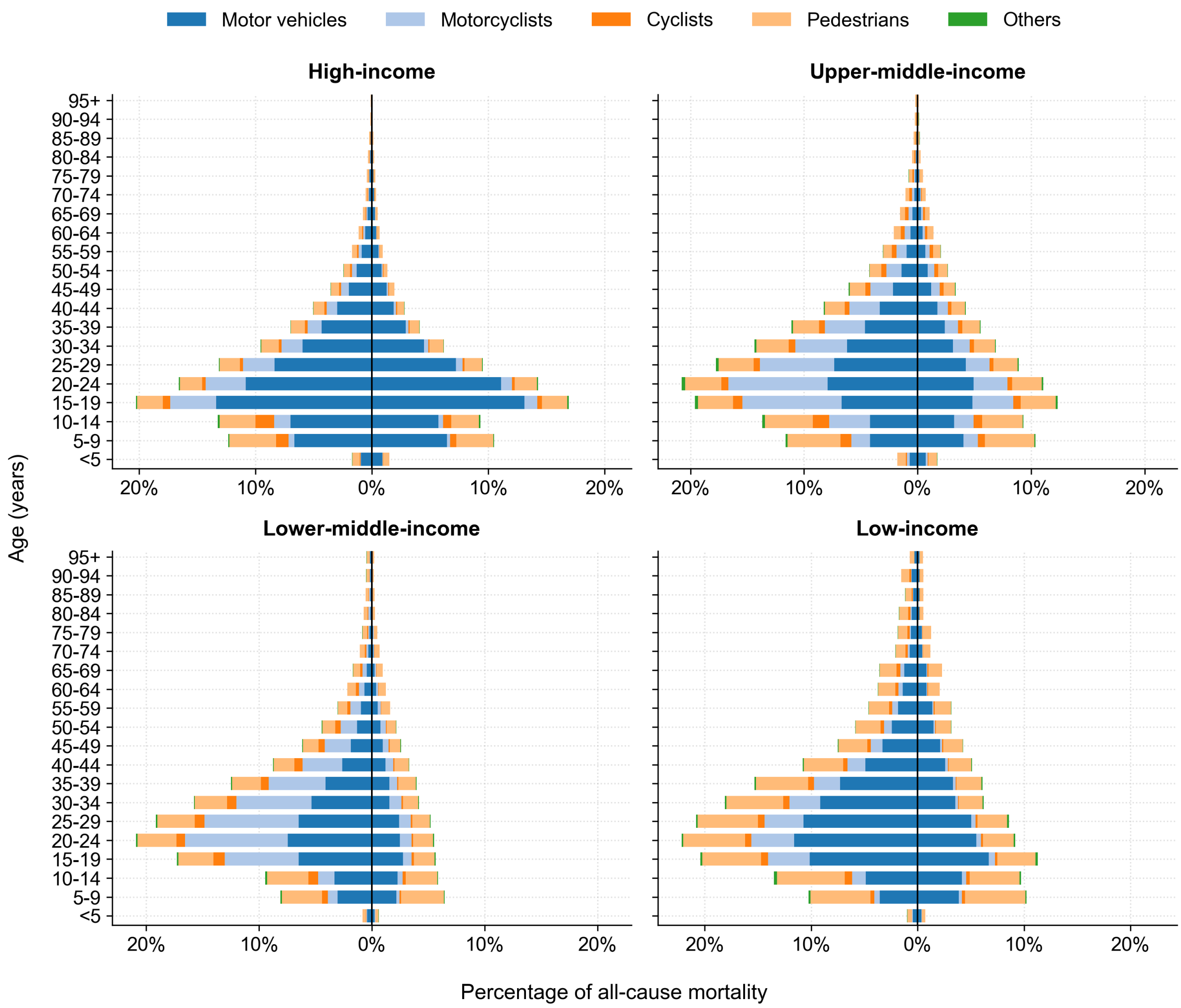

Figure S16. Road injury deaths as a percentage of all-cause mortality in 2023, stratified by sex, age group, Level 4 road injury cause, and World Bank income group.

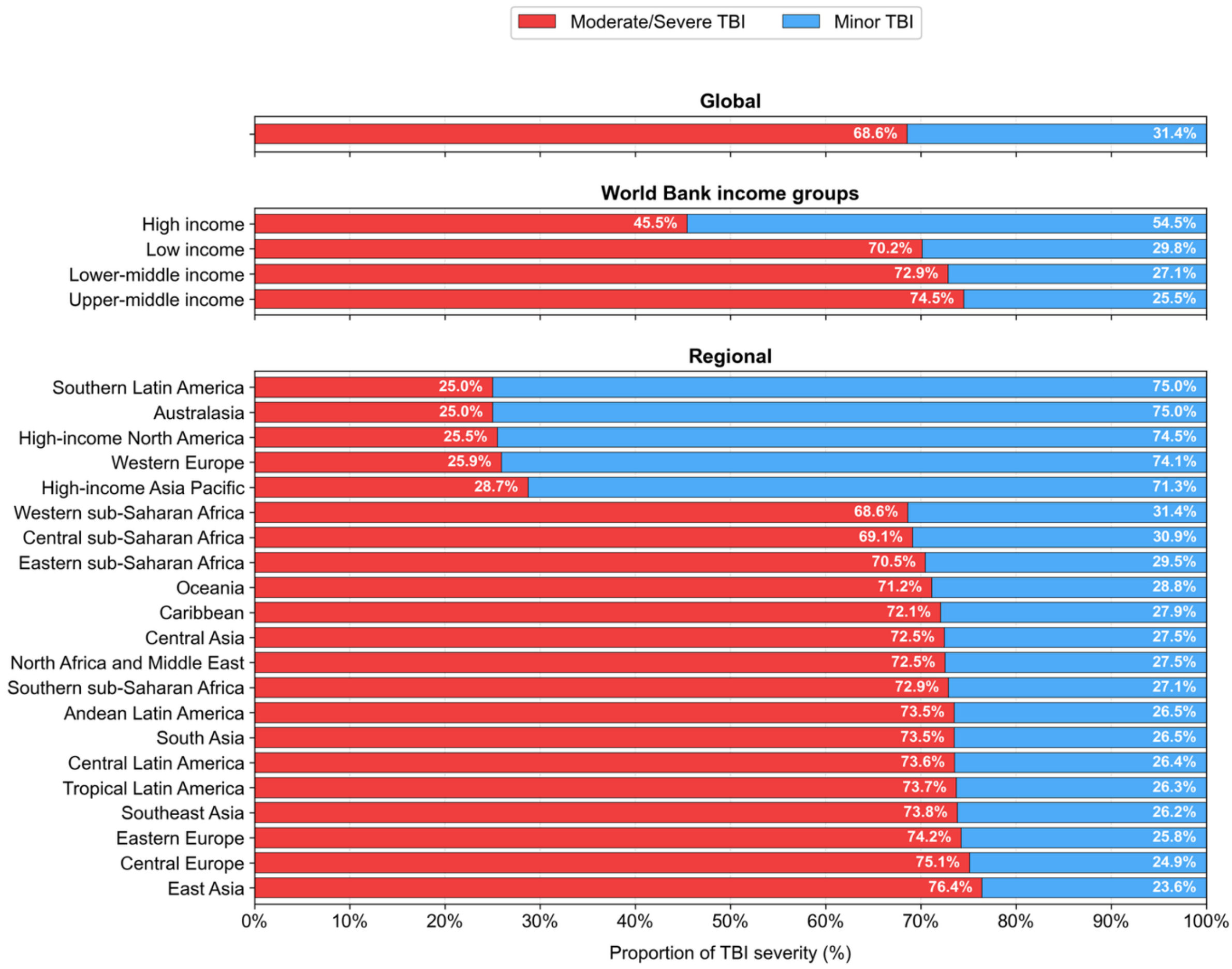

Figure S17. Proportion of minor and moderate/severe traumatic brain injuries (TBIs) among head injuries attributable to road injuries, globally and across World Bank income groups and 21 GBD regions in 2023

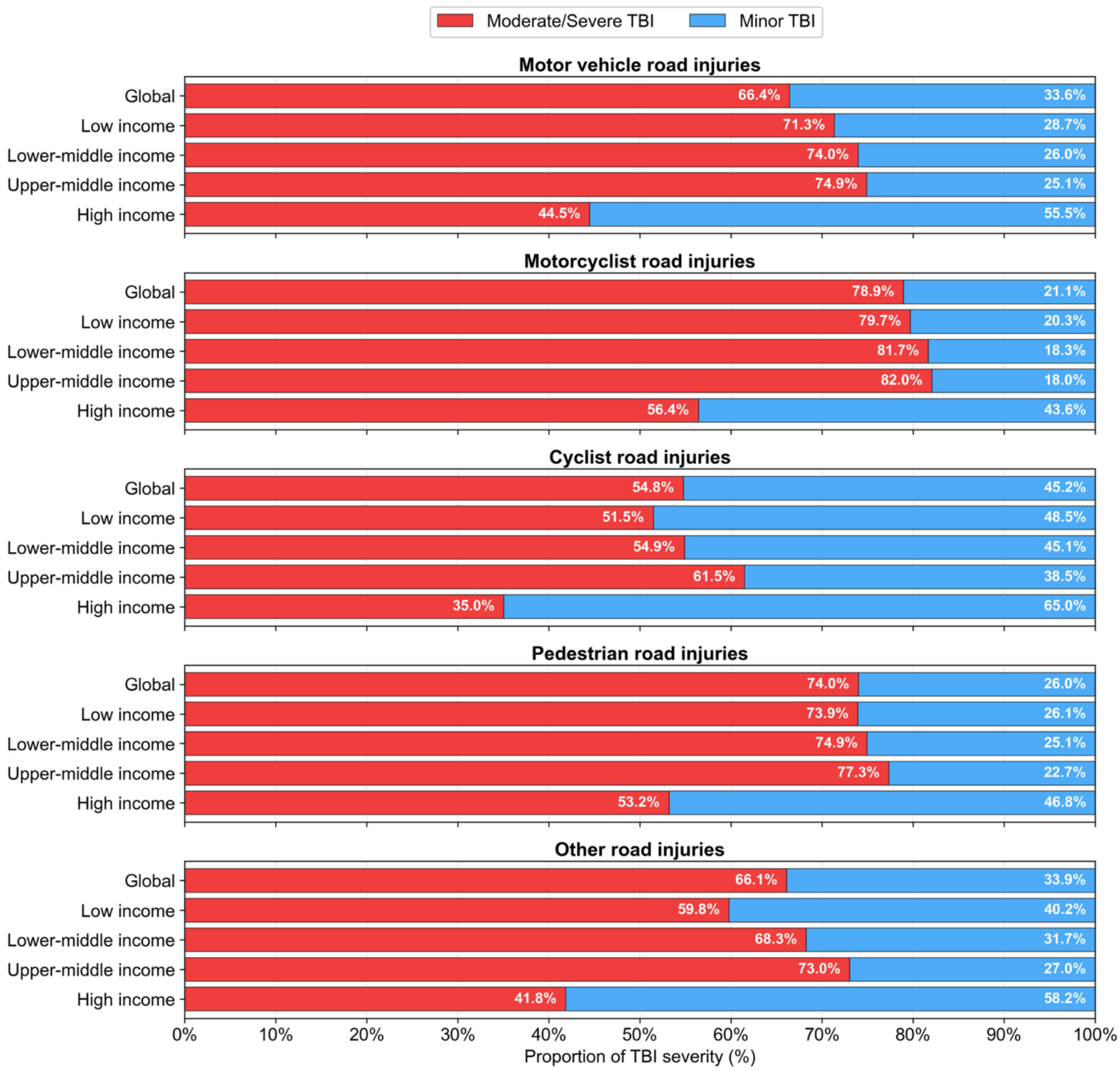

Figure S18. Proportion of minor and moderate/severe traumatic brain injuries (TBIs) among head injuries attributable to each Level 4 road injury cause, globally and across the World Bank income groups
