# Supplementary material for: Global, regional, and national burden of road injuries 1990–2023: a systematic analysis for the Global Burden of Disease Study 2023
Source: Lancet Public Health. 2026 Jul 20;11(9):e589–603. doi: 10.1016/S2468-2667(26)00141-6 (PMC13487794; doi:10.1016/S2468-2667(26)00141-6)
Supplement: Supplementary file 2 [file mmc2.pdf]

THE LANCET
Public Health

Supplementary appendix 2

This appendix formed part of the original submission and has been peer reviewed. We post it as supplied by the authors.

Supplement to: GBD 2023 Road Injuries Collaborators. Global, regional, and national burden of road injuries 1990–2023: a systematic analysis for the Global Burden of Disease Study 2023. *Lancet Public Health* 2026; published online July 20. https://doi.org/10.1016/S2468-2667(26)00141-6.

Appendix 2: Authorship appendix to "Global, regional, and national burden of road injuries: a systematic analysis from the Global Burden of Disease Study 2023"

This appendix provides further authorship detail for "Global, regional, and national burden of road injuries: a systematic analysis from the Global Burden of Disease Study 2023"

Table of Contents

Appendix 2: Authorship appendix to "Global, regional, and national burden of road injuries: a systematic analysis from the Global Burden of Disease Study 2023" 1

GBD 2023 Road Injuries Collaborators 2

Affiliations 7

Authors' Contributions 33

Providing data or critical feedback on data sources 33

Developing methods or computational machinery 35

Providing critical feedback on methods or results 35

Drafting the work or revising it critically for important intellectual content 40

Managing the estimation or publications process 43

GBD 2023 Road Injuries Collaborators

Stephanie C C van der Lubbe*, Madeline E Moberg*, Dominique Aluquin, Yawen Zhou, Hailey Lenox, Abdullah, Bhoomadevi A, Hasan Aalruz, Ukachukwu O Abaraogu, Zahra Abbasi Dolatabadi, Hedayat Abbastabar, Siddig Ibrahim Abdelwahab, Meriem Abdoun, Zinat Abdrakhmanova, Deldar Morad Abdulah, Auwal Abdullahi, Toufik Abdul-Rahman, Tesfaye Birhanu Abebe, Armita Abedi, Hansani Madushika Abeywickrama, Olumide Abiodun, Richard Gyan Aboagye, Abdullahi Tunde Aborode, Nagah M Abourashed, Mohamed Abouzid, Teklebirhan Abrha Abrha, Dariush Abtahi, Fuad Hamdi A Abuadas, Aminu Kende Abubakar, Rana Kamal Abu-Farha, Ahmad Y Abuhelwa, Hana J Abukhadijah, Niveen ME Abu-Rmeileh, Dina Abushanab, Apurba Acharya, Kiran Acharya, Aishah Fadila Adamu, Isaac Yeboah Addo, Tajudeen Adesanmi Adebisi, Babatope Oluwadamilare Adebiyi, Kamoru Ademola Adedokun, Victor Adekanmbi, Olumide Thomas Adeleke, Adekola George Adepoju, Miracle Ayomikun Adesina, Prince Owusu Adoma, Obed Adonteng-Kissi, Clifford Afoakwah, Aanuoluwapo Adeyimika Afolabi, Ebenezer Afrifa-Yamoah, Aiman P Afsar, Mahnaz Afshari, Saira Afzal, Vijay K Aggarwal, Mahdi Aghaalikhani, Salome Wumpini Agordoh, Shreyash Agrawal, Williams Agyemang-Duah, Abdul Momin Rizwan Ahmad, Aqeel Ahmad, Danish Ahmad, Khabir Ahmad, Muayyad M Ahmad, Sajjad Ahmad, Tauseef Ahmad, Ali Ahmadi, Ali Ahmed, Ayman Ahmed, Haroon Ahmed, Mehrunnisha Sharif Ahmed, Mohamed Sherif Ali Ahmed, Muktar Beshir Ahmed, Shabbir Ahmed, Dolapo Emmanuel Ajala, Marjan Ajami, Neda Akhoundi, Salah Al Awaidy, Syed Mahfuz Al Hasan, Mohammad Ahmmad Mahmoud Al Zoubi, Sami Saleh Alabdulwahab, Khurshid Alam, Mohammad Khursheed Alam, Mohammed Fasihul Alam, Mostafa Alam, Muath A Alammar, Fahad Mashhour Alanezi, Amani N. Alansari, Fahmi Y Al-Ashwal, Hussin Albargi, Mohammed Albashtawy, Shereen M Aleidi, Fadwa Naji Alhalaiqa, Amidu Alhassan, Ashraf Alhumaidi, Dari Alhuwail, Irfan Ali, Kamran Ali, Mohammad Shahid Ali, Mohammed Usman Ali, Sheikh Mohammad Alif, Samah W Al-Jabi, Moath Saleh Aljohani, Syed Mohamed Aljunid, Zaid I Alkhatib, Hazim Alkousheh, Sameer Abdulmalik Alkubati, Khaled S. Allemailem, Mohammed Z Allouh, Wesam Taher Almagharbeh, Sabah Al-Marwani, Ahmad Almatroudi, Joseph Uy Almazan, Khalid Almugheed, Mohmmad Minwer Alnaeem, Fahad Salman Alotibi, Mushtak Talib Salih Al-Ouqaili, Jaber S Alqahtani, Saeed Ali S Alqahtani, Ahmad Alrawashdeh, Intima Alrimawi, Sahel Majed Alrousan, Mohammed A Alsabri, Mohammed Amjed Alsaegh, Bandar Alsaif, Abdullah Alshami, Ali Salman Al-Shami, Abdalkarem Fedgash Alsharari, Zaid Altaany, Malik A Althobiani, Nelson Alvis-Guzman, Nelson J Alvis-Zakzuk, Yaser Mohammed Al-Worafi, Abdulmohsen Hamdan Al-Zalabani, Junaid Amin, Tarek Tawfik Amin, Saeed Amini, Kafayat Aminu, Anandhi Deva Amirtharaj, Ganiyu Adeniyi Amusa, David B Anderson, Abhishek Anil, A.H. Sruthi Anil Kumar, Aisha Ansari, Abebe Alemu Anshebo, Boluwatife Stephen Anuoluwa, Saeid Anvari, Jalal Arabloo, Jesil Mathew Aranjani, Aleksandr Y Aravkin, Demelash Areda, Benedetta Armocida, Anton A Artamonov, Francis Arthur-Holmes, Ashokan Arumugam, Mohammad Asghari-Jafarabadi, Mohammad Ashfaque, Tahira Ashraf, Muhammad Shahzad Aslam, Imad Asmar, Ömer Ataç, Mirbahador Athari, Seyyed Shamsadin Athari, Alok Atreya, Abadi Hailay Atsbaha, Julie Alaere Atta, Avinash Aujayeb, Leticia Avila-Burgos, Faseeh Bin Awais, Sana Javaid Awan, Seyyed HamidReza Ayatizadeh, Haleh Ayatollahi, Ahmad Ayed, Damilola Jeremiah Ayowole, AKM Azad, Arian Azadnia, Abrham W Azale, Alireza Azarboo, Tomasz Bączek, Andreea Corina Badache, Ashish D Badiye, Adewale Ismahil` Badru, Youngoh Bae, Nayereh Baghcheghi, Nasser Bagheri, Khlood K Baghlaf, Ruhai Bai, Atif Amin Baig, Lovenish Bains, Shankar M Bakkannavar, Yatan Pal Singh Balhara, Jose Balmori-de-la-Miyar, Khushbu Balsara, Ovidiu Constantin Baltatu, Samuel Alemu Bamboro, Juan S Barajas-Gamboa, Suzanne Lyn Barker-Collo, Amadou Barrow, Cornelia Anne Barth, Azadeh Bashiri, Rehana Basri, Mohammad-Mahdi Bastan, Abdul-Monim Batiha, Shazia Batool, Kavita Batra, Ravi Batra, Shahrzad

Bazargan-Hejazi, PhD, Neeraj Bedi, Narasimha M Beeraka, Desalegn Weldebrhan Bekru, Suleiman Ayalew Belay, Asnake Gashaw Belayneh, Enku Shiferaw Belayneh, Bashir Bello, Salaheddine Bendak, Samiun Nazrin Bente Kamal Tune, Aliyi Daba Benti, Alemshet Yirga Berhie, Mansi Sanjaykumar Bhadiyadra, Sonu Bhaskar, Arushee Bhatnagar, Sudip Bhattacharya, Jasvinder Singh Bhatti, Manpreet Singh Bhatti, Zulfiqar A Bhutta, Fengsai Bie, Corey B Bills, Abdulhakeem Binhambali, Paria Bolourinejad, Guilherme Borges, Dipan Bose, Samuel Adolf Bosoka, Alejandro Botero Carvajal, Souad Bouaoud, Soufiane Boufous, Dejana Braithwaite, Nicholas Allan Buckley, Tsion Samuel Bunare, Felix Busch, Yasser Bustanji, Senthilkumar C, Luis Alberto Cámera, Julio Cesar Campuzano Rincon, Zhong Cao, Angelo Capodici, Felix Carvalho, Ana Paula Carvalho-e-Silva, Joao Mauricio Castaldelli-Maia, Luca Cegolon, Ester Cerin, Sonia Cerrai, Sandip Chakraborty, Rama Mohan Chandika, Vijay Kumar Chattu, Anis Ahmad Chaudhary, Sirshendu Chaudhuri, Akashdeep Singh Chauhan, Akhilanand Chaurasia, Hana Chen, Patrick R Ching, Bryan Chong, Hitesh Chopra, Dinesh Kumar Choudhury, Jakir Ahmed Chowdhury, Mirza Farhana Iqbal Chowdhury, Steffan Wittrup McPhee Christensen, Isaac Sunday Chukwu, Eric Chung, Sunghyun Chung, Claudia Cosma, Stefania Curti, Omid Dadras, Hana Mahdi Dahir, Xiaochen Dai, Emanuele D'Amico, Lenards Obeng Damoah, Anh Kim Dang, Reza Darvishi Cheshmeh Soltani, Elham Davtalab Esmaeili, Bryan Gervais de Liyis, Ivan Delgado-Enciso, Andreas K Demetriades, Emina Dervišević, Aragaw Tesfaw Desale, Mitiku Desalegn, Vinoth Gnana Chellaiyan Devanbu, Devananda Devegowda, Arkadeep Dhali, Sreedhar Dharmagadda, Samath Dhamminda Dharmaratne, Bibha Dhungel, Adriana Dima, Mbekuveh Dinga-Nyoh, Vishal Diwan, Sushil Dohare, Jasmin Dönicke, Mario D'Oria, Fariba Dorostkar, Ojas Prakashbhai Doshi, Robert Kokou Dowou, Menayit Tamrat Dresse, Senbagam Duraisamy, Venkata Ravikanth Eddula, Yohannes Tekalegn Efa, Moaheda E H Eissa, Michael Ekholuenetale, Ibrahim Farahat El Bayoumy, Iman El Sayed, Mohamed Ahmed Eladl, Said El-Ashker, Iffat Elbarazi, Marwan El-Deyarbi, Hala Rashad Elhabashy, Muhammed Elhadi, Omar Abdelsadek Abdou Elmeligy, Ashraf A El-Metwally, Randa Elsheikh, Sharareh Eskandarieh, Adeniyi Francis Fagbamigbe, Ayesha Fahim, Ebenezer Gezahegn Fanta, Emerito Jose Aquino Faraon, Abdullah Farasani, Mohammad Fareed, Carla Sofia e Sá Farinha, Andre Faro, Syed Muhammad Yousaf Farooq, Nelsensius Klau Fauk, Mohammad Fayaz, Alireza Feizkhah, Ginenus Fekadu, Abdullah H Feroze, Nuno Ferreira, Aemon Berhane Fissha, Luisa S Flor, Behzad Foroutan, Matteo Foschi, Kayode Raphael Fowobaje, Richard Charles Franklin, Abdelrahman Gamil Gad, Muktar A Gadanya, Márió Gajdács, Balasubramanian Ganesh, Ezhilkugan Ganessane, Fernando Jr Barroga Garcia, Jacopo Garlasco, Zisis Gatzioufas, Ali Gerami Matin, Kalab Yigermal Gete, Mustafa Ghaderzadeh, Roya Ghafoury, Leila Ghalichi, Arin Ghamkhar, Ali Ghandili, Moein Ghasemi, Ahmad Ghashghaee, Sama Ghoba, Ali Gholamrezanezhad, Thinh Gia Gia Nguyen, Alem Abera Girmay, Alessandro Girombelli, Massimiliano Gobbo, Kimiya Gohari, Mahaveer Golechha, Pouya Goleij, Mohsen Golkar, Wei-Jie Gong, Shirisha Gooty, Shashikumar Channmgere Govindappa, Michal Grivna, Shi-Yang Guan, Giovanni Guarducci, Mohammed Ibrahim Mohialdeen Gubari, Damitha Asanga Gunawardane, Sapna Gupta, Pooja Gurnal, Reyna Alma Gutiérrez, Roberth Steven Gutiérrez-Murillo, Adrina Habibzadeh, Aryamawit Habteyesus, Shaymaa Hafedh Alkubaisy, Nguyen Hai Nam, Alazar Menbere Haile, Pritam Halder, Randah R Hamadeh, Fatimah Othman Hamarasool, Erin B Hamilton, Abdelrahman M Hamouda, Asif Hanif, Nasrin Hanifi, Mohd Anul Haq, Arief Hargono, Sara Harsini, Hamidreza Hasani, Reza Hashempour, Mohammad Hashem Hashempur, Nada Tawfig Hashim, Ibrahim Nagmeldin Hassan, Ikrama Hassan, Jeffrey J Hebert, Mohammad Heidari, Mojtaba Heydari, Matthew Hobbs, Ramesh Holla, Md Sabbir Hossain, Syed Billal Hossain, Mehdi Hosseinzadeh, Sorin Hostiuc, Junjie Huang, Ayesha Humayun, Waqar Husain, Salman Hussain, Segun Emmanuel Ibitoye, Aminu Alhassan Ibrahim, Fatma Magdi Ibrahim, Anel Ibrayeva, Olayinka Stephen Ilesanmi, Irena M Ilic, Milena D Ilic, Mustapha

Immurana, Endang Indriasih, Danish Iqbal, Bilal Irfan, Lalu Muhammad Irham, Md Rabiul Islam, Md Mojahedul Islam, Md Shahinul Islam, Chidozie Declan Iwu, Oluyinka Ajibola Iyiola, Juan S Izquierdo-Condoy, Jaison Jacob, Louis Jacob, Kathryn H Jacobsen, Ali Jadidi, Mihajlo Jakovljevic, Reza Jalilzadeh Yengejeh, Jerin James, Roland Dominic G Jamora, Balamurugan Janakiraman, Rajiv Janardhanan, Saad Javed, Talha Jawaid, Sathish Kumar Jayapal, Ruwan Duminda Jayasinghe, Achala Upendra Jayatilleke, Seogsong Jeong, Bijay Mukesh Jeswani, Ravi Prakash Jha, Joseph Johnson, Jost B Jonas, Tamas Joo, Akaninyene Paul Joseph, Nitin Joseph, Bhawana Joshi, Krupal Joshi, Charity Ehimwenma Joshua, Thenmozhi K, Zubair Kabir, Dler Hussein Kadir, Bashir Kaka, Ashish Kumar Kakkar, Leila R Kalankesh, Khalil Kalavani, Rajesh Kamath, Ramat T Kamorudeen, Jiseung Kang, Kehinde Kazeem Kanmodi, Rami S Kantar, Neeti Kapoor, Sunil Kumar Kar, Mehrdad Karajizadeh, Ibraheem M Karaye, Arman Karimi Behnagh, Samad Karkhah, Mohmed Isaqali Karobari, Faizan Zaffar Kashoo, Nehemia I Kassa, Joonas H Kauppila, Neda Kaydi, Peter Njenga Keiyoro, Yousef Saleh Khader, Morteza Abdullatif Khafaie, Ajmal Khan, Hayat Ahmad Khan, Iqra Hamid Khan, Maseer Khan, Md Abdullah Saeed Khan, Muhammad Arslan Khan, Serab Khan, Sumaiya Khan, Uzma Rahim Khan, Yusuf Saleem Khan, Zahid Khan, Srijana Khanal, Sameer Uttamaro Khasbage, Haitham Khatatbeh, Moawiah Mohammad Khatatbeh, Kavin Khatri, Hamid Reza Khayat Kashani, Afshin Khazaei, Khalid A Kheirallah, Daniel Kheradmand, Mehdi Khezeli, Milad Khorasani, Samira Khoshvaght, Jagdish Khubchandani, P Ratan Khuman, Yun Jin Kim, Adnan Kisa, Marta A Kisiel, Abdul Basith KM, Shivakumar KM, Prakash Babu Kodali, Ali-Asghar Kolahi, Farzad Kompani, Hamid Reza Koohestani, Wolyu Erkano Korma, Tapos Kormoker, Ayan Hussien Korse, Arthi S Kozhumam, Kewal Krishan, Varun Krishna, Burcu Kucuk Bicer, Mohammed Kuddus, Mukhtar Kulimbet, Emmanuel Kumah, Chandan Kumar, Dewesh Kumar, Kamal Kumar, Nithin Kumar, Pawan Kumar, Pradeep Kumar, Prakash Kumar, Radha Kumar, Rakesh Kumar, Sandeep Kumar, Sumit Kumar, Tushar Kumar, Om P Kurmi, Asep Kusnali, Dian Kusuma, John Paul Kuwornu, Ville Kytö, Pallavi L C, Carlo La Vecchia, Muhammad Awwal Ladan, Lucie Laflamme, Chandrakant Lahariya, Timo Lajunen, Balzhan Lakanova, Tri Laksono, Tamás Lantos, Savita Lasrado, Mahrukh Latif, Paolo Lauriola, Caterina Ledda, Ka Yiu Lee, Munjae Lee, Seung Won Lee, Wei-Chen Lee, Yo Han Lee, Ja-Ho Leigh, Vasileios Leivaditis, Dawit Alemu Lemma, Ming-Chieh Li, Xiaopan Li, Yan Li, Zhengrui Li, Ambreen Liaqat, Xuefeng Liu, José Francisco López-Gil, Platon D Lopukhov, Surbala Devi Lourembam, Abhilash Ludhiadch, Angelina M Lutambi, Miltiadis D Lytras, Ellina Lytvyak, Zheng Feei Ma, Monika Machoy-Rakoczy, Firoozeh Madadi, Ahmed A Madfa, Hassan Magdy Abd El Razek, Mohammed Magdy Abd El Razek, Sasikumar Mahalingam, Samatar Abshir Mahamed, Preeti Maharjan, Nozad Hussein Mahmood, Azeem Majeed, Mostafa Majidnia, Igor Lima Maldonado, Deborah Carvalho Malta, Lokesh Manjani, Ramon Martinez-Piedra, J Jenifer Florence Mary, Roy Rillera Marzo, Sammer Marzouk, Sharareh Massahi, Yasith Mathangasinghe, Neeta Mathur, Khurshid A Mattoo, Steven M McPhail, Tesfahun Mekene Meto, Agezegn Asegid Mekonnen, Tesfaye Hambisa Mekonnen, Tadesse Belayneh Melkie, Osires de Medeiros Melo Neto, Walter Mendoza, Godfred Antony Menezes, Ritesh G Menezes, Emiru Ayalew Mengistie, Anusha Sultan Meo, Tuomo J Meretoja, Tomislav Mestrovic, Chamila Dinushi Kukulege Mettananda, Sachith Mettananda, Mohamed M M Metwally, Bartosz Miazgowski, Ted R Miller, Giuseppe Minervini, Long Chiau Ming, Prasanna Mithra, Chaitanya Mittal, Dhruti Modi, Lia Solomon Mogus, Mona Gamal Mohamed, Nouh Saad Mohamed, Khabab Abbasher Hussien Mohamed Ahmed, Taj Mohammad, Mokhtar Mohammadi, Abdollah Mohammadian-Hafshejani, Shafiu Mohammed, Mohammad Mohseni, Ali H Mokdad, Mohammad Ali Moni, Julio Cesar Montañez, Marie Chantel Montás, Rafael Silveira Moreira, Dimitrios Moris, Mahdis Morovvati, Shane Douglas Morrison, Mahmoud M Morsy, Md Shihab Mostafa, Rohith Motappa, Kimia Mozahheb Yousefi, Sumaira Mubarik, Shiv Kumar Mudgal, Jibran Sualeh Muhammad,

Mubarak Muhammad, Sumoni Mukherjee, Francesk Mulita, Malaisamy Muniyandi, Efren Murillo-Zamora, Satta Muthu Murugan, Balaganapathy Muruganantham, Abdisalam Hassan Muse, David Musoke, Sathish Muthu, Ayoub Nafei, Ganesh R Naik, Gopal Nambi, Vinay Nangia, Shumaila Nargus, Abdulqadir J Nashwan, Mahmoud Nassar, Zuhair S Natto, Javaid Nauman, Zakira Naureen, Samidi Nirasha Kumari Navaratna, Biswa Prakash Nayak, Kinjal Nayak, Iffat Nayila, Javad Nazari, G Takop Nchanji, Nini Asfaw Negash, Ionut Negoi, Samata Nepal, Cuong Tat Nguyen, Hien Thu Nguyen, Trang Nguyen, Taxiarchis Konstantinos Nikolouzakis, Ali Nikoobar, Peishan Ning, Dina Nur Anggraini Ningrum, Vikram Niranjan, Richmond Nketia, Shuhei Nomura, Chisom Adaobi Nri-Ezedi, Jean Claude Nshimiyimana, Chimezie Igwegbe Nzoputam, Ogochukwu Janet Nzoputam, Bogdan Oancea, Kwabena Obiri - Yeboah, Fabio Massimo Oddi, Ismail A Odetokun, Felix Akpojene Ogbo, Abiola Ogunkoya, Olusegun Olatunji Ojedoyin, Hassan Okati-Aliabad, Osaretin Christabel Okonji, Oluwaseyi Isaiah Olabisi, Andrew T Olagunju, Abdulhakeem Abayomi Olorukooba, Oluseye Olalekan Oludoye, Ahmed Omar Bali, Goran Latif Omer, Michal Ordak, Hans Orru, Esteban Ortiz-Prado, Augustus Osborne, Alaa Oteir, Adrian Otoiu, Amel Ouyahia, Mayowa O Owolabi, Tope Oyelade, Mahesh P A, Kevin Pacheco-Barrios, Jagadish Rao Padubidri, Ashok Pandey, Sanjib Raj Pandey, Seithikurippu R Pandi-Perumal, Georgios D Panos, Ilias Papadimopoulos, Evangelos Pappas, Shahina Pardhan, Chulwoo Park, Eun-Cheol Park, Pragnesh Parmar, Ava Pashaei, Maja Pasovic, Roberto Passera, Bhargav Umeshchandra Patel, Hemal M Patel, Kunal Nitinkumar Patel, Niki Rut Patel, Sangram Kishor Patel, Vipin Patidar, Shankargouda Patil, Mohamed Anas Patni, Dimitrios Patoulias, Uttam Paudel, Shrikant Pawar, Hamidreza Pazoki Toroudi, Arun Mitra Peddireddy, Amy E Peden, Jarmila Pekarcikova, Veincent Christian Filipino Pepito, Prince Peprah, Daniela A Perez-Chadid, Simone Perna, Anil K Philip, Julian David Pillay, Mapa M Prabhath N Piyasena, Shiny PJ, Evgenii Plotnikov, Roman V Polibin, Reza Pourbabaki, Farzad Pourghazi, Mohsen Poursadeqiyan, Naeimeh Pourtaheri, Sergio I Prada, Harsh Priya, Muhammad Usman Qamar, Xiang Qi, Mohammad Qodrati, Nuzul Qur'aniati, Basuki Rachmat, Venkatraman Radhakrishnan, Amir Rafe, Ibrar Rafique, Yashpal Singh Raghav, Pracheth Raghuveer, Sajjad Rahimi, Vafa Rahimi-Movaghar, Firman Suryadi Rahman, Fryad Majeed Rahman, Mohammad Hifz Ur Rahman, Mosiur Rahman, Muhammad Aziz Rahman, Amir Masoud Rahmani, Amita Rai, Pramila Rai, Sunil Kumar Raina, Judah Rajendran, Shaman Rajindrajith, Pushp Lata Rajpoot, Mahmoud Mohammed Ramadan, Majed Ramadan, Kadar Ramadhan, Shakthi Kumaran Ramasamy, Balaji Ramraj, Lokesh Rana, Nemanja Rancic, Asad Gul Rao, Kumuda Rao, Mithun Rao, Sowmya J Rao, Prateek Rastogi, Devarajan Rathish, Rashita Ravi, Salman Rawaf, Karthik Rayapureddi, Ateeq Rehman, Rainer Reile, Mina Rezaei, Nazila Rezaei, Mohsen Rezaeian, Moattar Raza Rizvi, Jefferson Antonio Buendia Rodriguez, Leonardo Roever, Ravi Rohilla, Chanapong Rojanaworarit, Moustaq Karim Khan Rony, Himanshu Sekhar Rout, Bedanta Roy, Polani Rubeshkumar, Devarajulu Reddy S, Manjula S, Aly M A Saad, Siamak Sabour, Fatemeh Sadeghi-Ghyassi, Mohammad Reza Saeb, Mohd Saeed, Umar Saeed, Mehdi Safari, Sher Zaman Safi, Amene Saghazadeh, Fatemeh Saheb Sharif-Askari, Narjes Saheb Sharif-Askari, Amirhossein Sahebkar, Vaibhav Sahni, Durgesh Prasad Sahoo, Zahra Saif, Morteza Saki, Joseph W Sakshaug, Payman Salamati, Afeez Abolarinwa Salami, Samreen Saleem, Sohrab Salimi, Jayami Eshana Samaranayake, Yoseph Leonardo Samodra, Vijaya Paul Samuel, Fasineh Shekuba Samura, Abdallah M Samy, Rama Krishna Sanjeev, Milena M Santric-Milicevic, Aswini Saravanan, Yaser Sarikhani, Mohammad Sarmadi, Sachin C Sarode, Michele Sassano, Mukesh Kumar Sathya Narayanan, Maheswar Satpathy, Jennifer Saulam, Ganesh Kumar Saya, Christophe Schinckus, David C Schwebel, John W Scott, Hamidreza Seifmanesh, Siddharthan Selvaraj, Mohammad H Semreen, Subramanian Senthilkumaran, Francesco Sessa, Yashendra Sethi, Ahmed Nabil Shaaban, Shipraa Arpan Shah, Sweni Shah, Shazlin Shaharudin, Amira A Shaheen, Samiah Shahid, Moyad Jamal Shahwan,

Masood Ali Shaikh, Muhammad Aaqib Shamim, Amir Hossein Shams, Mehran Shams-Beyranvand, Alfiya Shamsutdinova, Dan Shan, Mohd Shanawaz, Amin Sharifan, Javad Sharifi Rad, Prof. Fariborz Sharifian Jazi, Bhoopesh Kumar Sharma, Bunty Sharma, Jyoti Sharma, Manoj Sharma, Ravi Kumar Sharma, Saurab Sharma, Vishal Sharma, Bereket Beyene Shashamo, Shamee Shastry, Ramzi Shawahna, Samrawit Shawel, Maryam Shayan, Ali Sheidaei, Nishat Ahmed Sheikh, Rahim Ali Sheikhi, Jiabin Shen, Samendra P Sherchan, Mahabalesh Shetty, Pavanchand Shetty, Premalatha K Shetty, Suraj S Shetty, Aminu Shittu, Nathan A Shlobin, Sina Shool, Seyed Afshin Shorofi, Kerem Shuval, Md. Kaoser Bin Siddique, Gustavo Correia Basto da Silva, Padam Prasad Simkhada, Akanksha Singh, Harmanjit Singh, Paramdeep Singh, Poornima Suryanath Singh, Surendra Singh, Surjit Singh, Natia Skhvitaridze, Valentin Yurievich Skryabin, Bogdan Socea, Md Salman Sohel, Solikhah Solikhah, Yizhe Song, Lokasani Vijaya Sree, Vignes Anand Srinivasalu, Muhammad Haroon Stanikzai, Paschalis Steiropoulos, Sebastian Straube, Chen-Yang Su, Omer Subasi, Vetriselvan Subramaniyan, Agus Sudaryanto, Nathan Suga, Thitiporn Sukaew, Surajo Kamilu Sulaiman, Mark J M Sullman, Jing Sun, Suraj Sundaragiri, Katharina S Sunnerhagen, Chandan Kumar Swain, Lukasz Szarpak, Sree Sudha T Y, Rafael Tabarés-Seisdedos, Seyyed Mohammad Tabatabaei, Celine Tabche, Ramin Tabibi, Moslem Taheri Soodejani, Iman M Talaat, Mircea Tampa, Ingan Ukur Tarigan, Mengistie Kassahun Tariku, Anika Tasnim, Aigul Yelgondiyevna Tazhiyeva, Nebyu Lakew Tefera, Emre Tel, Gebremaryam Temesgen, Mohamad-Hani Temsah, Reem Temsah, Azimeraw Arega Tesfu, Enoch Teye-Kwadjo, Dejina Thapa, Rekha Thapar, Ismaeel Tharwat, Jeevarathinam Thirumalai, Benson Thomas M, Harshal R Thube, Jansje Henny Vera Ticoalu, Madi Tleshev, Santo Imanuel Tonapa, Arole Darwin Touko, Marcos Roberto Tovani-Palone, Thang Huu Tran, Nguyen Tran Minh Duc, Domenico Trico, Mike Tuffour Amirikah, Lawrence Sena Tuglo, Munkhtuya Tumurkhuu, Ermias A Turuse, Biruk Shalmeno Tusa, Irfan Ullah, Saeed Ullah, Srikanth Umakanthan, Lawan Umar, Bhaskaran Unnikrishnan, Carolyn Anne Unsworth, Selamu Amanuel Untiso, Era Upadhyay, Sheela Upendra, Jibrin Sammani Usman, Rakesh V R, Asokan Govindaraj Vaithinathan, Pascual R Valdez, Tommi Juhani Vasankari, Narayanaswamy Venketasubramanian, Akshaya Kumar Verma, Massimiliano Veroux, Georgios-Ioannis Verras, Victor E Villalobos-Daniel, Tuan Vinh, Francesco S Violante, Vasily Vlassov, Yasir Waheed, Arvinder Wander, Wanzhou Wang, Wei Wang, Yanzhong Wang, Apichai Wattanapisit, Anthony Ike Wegbom, Fei-Long Wei, Andrea Werdecker, Taweewat Wiangkham, Dakshitha Praneeth Wickramasinghe, Faustine Williams, Andrew Awuah Wireko, Wanqing Xie, Patlolla Sriram Yadav, Vikas Yadav, Musa Yahaya, Saba Yahoo, Eesha Yaqoob, Sanni Yaya, Pengpeng Ye, Ali Cem Yekdeş, Siyan Yi, Arzu Yiğit, Vahit Yiğit, S Yogeshkumar, Dong Keon Yon, Naohiro Yonemoto, Mustafa Younis, Yong Yu, Siddhesh Zadey, Mubashir Zafar, Emilia Zainal Abidin, Dilmurat Zairov, Aurora Zanghì, Iman Zare, Michael Zastrozhin, Saad Zbiri, Mohammed G M Zeariya, Haijun Zhang, Yunquan Zhang, Zhi-Jiang Zhang, Claire Chenwen Zhong, Hao Zhou, Abzal Zhumagaliuly, Yossef Teshome Zikarg, Sa'ed H Zyoud, Shaher H Zyoud, Christopher J L Murray, Simon I Hay†, Marie Ng†, and Kanyin Liane Ong†.

*Co-first authors

†Co-senior authors

Affiliations

NUS-IHME Global Burden of Disease Research Centre, Yong Loo Lin School of Medicine (S C C van der Lubbe PhD, D Aluquin MPH, Y Zhou MD, M Ng PhD), Department of Medicine (B Chong MBBS), Yong Loo Lin School of Medicine (Prof N Venketasubramanian MSc), Saw Swee Hock School of Public Health (Prof S Yi PhD), National University of Singapore, Singapore, Singapore; Institute for Health Metrics and Evaluation (M E Moberg MS, H Lenox MSPH, A Y Aravkin PhD, X Dai PhD, Prof S D Dharmaratne MD, L S Flor PhD, S Ghoba MS, E B Hamilton MPH, T Mestrovic PhD, Prof A H Mokdad PhD, M Pasovic MEd, J W Scott MD, Prof C J L Murray DPhil, Prof S I Hay DSc, M Ng PhD, K L Ong PhD), Department of Applied Mathematics (A Y Aravkin PhD), Department of Health Metrics Sciences, School of Medicine (A Y Aravkin PhD, X Dai PhD, Prof S D Dharmaratne MD, L S Flor PhD, Prof A H Mokdad PhD, Prof C J L Murray DPhil, Prof S I Hay DSc), School of Health Systems and Public Health (C Iwu MPH), University of Washington, Seattle, WA, USA; Medicine Department (A MD), Rawalpindi Medical University (F B Awais MBBS), Rawalpindi Medical University, Rawalpindi, Pakistan; Amity Institute of Public Health and Hospital Administration (Prof B A PhD), Amity University Noida, Uttar Pradesh, India; Department of Nursing (H Aalruz PhD), Al Zaytoonah University of Jordan, Amman, Jordan; School of Health and Life Sciences (U O Abaraogu PhD), University of the West of Scotland, Paisley, UK; Department of Medical Rehabilitation (U O Abaraogu PhD), University of Nigeria Nsukka, Enugu, Nigeria; Department of Medical-Surgical Nursing (Z Abbasi Dolatabadi PhD), Advanced Diagnostic and Interventional Radiology Research Center (ADIR) (H Abbastabar PhD), School of Medicine (A Azarboo MD, M Morovvati MD, M Qodrati MD), Non-communicable Diseases Research Center (M Bastan MD, N Rezaei MD), Multiple Sclerosis Research Center (Prof S Eskandarieh PhD), Neurology Department of Imam Khomeini Hospital (M Ghasemi MD), Children's Medical Center (Prof F Kompani MD), Sina Trauma and Surgery Research Center (Prof V Rahimi-Movaghar MD, Prof P Salamati MD, S Shool MD), Research Center for Immunodeficiencies (A Saghazadeh MD), General Medicine Department (H Seifmanesh MD), School of Public Health (A Sheidaei PhD), Tehran University of Medical Sciences, Tehran, Iran; Health Research Centre (Prof S I Abdelwahab PhD), College of Nursing and Health Sciences (H Albargi PhD, M Shanawaz MD), Clinical Nutrition Department (R M Chandika PhD), Department of Public Health (S Dohare MD, P Rajpoot PhD), Department of Medical Laboratory Technology (A Farasani PhD), Epidemiology Program (M Khan MD), Department of Prosthetic Dental Sciences (K A Mattoo MD), Department of Mathematics (Y Raghav PhD), Jazan University, Jazan, Saudi Arabia (Prof N Bedi MD); Department of Medicine (Prof M Abdoun PhD), Department of Health (Prof M Abdoun PhD), University of Setif Algeria, Sétif, Algeria; Health Research Institute (Z Abdrakhmanova PhD, M Kulimbet MSc, B Lakanova MD), Al Farabi Kazakh National University, Almaty, Kazakhstan; Community and Maternity Nursing Unit (D M Abdulah MPH), University of Duhok, Duhok, Iraq; Department of Physiotherapy (A Abdullahi PhD), Department of Community Medicine (Prof M A Gadanya MD), Department of Nursing Science (M Ladan PhD), Bayero University Kano, Kano, Nigeria; Department of Physiotherapy (A Abdullahi PhD), Federal University Wukari, Wukari, Nigeria; Department of Research (T Abdul-Rahman MD), Toufik's World Medical Association, Sumy, Ukraine; School of Medicine (T B Abebe MD), Salale University, Fitche, Ethiopia; Department of Emergency Medicine (A Abedi MD), Department of Immunology (S Athari PhD), Department of Critical Care and Emergency Nursing (N Hanifi PhD), Zanjan University of Medical Sciences, Zanjan, Iran; School of Health Sciences (H M Abeywickrama PhD), Niigata University, Niigata, Japan; Department of Community Medicine (Prof O Abiodun MPH), Babcock University, Ilishan-Remo, Nigeria; Department of Family and Community Health (R G Aboagye MPH), School of Medicine (A F Adamu MD), Department of Epidemiology and Biostatistics (S A Bosoka MPhil, R K Dowou MPhil), Institute of Health Research (M

Immurana PhD), University of Health and Allied Sciences, Ho, Ghana; School of Population Health (R G Aboagye MPH), Centre for Social Research in Health (I Y Addo PhD), Transport and Road Safety (TARS) Research Centre (S Boufous PhD), The George Institute for Global Health (P Ye PhD), University of New South Wales, Sydney, NSW, Australia; Department of Research and Development (A T Aborode MSc), Healthy Africans Platform, Ibadan, Nigeria; Zoology Department (N M Abourashed PhD), Benha University, Benha, Egypt; Department of Physical Pharmacy and Pharmacokinetics (M Abouzid PharmD), Poznan University of Medical Sciences, Poznan, Poland; College of Medicine and Health Sciences (T A Abrha MD), Adigrat University, Adigrat, Ethiopia; Department of Anesthesiology (D Abtahi MD, S Salimi MD), Department of Epidemiology (Prof A Ahmadi PhD, Prof S Sabour PhD), National Nutrition and Food Technology Research Institute (Prof M Ajami PhD), Department of Oral and Maxillofacial Surgery (M Alam MSc, M Golkar MSc), Department of Health Information Technology and Management (M Ghaderzadeh PhD), Department of Health Management, Policy and Economics (R Hashempour PhD), Department of Neurosurgery (H Khayat Kashani MD), Social Determinants of Health Research Center (Prof A Kolahi MD, A Nikoobar MD), Anesthesiology Research Center (F Madadi MD), Research Institute for Health Sciences and Environment (M Safari PhD), Department of Health in Disaster & Emergency (M Safari PhD), Ophthalmic Research Center (ORC) (M Shayan MD), Shahid Beheshti University of Medical Sciences, Tehran, Iran; Primary Care Nursing Department (F H A Abuadas PhD), Al-Ahliyya Amman University, Amman, Jordan; Graduate School of Public Health (A K Abubakar MPH), St. Luke's International University, Tokyo, Japan; Division of Population Data Science (A K Abubakar MPH), National Cancer Center, Tokyo, Japan; Clinical Pharmacy and Therapeutics Department (Prof R K Abu-Farha PhD), Applied Science Private University, Amman, Jordan; Department Pharmacy Practice and Pharmacotherapeutics (A Y Abuhelwa PhD), College of Pharmacy (S M Aleidi PhD, Prof M H Semreen PhD), College of Dental Medicine (M A Alsaegh PhD), Research Institute for Medical and Health Sciences (M A Alsaegh PhD), Department of Physiotherapy (A Arumugam PhD), Department of Basic Biomedical Sciences (Prof Y Bustanji PhD), Department of Basic Medical Sciences (M A Eladl PhD), Department of Clinical Sciences (Prof M M Ramadan PhD, N Saheb Sharif-Askari PhD, Prof I M Talaat PhD), Sharjah Institute of Medical Sciences (F Saheb Sharif-Askari PhD), Research Institute of Medical & Health Sciences (Prof M H Semreen PhD), University of Sharjah, Sharjah, United Arab Emirates; Clinical Trials Unit (H J Abukhadijah MPH), Department of Pediatric Surgery (A Alansari MD), Al Wakra Hospital (A Alshami BSc), Nursing & Midwifery Research Department (NMRD) (A J Nashwan PhD), Hamad Medical Corporation, Doha, Qatar; College of Health Sciences (Prof N M Abu-Rmeileh PhD), Office of Vice President for Medical and Health Sciences (D Abushanab PhD), Public Health Department (M F Alam PhD), College of Nursing (Prof F N Alhalaiqa PhD), College of Dental Medicine (Prof K Ali PhD), Qatar University, Doha, Qatar; Birzeit University, Ramallah, Palestine (Prof N M Abu-Rmeileh PhD); Institute of International Health (A Acharya MD), Charité Medical University Berlin, Berlin, Germany; Department of Research (K Acharya MPH), New ERA, Kathmandu, Nepal; School of Medicine (I Y Addo PhD), Faculty of Medicine and Health (Prof N A Buckley MD), School of Health Science (A Carvalho-e-Silva PhD), University of Sydney, Sydney, NSW, Australia (Prof E Pappas PhD); Department of Microbiology (T A Adebisi BSc), Ladoke Akintola University, Osogbo, Nigeria; NMC Healthcare (T A Adebisi BSc), Independent Consultant, Sharjah, United Arab Emirates; Department of Pediatrics (B O Adebiyi PhD), University of Calgary, Calgary, AB, Canada; School of Public Health (B O Adebiyi PhD), University of the Western Cape, Bellville, South Africa; Department of Immunology (K A Adedokun MSc), Roswell Park Comprehensive Cancer Center, Buffalo, NY, USA; Graduate Program Division (K A Adedokun MSc), University at Buffalo, Buffalo, NY, USA; Department of Obstetrics and Gynecology (V Adekanmbi PhD),

Department of Family Medicine (W Lee PhD), University of Texas Medical Branch, Galveston, TX, USA; Department of Family Medicine (O T Adeleke MD), Bowen University Hospital (D E Ajala BNSc), College of Health Sciences (O I Olabisi PhD), Bowen University, Iwo, Nigeria; Department of Family Medicine (O T Adeleke MD), Operating Theatre (D E Ajala BNSc), Bowen University Teaching Hospital, Ogbomoso, Nigeria; Department of Health Informatics (A G Adepoju MD), Indiana University Indianapolis, Indianapolis, IN, USA; Slum and Rural Health Initiative Research Academy (M A Adesina BPT), Slum and Rural Health Initiative, Ibadan, Nigeria; Department of Physiotherapy (M A Adesina BPT), Department of Sociology (K Aminu PhD), Department of Anesthesia and Intensive Care (A I Badru Fellow), Department of Epidemiology and Medical Statistics (A F Fagbamigbe PhD), Department of Health Promotion and Education (S Ibitoye PhD, A Ogunkoya MPH), College of Medicine (O I Olabisi PhD), Department of Medicine (Prof M O Owolabi DrM), University of Ibadan, Ibadan, Nigeria; Department of Health Administration and Education (P O Adoma PhD), University of Education Winneba, Winneba, Ghana; School of Arts and Social Sciences (O Adonteng-Kissi PhD), Edith Cowan University, Bunbury, WA, Australia; Australian Centre for Health Services Innovation (C Afoakwah PhD, J Kuwornu PhD, Prof S M McPhail PhD), Queensland University of Technology, Kelvin Grove, QLD, Australia; Jamieson Trauma Institute (C Afoakwah PhD), Metro North Health, Herston, QLD, Australia; Technical Services Directorate (A A Afolabi MPH), MSI Nigeria Reproductive Choices, Abuja, Nigeria; School of Science (E Afrifa-Yamoah PhD), Edith Cowan University, Perth, WA, Australia; Department of Plastic Surgery (A P Afsar MBBS), Division of Infectious Disease (F Pourghazi MD), Mayo Clinic, Rochester, MN, USA; Social Determinants of Health Research Center (M Afshari PhD, H Koohestani PhD), Department of Nursing (N Baghcheghi PhD), Saveh University of Medical Sciences, Saveh, Iran; Department of Community Medicine (Prof S Afzal PhD), King Edward Memorial Hospital, Lahore, Pakistan; Department of Public Health (Prof S Afzal PhD), Public Health Institute, Lahore, Pakistan; University of Texas Health Science Center at San Antonio (V K Aggarwal MD), University of Texas, San Antonio, TX, USA; Department of Orthopedics and Sports Medicine (M Aghaalikhani MD), Boston Children's Hospital, Boston, MA, USA; Department of Global Health and Population (M C Montás MPH), Department of Physical Medicine and Rehabilitation (K Pacheco-Barrios MD), Department of Ophthalmology (M Shayan MD), Harvard T.H. Chan School of Public Health (M Tuffour Amirikah MD), Harvard University, Boston, MA, USA (M Aghaalikhani MD); Operations Department (S W Agordoh MPH), World Vision, Kete Krachi, Ghana; Department of Anesthesia (S Agrawal MD), ABVIMS and DR RAM MANOHAR LOHIA Hospital, New Delhi, India; Department of Public Health Sciences (W Agyemang-Duah PhD), Queen's University, Kingston, ON, Canada; Department of Human Nutrition & Dietetics (A Ahmad PhD), NUST School of Health Sciences (A Meo MPH), National University of Science and Technology (NUST), Islamabad, Pakistan; Department of Health Sciences (A Ahmad PhD), University of York, York, England; College of Medicine (A Ahmad PhD), Department of Family Medicine (Prof M A Alammar PhD), Shaqra University, Shaqra, Saudi Arabia; School of Medicine and Psychology (D Ahmad PhD), Australian National University, Canberra, ACT, Australia; Health Research Institute (D Ahmad PhD, Prof N Bagheri PhD), University of Canberra, Canberra, NSW, Australia; King Khaled Eye Specialist Hospital and Research Center, Riyadh, Saudi Arabia (Prof K Ahmad PhD); School of Nursing (Prof M M Ahmad PhD), University of Jordan, Amman, Jordan; Department of Health and Biological Sciences (S Ahmad PhD), Abasyn University, Peshawar, Pakistan; Department of Natural Sciences (S Ahmad PhD), Lebanese American University, Beirut, Lebanon; School of Public Health (T Ahmad PhD), Zhejiang University, Hangzhou, China; Department of Community Health Sciences (T Ahmad PhD), Sohail University, Karachi, Pakistan; Department of Epidemiology and Biostatistics (Prof A Ahmadi PhD), Community-Oriented Nursing Midwifery Research Center (M Heidari

PhD), Modeling in Health Research Center (A Mohammadian-Hafshejani PhD), Department of Health in Disasters and Emergencies (R Sheikhi BHlthSci), Shahrekord University of Medical Sciences, Shahrekord, Iran; Department of Pharmacy Practice (A Ahmed PhD), Riphah Institute of Pharmaceutical Sciences, Islamabad, Pakistan; Division of Infectious Diseases and Global Public Health (IDGPH) (A Ahmed PhD), University of California San Diego, San Diego, CA, USA; Institute of Endemic Diseases (A Ahmed PhD), Department of Oral Rehabilitation (N T Hashim PhD), Department of Medicine (I Hassan MD), Faculty of Medicine (K Mohamed Ahmed MD), University of Khartoum, Khartoum, Sudan; Pan-African One Health Institute (PAOHI), Kigali, Rwanda (A Ahmed PhD); Department of Biosciences (H Ahmed PhD), COMSATS Institute of Information Technology, Islamabad, Pakistan; College of Nursing (M S Ahmed MSc), Department of Computer and Information Science (M Haq PhD), Department of Physical Therapy and Health Rehabilitation (Prof F Z Kashoo PhD), Majmaah University, Al Majmaah, Saudi Arabia; Mansoura University Hospitals (M Ahmed MBBCh), Mansoura University Hospital, Mansoura, Egypt; College of Medicine and Public Health (M B Ahmed PhD, G R Naik PhD), Department of Nursing and Health Sciences (S Shorofi PhD), Flinders University, Adelaide, SA, Australia; Faculty of Public Health (M B Ahmed PhD, N L Tefera MSc), Jimma University, Jimma, Ethiopia; Maternal and Child Health Division (MCHD) (S Ahmed MDS), International Centre for Diarrhoeal Disease Research, Bangladesh, Dhaka, Bangladesh; Department of Radiology (N Akhoundi MD), Diagnostic Radiology Department (N Akhoundi MD), Children's Hospital of Philadelphia, Philadelphia, PA, USA; Department of Communicable Diseases (S Al Awaidy MSc), Ministry of Health, Muscat, Oman; Middle East, Eurasia, and Africa Influenza Stakeholders Network, Muscat, Oman (S Al Awaidy MSc); Division of Public Health Sciences (S Al Hasan PhD), Washington University in St. Louis, St. Louis, MO, USA; School of Public Health (M A M Al Zoubi PhD), Management Policy and Community Health (J A Atta MPH), University of Texas, Houston, TX, USA; Health Rehabilitation Sciences Department (Prof S S Alabdulwahab PhD), Pediatric Intensive Care Unit (Prof M Temsah MD), King Saud University, Riyadh, Saudi Arabia; Murdoch Business School (K Alam PhD), Murdoch University, Perth, WA, Australia; Preventive Dentistry Department (Prof M K Alam PhD), College of Nursing (Prof A F Alsharari PhD), College of Medicine (R Basri PhD), Jouf University, Sakaka, Saudi Arabia; Deanship of Preparatory Year and Supporting Studies (Prof S El-Ashker PhD), Department of Pathology (Prof R G Menezes MD), Imam Abdulrahman Bin Faisal University, Dammam, Saudi Arabia (F M Alanezi PhD); Department of Clinical Pharmacy (F Y Al-Ashwal PhD), Al-Ayen Iraqi University, Thi-Qar, Iraq; Department of Clinical Pharmacy and Pharmacy Practice (F Y Al-Ashwal PhD), University of Science and Technology, Sana'a, Yemen; Department of Community and Mental Health (Prof M Albashtawy PhD), Al Al-Bayt University, Mafraq, Jordan; School of Pharmacy (S M Aleidi PhD, Prof Y Bustanji PhD), The University of Jordan, Amman, Jordan; Department of Adult Health (A Alhassan MS), University of Cape Coast, Cape Coast, Ghana; Faculty of Dentistry (A Alhumaidi DDS), Ibn Al-Nafis University for Medical Sciences, Sana'a, Yemen; Department of Oral and Maxillofacial Surgery (A Alhumaidi DDS), Xi'an Jiaotong University, Xi'an, China; Information Science Department (D Alhuwail PhD), Kuwait University, Sabah Alsalem University City, Kuwait; Health Informatics Unit and Geohealth Lab (D Alhuwail PhD), Dasman Diabetes Institute, Dasman, Kuwait; Department of Statistics and Operations Research (I Ali PhD), Aligarh Muslim University, Aligarh, India; Department of Physical Therapy (M Ali PhD), Department of Medical Surgical Nursing (S A Alkubati PhD), Department of Public Health (B Alsaif PhD, M G M Zeariya PhD), Physiotherapy Department (J Amin MSc, A Ansari PhD), College of Applied Medical Sciences (S C Govindappa PhD), College of Medicine (Y S Khan MD), Department of Biochemistry (Prof M Kuddus PhD), Department of Health Management (R Kumar PhD), College of Dentistry (Prof A A Madfa PhD), Department of Biology (Prof M Saeed PhD), Department of

Family and Community Medicine (M Zafar PhD), University of Hail, Hail, Saudi Arabia; Department of Medical Rehabilitation (Physiotherapy) (M U Ali PhD), University of Maiduguri, Maiduguri, Nigeria; Nethersole School of Nursing (M U Ali PhD), Faculty of Medicine (J Huang MD), Jockey Club School of Public Health and Primary Care (C Zhong PhD), The Chinese University of Hong Kong, Hong Kong, China; Institute of Health and Wellbeing (S M Alif PhD), Federation University Australia, Melbourne, VIC, Australia; School of Public Health and Preventive Medicine (S M Alif PhD, Prof M Asghari-Jafarabadi PhD, P Maharjan MPH, P Rai PhD), General Practice (T H Mekonnen MPH), Monash University, Melbourne, VIC, Australia; Department of Clinical and Community Pharmacy (Prof S W Al-Jabi PhD, Prof S H Zyoud PhD), Faculty of Medicine and Health Science (A A Shaheen PhD), Department of Physiology, Pharmacology, and Toxicology (Prof R Shawahna PhD), An-Najah National University, Nablus, Palestine; Family and Community Medicine Department (M S Aljohani MD), Qassim University, Al Qassim, Saudi Arabia; Department of Public Health and Community Medicine (Prof S M Aljunid PhD), International Medical University, Kuala Lumpur, Malaysia; International Centre for Casemix and Clinical Coding (Prof S M Aljunid PhD), National University of Malaysia, Bandar Tun Razak, Malaysia; Department of Allied Medical Sciences (Z I Alkhatib MSc, A Alrawashdeh PhD), Faculty of Medicine (Prof M Z Allouh PhD), Department of Public Health (Prof Y S Khader PhD, Prof K A Kheirallah PhD), Department of Allied Health Sciences (A Oteir PhD), Jordan University of Science and Technology, Irbid, Jordan; Faculty of Medicine (H Alkousheh MD), The Hashemite University, Zarqa, Jordan; Department of Medical Laboratories, College of Applied Medical Sciences (Prof K Allemailem PhD), Qassim University, Saudi Arabia, Buraydah, Saudi Arabia; Department of Anatomy (Prof M Z Allouh PhD), Institute of Public Health (I Elbarazi DrPH, Prof M Grivna PhD), Pharmacology and Therapeutic Department (M El-Deyarbi PhD), College of Medicine and Health Sciences (Y Kim PhD, J Nauman PhD), United Arab Emirates University, Al Ain, United Arab Emirates; Faculty of Nursing (W T Almagharbeh PhD), Applied College (A A Baig PhD), University of Tabuk, Tabuk, Saudi Arabia; Independent Consultant, Amman, Jordan (S Al-Marwani MSc); Department of Medical Laboratories, College of Applied Medical Sciences (Prof A Almatroudi PhD), Qassim University, Buraydah, Saudi Arabia; Department of Medicine (J U Almazan PhD), Nazarbayev University, Astana, Kazakhstan; College of Nursing (K Almugheed PhD), Riyadh Elm University, Riyadh, Saudi Arabia; Department of Clinical Nursing (M M Alnaeem PhD), Al-Zaytoonah University of Jordan, Amman, Jordan; Department of Physiotherapy (F S Alotibi PhD), Taif University, Taif, Saudi Arabia; Department of Microbiology, College of Medicine (Prof M Al-Ouqaili PhD, Prof S Hafedh Alkubaisy PhD), University of Anbar, Ramadi, Anbar Governorate, Iraq; Department of Respiratory Care (J S Alqahtani PhD), Prince Sultan Military College of Health Sciences, Dammam, Saudi Arabia; Prince Sultan Military College of Health Sciences (S A S Alqahtani PhD), Saudi Ministry of Defence, Dhahran, Saudi Arabia; Department of Nursing (I Alrimawi PhD), Transplant Institute (D Moris PhD), Georgetown University, Washington, DC, USA; Macro-Fiscal Policy Department (S M Alrousan PhD), Ministry of Finance, Dubai, United Arab Emirates; Department of Emergency Medicine (M A Alsabri MD), Department of Pharmacology (A S Al-Shami MS), Sana'a University, Sanaa, Yemen; Pediatric Emergency Medicine Department (M A Alsabri MD), Drexel University, Philadelphia, PA, USA; College of Health and Life Science (A Alshami BSc), Hamad Bin Khalifa University, Doha, Qatar; Department of Basic Medical Sciences (A S Al-Shami MS), Universiti Malaysia Sarawak, Kota Samarahan, Malaysia; Yarmouk University (Z Altaany PhD), Faculty of Medicine, Irbid, Jordan; Higher Collage of Technology (Z Altaany PhD), Higher Collage of Technology, Abu Dhabi, United Arab Emirates; Department of Respiratory Therapy (M A Althobiani PhD), Pediatric Dentistry Department (K K Baghlaf PhD), Department of Dental Public Health (Prof Z S Natto DrPH), King Fahd Medical Research Center (KFMRC), Entrepreneurship & Innovation Unit

(Prof Z S Natto DrPH), King Abdulaziz University, Jeddah, Saudi Arabia; Department of Respiratory Medicine (M A Althobiani PhD), Division of Medicine (T Oyelade PhD), University College London, London, England; Research Group in Health Economics (Prof N Alvis-Guzman PhD), Universidad De Cartagena (University of Cartagena), Cartagena, Colombia; Research Group in Hospital Management and Health Policies (Prof N Alvis-Guzman PhD), Universidad De La Costa (University of the Coast), Barranquilla, Colombia; Department of Health Sciences (N J Alvis-Zakzuk MSc), Universidad De La Costa, Barranquilla, Colombia; Department of Health Technology Assessment (N J Alvis-Zakzuk MSc), ALZAK, Cartagena, Colombia; Fakeeh College for Medical Sciences (Prof Y M Al-Worafi PhD), Fakeeh College for Medical Sciences, Jeddah, Saudi Arabia; College of Medical Sciences (Prof Y M Al-Worafi PhD), Azal University for Human Development, Sana'a, Yemen; College of Medicine (Prof A H Al-Zalabani PhD), Taibah University, Madinah, Saudi Arabia; Clinical Sciences - Orthopaedic Department (J Amin MSc), University of Science Malaysia, Kota Baharu, Malaysia; Kasr Alainy Faculty of Medicine (Prof T T Amin MD), Department of Neurophysiology (Prof H R Elhabashy MD), Cairo University, Cairo, Egypt; Bio-Medical Research Department (Prof T T Amin MD), Armed Forces College of Medicine, Cairo, Egypt; Department of Health and Management Sciences (S Amini PhD), Khomein University of Medical Sciences, Khomein, Iran; College of Nursing (A D Amirtharaj PhD), SULTAN QABOOS University, Muscat, Oman; Department of Medicine (G A Amusa MD), University of Jos, Jos, Nigeria; Department of Internal Medicine (G A Amusa MD), Jos University Teaching Hospital, Jos, Nigeria; Faculty of Medicine and Health (D B Anderson PhD), Sydney Musculoskeletal Health (D B Anderson PhD), The University of Sydney, Sydney, NSW, Australia; Department of Pharmacology (A Anil MD), College of Nursing (J Jacob MSc), All India Institute of Medical Sciences, Bhubaneswar, India; Division of Medical Research, SRM Medical College Hospital and Research Centre (A Anil Kumar PhD, S PJ PhD), Sri Ramaswamy Memorial Institute of Science and Technology, Chengalpattu, India; Department of Midwifery (A A Anshebo PhD), Wachemo University, Hosaena, Ethiopia; Department of Environmental and Occupational Health (B S Anuoluwa PhD), University of Medical Sciences, Ondo, Ondo, Nigeria; Regenerative Medicine, Organ Procurement and Transplantation Multi-disciplinary Center (S Anvari MD), Department of Social Medicine and Epidemiology (A Feizkhah MD), Department of Medical-Surgical Nursing (S Karkhah MSc), Guilan University of Medical Sciences, Rasht, Iran; Health Management and Economics Research Center (J Arabloo PhD, H Ayatollahi PhD), Department of Health Information Management (H Ayatollahi PhD), School of Medicine (M Bastan MD), Department of Medical Laboratory Sciences (F Dorostkar PhD), Endocrine Research Center, Institute of Endocrinology and Metabolism (R Ghafoury MD), Minimally Invasive Surgery Research Center (A Karimi Behnagh MD), Artificial Intelligence in Health Research Center (A Karimi Behnagh MD), Antimicrobial Resistance Research Center (K Mozahheb Yousefi MD), Hazrat-e Rasool General Hospital (K Mozahheb Yousefi MD), Physiology Research Center (H Pazoki Toroudi PhD), Department of Physiology (H Pazoki Toroudi PhD), Center for Technology and Innovation in Cardiovascular Informatics (S Shool MD), Iran University of Medical Sciences, Tehran, Iran; Department of Pharmaceutical Biotechnology (J M Aranjani PhD), Department of Physiotherapy (A Arumugam PhD, S Sharma PhD), Department of Forensic Medicine and Toxicology (S M Bakkannavar MD), Department of Pharmaceutical Regulatory Affairs and Management (S Dharmagadda PhD), Kasturba Medical College Mangalore (R Holla MD, M Rao MD, Prof B Unnikrishnan MD), Prasanna School of Public Health (R Kamath MHA), Kasturba Medical College, Manipal (P L C MD), Kasturba Medical College (Prof S Shastry MD), Manipal College of Dental Sciences, Mangalore (Prof P K Shetty MDS), Manipal Academy of Higher Education, Manipal, India; College of Art and Science (D Areda PhD), Ottawa University, Surprise, AZ, USA; Department of Cardiovascular, Endocrine-Metabolic Diseases and Aging (B

Armocida MD), Istituto Superiore Di Sanità (ISS), Rome, Italy; Institute of Social, Medical, and Applied Research Technology (A A Artamonov PhD), Institute of Social, Medical, and Applied Research Technology, Moscow, Russia; Faculty of Business and Law (F Arthur-Holmes PhD), University of Portsmouth, Portsmouth, UK; Cabrini Research (Prof M Asghari-Jafarabadi PhD), Cabrini Health, Malvern, VIC, Australia; Department of Biosciences (M Ashfaque PhD), Integral University, Lucknow, India; Department of Biostatistics (T Ashraf PhD), Sakarya University, Sakarya, Türkiye (A Hanif PhD); School of Traditional Chinese Medicine (M Aslam PhD), Xiamen University Malaysia, Sepang, Malaysia; Department of Nursing (I Asmar MSc), Birzeit University, Birzeit, Palestine; Institute of Population and Social Research (Ö Ataç PhD), Marmara University, Istanbul, Türkiye; Department of Public Health (Ö Ataç PhD), Istanbul Medipol University, Istanbul, Türkiye; Keck School of Medicine (M Athari MD), Department of Radiology (A Gholamrezanezhad MD), University of Southern California, Los Angeles, CA, USA; Department of Forensic Medicine (A Atreya MD), Department of Community Medicine (S Nepal MD), Lumbini Medical College, Palpa, Nepal; College of Medicine and Health Sciences (A H Atsbaha MPH), Adigrat University, Adigart, Ethiopia; Northumbria HealthCare NHS Foundation Trust, Newcastle upon Tyne, UK (A Aujayeb MBBS); Center for Health Systems Research (L Avila-Burgos ScD), Centro De Investigaciones En Salud Poblacional (J Campuzano Rincon PhD), National Institute of Public Health, Cuernavaca, Mexico; Institute of Molecular Biology and Biotechnology (IMBB) (S J Awan PhD), The University of Lahore, lahore, Pakistan; Trauma Research Center (S Ayatizadeh MD, M Karajizadeh PhD), Health Information Management (A Bashiri PhD), Research Center for Traditional Medicine and History of Medicine (Prof M Hashempur PhD), Poostchi Ophthalmology Research Center (M Heydari PhD), Health Policy Research Center (Y Sarikhani PhD), Shiraz University of Medical Sciences, Shiraz, Iran; Faculty of Nursing (A Ayed PhD), Arab American University Palestine, Jenin, Palestine; Department of Community Medicine and Primary Care (D J Ayowole MBChB), Federal Medical Centre, Abeokuta, Nigeria; Department of Mathematics & Statistics (A Azad PhD), Department of Biology (A A Chaudhary PhD), Department of Pharmacology (T Jawaid PhD), Imam Mohammad Ibn Saud Islamic University, Riyadh, Saudi Arabia; Social Determinants of Health Research Center (A Azadnia PhD), Kurdistan University of Medical Sciences (M Majidnia PhD), Kurdistan University of Medical Sciences, Sanandaj, Iran; Brown School of Public Health (A W Azale MD), Washington University in St. Louis, St.Louis, MO, USA; Department of Pharmaceutical Chemistry (Prof T Bączek PharmD), Medical University of Gdansk, Gdansk, Poland; Institute of Health Sciences (Prof T Bączek PharmD), Pomeranian University in Slupsk, Slupsk, Poland; School of Health Sciences (A C Badache PhD), Orebro University, Orebro, Sweden; School of Public Health (A C Badache PhD), Babes-Bolyai University, Cluj Napoca, Romania; Department of Forensic Science (A D Badiye PhD, N Kapoor PhD), Government Institute of Forensic Science Nagpur, Nagpur, India; Rashtrasant Tukadoji Maharaj Nagpur University, Nagpur, India (A D Badiye PhD); Department of Neurological Surgery (Y Bae MD), University of Ulsan, Seoul, South Korea; Clinical Medical Research Center (Prof R Bai PhD), Nanjing Children's Hospital, Nanjing, China; School of Public Affairs (Prof R Bai PhD), Nanjing University of Science and Technology, Nanjing, China; Surgery, Global Surgery, Emergency Medicine Department (Prof L Bains MD), Maulana Azad Medical College, New Delhi, India; WHO Collaborating Centre for Research in Surgical Care Delivery in LMICs, Mumbai, India (Prof L Bains MD); National Drug Dependence Treatment Centre (Prof Y Balhara MD), Centre for Community Medicine (P Halder MD), Department of Biophysics (T Mohammad PhD), Centre for Dental Education and Research (H Priya MDS), Department of Radiation Oncology (V Sahni MDS), All India Institute of Medical Sciences, New Delhi, India; Anahuac Business School (J Balmori-de-la-Miyar PhD), Universidad Anahuac Mexico, Mexico City, Mexico; Department of International Health (K Balsara DDS, H Zhang

PhD), Department of Surgery (M Chowdhury MD), Bloomberg School of Public Health (E G Fanta MD), Russell H. Morgan Department of Radiology and Radiological Science (A G Gad MD), School of Medicine (B M Jeswani MBBS), Department of Epidemiology (K Obiri - Yeboah MD), Johns Hopkins University, Baltimore, MD, USA; Center of Innovation, Technology, and Education (CITE) (Prof O Baltatu PhD), Anhembi Morumbi University, São José dos Campos, Brazil; School of Public Health (S A Bamboro MPH), Department of Public Health (T Mekene Meto MPH), Department of Nursing (B B Shashamo MSc), Department of Midwifery (G Temesgen MSc), Arba Minch University, Arba Minch, Ethiopia; Digestive Disease Institute (J S Barajas-Gamboa MD), Cleveland Clinic Abu Dhabi, Abu Dhabi, United Arab Emirates; Centmed (J S Barajas-Gamboa MD), New York University Abu Dhabi, Abu Dhabi, United Arab Emirates; School of Psychology (Prof S L Barker-Collo PhD), University of Auckland, Auckland, New Zealand; Department of Public and Environmental Health (A Barrow MPH), University of The Gambia, Banjul, The Gambia; Department of Epidemiology (A Barrow MPH, D Braithwaite PhD), University of Florida, Gainesville, FL, USA; School of Health Professions (Prof C A Barth PhD), Bern University of Applied Sciences, Bern, Switzerland; Faculty of Nursing (Prof A Batiha PhD), King Khalid University, Mahyil Asir, Saudi Arabia; Institute of Chemical Sciences (S Batool MS), Bahauddin Zakariya University, Multan, Pakistan; Department of Medical Education (K Batra PhD), School of Public Health (R Batra MS), Department of Social and Behavioral Health (Prof M Sharma PhD), University of Nevada Las Vegas, Las Vegas, NV, USA; IT Department (R Batra MS), Coforge, Georgia, GA, USA; Department of Psychiatry (Prof S Bazargan-Hejazi, PhD BEP), Charles R. Drew University of Medicine and Science, Los Angeles, CA, USA; Department of Psychiatry and Biobehavioral Sciences (Prof S Bazargan-Hejazi, PhD BEP), University of California Los Angeles, Los Angeles, CA, USA; School of Public Health (Prof N Bedi MD), Dr. D. Y. Patil University, Mumbai, India; Department of Human Anatomy and Histology (Prof N M Beeraka PhD), Department of Epidemiology and Evidence-Based Medicine (P D Lopukhov PhD, R V Polibin PhD), I.M. Sechenov First Moscow State Medical University, Moscow, Russia; Department of Adult Health Nursing/School of Nursing (D W Bekru MSc), Mekelle University, Mekelle, Ethiopia; Department of Dermatovenereology (S A Belay MD), University of Gondar, Gondar city, Ethiopia; Department of Emergency and Critical Care Nursing (A G Belayneh MSc), Department of Nursing (A Y Berhie MSc), Department of Environmental Health (T S Bunare MPH), College of Medicine and Health Science (K Y Gete MD), Department of Adult Health Nursing (E A Mengistie MSc), Department of Midwifery (A A Tesfu MSc), Bahir Dar University, Bahir Dar, Ethiopia; Department of General Surgery, Plastic Surgery (E Belayneh MD), St. Paul's Hospital Millennium Medical College, Addis Ababa, Ethiopia; Bayero University Kano (Prof B Bello PhD), Higher National School of Veterinary Medicine, Kano, Nigeria; North-West University, Mafikeng, South Africa (Prof B Bello PhD); Department of Industrial Engineering (Prof S Bendak PhD), Haliç University, Istanbul, Türkiye; BRAC James P Grant School of Public Health (S Bente Kamal Tune MPH), School of Pharmacy (M Islam PhD), BRAC University, Dhaka, Bangladesh; Institute of Health Sciences (A D Benti MSc), Department of Pharmacy (G Fekadu PhD), Wollega University, Nekemte, Ethiopia; Ashok & Rita Patel Institute of Physiotherapy (M S Bhadiyadra MPT, P Khuman PhD, N R Patel MPT), Charotar University of Science and Technology, Changa, India; Global Health Neurology Lab (S Bhaskar MD), NSW Brain Clot Bank, Sydney, NSW, Australia; Division of Cerebrovascular Medicine and Neurology (S Bhaskar MD), National Cerebral and Cardiovascular Center, Suita, Japan; Family Medicine Department (A Bhatnagar MD), Texas Tech University, El Paso, TX, USA; Department of Community and Family Medicine (S Bhattacharya MD), College of Nursing (V Patidar MSc), Forensic Medicine and Toxicology (Prof N A Sheikh MD), Department of Pharmacology (S T Y MD), All India Institute of Medical Sciences, Deoghar, India; Department of Human Genetics and Molecular Medicine

(Prof J S Bhatti PhD), Central University of Punjab, Bathinda, India; Department of Botanical and Environmental Sciences (Prof M S Bhatti PhD), Guru Nanak Dev University, Amritsar, India; Centre for Global Child Health (Prof Z A Bhutta PhD), Department of Medical Imaging (S Harsini MD), University of Toronto, Toronto, ON, Canada; Institute for Global Health & Development (Prof Z A Bhutta PhD), Department of Emergency Medicine (U R Khan PhD), Aga Khan University, Karachi, Pakistan; School of Public Health (F Bie MPH), University of Nevada Reno, nevada, NV, USA; Department of Emergency Medicine (C B Bills MPH), University of Colorado, Aurora, CO, USA; Department of Emergency Medicine (C B Bills MPH), Center for Tobacco Control Research and Education (P Kodali PhD), Department of Bioengineering and Therapeutical Sciences (Prof M Zastrozhin PhD), University of California San Francisco, San Francisco, CA, USA; Department of Pathobiology (A Binhambali DVM), Tuskegee University, Tuskegee, AL, USA; Department of Health Services Management (M Mohseni PhD), Isfahan University of Medical Sciences, Isfahan, Iran (P Bolourinejad MD); Department of Epidemiology and Psychosocial Research (Prof G Borges DSc, R A Gutiérrez PhD), Ramón De La Fuente Muñiz National Institute of Psychiatry, Mexico City, Mexico; Transport and Logistics Knowledge Bank (D Bose PhD), World Bank, Washington, DC, USA; Disease Surveillance Department (S A Bosoka MPhil), Ghana Health Service, Ho, Ghana; Facultad De Salud (Faculty of Health) (Prof A Botero Carvajal PhD), Universidad Santiago De Cali, Cali, Colombia; Department of Medicine (Prof S Bouaoud DrPH), Faculty of Medicine (Prof A Ouyahia PhD), University Ferhat Abbas of Setif, Sétif, Algeria; Department of Epidemiology and Preventive Medicine (Prof S Bouaoud DrPH), University Hospital Saadna Abdenour, Sétif, Algeria; Cancer Population Sciences Program (D Braithwaite PhD), University of Florida Health Cancer Center, Gainesville, FL, USA; School of Medicine and Health (F Busch MD), Technical University of Munich, Munich, Germany; Department of Radiology (F Busch MD), University of Cambridge, Cambridge, England; The Apollo University (S C MOT), Apollo Hospital, Chittor, India; Department of Internal and Geriatric Medicine (Prof L A Cámera MD), Hospital Italiano De Buenos Aires (Italian Hospital of Buenos Aires), Buenos Aires, Argentina; Board of Directors (Prof L A Cámera MD), Argentine Society of Medicine, Buenos Aires, Argentina; School of Medicine (J Campuzano Rincon PhD), University of the Valley of Cuernavaca, Cuernavaca, Mexico; Heidelberg Institute of Global Health (Z Cao PhD), Heidelberg University Hospital and Faculty of Medicine (Prof S Mohammed PhD), Heidelberg University, Heidelberg, Germany; Interdisciplinary Research Center for Health Science (A Capodici MD), Sant'Anna School of Advanced Studies, Pisa, Italy; Research Unit on Applied Molecular Biosciences (UCIBIO) (Prof F Carvalho PhD), University of Porto, Porto, Portugal; Education Center of Australia (A Carvalho-e-Silva PhD), Health Science College, Sydney, NSW, Australia; Department of Psychiatry (Prof J Castaldelli-Maia PhD), University of Sao Paulo, São Paulo, Brazil; Department of Medical, Surgical, and Health Sciences (Prof L Cegolon PhD, Prof M D'Oria MD), University of Trieste, Trieste, Italy; Public Health Unit (Prof L Cegolon PhD), University Health Agency Giuliano-Isontina (ASUGI), Trieste, Italy; Mary MacKillop Institute for Health Research (Prof E Cerin PhD), Australian Catholic University, Melbourne, VIC, Australia; School of Public Health (Prof E Cerin PhD), University of Hong Kong, Hong Kong, China; Institute of Clinical Physiology (S Cerrai MSc), Italian National Council of Research, Pisa, Italy; State Disease Investigation Laboratory (S Chakraborty MVSc), Animal Resources Development Department, Agartala, India; Department of Public Health and Health Sciences (Prof V Chattu PhD), Tennessee State University (TSU), Nashville, TN, USA; Department of Community Medicine (Prof V Chattu PhD), Datta Meghe Institute of Medical Sciences, Sawangi, India; ICMR National Institute of Health Research, Dibrugarh (S Chaudhuri MD), Indian Council of Medical Research, Dibrugarh, India; Indian Institute of Public Health-Delhi (A S Chauhan PhD), Public Health Foundation of India, Gurugram, India; Department of Oral Medicine and

Radiology (Prof A Chaurasia MD), King George's Medical University, Lucknow, India; Faculty of Humanities and Health Sciences (H Chen MSc), Curtin University, Miri, Malaysia; Division of Infectious Diseases (P R Ching MD), Virginia Commonwealth University, Richmond, VA, USA; Centre for Research Impact & Outcome (H Chopra PhD), Centre for Research Impact and Outcome, Chitkara College of Pharmacy (Prof A Sahebkar PhD), Chitkara University, Rajpura, India; Department of Humanities and Social Sciences (D Choudhury PhD), Sri Sathya Sai Institute of Higher Learning, Sri Sathya Sai District, India; Department of Pharmaceutical Technology (J A Chowdhury PhD), University of Dhaka, Dhaka, Bangladesh; Department of Health Science and Technology (S W M Christensen PhD), Aalborg University, Aalborg, Denmark; Department of Physiotherapy (S W M Christensen PhD), University College of Northern Denmark, Aalborg, Denmark; Department of Paediatric Surgery (I S Chukwu BMedSc), Federal Medical Centre, Umuahia, Nigeria; Department of Urology (Prof E Chung MD), The University of Queensland, Brisbane, QLD, Australia (M Moni PhD); Department of AndroUrology (Prof E Chung MD), AndroUrology Centre, Brisbane, QLD, Australia; Department of Health Behavior (S Chung MPH), Texas A&M University, College Station, TX, USA; Department of Health Sciences (C Cosma MD), University of Florence, Florence, Italy; Department of Medical and Surgical Sciences (Prof S Curti PhD, M Sassano MD, Prof F S Violante MD), Department of Medicine and Surgery (I Papadimopoulos MD), University of Bologna, Bologna, Italy; Center for Immigrant and Refugee Health (O Dadras PhD), Public Health Institute, Los Angeles, CA, USA; School of Postgraduate Studies and Research (H M Dahir MPH), Amoud University, Hargiesa, Somalia; Department of Medical and Surgical Sciences (Prof E D'Amico MD, A Zanghì MD), University of Foggia, Foggia, Italy; School of Public Health (L Damoah MD, Z Kabir PhD), University College Cork, Cork, Ireland; Institute for Global Health Innovations (A K Dang MD), Duy Tan University, Hanoi, Viet Nam; Department of Environmental Health (R Darvishi Cheshmeh Soltani PhD), Department of Nursing (A Jadidi PhD), Department of Pediatrics (J Nazari MD), Arak University of Medical Sciences, Arak, Iran; Road Traffic Injury Research Center (E Davtalab Esmaeili PhD), School of Management and Medical Informatics (L R Kalankesh PhD), Tabriz University of Medical Sciences, Tabriz, Iran (F Sadeghi-Ghyassi PhD); Department of Neurosurgery (B G de Liyis MD), National Brain Center in Indonesia, Jakarta, Indonesia; School of Medicine (I Delgado-Enciso DSc), University of Colima, Colima, Mexico; Department of Research (I Delgado-Enciso DSc), State Cancerology Institute of Colima, IMSS-BIENESTAR, Colima, Mexico; Department of Neurosurgery (A K Demetriades MD), Deanery of Biomedical Sciences (R Elsheikh MD), College of Medicine and Veterinary Medicine (G Verras MSc), University of Edinburgh, Edinburgh, Scotland; Department of Neurosurgery (A K Demetriades MD), National Health Service (NHS) Scotland, Edinburgh, Scotland; Department of Forensic Medicine (Prof E Dervišević PhD), University of Sarajevo, Sarajevo, Bosnia and Herzegovina; Department of Public Health (A T Desale MPH), Debre Tabor University, Debre Tabor, Ethiopia; Department of Anesthesia (M Desalegn MSc), Department of Public Health (E A Turuse MPH), Department of Nursing (S Untiso MSc), Wachemo University, Hossana, Ethiopia; Chettinad Hospital & Research Institute (Prof V Devanbu MD), Chettinad Academy of Research and Education, Chennai, India; JSS Medical College (D Devegowda PhD), Jagadguru Sri Shivarathreeswara Academy of Health Education and Research, Mysuru, India; Sheffield Teaching Hospitals NHS Foundation Trust, Sheffield, UK (A Dhali MBBS); Department of Community Medicine (Prof S D Dharmaratne MD), Department of Oral Medicine and Periodontology (Prof R D Jayasinghe MS), University of Peradeniya, Peradeniya, Sri Lanka; Population Interventions Unit (B Dhungel DrPH), University of Melbourne, Melbourne, VIC, Australia; Faculty of Management (Prof A Dima PhD), Department of Statistics and Econometrics (A Otoiu PhD), Bucharest University of Economic Studies, Bucharest, Romania; Care and Health Program (M Dinga-Nyoh MD), Care and Health Program,

Yaounde, Cameroon; National Institute for Research in Environmental Health (V Diwan PhD), Indian Council of Medical Research, Bhopal, India; Department of Global Public Health (V Diwan PhD, Prof L Laflamme PhD, A N Shaaban PhD), Department of Molecular Medicine and Surgery (Prof J H Kauppila MD), Karolinska Institute, Stockholm, Sweden; Institute of Medical Biostatistics, Epidemiology, and Informatics (J Dönicke MSc), Johannes Gutenberg University Mainz, Mainz, Germany; Cardio-Thoraco-Vascular Department (Prof M D'Oria MD), Azienda Sanitaria Universitaria Giuliano Isontina, Trieste, Italy; Independent Consultant, Bridgewater, NJ, USA (O P Doshi MS); Department of Epidemiology (M Dresse MD), University of Pittsburgh, Pittsburgh, PA, USA; Department of Psychiatry (M Dresse MD), University of Pittsburgh Medical Center, Pittsburgh, PA, USA; Faculty of Science and Humanities (S Duraisamy PhD), SRM Institute of Science and Technology, Kattankulathur, India; Department of Dermatology, Venereology, and Leprology (V Eddula MD), Apollo Institute of Medical Sciences and Research Chittoor, Chittoor, India; School of Public Health (Y Efa MPH), University of Adelaide, Adelaide, SA, Australia; Biotechnology Department (M Eissa MSc), Suez Canal University, Ismailia, Egypt; Faculty of Science and Health (M Ekholuenetale PhD), University of Portsmouth, Hampshire, UK; Department of Public Health and Community Medicine (Prof I F El Bayoumy DrPH), Tanta University, Tanta City, Egypt; School of Public Health (Prof I F El Bayoumy DrPH), Texila American University, Guyana, Guyana; Biomedical Informatics and Medical Statistics Department (Prof I El Sayed PhD), Pediatric Dentistry and Dental Public Health Department (Prof O A A Elmeligy PhD), Department of Pathology (Prof I M Talaat PhD), Alexandria University, Alexandria, Egypt; Department of Anatomy and Embryology (M A Eladl PhD), Nursing Faculty (F M Ibrahim PhD), Mansoura Faculty of Medicine (H Magdy Abd El Razek MD), Department of Cardiology (Prof M M Ramadan PhD), Mansoura University, Mansoura, Egypt; College of Medicine (M Elhadi MD), Department of Biomedical Informatics (Prof S Jeong PhD), School of Health and Environmental Science (Prof J Kang PhD), Department of Preventive Medicine (Prof Y Lee PhD), Korea University, Seoul, South Korea; Houston Methodist Hospital, Houston, TX, USA (M Elhadi MD); College of Public Health and Health Informatics (Prof A A El-Metwally PhD), King Saud Bin Abdulaziz University for Health Sciences, Riyadh, Saudi Arabia; King Abdullah International Medical Research Center, Riyadh, Saudi Arabia (Prof A A El-Metwally PhD); Research Centre for Healthcare and Community (A F Fagbamigbe PhD), Coventry University, Coventry, UK; Tanner College of Dental Medicine (Prof A Fahim PhD), University of Pikeville, Pikeville, KY, USA; Department of Surgery, and Clinical and Public Health Research (E G Fanta MD), Department of Surgery (N I Kassa MD), Department of Medicine (N Suga MD), MyungSung Medical College, Addis Ababa, Ethiopia; Department of Health Policy and Administration (E A Faraon MD, Prof F B Garcia PhD), Department of Neurosciences (Prof R G Jamora PhD), University of the Philippines Manila, Manila, Philippines; College of Medicine (M Fareed PhD), AlMaarefa University, Riyadh, Saudi Arabia; Environmental Statistics Unit (C S E S Farinha PhD), National Institute of Statistics, Lisbon, Portugal; Ecological Economics and Environmental Management (C S E S Farinha PhD), NOVA University of Lisbon, Lisbon, Portugal; Department of Psychology (A Faro PhD), Federal University of Sergipe, São Cristóvão, Brazil; Department of Radiography and Imaging Technology (S Farooq PhD), Green International University, Lahore, Pakistan; Centre for Public Health, Equity and Human Flourishing (N K Fauk PhD), Torrens University Australia, Adelaide, SA, Australia; Institute of Resource Governance and Social Change, Kupang, Indonesia (N K Fauk PhD); School of Medicine (Prof M Fayaz PhD), Shahed University, Tehran, Iran; Department of Infectious Diseases and Public Health (G Fekadu PhD), City University of Hong Kong, Hong Kong, China; Department of Neurosurgery (A H Feroze MD), Children's National Medical Center, Washington, DC, USA; Department of Neurosurgery (A H Feroze MD), School of Engineering and Applied Science (A Gerami Matin PhD), Milken Institute School of Public Health (L S

Mogus MD), George Washington University, Washington, DC, USA; Department of Social Sciences (Prof N Ferreira PhD, Prof M J M Sullman PhD), Department of Life and Health Sciences (Prof M J M Sullman PhD), University of Nicosia, Nicosia, Cyprus; Institute of Public Health (A B Fissha MD), Washington University in St. Louis, St Louis, MO, USA; School of Medicine (A B Fissha MD), Department of Orthopedics and Trauma Surgery (A Haile MD), Department of Anatomy (Y T Zikarg MSc), Addis Ababa University, Addis Ababa, Ethiopia; Department of Pharmacology (Prof B Foroutan PhD), Iranshahr University of Medical Sciences, Iranshahr, Iran; Department of Neuroscience (M Foschi MD), Multiple Sclerosis Research Center, Ravenna, Italy; Department of Biotechnological and Applied Clinical Sciences (M Foschi MD), University of L'Aquila, L'Aquila, Italy; Division of Clinical Epidemiology (KEP) (K R Fowobaje PhD), Karolinska Institute, Stockholms, Sweden; College of Medicine, Dentistry and Public Health (Prof R C Franklin PhD), College of Public Health, Medical, and Veterinary Sciences (A E Peden PhD), James Cook University, Townsville, QLD, Australia; Department of Community Medicine (Prof M A Gadanya MD), Aminu Kano Teaching Hospital, Kano, Nigeria; Department of Public Health (M Gajdács PhD), Department of Medical Physics and Informatics (T Lantos PhD), University of Szeged, Szeged, Hungary; Laboratory Sciences Division (B Ganesh PhD), National Institute of Epidemiology, Chennai, India; Trauma and Emergency Medicine Department (E Ganessane MD), All India Institute of Medical Sciences (AIIMS), Madurai, Madurai, India; Infectious Diseases Unit (J Garlasco MD), University of Verona, Verona, Italy; Department of Ophthalmology (Prof Z Gatzioufas PhD), University of Basel, Basel, Switzerland; Department of Applied Health Sciences (L Ghalichi PhD), College of Medicine and Health (J Muhammad PhD), University of Birmingham, Birmingham, England; Research Committee of Qom University of Medical Sciences (A Ghamkhar BSc), Qom University of Medical Sciences, Qom, Iran; Department of Medical Biochemical Analysis (A Ghandili PhD), Department of Business Administrations (Prof D H Kadir PhD), Cihan University-Erbil, Erbil, Iraq; School of Public Health (A Ghashghaee BSc), Qazvin University of Medical Sciences, Qazvin, Iran; International University of Health and Welfare (T G Gia Nguyen MD), International University of Health and Welfare, Narita, Japan; Department of Nursing (A A Girmay MSc), Aksum University, Aksum, Ethiopia; Department of Anesthesiology and Critical Care Medicine (A Girombelli MD), Ospedale SS Annunziata Savigliano, Savigliano, Italy; Department of Clinical and Experimental Sciences (Prof M Gobbo MD), University of Brescia, Brescia, Italy; IRCSS Fondazione Don Gnocchi (Prof M Gobbo MD), IRCCS Fondazione Don Gnocchi, Rovato (Brescia), Italy; Department of Biostatistics (K Gohari MS), Tarbiat Modares University, Tehran, Iran; Quantitative Department (K Gohari MS, A Sheidaei PhD), Non-Communicable Diseases Research Center (NCDRC), Tehran, Iran; Department of Health Systems and Policy Research (Prof M Golechha PhD), Indian Institute of Public Health, Gandhinagar, India; Department of Genetics (P Goleij MSc), Sana Institute of Higher Education, Sari, Iran; Universal Scientific Education and Research Network (USERN) (P Goleij MSc), Social Development and Health Promotion Research Center (M Khezeli PhD), Kermanshah University of Medical Sciences, Kermanshah, Iran; Department of Family Medicine (W Gong PhD), Shenzhen University Medical School, Shenzhen, China; Shenzhen Clinical Research Center for Trauma Treatment (W Gong PhD), Shenzhen University, Shenzhen, China; School of Medical Sciences (S Gooty MPH), University of Hyderabad, Hyderabad, India; Department of Public Health and Preventive Medicine (Prof M Grivna PhD), Charles University, Prague, Czech Republic; Department of Epidemiology and Biostatistics (S Guan MD), Anhui Medical University, Hefei, China; Medical Directorate (G Guarducci MD), Local Health Authority of Ferrara, Ferrara, Italy; Department of Clinical Science (M I M Gubari PhD), University Of Sulaimani, Sulaimani, Iraq; Department of Community Medicine (Prof D A Gunawardane MD, Prof S N K Navaratna MD), University of Peradeniya, Kandy, Sri Lanka; Department of Toxicology (S Gupta PhD), Shriram

Institute for Industrial Research, Delhi, India; Department of Anaesthesia (P Gurnal MD), All India Institute of Medical Sciences, Bilaspur, India (L Rana MD); Doctoral Program in Biomedical Gerontology (R S Gutiérrez-Murillo PhD), Pontifical Catholic University of Rio Grande Do Sul, Porto Alegre, Brazil; Neurosurgery Department (A Habibzadeh MD), Fasa University of Medical Sciences, Shiraz, Iran; Bloomberg School of Public Health (A Habteyesus MPH), Johns Hopkins University, Arlington, VA, USA; Department of Hepato-Pancreatico-Biliary Surgery (N Hai Nam PhD), Thong Nhat Hospital, Ho Chi Minh City, Viet Nam; Department of Community Medicine (P Halder MD, A KM MD, P Kumar PhD), Department of Pharmacology (A K Kakkar MD), Post Graduate Institute of Medical Education and Research, Chandigarh, India; Department of Family and Community Medicine (Prof R R Hamadeh PhD), Arabian Gulf University, Manama, Bahrain; Department of Statistics and Informatics (F O Hamarasool MSc), Department of Statistics (Prof D H Kadir PhD), Salahaddin University-Erbil, Erbil, Iraq; Department of Neurosurgery (A M Hamouda MD), Mayo Clinic, Rochester, USA; Department of Epidemiology, Biostatistics, Population Studies and Health Promotion (A Hargono Dr), Faculty of Public Health, Universitas Airlangga, Surabaya, Indonesia; Department of Ophthalmology (H Hasani MD), School of Medicine (M Shams-Beyranvand MSc), Alborz University of Medical Sciences, Karaj, Iran; Department of Periodontics (N T Hashim PhD), Department of Community Medicine (M Patni MD), RAK Medical and Health Sciences University, Ras Al Khaimah, United Arab Emirates; Department of Community Medicine (I Hassan PhD), Federal University Teaching Hospital, Lafia, Nigeria; Department of Epidemiology and Community Medicine (I Hassan PhD), Federal University of Lafia, Lafia, Nigeria; Faculty of Kinesiology (Prof J J Hebert PhD), University of New Brunswick, Fredericton, NB, Canada; School of Allied Health (Prof J J Hebert PhD), Murdoch University, Murdoch, WA, Australia; Faculty of Health (M Hobbs PhD), University of Canterbury, Christchurch, New Zealand; Department of Statistics (M Hossain MSc, M Islam BSc, M Mostafa BSc), Shahjalal University of Science and Technology, Sylhet, Bangladesh; Department of Public Health (S Hossain MPH), University of Science and Technology Chittagong (USTC), Chattogram, Bangladesh; Department of Public Health (S Hossain MPH), Daffodil International University (DIU), Dhaka, Bangladesh; School of Engineering and Technology (Prof M Hosseinzadeh PhD), Institute of Research and Development (S Khoshvaght MSc, A Omar Bali PhD), School of Engineering & Technology (S Khoshvaght MSc), Institute for Global Health Innovations (C T Nguyen MPH, H T Nguyen MD), Faculty of Medicine (T Nguyen PhD), Duy Tan University, Da Nang, Viet Nam; International Center for Materials Sciences and Technology (Prof M Hosseinzadeh PhD), Western Caspian University, Baku, Azerbaijan; Department of Legal Medicine and Bioethics (Prof S Hostiuc PhD), Department of General Surgery (I Negoi PhD, B Socea PhD), Department of Dermatology (M Tampa PhD), Carol Davila University of Medicine and Pharmacy, Bucharest, Romania; Department of Clinical Legal Medicine (Prof S Hostiuc PhD), National Institute of Legal Medicine Mina Minovici, Bucharest, Romania; Department of Public Health and Community Medicine (Prof A Humayun PhD), Shaikh Zayed Postgraduate Medical Institute, Lahore, Pakistan; Department of Humanities (W Husain PhD), COMSATS University Islamabad, Islamabad, Pakistan; Czech National Centre for Evidence-Based Healthcare and Knowledge Translation (S Hussain PhD), Institute of Biostatistics and Analyses (S Hussain PhD), Masaryk University, Brno, Czech Republic; Department of Physiotherapy (A A Ibrahim PhD, S K Sulaiman PhD), Tishk International University, Erbil, Iraq; RAK Medical and Health Science University, RAK College of Nursing (F M Ibrahim PhD), Arak University of Medical Sciences, RAK, United Arab Emirates; Scientific and Technological Park (A Ibrayeva PhD), Department of Science (A Shamsutdinova MD, A Y Tazhiyeva PhD), Department of Prosthetic Dentistry (M Tleshev MSc), Atchabarov Scientific Research Institute of Fundamental and Applied Medicine (A Zhumagaliuly MD), Kazakh National Medical University, Almaty, Kazakhstan; West

Africa RCC (O S Ilesanmi PhD), Africa Centre for Disease Control and Prevention, Abuja, Nigeria; Department of Community Medicine (O S Ilesanmi PhD), Department of Medicine (Prof M O Owolabi DrM), Department of Oral and Maxillofacial Surgery (A A Salami BDS), University College Hospital, Ibadan, Ibadan, Nigeria; Faculty of Medicine (I M Ilic PhD, Prof M M Santric-Milicevic PhD), School of Public Health and Health Management (Prof M M Santric-Milicevic PhD), University of Belgrade, Belgrade, Serbia; Faculty of Medical Sciences (Prof M D Ilic PhD), University of Kragujevac, Kragujevac, Serbia; Research Center for Public Health and Nutrition (B Rachmat MPH), National Research and Innovation Agency, Jakarta, Indonesia (E Indriasih PhD, I U Tarigan PhD); College of Nursing (D Iqbal PhD), Onaizah Colleges, Qassim, Saudi Arabia; University of Michigan, Ann Arbor, MI, USA (B Irfan MS); Faculty of Pharmacy (L M Irham PhD), Faculty of Public Health (Prof S Solikhah DrPH), Universitas Ahmad Dahlan, Yogyakarta, Indonesia; Department of Public Health (M Islam PhD), Miyan Research Institute (M Rony MPH), International University of Business Agriculture and Technology, Dhaka, Bangladesh; 4-Green Research Society, Bangladesh, Dhaka, Bangladesh (M Islam PhD); Department of Zoology (Prof O A Iyiola PhD), Department of Veterinary Public Health and Preventive Medicine (I A Odetokun PhD), University of Ilorin, Ilorin, Nigeria; One Health Research Group (Prof J S Izquierdo-Condoy MSc, Prof E Ortiz-Prado PhD), Universidad De Las Américas, Quito, Ecuador; Department of Physical Medicine and Rehabilitation (L Jacob MD), Université Paris Cité, Paris, France; Research and Development Unit (L Jacob MD), Biomedical Research Networking Center for Mental Health Network (CiberSAM), Barcelona, Spain; Department of Health Studies (K H Jacobsen PhD), University of Richmond, Richmond, VA, USA; Section of Economic & Social Sciences, Humanities & Arts (Prof M Jakovljevic PhD), The World Academy of Sciences UNESCO-TWAS, Trieste, Italy; Shaanxi University of Technology, Hanzhong, China (Prof M Jakovljevic PhD); Department of Environmental Engineering (Prof R Jalilzadeh Yengejeh PhD), Islamic Azad University, Ahvaz, Iran; SRM Medical College Hospital and Research Centre (J James MD), Division of Medical Research (R Janardhanan PhD), Sri Ramaswamy Memorial Institute of Science and Technology, Kattankulathur, India; Institute for Neurosciences (Prof R G Jamora PhD), St. Luke's Medical Center, Bonifacio Global City, Philippines; SRM College of Physiotherapy (Prof B Janakiraman PhD), SRM Institute of Science and Technology (SRMIST), Kattankulathur, Tamil Nadu, India; Department of Neurosurgery (S Javed MS), Research Institute at Nationwide Children's Hospital, Rawalpindi, Pakistan; The Medical City for Military and Security Services School, Muscat, Oman (S Jayapal PhD); Department of Oral Medicine and Periodontology (Prof R D Jayasinghe MS), Saveetha Medical College and Hospital (Prof M Karobari PhD), Saveetha Dental College and Hospitals (G Minervini PhD, M Tovani-Palone PhD), Department of Paediatric and Neonatal Sciences (J Thirumalai MPT), Saveetha University, Chennai, India; Postgraduate Institute of Medicine (A U Jayatilleke PhD, Prof S N K Navaratna MD, J Samaranayake MBBS), Department of Anatomy, Genetics and Biomedical Informatics (Y Mathangasinghe PhD), Department of Paediatrics (Prof S Rajindrajith PhD), Department of Surgery (D P Wickramasinghe MD), University of Colombo, Colombo, Sri Lanka; Faculty of Graduate Studies (A U Jayatilleke PhD), Institute for Violence and Injury Prevention, Colombo, Sri Lanka; Department of Community Medicine (R P Jha MSc), Dr. Baba Saheb Ambedkar Medical College & Hospital, Delhi, India; Department of Community Medicine (R P Jha MSc), Banaras Hindu University, Varanasi, India; Department of Orthopedic Surgery (J Johnson MD), University of Alabama at Birmingham, Birmingham, AL, USA; Rothschild Foundation Hospital (Prof J B Jonas MD), Rothschild Foundation Hospital, Paris, France; Singapore Eye Research Institute (Prof J B Jonas MD), Singapore Eye Research Institute, Singapore, Singapore; Health Data Science and Artificial Intelligence Centre, Health Services Management Training Centre, Faculty of Health and Public Administration (T Joo PhD), Semmelweis University, Budapest, Hungary; Hungarian Health

Management Association, Budapest, Hungary (T Joo PhD); School of Engineering and Technology (A P Joseph PhD), The University of New South Wales, Canberra, NSW, Australia; Department of Community Medicine (N Joseph MD, N Kumar MD, P Mithra MD, R Motappa MD, R Thapar MD), Kasturba Medical College (G A Menezes PhD), Department of Forensic Medicine and Toxicology (Prof J Padubidri MD, Prof P Rastogi MD), Manipal Academy of Higher Education, Mangalore, India; Faculty of Applied & Basic Sciences (B Joshi PhD), SGT University of Medical Sciences, Gurugram, India; Institute of National Importance (K Joshi MD), All India Institute of Medical Sciences, Rajkot, India; Department of Economics (C E Joshua BSc), National Open University, Benin City, Nigeria; School of Computer Science and Engineering (T K PhD), RV University, Bengaluru, India; Research Department (Z Kabir PhD), TobaccoFree Research Institute Ireland, Dublin, Ireland; Physiotherapy Department (Prof B Kaka PhD), Baylor University, Kano, Nigeria; Department of Physiotherapy (Prof B Kaka PhD, O O Ojedoyin PhD), University of KwaZulu-Natal, Durban, South Africa; Department of Health (K Kalavani PhD), Khoy Medical Sciences, Khoy, Iran; Care and Public Health Research Institute (CAPHRI) (R Kamath MHA), Maastricht University, Maastricht, Netherlands; Elderwood Rehabilitation Clinical Centre, Buffalo, NY, USA (R T Kamorudeen MPH); Department of Public Health (R T Kamorudeen MPH), South Wales University, Treforest, UK; Department of Anesthesia, Critical Care and Pain Medicine (Prof J Kang PhD), Department of Orthopaedics (O Subasi PhD), Massachusetts General Hospital, Boston, MA, USA; Office of the Executive Director (Prof K K Kanmodi MPH), Cephas Health Research Initiative Inc., Ibadan, Nigeria; Department of Public Health (Prof K K Kanmodi MPH), Thomas Adewumi University, Oko, Nigeria; The Hansjörg Wyss Department of Plastic and Reconstructive Surgery (R S Kantar MD), Rory Meyers College of Nursing (X Qi PhD), New York University, New York, NY, USA; Department of Analytical & Applied Economics (S K Kar MPhil), Department of Analytical and Applied Economics (Prof H Rout PhD, C Swain MPhil), RUSA Centre of Excellence in Public Policy and Governance (Prof H Rout PhD), UGC Centre of Advanced Study in Psychology (Prof M Satpathy PhD), Utkal University, Bhubaneswar, India; School of Health Professions and Human Services (I M Karaye MD), Department of Population Health (Prof C Rojanaworarit PhD), Hofstra University, Hempstead, NY, USA; Department of Anesthesiology (I M Karaye MD), Montefiore Medical Center, Bronx, NY, USA; Surgery Research Unit (Prof J H Kauppila MD), University of Oulu, Oulu, Finland; Medical Basic Sciences Research Institute (N Kaydi PhD), Student Research Committee (N Kaydi PhD), Department of Public Health (M A Khafaie PhD), Environmental Technologies Research Center, Medical Basic Sciences Research Institute (M A Khafaie PhD), Department of Microbiology (M Saki PhD), Ahvaz Jundishapur University of Medical Sciences, Ahvaz, Iran; Open, Distance and eLearning Campus (Prof P N Keiyoro PhD), University of Nairobi, Nairobi, Kenya; Natural and Medical Sciences Research Center (A Khan PhD), Department of Biological Sciences and Chemistry (DBSC) (Z Naureen PhD), School of Pharmacy (A K Philip PhD), University of Nizwa, Nizwa, Oman; Dr Sulaiman Al Habib Hospital (H A Khan MCHorthopedics), Sheri Kashmir Institute of Medical Sciences, Riyadh, Saudi Arabia; University Institute of Public Health (I H Khan PhD, S Nargus PhD), University Institute of Radiological Sciences and Medical Imaging Technology (M Latif PhD), Institute of Molecular Biology and Biotechnology (IMBB) (S Shahid PhD), Research Centre for Health Sciences (RCHS) (S Shahid PhD), The University of Lahore, Lahore, Pakistan; Department of Community Medicine (M Khan MPH), National Institute of Preventive and Social Medicine, Dhaka, Bangladesh; Department of Pharmacy (M Khan MPhil), Minhaj University Lahore, Lahore, Pakistan; International Center for Chemical and Biological Sciences (S Khan PhD), International Center for Chemical and Biological Sciences, Karachi, Pakistan; Centre for Interdisciplinary Research in Basic Sciences (S Khan MSc), Centre For Interdisciplinary Research In Basic Sciences (CIRBSc) (T Mohammad PhD), Jamia Millia Islamia, New Delhi, India; Department of Cardiology (Z Khan MD),

University of South Wales, Treforest, UK; Department of Cardiology (Z Khan MD), University of Buckingham, Buckingham, UK; Central Department of Zoology (S Khanal MSc), Faculty of Humanities and Social Sciences (U Paudel PhD), Tribhuvan University, Kathmandu, Nepal; Department of Pharmacology (S U Khasbage MD), All India Institute of Medical Sciences, Raipur, India; Faculty of Nursing (H Khatatbeh PhD), Department of Basic Medical Sciences (Prof M M Khatatbeh PhD), Yarmouk University, Irbid, Jordan; Department of Orthopaedics (K Khatri MS), Postgraduate Medical Institute, Sangrur, India; Asadabad School of Medical Sciences (A Khazaei PhD), Asadabad School of Medical Sciences, Asadabad, Iran; Department of Neurosurgery (D Kheradmand MD), Biotechnology Research Center (Prof A Sahebkar PhD), Department of Medical Informatics (S Tabatabaei PhD), Applied Biomedical Research Center (S Tabatabaei PhD), Mashhad University of Medical Sciences, Mashhad, Iran; Department of Biochemistry and Nutrition (M Khorasani PhD), Neyshabur University of Medical Sciences, Neyshabur, Iran; Department of Public Health (J Khubchandani PhD), New Mexico State University, Las Cruces, NM, USA; School of Health Sciences (Prof A Kisa PhD), Kristiania University College, Oslo, Norway; Department of International Health and Sustainable Development (Prof A Kisa PhD), Tulane School of Public Health and Tropical Medicine (E Lytvyak MD), Department of Environmental Health Sciences (S P Sherchan PhD), Tulane University, New Orleans, LA, USA; Department of Medical Sciences (M A Kisiel PhD), Uppsala University Hospital, Uppsala, Sweden; Department of Public Health Dentistry (Prof S KM PhD), Krishna Vishwa Vidyapeeth (Deemed to Be University), Karad, India; Department of Public Health and Community Medicine (P Kodali PhD), Central University of Kerala, Kasaragod, India; College of Medicine and Health Science (W E Korma MSc), Werabe University, Werabe, Ethiopia; Department of Science and Environmental Studies (T Kormoker MSc), The Education University of Hong Kong, Hong Kong, China; School of Post Graduate (A H Korse MPH), Amoud University, Hargeisa, Somalia; Feinberg School of Medicine (A S Kozhumam MSc), Medical Scientist Training Program (S Marzouk MA), Northwestern University, Chicago, IL, USA; Department of Anthropology (Prof K Krishan PhD), Department of Forensic Science (Prof V Sharma PhD), Panjab University, Chandigarh, India; Department of Forensic Medicine and Toxicology (V Krishna MD), Pondicherry University, Puducherry, India; Department of Medical Education and Informatics (B Kucuk Bicer PhD), Gazi University Faculty of Medicine, Ankara, Türkiye; Department of Research Management (M Kulimbet MSc, B Lakanova MD), Research Institute of Cardiology and Internal Diseases, Almaty, Kazakhstan; Department of Health Administration and Education (E Kumah PhD), University of Education, Winneba, Winneba, Ghana; Amity Institute of Health Allied Sciences (Prof C Kumar PhD), Amity University Noida, Uttar Pradesh, Noida, India; Department of Community Medicine (D Kumar MD), Department of Anaesthesiology (T Kumar DNB), Rajendra Institute of Medical Sciences, Ranchi, India; Department of Mathematics (Prof K Kumar PhD), Amity University Haryana, Gurugram, India; Wits Advanced Drug Delivery Platform Research Unit (Prof P Kumar PhD), Wits Infectious Diseases and Oncology Research Institute (Prof P Kumar PhD), University of the Witwatersrand, Johannesburg, South Africa; Monitoring & Evaluation (P Kumar PhD), IGP Health Initiatives Pvt. Ltd., Delhi, India; Department of Paediatrics (R Kumar MD), School of Public Health (B Thomas M PhD), Sri Ramaswamy Memorial Institute of Science and Technology, Chennai, India; Faculty of Education (S Kumar PhD), Department of Forensic Science (Prof B K Sharma PhD), Shree Guru Gobind Singh Tricentenary University, Gurugram, India; Department of Economics (S Kumar PhD), Central University of Haryana, Jant-Pali, India; Lincoln Institute of Rural and Coastal Health (O P Kurmi PhD), University of Lincoln, Lincoln, England; Department of Medicine (O P Kurmi PhD), Department of Psychiatry and Behavioural Neurosciences (Prof A T Olagunju PhD), McMaster University, Hamilton, ON, Canada; National Research and Innovation Agency, Bogor,

Indonesia (A Kusnali MA); Department of Public Health and Epidemiology (D Kusuma DSc), Khalifa University of Science and Technology, Abu Dhabi, United Arab Emirates; Faculty of Public Health (D Kusuma DSc), University of Indonesia, Depok, Indonesia; Clinical Research Center (V Kytö MD), Turku University Hospital, Turku, Finland; Heart Center (V Kytö MD), University of Turku and Turku University Hospital, Turku, Finland; Department of Clinical Sciences and Community Health (Prof C La Vecchia MD), Department of Food, Environmental and Nutritional Sciences (Prof S Perna PhD), University of Milan, Milan, Italy; Institute for Social and Health Sciences (Prof L Laflamme PhD), University of South Africa, Pretoria, South Africa; Division of Evidence Synthesis (C Lahariya MD), Foundation for People-centric Health Systems, New Delhi, India; Division of Lifestyle Medicine (C Lahariya MD), Centre for Health: The Specialty Practice, New Delhi, India; Department of Psychology (Prof T Lajunen PhD), University of Helsinki, Helsinki, Finland (T J Meretoja MD); Department of Psychology (Prof T Lajunen PhD), Department of Circulation and Medical Imaging (J Nauman PhD), Norwegian University of Science and Technology, Trondheim, Norway; Department of Physiotherapy (T Laksono MS), Universitas Aisyiyah Yogyakarta, Yogyakarta, Indonesia; Institute of Allied Health Sciences (T Laksono MS), National Cheng Kung University, Tainan, Taiwan; Department of Otorhinolaryngology (Prof S Lasrado MS), Father Muller Medical College, Mangalore, India; International Society of Doctors for the Environment, Arezzo, Italy (P Lauriola MD); Department of Clinical and Experimental Medicine (Prof C Ledda PhD), Department of Medical and Surgical Sciences and Advanced Technologies "GF Ingrassia" (Prof M Veroux PhD), University of Catania, Catania, Italy; School of Psychology, Social Work and Public Health (K Lee PhD), Oxford Brookes University, Oxford, UK; Brookes Epidemiological Surveillance Taskforce, Oxford, UK (K Lee PhD); Graduate School of Transdisciplinary Health Sciences (M Lee PhD), Department of Preventive Medicine (Prof E Park PhD), Institute of Health Services Research (Prof E Park PhD), Yonsei University, Seoul, South Korea; Department of Precision Medicine (Prof S Lee MD), Sungkyunkwan University, Suwon-si, South Korea; Department of Rehabilitation Medicine (Prof J Leigh MD), Seoul National University Hospital, Seoul, South Korea; Traffic Injury Rehabilitation Research Institute (Prof J Leigh MD), National Traffic Injury Rehabilitation Hospital, Yangpyeong, South Korea; Department of Cardiothoracic and Vascular Surgery (V Leivaditis PhD), Westpfalz Klinikum, Kaiserslautern, Germany; Department of Cardiothoracic Surgery (V Leivaditis PhD), University of Patras, Patras, Greece; Department of Pharmacy (D A Lemma MSc), College of Medicine and Health Science (A A Mekonnen PhD), Wachemo University, Hosanna, Ethiopia; Department of Health Promotion and Health Education (M Li PhD), National Taiwan Normal University, Taipei, Taiwan; Department of Health Management Center (X Li PhD), Fudan University, Shanghai, China; School of Nursing (Y Li PhD), Department of Rehabilitation Sciences (J S Usman PhD), Hong Kong Polytechnic University, Hong Kong, China; School of Medicine (Z Li PhD), Shanghai Jiao Tong University, Shanghai, China; Clinical Sciences Department (A Liaqat FCPS), Shaqra University, Sharqa, Saudi Arabia; Lerner Research Institute (Prof X Liu PhD), Department of Cardiovascular Medicine (J Rajendran MD), Cleveland Clinic, Cleveland, OH, USA; Department of Quantitative Health Science (Prof X Liu PhD), Case Western Reserve University, Cleveland, OH, USA; School of Medicine (J López-Gil PhD), Universidad Espíritu Santo, Samborondón, Ecuador (J Sharifi Rad PhD); Vicerrectoría De Investigación Y Postgrado (J López-Gil PhD), Universidad De Los Lagos, Osorno, Chile; Ashok & Rita Patel Institute of Physiotherapy (S D Lourembam PhD, D Modi MPT, B U Patel MPT), Department of Physiotherapy (H M Patel PhD), Charotar University of Science and Technology, Anand, India; Department of Community and Family Medicine (A Ludhiadch PhD), Department of Radiodiagnosis (P Singh MD), Department of Paediatrics (A Wander DM), All India Institute of Medical Sciences, Bathinda, India; Dodoma Medical Research Centre (A M Lutambi PhD), National Institute for

Medical Research in Tanzania, Dodoma, Tanzania; College of Engineering (Prof M D Lytras PhD), Effat University, Jeddah, Saudi Arabia; Management of Information Systems Department (Prof M D Lytras PhD), The American College of Greece, Aghia Paraskevi, Greece; Department of Medicine (E Lytvyak MD), Division of Preventive Medicine (Prof S Straube DPhil), School of Public Health (Prof S Straube DPhil), University of Alberta, Edmonton, AB, Canada; Centre for Public Health and Wellbeing (Z Ma PhD), University of the West of England, Bristol, England; Department of Periodontology (Prof M Machoy-Rakoczy PhD), Clinical Emergency Department (B Miazgowski MD), Pomeranian Medical University, Szczecin, Poland (B Miazgowski MD); Thamar University, Dhamar, Yemen (Prof A A Madfa PhD); Ophthalmology Department (M Magdy Abd El Razek PhD), Dr. Soliman Fakeeh Hospital, Riyadh, Saudi Arabia; Department of Emergency Medicine and Trauma (S Mahalingam MD), Karpaga Vinayaga Educational Lore for Learning (Deemed-to-be-University), TamilNadu, India; Department of One Health in Tropical Infectiousness Diseases (S A Mahamed MSc), Jigjiga University, Jigjiga, Ethiopia; College of Health Science (S A Mahamed MSc), Amoud University (Prof A Muse PhD), Amoud University, Borama, Somalia; Research Center (N H Mahmood PhD), College of Health Sciences (F M Rahman PhD), Cihan University-Sulaimaniya, Sulaymaniyah, Iraq; Department of Primary Care and Public Health (Prof A Majeed MD, Prof S Rawaf MD, C Tabche MSc), The George Institute for Global Health (Prof S Yaya PhD), Imperial College London, London, England; INSERM, Imaging Brain & Neuropsychiatry iBraiN (Prof I L Maldonado PhD), Université De Tours, Tours, France; Service De Médecine Et Chirurgie Bucco-Dentaire (Prof I L Maldonado PhD), Tours University Hospital, Tours, France; Department of Maternal-Child Nursing and Public Health (Prof D C Malta PhD), Faculty of Dentistry (Prof G C B D Silva PhD), Federal University of Minas Gerais, Belo Horizonte, Brazil; Internal Medicine Department (L Manjani MD), MedStar Health, Washington, DC, USA; Department of Non-communicable Diseases and Mental Health (R Martinez-Piedra BSc), Pan American Health Organization, Washington, DC, USA; Department of Community Medicine (J Mary MD), Sri Balaji Vidyapeeth (Deemed to Be University), Pondicherry, India; Faculty of Humanities and Health Sciences (Prof R R Marzo MD), Curtin University, Sarawak, Malaysia; Jeffrey Cheah School of Medicine and Health Sciences (Prof R R Marzo MD), Monash University, Subang Jaya, Malaysia; Economics Department (S Massahi MSc), University of Oklahoma, Norman, OK, USA; Department of Anatomy and Developmental Biology (Y Mathangasinghe PhD), Department of Neuroscience (Prof C A Unsworth PhD), Monash University, Clayton, VIC, Australia; Department of Community Medicine (N Mathur MD, S Yahoo MD), Apollo Institute of Medical Sciences and Research, Hyderabad, India; Digital Health and Informatics Directorate (Prof S M McPhail PhD), Queensland Health, Brisbane, QLD, Australia; College of Medicine and Department of Nursing (A A Mekonnen PhD), Komar University of Science and Technology, Kurdistan Sulemaniya, Iraq; School of Medicine and Institute of Public Health (T B Melkie PhD), College of Medicine and Health Sciences (N A Negash MSc), University of Gondar, Gondar, Ethiopia; Department of Civil Engineering (Prof O Melo Neto DSc), Federal University of Lavras, Lavras, Brazil; Dirección General De Investigación, Desarrollo E Innovación (DGIDI) (W Mendoza MD), Universidad Científica Del Sur (University of the South), Lima, Peru; Department of Medical Microbiology and Immunology (G A Menezes PhD), Trinity Medical Sciences University, St. Vincent, Saint Vincent and the Grenadines; Comprehensive Cancer Center (T J Meretoja MD), Helsinki University Hospital, Helsinki, Finland; University Centre Varazdin (T Mestrovic PhD), University North, Varazdin, Croatia; Department of Pharmacology (Prof C D K Mettananda PhD), Department of Paediatrics (Prof S Mettananda DPhil), University of Kelaniya, Ragama, Sri Lanka; Clinical Medicine Department (Prof C D K Mettananda PhD), University Paediatrics Unit (Prof S Mettananda DPhil), Colombo North Teaching Hospital, Ragama, Sri Lanka; Department of Pathology (Prof M M M Metwally

PhD), Cardiovascular Department (Prof A M A Saad MD), Zagazig University, Zagazig, Egypt; College of Human Medicine (T R Miller PhD), Michigan State University, Flint, MI, USA; Multidisciplinary Department of Medical-Surgical and Dental Specialties (G Minervini PhD), University of Campania Luigi Vanvitelli, Naples, Italy; School of Pharmacy (Prof L Ming PhD), Sunway University, Sunway City, Malaysia; Department of Forensic Medicine and Toxicology (C Mittal MD), All India Institute of Medical Sciences, Patna, India; RAK College of Nursing (M Mohamed PhD), RAK Medical and Health Sciences University, Ras Al-Khaimah, United Arab Emirates; Nursing College (M Mohamed PhD), Sohag University, Sohag, Egypt; Molecular Biology Unit (N S Mohamed MSc), Bio-Statistical and Molecular Biology Department (N S Mohamed MSc), Sirius Training and Research Centre, Khartoum, Sudan; Department of Pathophysiology, St.George's University, School of Medicine (K Mohamed Ahmed MD), St. George's University, St.George's, Grenada; Department of Information Technology (M Mohammadi PhD), Lebanese French University, Erbil, Iraq; Center of Excellence in Precision Medicine and Digital Health (M Mohammadi PhD), Center of Excellence in Precision Medicine and Digital Health, Department of Physiology, Faculty of Dentistry (Prof A Rehman PhD), Chulalongkorn University, Bangkok, Thailand; Health Systems and Policy Research Unit (Prof S Mohammed PhD), Department of Community Medicine (A A Olorukooba MD), Ahmadu Bello University, Zaria, Nigeria; Department of Health Services Management (M Mohseni PhD), Iran University of Medical Sciences, Iran, Iran; AI & Cyber Futures Institute (M Moni PhD), Charles Sturt University, Bathurst, NSW, Australia; Health Systems Research Center (Prof J C Montañez MSc), National Health Research Institutes, Cuernavaca, Mexico; Department of Public Health (Prof R S Moreira PhD), Oswaldo Cruz Foundation, Recife, Brazil; Department of Public Health (Prof R S Moreira PhD), Federal University of Pernambuco, Recife, Brazil; Division of Plastic and Reconstructive Surgery (S D Morrison MD), University of Washington Medical Center, Seattle, WA, USA; American Society for Inclusion, Diversity, and Equity in Healthcare, Delaware, DE, USA (M Morsy MD); Unit of Pharmacotherapy, Epidemiology and Economics (Prof S Mubarik PhD), University of Groningen (Rijksuniversiteit Groningen), Groningen, Netherlands; Department of Epidemiology and Biostatistics (Prof S Mubarik PhD), School of Public Health (Prof Z Zhang PhD), Wuhan University, Wuhan, China; College of Nursing (S Mudgal PhD), Dr. Ram Manohar Lohia Institute of Medical Sciences, Lucknow, India; Department of Physiotherapy (M Muhammad PhD), Tishk International University, Sulaimaniyah, Iraq; Knowledge Management Department (S Mukherjee PhD), Prahlad Omkarwati Foundation (POF), Mumbai, India; Changescape Consulting (S Mukherjee PhD), Changescape Consulting, New Delhi, India; Department of Surgery (F Mulita PhD), General University Hospital of Patras, Patras, Greece; Faculty of Medicine (F Mulita PhD), University of Thessaly, Larissa, Greece; Department of Health Economics (M Muniyandi PhD), Department of Epidemiology (M Sathya Narayanan MBBS), Department of Clinical Research (V Srinivasalu MBBS), National Institute for Research in Tuberculosis, Chennai, India; Clinical Epidemiology Research Unit (E Murillo-Zamora PhD), Mexican Institute of Social Security, Villa de Alvarez, Mexico; Postgraduate in Medical Sciences (E Murillo-Zamora PhD), Universidad De Colima, Colima, Mexico; Centre for Early Childhood Caries Research (Prof S Murugan PhD), Sri Ramachandra University, Chennai, India; Santosh University, New Delhi, India (Prof S Murugan PhD); Faculty of Physiotherapy (Prof B Muruganantham PhD), Sigma University, Anand, India; School of Public Health (D Musoke PhD), Makerere University, Kampala, Uganda; Department of Research Methods (S Muthu PhD), Orthopaedic Research Group, Coimbatore, India; Central Research Laboratory (S Muthu PhD), Vinayaka Mission's Research Foundation (Deemed to Be University), Puducherry, India; Elderly Health Research Center (A Nafei PhD), Research and Academic Institution, Tehran, Iran; Department of Computer Science and IT (G R Naik PhD), Torrens University, Adelaide, SA, Australia; Department of Health and

Rehabilitation Sciences (Prof G Nambi PhD), Prince Sattam Bin Abdulaziz University, Al Kharj, Saudi Arabia; Suraj Eye Institute, Nagpur, India (V Nangia PhD); Division of Endocrinology and Diabetes (M Nassar PhD), University of Vermont, South Burlington, VT, USA; Amity Institute of Forensic Sciences (B P Nayak PhD), Amity Institute of Public Health and Hospital Administration (A Singh PhD, P S Singh PhD), Amity University, Noida, India; College of Public Health (K Nayak MPH), Kent State University, Kent, OH, USA; Department of Pharmacy (I Nayila PhD), The University of Lahore, Sargodha, Pakistan; Department of Molecular Biology and Chemistry (I Nayila PhD), Sargodha Medical College, Sargodha, Pakistan; TroDDIVaT Initiative (G Nchanji PhD), Frontline Association, Buea, Cameroon; Department of Microbiology and Parasitology (G Nchanji PhD), University of Buea, Buea, Cameroon; Department of General Surgery (I Negoi PhD), Emergency University Hospital Bucharest, Bucharest, Romania; Faculty of Public Health (T Nguyen PhD), VNU University of Medicine and Pharmacy, Hanoi, Viet Nam; Department of General Surgery (T K Nikolouzakis PhD), University Hospital of Heraklion, Heraklion, Greece; Laboratory of Toxicology (T K Nikolouzakis PhD), University of Crete, Heraklion, Greece; Department of Epidemiology and Health Statistics (Prof P Ning PhD), Central South University, Changsha, China; Public Health Department (D N A Ningrum PhD), Universitas Negeri Semarang (State University of Semarang), Kota Semarang, Indonesia; School of Medicine (V Niranjan PhD), University of Limerick, Limerick, Ireland; School of Health and Wellbeing (R Nketia MSc), University of Glasgow, Glasgow, Scotland; Department of Global Health Policy and Development (R Nketia MSc), Sub-Saharan Africa Research for Sustainable Development (SSARSD) Project, Berekum, Ghana; Global Health Policy Lab (Prof S Nomura PhD), Tohoku University, Miyagi, Japan; Department of Health Policy and Management (Prof S Nomura PhD), Keio University, Tokyo, Japan; Department of Paediatrics (C A Nri-Ezedi PhD), Nnamdi Azikiwe University, Awka, Nigeria; Global Health Department (J Nshimiyimana MPH), Euclid University, Banqui, Central African Republic; Center of Excellence in Reproductive Health Innovation (CERHI) (C I Nzoputam MPH), University of Benin, Benin City, Nigeria; Department of Physiology (O J Nzoputam PhD), University of Benin, Edo, Nigeria; Department of Physiology (O J Nzoputam PhD), Benson Idahosa University, Benin City, Nigeria; Department of Applied Economics and Quantitative Analysis (Prof B Oancea PhD), University of Bucharest, Bucharest, Romania; Bioinformatics Department (Prof B Oancea PhD), National Institute of Research and Development for Biological Sciences, Bucharest, Romania; Department of Biomedicine and Prevention (F Oddi PhD), University of Rome "Tor Vergata", Rome, Italy; Translational Health Research Institute (F A Ogbo PhD), Western Sydney University, Sydney, NSW, Australia; Department of Physiotherapy (O O Ojedoyin PhD), Federal University of Health Sciences Ila Orangun Osun, Ila-Orangun, Nigeria; Health Promotion Research Center (H Okati-Aliabad PhD), Zahedan University of Medical Sciences, Zahedan, Iran; School of Pharmacy (O C Okonji MSc), University of the Western Cape, Cape Town, South Africa; Department of Psychiatry (Prof A T Olagunju PhD), University of Lagos, Lagos, Nigeria; School of Health and Life Sciences (O O Oludoye PhD), Teesside University, Middlesbrough, UK; Jadara Research Center (A Omar Bali PhD), Jadara University, Irbid, Jordan; Surgery Department (G L Omer MD), Sulaimani University, Sulaimani, Iraq; ENT Department (G L Omer MD), Tor Vergata University of Rome, Rome, Italy; Centre of Regenerative Medicine (M Ordak PhD), Medical University of Bialystok, Bialystok, Poland; Institute of Family Medicine and Public Health (Prof H Orru PhD), University of Tartu, Tartu, Estonia; Section of Sustainable Health (Prof H Orru PhD), Umeå University, Umea, Sweden; Department of Biological Sciences (A Osborne MSc), Entomology VectorLink (F S Samura MPH), Njala University, Freetown, Sierra Leone; Division of Infectious Diseases (Prof A Ouyahia PhD), University Hospital of Setif, Sétif, Algeria; School of Medicine (T Oyelade PhD), Keele University, Keele, England; Department of Respiratory Medicine (Prof M P A DNB), Jagadguru Sri

Shivarathreeswara Academy of Health Education and Research, Mysore, India; Universidad San Ignacio De Loyola, Lima, Peru (K Pacheco-Barrios MD); Research Department (A Pandey MPH, U Paudel PhD), Nepal Health Research Council, Kathmandu, Nepal; Research Department (A Pandey MPH), Public Health Research Society Nepal, Kathmandu, Nepal; The Royal Marsden NHS Foundation Trust (S R Pandey PhD), The Royal Marsden NHS Foundation Trust, London, England; Faculty of Science and Engineering, School - Computing and Information Science (S R Pandey PhD), Anglia Ruskin University, London, England; Centre for Research and Development (Prof S R Pandi-Perumal MSc), Chandigarh University, Punjab, India; Division of Research and Development (Prof S R Pandi-Perumal MSc), Lovely Professional University, Phagwara, India; Division of Ophthalmology & Visual Sciences (Prof G D Panos MD), University of Nottingham, Nottingham, England; First Department of Ophthalmology (Prof G D Panos MD), Second Propedeutic Department of Internal Medicine (Prof D Patoulias PhD), Aristotle University of Thessaloniki, Thessaloniki, Greece; Medical University of Vienna, Vienna, Austria (I Papadimopoulos MD); School of Health and Biomedical Sciences (Prof E Pappas PhD), Royal Melbourne Institute of Technology (RMIT) University, Melbourne, VIC, Australia; Vision and Eye Research Institute (Prof S Pardhan PhD, M Piyasena PhD), Anglia Ruskin University, Cambridge, England; Department of Sociology, Anthropology, and Public Health (C Park DrPH), University of Maryland Baltimore County, Baltimore, MD, USA; Forensic Medicine and Toxicology (P Parmar MD), Department of Community Medicine and Family Medicine (A M Peddireddy MD), All India Institute of Medical Sciences, Hyderabad, India; School of Nursing (A Pashaei MSc), University of British Columbia, Vancouver, BC, Canada; Department of Medical Sciences (R Passera PhD), University of Torino, Torino, Italy; Department of Imaging (R Passera PhD), AOU Città Della Salute E Della Scienza Di Torino (AOU City of Health and Science of Turin), Torino, Italy; Department of Cardiology (K Patel MD), University of Kansas Medical Center, Kansas City, KS, USA; Department of Cardiology (K Patel MD), US Department of Veterans Affairs (VA), Kansas City, MO, USA; Department of Research and Training (S K Patel PhD), Population Council Institute, New Delhi, India; College of Dental Medicine (Prof S Patil PhD), Roseman University of Health Sciences, South Jordan, UT, USA; Department of Genetics (S Pawar PhD), Yale University, New Haven, CT, USA; School of Population Health (A E Peden PhD), University of New South Wales, Kensington, NSW, Australia; Department of Public Health (J Pekarcikova PhD), Trnava University, Trnava, Slovakia; Center for Research and Innovation (V F Pepito MSc), Ateneo De Manila University, Pasig City, Philippines; Australian Institute of Health Innovation (P Peprah MSc), Macquarie University, Sydney, NSW, Australia; Fellowship (D A Perez-Chadid MD), The G4 Alliance, Chicago, IL, USA; Department of Basic Medical Sciences (J D Pillay PhD), Durban University of Technology, Durban, South Africa; Mental Health Research Institute (E Plotnikov PhD), Tomsk National Research Medical Center, Tomsk, Russia; Siberian State Medical University, Tomsk, Russia (E Plotnikov PhD); Department of Occupational Health Engineering (R Pourbabaki PhD), Sirjan School of Medical Sciences, Sirjan, Iran; Department of Occupational Health and Safety Engineering (Prof M Poursadeqiyan PhD), Ardabil University of Medical Science, Ardabil, Iran; Non-communicable Diseases Research Center (N Pourtaheri PhD), Bam University of Medical Sciences, Bam, Iran; Centro De Investigaciones Clinicas (Clinical Research Center) (S I Prada PhD), Fundación Valle Del Lili (Valle Del Lili Foundation), Cali, Colombia; Centro PROESA (S I Prada PhD), Universidad ICESI, Cali, Colombia; Institute of Microbiology (M Qamar PhD), Government College University Faisalabad, Faisalabad, Pakistan; College of Public Health (M Qodrati MD), George Mason University, Fairfax, VA, USA; Faculty of Nursing (N Qur'aniati PhD), Universitas Airlangga, Surabaya, Indonesia; Research Center in Advancing Community Healthcare, Surabaya, Indonesia (N Qur'aniati PhD); Department of Medical Oncology (Prof V Radhakrishnan MD), Cancer Institute (W.I.A), Chennai,

India; Ingram School of Engineering (A Rafe PhD), Texas State University, San Marcos, TX, USA; Health Research Institute (HRI) (I Rafique PhD), National Institutes of Health, Islamabad, Pakistan; Department of Epidemiology (P Raghuveer MD), National Institute of Mental Health and Neurosciences, Bengaluru, India; Department of Environmental Health Engineering (S Rahimi PhD), Health Science Research Centre (S Rahimi PhD), Health Sciences Research Center (M Sarmadi MSc), Torbat Heydariyeh University of Medical Sciences, Torbat Heydariyeh, Iran; Department of Public Health (F S Rahman PhD), Universitas Airlangga (Airlangga University), Surabaya, Indonesia; Department of Biology (F M Rahman PhD), University of Sulaimani, Sulaymaniyah, Iraq; College of Medicine and Health Sciences (M Rahman PhD), National University of Science and Technology, Sohar, Oman; Department of Population Science and Human Resource Development (Prof M Rahman DrPH), University of Rajshahi, Rajshahi, Bangladesh; Institute of Health and Wellbeing (Prof M Rahman PhD), Federation University Australia, Berwick, VIC, Australia; Future Technology Research Center (A Rahmani PhD), National Yunlin University of Science and Technology, Yunlin, Taiwan; Pharmaceutical Analysis (A Rai PhD), National Institute of Pharmaceutical Education and Research, Hajipur, India; Dr. Rajendra Prasad Government Medical College, Tanda, Kangra, India (Prof S K Raina MD); Department of Population Health (M Ramadan DrPH), King Saud Bin Abdulaziz University for Health Sciences, Jeddah, Saudi Arabia; Department of Midwifery (K Ramadhan MPH), Ministry of Health of the Republic of Indonesia, Palu, Indonesia; Department of Radiology (S Ramasamy MD), Stanford University, Stanford, CA, USA; National Institute for Research in Tuberculosis (B Ramraj MD, D R S MPH), Indian Council of Medical Research, Chennai, India; Centre for Clinical Pharmacology (Prof N Rancic PhD), Military Medical Academy, Belgrade, Serbia; Division of Pediatric Surgery (A G Rao MBBS), Stanford University, Palo Alto, CA, USA; Nitte (Deemed to Be University), AB Shetty Memorial Institute of Dental Sciences (K Rao PhD), KS Hegde Medical Academy (Prof P Shetty MD, S S Shetty MD), Nitte University, Mangalore, India; Department of Oral Pathology, Microbiology and Forensic Odontology (S J Rao MDS), Sharavathi Dental College and Hospital, Shimogga, India; Department of Family Medicine (Prof D Rathish PhD), Rajarata University of Sri Lanka, Anuradhapura, Sri Lanka; Department of Public Health (R Ravi MHAHSM), Amrita Institute of Medical Sciences, Ernakulam, India; Academic Public Health England (Prof S Rawaf MD), Public Health England, London, England; Internal Medicine Department (K Rayapureddi MD), Armed Forces Medical College, Pune, India; Computer Science and Engineering Department (Prof A Rehman PhD), Saveetha Institute of Medical and Technical Sciences, Chennai, India; Department for Epidemiology and Biostatistics (R Reile PhD), National Institute for Health Development, Tallinn, Estonia; School of Environment (M Rezaei PhD), Tehran University, Tehran, Iran; Department of Epidemiology and Biostatistics (Prof M Rezaeian PhD), Rafsanjan University of Medical Sciences, Rafsanjan, Iran; Faculty of Allied Health Sciences (Prof M R Rizvi PhD), Santosh (Deemed to Be University), Delhi, India; Department of Pharmacology and Toxicology (Prof J A B Rodriguez PhD), University of Antioquia, Medellin, Colombia; Warwick Medical School (Prof J A B Rodriguez PhD), University of Warwick, Coventry, England; Department of Clinical Research (Prof L Roever PhD), University of Sao Paulo, Ribeirão Preto, Brazil; Department of Community Medicine (R Rohilla MD), Government Medical College, Chandigarh, India; Faculty of Medicine (B Roy PhD), Quest International University Perak, Ipoh, Malaysia; Communicable Disease Surveillance Centre (P Rubeshkumar MPH), Public Health Wales, Cardiff, Wales; Department of Oral and Maxillofacial Surgery (M S MDS), Jagadguru Sri Shivarathreeswara University, Mysore, India; Department of Pharmaceutical Chemistry (Prof M Saeb PhD), International Medical University, Gdańsk, Poland; Széchenyi István University (Prof U Saeed PhD), Egyetem Square 1 (Prof Y Waheed PhD), Széchenyi István University, Győr, Hungary; Irfan Suat Gunsel Operational Research Institute (Prof U Saeed PhD),

Near East University, Turkey, Nicosia, Türkiye; Faculty of Medicine (S Safi PhD), MAHSA University, Selangor, Malaysia; Community Medicine and Family Medicine (D Sahoo MD), All India Institute of Medical Sciences, Bibinagar, Hyderabad, India; Department of Psychiatry (Z Saif MBA), Ministry of Health, Manama, Bahrain; LMU-Munich, Munich, Germany (J W Sakshaug PhD); Institute for Employment Research, Nuremberg, Germany (J W Sakshaug PhD); Cephas Health Research Initiative Inc, Ibadan, Nigeria (A A Salami BDS); Department of Human Nutrition & Dietetics (S Saleem PhD), Public Health Department (E Yaqoob PhD), Health Services Academy, Islamabad, Pakistan; Surgical Department (J Samaranayake MBBS), North Colombo Teaching Hospital, Ragama, Sri Lanka; Institute of Epidemiology and Preventive Medicine (Y L Samodra PhD), National Taiwan University, Taipei, Taiwan; Benang Merah Research Center (Y L Samodra PhD), Benang Merah Research Center (BMRC), Minahasa Utara, Indonesia; Department of Anatomy (Prof V P Samuel PhD), Ras Al Khaimah Medical and Health Sciences University, Ras Al Khaimah, United Arab Emirates; Department of Entomology (A M Samy PhD), Medical Ain Shams Research Institute (MASRI) (A M Samy PhD), Ain Shams University, Cairo, Egypt; Department of Pediatrics (Prof R Sanjeev MD), SRM Medical College Hospital And Research Centre, Kattankulathur, India; Department of Pharmacology (A Saravanan MD, M Shamim MBBS, S Singh MD), All India Institute of Medical Sciences, Jodhpur, India; Indira Gandhi Medical College and Research Institute, Puducherry, India (A Saravanan MD); Department of Public Health (Y Sarikhani PhD), Jahrom University of Medical Sciences, Jahrom, Iran; Faculty of Science (M Sarmadi MSc), Queensland University of Technology, Brisbane, QLD, Australia; Department of Oral Pathology and Microbiology (Prof S C Sarode PhD), Dr. D. Y. Patil Vidyapeeth, Pune (Deemed to Be University), Pune, India; Udyam-Global Association for Sustainable Development, Bhubaneswar, India (Prof M Satpathy PhD); Department of Food Processing and Nutrition (J Saulam PhD), Karnataka State Akkamahadevi Women University, Vijayapura, India; Department of Preventive and Social Medicine (G Saya MD), Jawaharlal Institute of Postgraduate Medical Education and Research, Puducherry, India; Universidad ESAN (Prof C Schinckus PhD), Universidad ESAN, Lima, Peru; Graduate School of Business (Prof C Schinckus PhD), University of the Fraser Valley, Abbotsford, BC, Canada; Office of the Vice President for Research (D C Schwebel PhD), University of Iowa, Iowa City, IA, USA; Faculty of Dentistry (Prof S Selvaraj PhD), University of Puthisastra, Phnom Penh, Cambodia; Emergency Department (S Senthilkumaran PhD), Manian Medical Centre, Erode, India; Department of Psychology and Health Sciences Faculty of Human Sciences, Education, and Sports (Prof F Sessa PhD), Pegaso Telematic University, Naples, Italy; Department of Medicine (Y Sethi MD), Swami Vivekanand Subharti University, Meerut, India; Charotar University of Science and Technology (CHARUSAT) (S A Shah MPT), Ashok and Rita Patel Institute of Physiotherapy, Changa, India; Rita A. Patel Institute of Physiotherapy (S Shah PhD), The Charutar Vidya Mandal(CVM) University, Anand, India; School of Health Sciences (S Shaharudin PhD), Universiti Sains Malaysia, Kota Bharu, Malaysia; National Institute of Education (S Shaharudin PhD), Nanyang Technological University, Singapore, Singapore; Center for Medical and Bio-Allied Health Sciences Research (Prof M J Shahwan PhD), Ajman University, Ajman, United Arab Emirates; Independent Consultant, Karachi, Pakistan (M A Shaikh MD); Trauma Research Center (A Shams MD), Shiraz University of Medical Sciences, shiraz, Iran; Lancaster University, Lancaster, England (D Shan PhD); Columbia University, New York, NY, USA (D Shan PhD); Department for Evidence-based Medicine and Evaluation (A Sharifan PharmD), University for Continuing Education Krems, Krems, Austria; School of Science and Technology (Prof P Sharifian Jazi PhD), The University of Georgia, Tbilisi, Georgia; Department of Biotechnology (B Sharma PhD), Graphic Era (Deemed to Be University), Dehradun, India; Department of Physiotherapy (J Sharma PhD), Galgotas University, Greater Noida, India; Department of University Institute of Biotechnology (R Sharma PhD),

Chandigarh University, Mohali, India; Michael J. Cousins Pain Management and Research Centre (S Sharma PhD), Royal North Shore Hospital, Sydney, NSW, Australia; School of Public Health (S Shawel MPH), Department of Epidemiology and Biostatistics (B S Tusa MPH), Haramaya University, Harar, Ethiopia; Psychology Department (J Shen PhD), University of Massachusetts Lowell, Boston, MA, USA; Department of Biology (S P Sherchan PhD), Morgan State University, Baltimore, MD, USA; Alva's Institute of Medical Sciences & Research Centre (Prof M Shetty MD), Rajiv Gandhi University of Health Sciences, Moodubidire, India; Department of Veterinary Public Health and Preventive Medicine (A Shittu MSc), Usmanu Danfodiyo University, Sokoto, Sokoto, Nigeria; Department of Neurosurgery (N A Shlobin MD), Columbia University Medical Center, New York, NY, USA; Department of Medical-Surgical Nursing (S Shorofi PhD), Mazandaran University of Medical Sciences, Sari, Iran; Kenneth H. Cooper Institute (Prof K Shuval PhD), Texas Tech University Health Sciences Center, Dallas, TX, USA; TMSS Nursing College, Bogura (M Siddique MPH), TMSS Grand Health Sector, Bogura, Bangladesh, Bogura, Bangladesh; MS Foundation Healthcare & Physiotherapy (M Siddique MPH), MS Foundation Healthcare & Physiotherapy, Sherpur, Bangladesh; Research and Innovation (Prof P P Simkhada PhD), University of Chester, Chester, UK; Department of Pharmacology (H Singh DM), Government Medical College and Hospital, Chandigarh, India; Department of Community Medicine (S Singh MD), Veer Chandra Singh Garhwali Government Institute of Medical Science and Research, Srinagar Garhwal, India; Global and European Health Education and Study Institute (Prof N Skhvitaridze PhD), University of Georgia, Tbilisi, Georgia; National Center for Disease Control and Public Health, Tbilisi, Georgia (Prof N Skhvitaridze PhD); Books Committee (V Y Skryabin MD), Royal College of Psychiatrists, London, UK; Department of Surgery (B Socea PhD), "Sf. Pantelimon" Emergency Clinical Hospital Bucharest, Bucharest, Romania; Department of Development Studies (M Sohel MPH), Daffodil International University, Dhaka, Bangladesh; Department of Medicine (Y Song MD), Washington University in St. Louis, Saint Louis, MO, USA; Department of Health (L V Sree MD), A.C.S. Medical College and Hospital, Hyderabad, India; Department of Public Health (M Stanikzai MPH), Kandahar University, Kandahar, Afghanistan; Department of Medicine (P Steiropoulos MD), Democritus University of Thrace, Alexandroupolis, Greece; McGill University, Montreal, QC, Canada (C Su PhD); Department of Orthopaedics (O Subasi PhD), Harvard University, Cambridge, MA, USA; Department of Medical Sciences (Prof V Subramaniyan PhD), Sunway University, Subang Jaya, Malaysia; Department of Nursing (A Sudaryanto MPH), Muhammadiyah University of Surakarta, Surakarta, Indonesia; Praboromarajchanok Institute (T Sukaew PhD), Ministry of Public Health, Nonthaburi, Thailand; Data Science Institute (Prof J Sun PhD), University of Technology Sydney, Sydney, NSW, Australia; Heart Research Institute (Prof J Sun PhD), University of Sydney, Sydney, QLD, Australia; Gandhi Medical College (S Sundaragiri MD), Kaloji Narayana Rao University of Health Sciences (KNRUHS), Secunderabad, India; Institute of Neuroscience and Physiology (Prof K S Sunnerhagen PhD), University of Gothenburg, Gothenburg, Sweden; Department of Rehabilitation Medicine (Prof K S Sunnerhagen PhD), Sabzevar University of Medical Sciences, Gothenburg, Sweden; Institute of Medical Sciences (Prof L Szarpak PhD), The John Paul II Catholic University of Lublin, Lublin, Poland; Department of Medicine (Prof R Tabarés-Seisdedos PhD), University of Valencia, Valencia, Spain; Carlos III Health Institute (Prof R Tabarés-Seisdedos PhD), Biomedical Research Networking Center for Mental Health Network (CiberSAM), Madrid, Spain; Department of Health, Safety, and Environmental Management (R Tabibi PhD), Abadan University of Medical Sciences, Abadan, Iran; Department of Biostatistics and Epidemiology (M Taheri Soodejani PhD), Shahid Sadoughi University of Medical Science, Yazd, Iran; Department of Dermato-Venereology (M Tampa PhD), Dr. Victor Babes Clinical Hospital of Infectious Diseases and Tropical Diseases, Bucharest, Romania; Department of Public Health (M K Tariku

MPH), Debre Markos University, Debre Markos, Ethiopia; Department of Public Health and Informatics (A Tasnim MPH), Bangladesh Medical University, Dhaka, Bangladesh; Department of Public Health (E Tel MD), Trakya University, Faculty of Medicine, Türkiye, Edirne, Türkiye; College of Medicine (Prof M Temsah MD), College of Pharmacy (R Temsah PharmD), Alfaisal University, Riyadh, Saudi Arabia; Department of Psychology (E Teye-Kwadjo PhD), University of Ghana, Legon, Ghana; Department of Industrial Psychology (E Teye-Kwadjo PhD), Stellenbosch University, Cape Town, South Africa; Research Department (D Thapa PhD), Policy Research Institute, Kathmandu, Nepal; Department of Economics (I Tharwat PhD), The American University in Cairo, Cairo, Egypt; Forensic Medicine & Toxicology (H R Thube MD), College of Nursing (R V R MSc), All India Institute of Medical Sciences, Nagpur, India; Faculty of Public Health (J H V Ticoalu MPH), Universitas Sam Ratulangi (Sam Ratulangi University), Manado, Indonesia; College of Nursing (S I Tonapa PhD), Kaohsiung Medical University, Taiwan, Kaohsiung, Taiwan; Global Surgery Department (A Touko MSc), Data Science Center for the Study of Surgery, Injury and Equity in Africa, Buea, Cameroon; Department of Internal Medicine (T H Tran MD), University of Medicine and Pharmacy at Ho Chi Minh City, Ho Chi Minh City, Viet Nam; Department of Business Analytics (T H Tran MD), University of Massachusetts Dartmouth, Dartmouth, MA, USA; Research and Advocacy Initiative (N Tran Minh Duc MD), ALS Vietnam, Quang Ngai, Viet Nam; Department of Clinical and Experimental Medicine (D Trico MD), University of Pisa, Pisa, Italy; Infectious Disease Epidemiology Research Group (L S Tuglo MPH), Kwame Nkrumah University of Science and Technology, Kumasi, Ghana; Department of Internal Medicine (M Tumurkhuu PhD), Wake Forest University, Winston-Salem, NC, USA; University Hospitals Cleveland Medical Center, Cleveland, OH, USA (I Ullah MBBS); International Center for Chemical and Biological Sciences (S Ullah MSc), University of Karachi, Karachi, Pakistan; Department of Biology and Biochemistry (S Ullah MSc), University of Houston, Houston, TX, USA; Independent Consultant, St. Augustine, Trinidad and Tobago (S Umakanthan MD); North Central Regional Health Authority, Champ Fleurs, Trinidad and Tobago (S Umakanthan MD); Department of Physiotherapy (L Umar PhD), Federal Ministry of Health, Gusau, Nigeria; Institute of Health and Wellbeing (Prof C A Unsworth PhD), Federation University Australia, Churchill, VIC, Australia; Amity Institute of Biotechnology (E Upadhyay PhD), Amity University Rajasthan, Jaipur, India; Symbiosis College of Nursing (S Upendra PhD), Symbiosis International University, Pune, India; Department of Physiotherapy (J S Usman PhD), Bayero University, Kano, Nigeria; College of Health and Sport Sciences (A G Vaithinathan MSc), University of Bahrain, Zallaq, Bahrain; Sociedad Argentina De Medicina, Buenos Aires, Argentina (Prof P R Valdez PhD); Hospital Vélez Sarsfield, Buenos Aires, Argentina (Prof P R Valdez PhD); UKK Institute, Tampere, Finland (Prof T J Vasankari PhD); Faculty of Medicine and Health Technology (Prof T J Vasankari PhD), Tampere University, Tampere, Finland; Raffles Neuroscience Centre (Prof N Venketasubramanian MSc), Raffles Hospital, Singapore, Singapore; Siksha 'O' Anusandhan (Deemed to Be University), Bhubaneswar, India (A K Verma PhD); Department of Surgery (G Verras MSc), University of Southampton, Southampton, England; Centro De Investigación En Nutrición Y Salud (Prof V E Villalobos-Daniel PhD), National Institute of Public Health, Mexico City, Mexico; Health Initiative of the Americas (Prof V E Villalobos-Daniel PhD), University of California Berkeley, Berkeley, CA, USA; Radcliffe Department of Medicine (T Vinh BS), University of Oxford, Oxford, England; Occupational Medicine Unit (Prof F S Violante MD), Sant'Orsola Malpighi Hospital, Bologna, Italy; Department of Health Care Administration and Economics (Prof V Vlassov MD), National Research University Higher School of Economics, Moscow, Russia; Irfan Suat Gunsel Operational Research Institute (Prof Y Waheed PhD), Near East University, Nicosia, Türkiye; National Institute of Health Data Science (W Wang PhD), Peking University, Beijing, China; School of Public Health (Prof W Wang PhD), Xuzhou Medical University,

Xuzhou, China; School of Life Course and Population Sciences (Prof Y Wang PhD), King's College London, London, England; Department of Family and Preventive Medicine, Faculty of Medicine (Prof A Wattanapisit PhD), Prince of Songkla University, Songkhla, Thailand; Department of Public Health Sciences (A I Wegbom PhD), Rivers State University, Port Harcourt, Nigeria; Department of Orthopaedics (F Wei PhD), General Hospital of Central Theater Command, Wuhan, China; Fourth Military Medical University, Xi'an, China (F Wei PhD); Demographic Change and Aging Research Area (A Werdecker PhD), Federal Institute for Population Research, Wiesbaden, Germany; Department of Physical Therapy (T Wiangkham PhD), Naresuan University, Phitsanulok, Thailand; Independent Investigator, Washington, DC, USA (F Williams PhD); Research Organisation (A Wireko MD), Inter-Continental Omni-Research in Medicine Collaborative, Berlin, Germany; Department of Intelligent Medical Engineering (Prof W Xie DrPH), Anhui Medical University, Anhui, China; Department of Surgery (Prof W Xie DrPH), The First Affiliated Hospital of Anhui Medical University, Hefei, Anhui, China; School of Public Health Sciences and Technology (P Yadav PhD), Malla Reddy Vishwavidyapeeth, Hyderabad, Telangana, India; Department of Environmental Health and Epidemiology (V Yadav MD), National Institute for Research in Environmental Health, Bhopal, India; Zilber College of Public Health (M Yahaya PhD), University of Wisconsin-Milwaukee, Milwaukee, WI, USA; National Center for Chronic and Noncommunicable Disease Control and Prevention (P Ye PhD), Chinese Center for Disease Control and Prevention, Beijing, China; Department of Public Health (A Yekdeş MD), Trakya University, Edirne, Türkiye; KHANA Center for Population Health Research, Phnom Penh, Cambodia (Prof S Yi PhD); Department of Health Management (A Yiğit PhD, Prof V Yiğit PhD), Süleyman Demirel Üniversitesi (Süleyman Demirel University), Isparta, Türkiye; Community Medicine Department (S Yogeshkumar MD), Jawaharlal Nehru Medical College, KLE Academy of Higher Education and Research, Belagavi, India; Department of Pediatrics (Prof D Yon MD), Kyung Hee University, Seoul, South Korea; Department of Biostatistics (Prof N Yonemoto PhD), University of Toyama, Toyama, Japan; Department of Public Health (Prof N Yonemoto PhD), Juntendo University, Tokyo, Japan; Department of Health Policy and Management (Prof M Younis PhD), Jackson State University, Jackson, MS, USA; School of Business & Economics (Prof M Younis PhD), Tsinghua University, Beijing, China; School of Public Health (Prof Y Yu MS), Hubei University of Medicine, Shiyan, China; Association for Socially Applicable Research (ASAR), Pune, India (S Zadey MS); Department of Emergency Medicine (S Zadey MS), Global Emergency Medicine Innovation and Implementation (GEMINI) Research Center, Durham, NC, USA; Department of Environmental and Occupational Health (E Zainal Abidin PhD), Universiti Putra Malaysia, Serdang, Malaysia; Department of Oncology and Mammology (D Zairov MD), Kazakh Russian Medical University, Almaty, Kazakhstan; Research and Development Department (I Zare BSc), Sina Medical Biochemistry Technologies, Shiraz, Iran; Department of Administration (Prof M Zastrozhin PhD), PGxAI, San Francisco, CA, USA; Mohammed VI International School of Public Health (Prof S Zbiri PhD), Mohammed VI University of Sciences and Health (UM6SS), Casablanca, Morocco; Institute for Healthcare System Analysis (IA2S), Paris, France (Prof S Zbiri PhD); Department of Zoology and Entomology (M G M Zeariya PhD), Al-Azhar University, Cairo, Egypt; School of Public Health (H Zhang PhD), Huazhong University of Science and Technology, Wuhan, China; School of Public Health (Y Zhang PhD), Hubei Province Key Laboratory of Occupational Hazard Identification and Control (Y Zhang PhD), Wuhan University of Science and Technology, Wuhan, China; Department of Surgery (H Zhou MD), Zhejiang University, Zhejiang Province, China; Clinical Research Centre (Prof S H Zyoud PhD), An-Najah National University Hospital, Nablus, Palestine; Department of Building Engineering and Environment (S H Zyoud PhD), Civil Engineering and Sustainable Structures (S H Zyoud PhD), Palestine Technical University (Kadoorie), Tulkarem, Palestine.

Authors' Contributions

Providing data or critical feedback on data sources

Abdullah, Bhoomadevi A, Mahesh P A, Ukachukwu O Abaraogu, Hedayat Abbastabar, Siddig Ibrahim Abdelwahab, Zinat Abdrakhmanova, Toufik Abdul-Rahman, Auwal Abdullahi, Armita Abedi, Emilia Zainal Abidin, Richard Gyan Aboagye, Abdullahi Tunde Aborode, Teklebirhan Abrha Abrha, Niveen ME Abu-Rmeileh, Fuad Hamdi A. Abuadas, Hana J Abukhadijah, Dina Abushanab, Apurba Acharya, Kamoru Ademola Adedokun, Victor Adekanmbi, Olumide Thomas Adeleke, Obed Adonteng-Kissi, Ebenezer Afrifa-Yamoah, Aiman P Afsar, Mahnaz Afshari, Saira Afzal, Mahdi Aghaalikhani, Shreyash Agrawal, Williams Agyemang-Duah, Muayyad M Ahmad, Sajjad Ahmad, Tauseef Ahmad, Ali Ahmadi, Ali Ahmed, Ayman Ahmed, Haroon Ahmed, Khabab Abbasher Hussien Mohamed Ahmed, Mehrunnisha Sharif Ahmed, Mohamed Sherif Ali Ahmed, Muktar Beshir Ahmed, Samah W Al-Jabi, Sabah Al-Marwani, Mushtak Talib Salih Al-Ouqaili, Ali Salman Al-Shami, Abdulmohsen Hamdan Al-Zalabani, Mostafa Alam, Fahad Mashhour Alanezi, Amani N. Alansari, Mohammed Albashtawy, Amidu Alhassan, Ashraf Alhumaidi, Mohammad Shahid Ali, Sheikh Mohammad Alif, Syed Mohamed Aljunid, Hazim Alkousheh, Shaymaa Hafedh Alkubaisy, Sameer Abdulmalik Alkubati, Mohammed Z. Allouh, Wesam Taher Almagharbeh, Joseph Uy Almazan, Khalid Almugheed, Mohmmad Minwer Alnaeem, Jaber S Alqahtani, Saeed Ali S Alqahtani, Intima Alrimawi, Mohammed Amjed Alsaegh, Bandar Alsaif, Abdalkarem Fedgash Alsharari, Malik A Althobiani, Nelson Alvis-Guzman, Nelson J Alvis-Zakzuk, Tarek Tawfik Amin, Saeed Amini, Mike Tuffour Amirikah, Ganiyu Adeniyi Amusa, Aisha Ansari, Abebe Alemu Anshebo, Saeid Anvari, Jalal Arabloo, Jesil Mathew Aranjani, Benedetta Armocida, Anton A Artamonov, Francis Arthur-Holmes, Tahira Ashraf, Muhammad Shahzad Aslam, Imad Asmar, Mirbahador Athari, Seyyed Shamsadin Athari, Alok Atreya, Abadi Hailay Atsbaha, Avinash Aujayeb, Salah Al Awaidy, Faseeh Bin Awais, Sana Javaid Awan, Damilola Jeremiah Ayowole, AKM Azad, Arian Azadnia, Abrham W Azale, Andreea Corina Badache, Youngoh Bae, Khlood K Baghlaf, Ruhai Bai, Atif Amin Baig, Yatan Pal Singh Balhara, Ahmed Omar Bali, Ovidiu Constantin Baltatu, Juan S. Barajas-Gamboa, Amadou Barrow, Mohammad-Mahdi Bastan, Abdul-Monim Batiha, Shazia Batool, Ibrahim Farahat El Bayoumy, Narasimha M Beeraka, Arman Karimi Behnagh, Desalegn Weldebrhan Bekru, Bashir Bello, Salaheddine Bendak, Aliyi Daba Benti, Mansi Sanjaykumar Bhadiyadra, Sonu Bhaskar, Sudip Bhattacharya, Jasvinder Singh Bhatti, Zulfiqar A Bhutta, Paria Bolourinejad, Souad Bouaoud, Soufiane Boufous, Dejana Braithwaite, Nicholas Allan Buckley, Felix Busch, Pallavi L C, Senthilkumar C, Joao Mauricio Castaldelli-Maia, Vijay Kumar Chattu, Sirshendu Chaudhuri, Akhilanand Chaurasia, Bryan Chong, Hitesh Chopra, Sunghyun Chung, Xiaochen Dai, Anh Kim Dang, Ivan Delgado-Enciso, Andreas K Demetriades, Vinoth Gnana Chellaiyan Devanbu, Devananda Devegowda, Sreedhar Dharmagadda, Samath Dhamminda Dharmaratne, Zahra Abbasi Dolatabadi, Fariba Dorostkar, Ojas Prakashbhai Doshi, Robert Kokou Dowou, Senbagam Duraisamy, Moaheda E. H. Eissa, Michael Ekholuenetale, Ashraf A El-Metwally, Randa Elsheikh, Adeniyi Francis Fagbamigbe, Ayesha Fahim, Ebenezer Gezahegn Fanta, Abdullah Farasani, Mohammad Fareed, Syed Muhammad Yousaf Farooq, Mohammad Fayaz, Alireza Feizkhah, Ginenus Fekadu, Aemon Berhane Fissha, Matteo Foschi, Kayode Raphael Fowobaje, Richard Charles Franklin, Abdelrahman Gamil Gad, Muktar A Gadanya, Balasubramanian Ganesh, Ezhilkugan Ganessane, Zisis Gatzioufas, Kalab Yigermal Gete, Mustafa Ghaderzadeh, Arin Ghamkhar, Ahmad Ghashghaee, Sama Ghoba, Alem Abera Girmay, Kimiya Gohari, Mahaveer Golechha, Pouya Goleij, Mohsen Golkar, Shi-Yang Guan, Damitha Asanga Gunawardane, Sapna Gupta, Pooja Gurnal, Aryamawit Habteyesus, Alazar Menbere Haile, Pritam Halder, Erin B

Hamilton, Asif Hanif, Nasrin Hanifi, Arief Hargono, Sara Harsini, Nada Tawfig Hashim, Simon I Hay, Jeffrey J Hebert, Mohammad Heidari, Matthew Hobbs, Mehdi Hosseinzadeh, Salman Hussain, Segun Emmanuel Ibitoye, Aminu Alhassan Ibrahim, Fatma Magdi Ibrahim, Olayinka Stephen Ilesanmi, Lalu Muhammad Irham, Oluyinka Ajibola Iyiola, Jaison Jacob, Kathryn H. Jacobsen, Mihajlo Jakovljevic, Jerin James, Balamurugan Janakiraman, Rajiv Janardhanan, Saad Javed, Talha Jawaid, Sathish Kumar Jayapal, Achala Upendra Jayatilleke, Bijay Mukesh Jeswani, Joseph Johnson, Jost B Jonas, Tamas Joo, Akaninyene Paul Joseph, Bhawana Joshi, Krupal Joshi, Charity Ehimwenma Joshua, Zubair Kabir, Dler Hussein Kadir, Bashir Kaka, Ashish Kumar Kakkar, Leila R Kalankesh, Khalil Kalavani, Ramat T. Kamorudeen, Jiseung Kang, Rami S Kantar, Samad Karkhah, Mohmed Isaqali Karobari, Faizan Zaffar Kashoo, Nehemia I. Kassa, Neda Kaydi, Peter Njenga Keiyoro, Yousef Saleh Khader, Morteza Abdullatif Khafaie, Ajmal Khan, Hayat Ahmad Khan, Iqra Hamid Khan, Maseer Khan, Muhammad Arslan Khan, Uzma Rahim Khan, Zahid Khan, Sameer Uttamaro Khasbage, Khalid A Kheirallah, Daniel Kheradmand, Milad Khorasani, Samira Khoshvaght, Jagdish Khubchandani, Yun Jin Kim, Adnan Kisa, Marta A. Kisiel, Shivakumar KM, Tapos Kormoker, Ayan Hussien Korse, Kewal Krishan, A.H. Sruthi Anil Kumar, Chandan Kumar, Dewesh Kumar, Kamal Kumar, Pawan Kumar, Pradeep Kumar, Sandeep Kumar, Asep Kusnali, Dian Kusuma, Ville Kytö, Muhammad Awwal Ladan, Chandrakant Lahariya, Tri Laksono, Savita Lasrado, Mahrukh Latif, Caterina Ledda, Ka Yiu Lee, Munjae Lee, Seung Won Lee, Wei-Chen Lee, Vasileios Leivaditis, Dawit Alemu Lemma, Zhengrui Li, Xuefeng Liu, Bryan Gervais de Liyis, Angelina M Lutambi, Miltiadis D Lytras, Ellina Lytvyak, Zheng Feei Ma, Monika Machoy-Rakoczy, Sasikumar Mahalingam, Samatar Abshir Mahamed, Preeti Maharjan, Nozad Hussein Mahmood, Mostafa Majidnia, Deborah Carvalho Malta, J Jenifer Florence Mary, Roy Rillera Marzo, Sammer Marzouk, Ali Gerami Matin, Steven M McPhail, Tesfaye Hambisa Mekonnen, Tadesse Belayneh Melkie, Walter Mendoza, Godfred Antony Menezes, Ritesh G Menezes, Emiru Ayalew Mengistie, Anusha Sultan Meo, Tesfahun Mekene Meto, Bartosz Miazgowski, Ted R Miller, Giuseppe Minervini, Chaitanya Mittal, Madeline E Moberg, Mona Gamal Mohamed, Nouh Saad Mohamed, Mokhtar Mohammadi, Abdollah Mohammadian-Hafshejani, Shafiu Mohammed, Ali H Mokdad, Dimitrios Moris, Mahdis Morovvati, Mahmoud M Morsy, Rohith Motappa, Sumaira Mubarik, Shiv Kumar Mudgal, Jibran Sualeh Muhammad, Sumoni Mukherjee, Francesk Mulita, Efren Murillo-Zamora, Christopher J L Murray, Abdisalam Hassan Muse, David Musoke, Ganesh R Naik, Nguyen Hai Nam, Gopal Nambi, Shumaila Nargus, Zuhair S Natto, Zakira Naureen, Samidi Nirasha Kumari Navaratna, Biswa Prakash Nayak, Kinjal Nayak, Iffat Nayila, Javad Nazari, Nini Asfaw Negash, Ionut Negoi, Cuong Tat Nguyen, Hien Thu Nguyen, Thinh Gia Gia Nguyen, Trang Nguyen, Richmond Nketia, Shuhei Nomura, Chimezie Igwegbe Nzoputam, Ogochukwu Janet Nzoputam, Bogdan Oancea, Ismail A Odetokun, Abiola Ogunkoya, Olusegun Olatunji Ojedoyin, Osaretin Christabel Okonji, Andrew T Olagunju, Abdulhakeem Abayomi Olorukooba, Kanyin Liane Ong, Esteban Ortiz-Prado, Amel Ouyahia, Mayowa O Owolabi, Kevin Pacheco-Barrios, Jagadish Rao Padubidri, Seithikurippu R Pandi-Perumal, Shahina Pardhan, Chulwoo Park, Eun-Cheol Park, Maja Pasovic, Bhargav Umeshchandra Patel, Hemal M Patel, Kunal Nitinkumar Patel, Niki Rut Patel, Sangram Kishor Patel, Shankargouda Patil, Mohamed Anas Patni, Uttam Paudel, Shrikant Pawar, Jarmila Pekarcikova, Prince Peprah, Simone Perna, Anil K Philip, Shiny PJ, Mohsen Poursadeqiyan, Naeimeh Pourtaheri, Xiang Qi, Nuzul Qur'aniati, Rakesh V R, Javad Sharifi Rad, Venkatraman Radhakrishnan, Amir Rafe, Pracheth Raghuveer, Sajjad Rahimi, Vafa Rahimi-Movaghar, Firman Suryadi Rahman, Amir Masoud Rahmani, Amita Rai, Sunil Kumar Raina, Judah Rajendran, Shaman Rajindrajith, Pushp Lata Rajpoot, Mahmoud Mohammed Ramadan, Kadar Ramadhan, Shakthi Kumaran Ramasamy, Balaji Ramraj, Lokesh Rana, Nemanja Rancic, Asad Gul Rao, Kumuda Rao, Mithun Rao, Sowmya J Rao, Prateek Rastogi, Rashita Ravi, Salman Rawaf, Karthik Rayapureddi, Hassan Magdy

Abd El Razek, Mohammed Magdy Abd El Razek, Ateeq Rehman, Rainer Reile, Mina Rezaei, Moattar Raza Rizvi, Jefferson Antonio Buendia Rodriguez, Leonardo Roever, Moustaq Karim Khan Rony, Devarajulu Reddy S, Aly M A Saad, Siamak Sabour, Mohd Saeed, Umar Saeed, Mehdi Safari, Sher Zaman Safi, Vaibhav Sahni, Zahra Saif, Morteza Saki, Joseph W Sakshaug, Afeez Abolarinwa Salami, Samreen Saleem, Vijaya Paul Samuel, Fasineh Shekuba Samura, Abdallah M Samy, Milena M Santric-Milicevic, Mohammad Sarmadi, Maheswar Satpathy, Iman El Sayed, Christophe Schinckus, David C Schwebel, Siddharthan Selvaraj, Subramanian Senthilkumaran, Francesco Sessa, Yashendra Sethi, Ahmed Nabil Shaaban, Shipraa Arpan Shah, Sweni Shah, Samiah Shahid, Masood Ali Shaikh, Mehran Shams-Beyranvand, Alfiya Shamsutdinova, Dan Shan, Fatemeh Saheb Sharif-Askari, Narjes Saheb Sharif-Askari, Amin Sharifan, Bhoopesh Kumar Sharma, Jyoti Sharma, Ravi Kumar Sharma, Vishal Sharma, Ramzi Shawahna, Maryam Shayan, Ali Sheidaei, Nishat Ahmed Sheikh, Jiabin Shen, Samendra P Sherchan, Mahabalesh Shetty, Premalatha K Shetty, Aminu Shittu, Nathan A Shlobin, Md. Kaoser Bin Siddique, Padam Prasad Simkhada, Harmanjit Singh, Paramdeep Singh, Surendra Singh, Valentin Yurievich Skryabin, Solikhah Solikhah, Yizhe Song, Lokasani Vijaya Sree, Muhammad Haroon Stanikzai, Chen-Yang Su, Vetriselvan Subramaniyan, Agus Sudaryanto, Nathan Suga, Chandan Kumar Swain, Lukasz Szarpak, Rafael Tabarés-Seisdedos, Seyyed Mohammad Tabatabaei, Celine Tabche, Ramin Tabibi, Mircea Tampa, Anika Tasnim, Aigul Yelgondiyevna Tazhiyeva, Nebyu Lakew Tefera, Gebremaryam Temesgen, Dejina Thapa, Ismaeel Tharwat, Jeevarathinam Thirumalai, Harshal R Thube, Madi Tleshev, Santo Imanuel Tonapa, Arole Darwin Touko, Marcos Roberto Tovani-Palone, Domenico Trico, Lawrence Sena Tuglo, Munkhtuya Tumurkhuu, Samiun Nazrin Bente Kamal Tune, Irfan Ullah, Srikanth Umakanthan, Lawan Umar, Bhaskaran Unnikrishnan, Selamu Amanuel Untiso, Era Upadhyay, Pascual R Valdez, Tommi Juhani Vasankari, Narayanaswamy Venketasubramanian, Georgios-Ioannis Verras, Tuan Vinh, Vasily Vlassov, Yasir Waheed, Wanzhou Wang, Andrea Werdecker, Taweewat Wiangkham, Dakshitha Praneeth Wickramasinghe, Andrew Awuah Wireko, Sree Sudha T Y, Eesha Yaqoob, Sanni Yaya, Pengpeng Ye, Kwabena Obiri-Yeboah, Siyan Yi, Arzu Yiğit, Vahit Yiğit, Naohiro Yonemoto, Mustafa Younis, Yong Yu, Mubashir Zafar, Iman Zare, Michael Zastrozhin, Mohammed G M Zeariya, Haijun Zhang, Abzal Zhumagaliuly, Yossef Teshome Zikarg, Sa'ed H Zyoud, and Shaher H Zyoud.

Developing methods or computational machinery

Aleksandr Y Aravkin, Xiaochen Dai, Sama Ghoba, Erin B Hamilton, Simon I Hay, Madeline E Moberg, Ali H Mokdad, Christopher J L Murray, Kanyin Liane Ong, and Maja Pasovic.

Providing critical feedback on methods or results

Abdullah, Bhoomadevi A, Mahesh P A, Ukachukwu O Abaraogu, Hedayat Abbastabar, Siddig Ibrahim Abdelwahab, Meriem Abdoun, Zinat Abdrakhmanova, Toufik Abdul-Rahman, Deldar Morad Abdulah, Auwal Abdullahi, Tesfaye Birhanu Abebe, Armita Abedi, Emilia Zainal Abidin, Richard Gyan Aboagye, Abdullahi Tunde Aborode, Nagah M. Abourashed, Mohamed Abouzid, Teklebirhan Abrha Abrha, Dariush Abtahi, Rana Kamal Abu-Farha, Fuad Hamdi A. Abuadas, Ahmad Y Abuhelwa, Hana J Abukhadijah, Dina Abushanab, Apurba Acharya, Kiran Acharya, Aishah Fadila Adamu, Isaac Yeboah Addo, Tajudeen Adesanmi Adebisi, Babatope Oluwadamilare Adebiyi, Kamoru Ademola Adedokun, Victor Adekanmbi, Olumide Thomas Adeleke, Adekola George Adepoju, Prince Owusu Adoma, Obed Adonteng-Kissi, Clifford Afoakwah, Aanuoluwapo Adeyimika Afolabi, Ebenezer Afrifa-Yamoah, Aiman P Afsar, Mahnaz Afshari, Saira Afzal, Vijay K Aggarwal, Mahdi Aghaalikhani, Salome Wumpini Agordoh, Shreyash Agrawal, Williams Agyemang-Duah, Abdul Momin Rizwan Ahmad, Aqeel Ahmad, Danish Ahmad, Khabir Ahmad, Muayyad M Ahmad, Sajjad Ahmad, Tauseef Ahmad, Ali Ahmadi, Ali Ahmed, Ayman Ahmed, Haroon

Ahmed, Khabab Abbasher Hussien Mohamed Ahmed, Mehrunnisha Sharif Ahmed, Mohamed Sherif Ali Ahmed, Muktar Beshir Ahmed, Shabbir Ahmed, Dolapo Emmanuel Ajala, Neda Akhoundi, Fahmi Y Al-Ashwal, Samah W Al-Jabi, Sabah Al-Marwani, Mushtak Talib Salih Al-Ouqaili, Ali Salman Al-Shami, Yaser Mohammed Al-Worafi, Abdulmohsen Hamdan Al-Zalabani, Sami Saleh Alabdulwahab, Khurshid Alam, Mohammad Khursheed Alam, Mohammed Fasihul Alam, Mostafa Alam, Muath A Alammar, Fahad Mashhour Alanezi, Amani N. Alansari, Hussin Albargi, Mohammed Albashtawy, Shereen M Aleidi, Amidu Alhassan, Ashraf Alhumaidi, Dari Alhuwail, Irfan Ali, Kamran Ali, Mohammad Shahid Ali, Mohammed Usman Ali, Sheikh Mohammad Alif, Moath Saleh Aljohani, Syed Mohamed Aljunid, Zaid I. Alkhatib, Hazim Alkousheh, Shaymaa Hafedh Alkubaisy, Sameer Abdulmalik Alkubati, Khaled S. Allemailem, Mohammed Z. Allouh, Wesam Taher Almagharbeh, Ahmad Almatroudi, Joseph Uy Almazan, Khalid Almugheed, Mohmmad Minwer Alnaeem, Jaber S Alqahtani, Saeed Ali S Alqahtani, Ahmad Alrawashdeh, Intima Alrimawi, Mohammed A Alsabri, Mohammed Amjed Alsaegh, Bandar Alsaif, Abdullah Alshami, Abdalkarem Fedgash Alsharari, Zaid Altaany, Malik A Althobiani, Nelson Alvis-Guzman, Nelson J Alvis-Zakzuk, Junaid Amin, Tarek Tawfik Amin, Saeed Amini, Kafayat Aminu, Mike Tuffour Amirikah, Anandhi Deva Amirtharaj, Ganiyu Adeniyi Amusa, David B Anderson, Aisha Ansari, Abebe Alemu Anshebo, Boluwatife Stephen Anuoluwa, Saeid Anvari, Jalal Arabloo, Jesil Mathew Aranjani, Demelash Areda, Anton A Artamonov, Francis Arthur-Holmes, Ashokan Arumugam, Mohammad Asghari-Jafarabadi, Mohammad Ashfaque, Tahira Ashraf, Muhammad Shahzad Aslam, Imad Asmar, Ömer Ataç, Mirbahador Athari, Seyyed Shamsadin Athari, Alok Atreya, Abadi Hailay Atsbaha, Julie Alaere Atta, Avinash Aujayeb, Leticia Avila-Burgos, Salah Al Awaidy, Faseeh Bin Awais, Sana Javaid Awan, Seyyed HamidReza Ayatizadeh, Haleh Ayatollahi, Damilola Jeremiah Ayowole, AKM Azad, Arian Azadnia, Abrham W Azale, Alireza Azarboo, Andreea Corina Badache, Ashish D Badiye, Youngoh Bae, Nayereh Baghcheghi, Nasser Bagheri, Khlood K Baghlaf, Ruhai Bai, Atif Amin Baig, Lovenish Bains, Yatan Pal Singh Balhara, Ahmed Omar Bali, Jose Balmori-de-la-Miyar, Khushbu Balsara, Ovidiu Constantin Baltatu, Samuel Alemu Bamboro, Juan S. Barajas-Gamboa, Amadou Barrow, Rehana Basri, Mohammad-Mahdi Bastan, Abdul-Monim Batiha, Shazia Batool, Kavita Batra, Ravi Batra, Ibrahim Farahat El Bayoumy, Narasimha M Beeraka, Arman Karimi Behnagh, Desalegn Weldebrhan Bekru, Suleiman Ayalew Belay, Asnake Gashaw Belayneh, Enku Shiferaw Belayneh, Bashir Bello, Aliyi Daba Benti, Alemshet Yirga Berhie, Mansi Sanjaykumar Bhadiyadra, Sonu Bhaskar, Arushee Bhatnagar, Sudip Bhattacharya, Jasvinder Singh Bhatti, Manpreet Singh Bhatti, Zulfiqar A Bhutta, Burcu Kucuk Bicer, Fengsai Bie, Corey B Bills, Abdulhakeem Binhambali, Paria Bolourinejad, Guilherme Borges, Dipan Bose, Samuel Adolf Bosoka, Souad Bouaoud, Soufiane Boufous, Dejana Braithwaite, Nicholas Allan Buckley, Tsion Samuel Bunare, Felix Busch, Yasser Bustanji, Tomasz Bączek, Pallavi L C, Senthilkumar C, Zhong Cao, Angelo Capodici, Alejandro Botero Carvajal, Ana Paula Carvalho-e-Silva, Joao Mauricio Castaldelli-Maia, Luca Cegolon, Ester Cerin, Rama Mohan Chandika, Vijay Kumar Chattu, Sirshendu Chaudhuri, Akashdeep Singh Chauhan, Akhilanand Chaurasia, Hana Chen, Bryan Chong, Hitesh Chopra, Jakir Ahmed Chowdhury, Mirza Farhana Iqbal Chowdhury, Isaac Sunday Chukwu, Eric Chung, Sunghyun Chung, Stefania Curti, Luis Alberto Cámera, Mario D'Oria, Omid Dadras, Hana Mahdi Dahir, Xiaochen Dai, Lenards Obeng Damoah, Anh Kim Dang, Ivan Delgado-Enciso, Andreas K Demetriades, Emina Dervišević, Aragaw Tesfaw Desale, Mitiku Desalegn, Vinoth Gnana Chellaiyan Devanbu, Devananda Devegowda, Arkadeep Dhali, Sreedhar Dharmagadda, Samath Dhamminda Dharmaratne, Bibha Dhungel, Adriana Dima, Mbekuveh Dinga-Nyoh, Vishal Diwan, Zahra Abbasi Dolatabadi, Fariba Dorostkar, Ojas Prakashbhai Doshi, Robert Kokou Dowou, Nguyen Tran Minh Duc, Senbagam Duraisamy, Jasmin Dönicke, Venkata Ravikanth Eddula, Yohannes Tekalegn Efa, Moaheda E. H. Eissa, Michael Ekholuenetale, Said El-Ashker, Ashraf A El-Metwally, Mohamed Ahmed

Eladl, Hala Rashad Elhabashy, Muhammed Elhadi, Omar Abdelsadek Abdou Elmeligy, Randa Elsheikh, Sharareh Eskandarieh, Elham Davtalab Esmaeili, Adeniyi Francis Fagbamigbe, Ayesha Fahim, Ebenezer Gezahegn Fanta, Emerito Jose Aquino Faraon, Abdullah Farasani, Mohammad Fareed, Carla Sofia e Sá Farinha, Andre Faro, Syed Muhammad Yousaf Farooq, Alireza Feizkhah, Ginenus Fekadu, Abdullah H Feroze, Aemon Berhane Fissha, Luisa S Flor, Matteo Foschi, Kayode Raphael Fowobaje, Richard Charles Franklin, Abdelrahman Gamil Gad, Muktar A Gadanya, Márió Gajdács, Balasubramanian Ganesh, Ezhilkugan Ganessane, Fernando Jr. Barroga Garcia, Jacopo Garlasco, Zisis Gatzioufas, Kalab Yigermal Gete, Mustafa Ghaderzadeh, Roya Ghafoury, Leila Ghalichi, Arin Ghamkhar, Ali Ghandili, Moein Ghasemi, Ahmad Ghashghaee, Ali Gholamrezanezhad, Alem Abera Girmay, Alessandro Girombelli, Massimiliano Gobbo, Kimiya Gohari, Mahaveer Golechha, Mohsen Golkar, Wei-Jie Gong, Shirisha Gooty, Shashikumar Channmgere Govindappa, Michal Grivna, Shi-Yang Guan, Mohammed Ibrahim Mohialdeen Gubari, Damitha Asanga Gunawardane, Sapna Gupta, Pooja Gurnal, Reyna Alma Gutiérrez, Roberth Steven Gutiérrez-Murillo, Adrina Habibzadeh, Aryamawit Habteyesus, Alazar Menbere Haile, Pritam Halder, Randah R Hamadeh, Fatimah Othman hamarasool, Abdelrahman M Hamouda, Asif Hanif, Nasrin Hanifi, Mohd Anul Haq, Sara Harsini, Syed Mahfuz Al Hasan, Hamidreza Hasani, Reza Hashempour, Mohammad Hashem Hashempur, Ibrahim Nagmeldin Hassan, Ikrama Hassan, Simon I Hay, Mohammad Heidari, Mojtaba Heydari, Matthew Hobbs, Ramesh Holla, Md Sabbir Hossain, Syed Billal Hossain, Mehdi Hosseinzadeh, Sorin Hostiuc, Ayesha Humayun, Waqar Husain, Salman Hussain, Segun Emmanuel Ibitoye, Aminu Alhassan Ibrahim, Fatma Magdi Ibrahim, Olayinka Stephen Ilesanmi, Irena M Ilic, Milena D Ilic, Mustapha Immurana, Danish Iqbal, Lalu Muhammad Irham, Md Rabiul Islam, Md. Mojahedul Islam, Md. Shahinul Islam, Chidozie Declan Iwu, Oluyinka Ajibola Iyiola, Juan S Izquierdo-Condoy, Jaison Jacob, Louis Jacob, Kathryn H. Jacobsen, Ali Jadidi, Mihajlo Jakovljevic, Jerin James, Roland Dominic G Jamora, Balamurugan Janakiraman, Rajiv Janardhanan, Saad Javed, Sathish Kumar Jayapal, Ruwan Duminda Jayasinghe, Achala Upendra Jayatilleke, Prof. Fariborz Sharifian Jazi, Seogsong Jeong, Bijay Mukesh Jeswani, Ravi Prakash Jha, Joseph Johnson, Jost B Jonas, Tamas Joo, Akaninyene Paul Joseph, Nitin Joseph, Bhawana Joshi, Krupal Joshi, Charity Ehimwenma Joshua, Thenmozhi K, Zubair Kabir, Dler Hussein Kadir, Bashir Kaka, Ashish Kumar Kakkar, Leila R Kalankesh, Khalil Kalavani, Rajesh Kamath, Ramat T. Kamorudeen, Jiseung Kang, Kehinde Kazeem Kanmodi, Rami S Kantar, Neeti Kapoor, Sunil Kumar Kar, Mehrdad Karajizadeh, Ibraheem M Karaye, Samad Karkhah, Mohmed Isaqali Karobari, Faizan Zaffar Kashoo, Nehemia I. Kassa, Joonas H Kauppila, Neda Kaydi, Peter Njenga Keiyoro, Yousef Saleh Khader, Morteza Abdullatif Khafaie, Ajmal Khan, Hayat Ahmad Khan, Iqra Hamid Khan, Maseer Khan, Md Abdullah Saeed Khan, Muhammad Arslan Khan, Sumaiya Khan, Uzma Rahim Khan, Yusuf Saleem Khan, Zahid Khan, Srijana Khanal, Sameer Uttamaro Khasbage, Haitham Khatatbeh, Moawiah Mohammad Khatatbeh, Kavin Khatri, Afshin Khazaei, Khalid A Kheirallah, Daniel Kheradmand, Mehdi Khezeli, Milad Khorasani, Samira Khoshvaght, Jagdish Khubchandani, P Ratan Khuman, Yun Jin Kim, Adnan Kisa, Marta A. Kisiel, Abdul Basith KM, Shivakumar KM, Prakash Babu Kodali, Ali-Asghar Kolahi, Farzad Kompani, Hamid Reza Koohestani, Wolyu Erkano Korma, Tapos Kormoker, Ayan Hussien Korse, Arthi S Kozhumam, Kewal Krishan, Varun Krishna, Mohammed Kuddus, Emmanuel Kumah, A.H. Sruthi Anil Kumar, Chandan Kumar, Dewesh Kumar, Kamal Kumar, Nithin Kumar, Pawan Kumar, Pradeep Kumar, Prakash Kumar, Radha Kumar, Sandeep Kumar, Sumit Kumar, Tushar Kumar, Om P Kurmi, Asep Kusnali, Dian Kusuma, John Paul Kuwornu, Ville Kytö, Muhammad Awwal Ladan, Lucie Laflamme, Chandrakant Lahariya, Timo Lajunen, Tri Laksono, Tamás Lantos, Savita Lasrado, Mahrukh Latif, Paolo Lauriola, Caterina Ledda, Ka Yiu Lee, Munjae Lee, Seung Won Lee, Wei-Chen Lee, Yo Han Lee, Vasileios Leivaditis, Dawit Alemu Lemma, Ming-Chieh Li, Xiaopan Li, Yan Li, Zhengrui Li, Ambreen Liaqat, Xuefeng Liu, Bryan Gervais de

Liyis, Surbala Devi Lourembam, Stephanie C. C. van der Lubbe, Abhilash Ludhiadch, Miltiadis D Lytras, Ellina Lytvyak, José Francisco López-Gil, Benson Thomas M, Zheng Feei Ma, Monika Machoy-Rakoczy, Firoozeh Madadi, Ahmed A. Madfa, Sasikumar Mahalingam, Samatar Abshir Mahamed, Preeti Maharjan, Nozad Hussein Mahmood, Azeem Majeed, Mostafa Majidnia, Igor Lima Maldonado, Deborah Carvalho Malta, Lokesh Manjani, Ramon Martinez-Piedra, J Jenifer Florence Mary, Roy Rillera Marzo, Sammer Marzouk, Sharareh Massahi, Yasith Mathangasinghe, Neeta Mathur, Ali Gerami Matin, Khurshid A Mattoo, Steven M McPhail, Agezegn Asegid Mekonnen, Tesfaye Hambisa Mekonnen, Tadesse Belayneh Melkie, Walter Mendoza, Godfred Antony Menezes, Ritesh G Menezes, Emiru Ayalew Mengistie, Anusha Sultan Meo, Tuomo J Meretoja, Tomislav Mestrovic, Tesfahun Mekene Meto, Chamila Dinushi Kukulege Mettananda, Sachith Mettananda, Mohamed M. M. Metwally, Bartosz Miazgowski, Ted R Miller, Giuseppe Minervini, Long Chiau Ming, Prasanna Mithra, Chaitanya Mittal, Madeline E Moberg, Dhruti Modi, Lia Solomon Mogus, Mona Gamal Mohamed, Nouh Saad Mohamed, Taj Mohammad, Mokhtar Mohammadi, Abdollah Mohammadian-Hafshejani, Shafiu Mohammed, Mohammad Mohseni, Ali H Mokdad, Mohammad Ali Moni, Julio Cesar Montañez, Marie Chantel Montás, Rafael Silveira Moreira, Dimitrios Moris, Mahdis Morovvati, Mahmoud M Morsy, Md. Shihab Mostafa, Rohith Motappa, Sumaira Mubarik, Shiv Kumar Mudgal, Jibran Sualeh Muhammad, Mubarak Muhammad, Sumoni Mukherjee, Francesk Mulita, Malaisamy Muniyandi, Efren Murillo-Zamora, Christopher J L Murray, Satta Muthu Murugan, Balaganapathy Muruganantham, Abdisalam Hassan Muse, David Musoke, Sathish Muthu, Ayoub Nafei, Ganesh R Naik, Nguyen Hai Nam, Gopal Nambi, Vinay Nangia, Mukesh Kumar Sathya Narayanan, Shumaila Nargus, Abdulqadir J Nashwan, Mahmoud Nassar, Zuhair S Natto, Javaid Nauman, Zakira Naureen, Samidi Nirasha Kumari Navaratna, Biswa Prakash Nayak, Kinjal Nayak, Iffat Nayila, Javad Nazari, G. Takop Nchanji, Nini Asfaw Negash, Ionut Negoi, Osires de Medeiros Melo Neto, Cuong Tat Nguyen, Hien Thu Nguyen, Thinh Gia Gia Nguyen, Trang Nguyen, Ali Nikoobar, Peishan Ning, Dina Nur Anggraini Ningrum, Vikram Niranjan, Richmond Nketia, Shuhei Nomura, Chisom Adaobi Nri-Ezedi, Jean Claude Nshimiyimana, Chimezie Igwegbe Nzoputam, Ogochukwu Janet Nzoputam, Bogdan Oancea, Ismail A Odetokun, Felix Akpojene Ogbo, Abiola Ogunkoya, Olusegun Olatunji Ojedoyin, Hassan Okati-Aliabad, Osaretin Christabel Okonji, Oluwaseyi Isaiah Olabisi, Andrew T Olagunju, Abdulhakeem Abayomi Olorukooba, Oluseye Olalekan Oludoye, Goran Latif Omer, Kanyin Liane Ong, Michal Ordak, Hans Orru, Esteban Ortiz-Prado, Alaa Oteir, Adrian Otoiu, Amel Ouyahia, Mayowa O Owolabi, Tope Oyelade, Kevin Pacheco-Barrios, Jagadish Rao Padubidri, Sanjib Raj Pandey, Seithikurippu R Pandi-Perumal, Georgios D Panos, Ilias Papadimopoulos, Evangelos Pappas, Chulwoo Park, Eun-Cheol Park, Pragnesh Parmar, Ava Pashaei, Maja Pasovic, Roberto Passera, Bhargav Umeshchandra Patel, Hemal M Patel, Kunal Nitinkumar Patel, Niki Rut Patel, Sangram Kishor Patel, Vipin Patidar, Shankargouda Patil, Mohamed Anas Patni, Dimitrios Patoulias, Uttam Paudel, Shrikant Pawar, Arun Mitra Peddireddy, Amy E Peden, Jarmila Pekarcikova, Veincent Christian Filipino Pepito, Prince Peprah, Daniela A. Perez-Chadid, Simone Perna, Anil K Philip, Julian David Pillay, Mapa M Prabhath N Piyasena, Shiny PJ, Evgenii Plotnikov, Roman V Polibin, Reza Pourbabaki, Farzad Pourghazi, Mohsen Poursadeqiyan, Naeimeh Pourtaheri, Sergio I Prada, Harsh Priya, Muhammad Usman Qamar, Xiang Qi, Mohammad Qodrati, Nuzul Qur'aniati, Rakesh V R, Basuki Rachmat, Javad Sharifi Rad, Venkatraman Radhakrishnan, Amir Rafe, Ibrar Rafique, Yashpal Singh Raghav, Pracheth Raghuveer, Sajjad Rahimi, Vafa Rahimi-Movaghar, Firman Suryadi Rahman, Fryad Majeed Rahman, Mohammad Hifz Ur Rahman, Mosiur Rahman, Muhammad Aziz Rahman, Amir Masoud Rahmani, Amita Rai, Pramila Rai, Sunil Kumar Raina, Judah Rajendran, Shaman Rajindrajith, Pushp Lata Rajpoot, Mahmoud Mohammed Ramadan, Majed Ramadan, Kadar Ramadhan, Shakthi Kumaran Ramasamy, Balaji Ramraj, Lokesh Rana, Nemanja Rancic, Asad Gul Rao, Kumuda Rao,

Mithun Rao, Sowmya J Rao, Prateek Rastogi, Devarajan Rathish, Rashita Ravi, Salman Rawaf, Karthik Rayapureddi, Hassan Magdy Abd El Razek, Mohammed Magdy Abd El Razek, Ateeq Rehman, Rainer Reile, Mina Rezaei, Nazila Rezaei, Mohsen Rezaeian, Moattar Raza Rizvi, Jefferson Antonio Buendia Rodriguez, Leonardo Roever, Ravi Rohilla, Chanapong Rojanaworarit, Moustaq Karim Khan Rony, Himanshu Sekhar Rout, Polani Rubeshkumar, Devarajulu Reddy S, Manjula S, Aly M A Saad, Siamak Sabour, Fatemeh Sadeghi-Ghyassi, Mohammad Reza Saeb, Mohd Saeed, Umar Saeed, Mehdi Safari, Sher Zaman Safi, Amene Saghazadeh, Vaibhav Sahni, Durgesh Prasad Sahoo, Zahra Saif, Morteza Saki, Joseph W Sakshaug, Payman Salamati, Afeez Abolarinwa Salami, Samreen Saleem, Sohrab Salimi, Jayami Eshana Samaranayake, Yoseph Leonardo Samodra, Vijaya Paul Samuel, Fasineh Shekuba Samura, Abdallah M Samy, Rama Krishna Sanjeev, Milena M Santric-Milicevic, Yaser Sarikhani, Mohammad Sarmadi, Sachin C Sarode, Michele Sassano, Maheswar Satpathy, Jennifer Saulam, Ganesh Kumar Saya, Iman El Sayed, Christophe Schinckus, David C Schwebel, John W Scott, Hamidreza Seifmanesh, Mohammad H Semreen, Subramanian Senthilkumaran, Francesco Sessa, Yashendra Sethi, Ahmed Nabil Shaaban, Shipraa Arpan Shah, Sweni Shah, Shazlin Shaharudin, Amira A Shaheen, Samiah Shahid, Masood Ali Shaikh, Muhammad Aaqib Shamim, Amir Hossein Shams, Mehran Shams-Beyranvand, Alfiya Shamsutdinova, Dan Shan, Mohd Shanawaz, Fatemeh Saheb Sharif-Askari, Narjes Saheb Sharif-Askari, Amin Sharifan, Bhoopesh Kumar Sharma, Bunty Sharma, Jyoti Sharma, Ravi Kumar Sharma, Saurab Sharma, Vishal Sharma, Bereket Beyene Shashamo, Shamee Shastry, Ramzi Shawahna, Samrawit Shawel, Maryam Shayan, Ali Sheidaei, Nishat Ahmed Sheikh, Rahim Ali Sheikhi, Jiabin Shen, Samendra P Sherchan, Mahabalesh Shetty, Aminu Shittu, Nathan A Shlobin, Sina Shool, Seyed Afshin Shorofi, Kerem Shuval, Md. Kaoser Bin Siddique, Gustavo Correia Basto da Silva, Padam Prasad Simkhada, Harmanjit Singh, Paramdeep Singh, Surendra Singh, Natia Skhvitaridze, Valentin Yurievich Skryabin, Bogdan Socea, Md Salman Sohel, Solikhah Solikhah, Reza Darvishi Cheshmeh Soltani, Yizhe Song, Moslem Taheri Soodejani, Lokasani Vijaya Sree, Vignes Anand Srinivasalu, Muhammad Haroon Stanikzai, Sebastian Straube, Chen-Yang Su, Omer Subasi, Vetriselvan Subramaniyan, Agus Sudaryanto, Nathan Suga, Thitiporn Sukaew, Surajo Kamilu Sulaiman, Mark J M Sullman, Jing Sun, Suraj Sundaragiri, Katharina S Sunnerhagen, Chandan Kumar Swain, Lukasz Szarpak, Rafael Tabarés-Seisdedos, Seyyed Mohammad Tabatabaei, Celine Tabche, Ramin Tabibi, Iman M Talaat, Mircea Tampa, Ingan Ukur Tarigan, Mengistie Kassahun Tariku, Anika Tasnim, Aigul Yelgondiyevna Tazhiyeva, Nebyu Lakew Tefera, Emre Tel, Gebremaryam Temesgen, Mohamad-Hani Temsah, Azimeraw Arega Tesfu, Enoch Teye-Kwadjo, Dejina Thapa, Rekha Thapar, Ismaeel Tharwat, Jeevarathinam Thirumalai, Harshal R Thube, Jansje Henny Vera Ticoalu, Madi Tleshev, Santo Imanuel Tonapa, Arole Darwin Touko, Marcos Roberto Tovani-Palone, Domenico Trico, Lawrence Sena Tuglo, Munkhtuya Tumurkhuu, Samiun Nazrin Bente Kamal Tune, Ermias A Turuse, Biruk Shalmeno Tusa, Irfan Ullah, Saeed Ullah, Srikanth Umakanthan, Lawan Umar, Bhaskaran Unnikrishnan, Carolyn Anne Unsworth, Selamu Amanuel Untiso, Era Upadhyay, Sheela Upendra, Jibrin Sammani Usman, Carlo La Vecchia, Narayanaswamy Venketasubramanian, Massimiliano Veroux, Georgios-Ioannis Verras, Victor E Villalobos-Daniel, Tuan Vinh, Francesco S Violante, Vasily Vlassov, Yasir Waheed, Arvinder Wander, Wanzhou Wang, Wei Wang, Yanzhong Wang, Apichai Wattanapisit, Anthony Ike Wegbom, Fei-Long Wei, Andrea Werdecker, Taweewat Wiangkham, Dakshitha Praneeth Wickramasinghe, Andrew Awuah Wireko, Wanqing Xie, Sree Sudha T Y, Patlolla Sriram Yadav, Vikas Yadav, Musa Yahaya, Saba Yahoo, Eesha Yaqoob, Sanni Yaya, Pengpeng Ye, Kwabena Obiri-Yeboah, Ali Cem Yekdeş, Reza Jalilzadeh Yengejeh, Siyan Yi, Arzu Yiğit, Vahit Yiğit, S Yogeshkumar, Dong Keon Yon, Naohiro Yonemoto, Mustafa Younis, Kimia Mozahheb Yousefi, Yong Yu, Siddhesh Zadey, Mubashir Zafar, Dilmurat Zairov, Michael Zastrozhin, Saad Zbiri, Mohammed G M Zeariya, Haijun Zhang, Yunquan Zhang,

Claire Chenwen Zhong, Hao Zhou, Abzal Zhumagaliuly, Yossef Teshome Zikarg, Mohammad Ahmmad Mahmoud Al Zoubi, Sa'ed H Zyoud, and Shaher H Zyoud.

Drafting the work or revising it critically for important intellectual content

Abdullah, Mahesh P A, Hasan Aalruz, Ukachukwu O Abaraogu, Hedayat Abbastabar, Siddig Ibrahim Abdelwahab, Toufik Abdul-Rahman, Auwal Abdullahi, Tesfaye Birhanu Abebe, Armita Abedi, Hansani Madushika Abeywickrama, Emilia Zainal Abidin, Olumide Abiodun, Abdullahi Tunde Aborode, Nagah M. Abourashed, Mohamed Abouzid, Teklebirhan Abrha Abrha, Rana Kamal Abu-Farha, Niveen ME Abu-Rmeileh, Fuad Hamdi A. Abuadas, Aminu Kende Abubakar, Ahmad Y Abuhelwa, Hana J Abukhadijah, Dina Abushanab, Apurba Acharya, Kiran Acharya, Aishah Fadila Adamu, Isaac Yeboah Addo, Tajudeen Adesanmi Adebisi, Babatope Oluwadamilare Adebiyi, Kamoru Ademola Adedokun, Victor Adekanmbi, Olumide Thomas Adeleke, Miracle Ayomikun Adesina, Prince Owusu Adoma, Obed Adonteng-Kissi, Aanuoluwapo Adeyimika Afolabi, Ebenezer Afrifa-Yamoah, Aiman P Afsar, Mahnaz Afshari, Saira Afzal, Mahdi Aghaalikhani, Salome Wumpini Agordoh, Shreyash Agrawal, Abdul Momin Rizwan Ahmad, Danish Ahmad, Khabir Ahmad, Muayyad M Ahmad, Tauseef Ahmad, Ali Ahmadi, Ali Ahmed, Ayman Ahmed, Haroon Ahmed, Khabab Abbasher Hussien Mohamed Ahmed, Mehrunnisha Sharif Ahmed, Mohamed Sherif Ali Ahmed, Muktar Beshir Ahmed, Shabbir Ahmed, Dolapo Emmanuel Ajala, Marjan Ajami, Fahmi Y Al-Ashwal, Samah W Al-Jabi, Mushtak Talib Salih Al-Ouqaili, Ali Salman Al-Shami, Yaser Mohammed Al-Worafi, Abdulmohsen Hamdan Al-Zalabani, Sami Saleh Alabdulwahab, Khurshid Alam, Mohammed Fasihul Alam, Mostafa Alam, Muath A Alammar, Amani N. Alansari, Hussin Albargi, Mohammed Albashtawy, Fadwa Naji Alhalaiqa, Amidu Alhassan, Ashraf Alhumaidi, Dari Alhuwail, Kamran Ali, Mohammad Shahid Ali, Mohammed Usman Ali, Moath Saleh Aljohani, Hazim Alkousheh, Shaymaa Hafedh Alkubaisy, Sameer Abdulmalik Alkubati, Khaled S. Allemailem, Mohammed Z. Allouh, Wesam Taher Almagharbeh, Ahmad Almatroudi, Khalid Almugheed, Mohmmad Minwer Alnaeem, Fahad Salman Alotibi, Jaber S Alqahtani, Saeed Ali S Alqahtani, Ahmad Alrawashdeh, Intima Alrimawi, Sahel Majed Alrousan, Mohammed Amjed Alsaegh, Bandar Alsaif, Abdalkarem Fedgash Alsharari, Zaid Altaany, Malik A Althobiani, Dominique Aluquin, Nelson Alvis-Guzman, Nelson J Alvis-Zakzuk, Junaid Amin, Tarek Tawfik Amin, Saeed Amini, Kafayat Aminu, Mike Tuffour Amirikah, Anandhi Deva Amirtharaj, Ganiyu Adeniyi Amusa, David B Anderson, Abhishek Anil, Aisha Ansari, Abebe Alemu Anshebo, Boluwatife Stephen Anuoluwa, Saeid Anvari, Jalal Arabloo, Jesil Mathew Aranjani, Benedetta Armocida, Ashokan Arumugam, Mohammad Ashfaque, Muhammad Shahzad Aslam, Imad Asmar, Ömer Ataç, Seyyed Shamsadin Athari, Alok Atreya, Avinash Aujayeb, Leticia Avila-Burgos, Salah Al Awaidy, Faseeh Bin Awais, Seyyed HamidReza Ayatizadeh, Ahmad Ayed, Damilola Jeremiah Ayowole, AKM Azad, Arian Azadnia, Abrham W Azale, Alireza Azarboo, Andreea Corina Badache, Ashish D Badiye, Adewale Ismahil` Badru, Ruhai Bai, Atif Amin Baig, Lovenish Bains, Shankar M Bakkannavar, Yatan Pal Singh Balhara, Ahmed Omar Bali, Jose Balmori-de-la-Miyar, Khushbu Balsara, Ovidiu Constantin Baltatu, Samuel Alemu Bamboro, Juan S. Barajas-Gamboa, Suzanne Lyn Barker-Collo, Amadou Barrow, Cornelia Anne Barth, Azadeh Bashiri, Mohammad-Mahdi Bastan, Abdul-Monim Batiha, Shazia Batool, Ibrahim Farahat El Bayoumy, Neeraj Bedi, Narasimha M Beeraka, Desalegn Weldebrhan Bekru, Suleiman Ayalew Belay, Bashir Bello, Salaheddine Bendak, Sonu Bhaskar, Sudip Bhattacharya, Jasvinder Singh Bhatti, Burcu Kucuk Bicer, Abdulhakeem Binhambali, Paria Bolourinejad, Guilherme Borges, Samuel Adolf Bosoka, Souad Bouaoud, Soufiane Boufous, Dejana Braithwaite, Felix Busch, Yasser Bustanji, Tomasz Bączek, Pallavi L C, Senthilkumar C, Zhong Cao, Angelo Capodici, Alejandro Botero Carvajal, Felix Carvalho, Ana Paula Carvalho-e-Silva, Joao Mauricio Castaldelli-Maia, Luca Cegolon, Ester Cerin, Sonia Cerrai, Sandip Chakraborty, Rama Mohan Chandika, Vijay Kumar Chattu, Anis Ahmad Chaudhary, Sirshendu Chaudhuri,

Akhilanand Chaurasia, Hana Chen, Patrick R Ching, Bryan Chong, Hitesh Chopra, Dinesh Kumar Choudhury, Steffan Wittrup McPhee Christensen, Eric Chung, Sunghyun Chung, Claudia Cosma, Stefania Curti, Emanuele D'Amico, Mario D'Oria, Hana Mahdi Dahir, Anh Kim Dang, Ivan Delgado-Enciso, Andreas K Demetriades, Emina Dervišević, Vinoth Gnana Chellaiyan Devanbu, Devananda Devegowda, Arkadeep Dhali, Samath Dhamminda Dharmaratne, Adriana Dima, Vishal Diwan, Sushil Dohare, Zahra Abbasi Dolatabadi, Ojas Prakashbhai Doshi, Menayit Tamrat Dresse, Nguyen Tran Minh Duc, Senbagam Duraisamy, Jasmin Dönicke, Venkata Ravikanth Eddula, Yohannes Tekalegn Efa, Moaheda E. H. Eissa, Michael Ekholuenetale, Marwan El-Deyarbi, Ashraf A El-Metwally, Iffat Elbarazi, Muhammed Elhadi, Omar Abdelsadek Abdou Elmeligy, Randa Elsheikh, Sharareh Eskandarieh, Adeniyi Francis Fagbamigbe, Ayesha Fahim, Ebenezer Gezahegn Fanta, Emerito Jose Aquino Faraon, Abdullah Farasani, Mohammad Fareed, Carla Sofia e Sá Farinha, Andre Faro, Nelsensius Klau Fauk, Nuno Ferreira, Aemon Berhane Fissha, Behzad Foroutan, Matteo Foschi, Muktar A Gadanya, Márió Gajdács, Balasubramanian Ganesh, Ezhilkugan Ganessane, Fernando Jr. Barroga Garcia, Jacopo Garlasco, Zisis Gatzioufas, Kalab Yigermal Gete, Mustafa Ghaderzadeh, Roya Ghafoury, Leila Ghalichi, Arin Ghamkhar, Alem Abera Girmay, Alessandro Girombelli, Massimiliano Gobbo, Mohsen Golkar, Wei-Jie Gong, Shirisha Gooty, Shashikumar Channmgere Govindappa, Michal Grivna, Shi-Yang Guan, Giovanni Guarducci, Damitha Asanga Gunawardane, Sapna Gupta, Pooja Gurnal, Reyna Alma Gutiérrez, Roberth Steven Gutiérrez-Murillo, Adrina Habibzadeh, Alazar Menbere Haile, Randah R Hamadeh, Fatimah Othman Hamarasool, Abdelrahman M Hamouda, Nasrin Hanifi, Mohd Anul Haq, Arief Hargono, Sara Harsini, Syed Mahfuz Al Hasan, Hamidreza Hasani, Mohammad Hashem Hashempur, Nada Tawfig Hashim, Ibrahim Nagmeldin Hassan, Simon I Hay, Jeffrey J Hebert, Matthew Hobbs, Ramesh Holla, Md Sabbir Hossain, Syed Billal Hossain, Mehdi Hosseinzadeh, Sorin Hostiuc, Junjie Huang, Ayesha Humayun, Waqar Husain, Salman Hussain, Segun Emmanuel Ibitoye, Aminu Alhassan Ibrahim, Fatma Magdi Ibrahim, Anel Ibrayeva, Olayinka Stephen Ilesanmi, Irena M Ilic, Milena D Ilic, Mustapha Immurana, Endang Indriasih, Bilal Irfan, Lalu Muhammad Irham, Md Rabiul Islam, Md. Mojahedul Islam, Md. Shahinul Islam, Chidozie Declan Iwu, Oluyinka Ajibola Iyiola, Juan S Izquierdo-Condoy, Jaison Jacob, Louis Jacob, Kathryn H. Jacobsen, Mihajlo Jakovljevic, Jerin James, Balamurugan Janakiraman, Rajiv Janardhanan, Saad Javed, Sathish Kumar Jayapal, Ruwan Duminda Jayasinghe, Achala Upendra Jayatilleke, Prof. Fariborz Sharifian Jazi, Seogsong Jeong, Bijay Mukesh Jeswani, Joseph Johnson, Jost B Jonas, Tamas Joo, Akaninyene Paul Joseph, Nitin Joseph, Bhawana Joshi, Charity Ehimwenma Joshua, Bashir Kaka, Ashish Kumar Kakkar, Leila R Kalankesh, Khalil Kalavani, Rajesh Kamath, Ramat T. Kamorudeen, Jiseung Kang, Kehinde Kazeem Kanmodi, Rami S Kantar, Neeti Kapoor, Samad Karkhah, Mohmed Isaqali Karobari, Hamid Reza Khayat Kashani, Faizan Zaffar Kashoo, Nehemia I. Kassa, Joonas H Kauppila, Neda Kaydi, Peter Njenga Keiyoro, Yousef Saleh Khader, Morteza Abdullatif Khafaie, Ajmal Khan, Hayat Ahmad Khan, Iqra Hamid Khan, Maseer Khan, Md Abdullah Saeed Khan, Serab Khan, Sumaiya Khan, Uzma Rahim Khan, Yusuf Saleem Khan, Zahid Khan, Sameer Uttamaro Khasbage, Haitham Khatatbeh, Moawiah Mohammad Khatatbeh, Khalid A Kheirallah, Milad Khorasani, Samira Khoshvaght, Jagdish Khubchandani, Yun Jin Kim, Adnan Kisa, Marta A. Kisiel, Abdul Basith KM, Shivakumar KM, Prakash Babu Kodali, Tapos Kormoker, Kewal Krishan, Varun Krishna, Mohammed Kuddus, Mukhtar Kulimbet, Chandan Kumar, Dewesh Kumar, Kamal Kumar, Pawan Kumar, Pradeep Kumar, Prakash Kumar, Radha Kumar, Rakesh Kumar, Sandeep Kumar, Sumit Kumar, Tushar Kumar, Om P Kurmi, Asep Kusnali, Dian Kusuma, John Paul Kuwornu, Ville Kytö, Muhammad Awwal Ladan, Lucie Laflamme, Chandrakant Lahariya, Balzhan Lakanova, Tamás Lantos, Savita Lasrado, Caterina Ledda, Ka Yiu Lee, Munjae Lee, Ja-Ho Leigh, Vasileios Leivaditis, Dawit Alemu Lemma, Yan Li, Zhengrui Li, Bryan Gervais de Liyis, Platon D Lopukhov, Stephanie C. C. van der Lubbe,

Abhilash Ludhiadch, Angelina M Lutambi, Miltiadis D Lytras, Ellina Lytvyak, José Francisco López-Gil, Zheng Feei Ma, Monika Machoy-Rakoczy, Sasikumar Mahalingam, Samatar Abshir Mahamed, Preeti Maharjan, Nozad Hussein Mahmood, Mostafa Majidnia, Igor Lima Maldonado, Deborah Carvalho Malta, Ramon Martinez-Piedra, J Jenifer Florence Mary, Roy Rillera Marzo, Sammer Marzouk, Yasith Mathangasinghe, Ali Gerami Matin, Khurshid A Mattoo, Steven M McPhail, Tesfaye Hambisa Mekonnen, Tadesse Belayneh Melkie, Walter Mendoza, Godfred Antony Menezes, Ritesh G Menezes, Emiru Ayalew Mengistie, Anusha Sultan Meo, Tuomo J Meretoja, Tomislav Mestrovic, Chamila Dinushi Kukulege Mettananda, Sachith Mettananda, Mohamed M. M. Metwally, Ted R Miller, Giuseppe Minervini, Prasanna Mithra, Chaitanya Mittal, Madeline E Moberg, Mona Gamal Mohamed, Nouh Saad Mohamed, Taj Mohammad, Mokhtar Mohammadi, Abdollah Mohammadian-Hafshejani, Shafiu Mohammed, Ali H Mokdad, Mohammad Ali Moni, Marie Chantel Montás, Rafael Silveira Moreira, Mahdis Morovvati, Shane Douglas Morrison, Mahmoud M Morsy, Md. Shihab Mostafa, Rohith Motappa, Shiv Kumar Mudgal, Jibran Sualeh Muhammad, Mubarak Muhammad, Francesk Mulita, Malaisamy Muniyandi, Efren Murillo-Zamora, Christopher J L Murray, Satta Muthu Murugan, Balaganapathy Muruganantham, Abdisalam Hassan Muse, David Musoke, Sathish Muthu, Ayoub Nafei, Nguyen Hai Nam, Gopal Nambi, Mukesh Kumar Sathya Narayanan, Shumaila Nargus, Abdulqadir J Nashwan, Mahmoud Nassar, Zuhair S Natto, Javaid Nauman, Samidi Nirasha Kumari Navaratna, Biswa Prakash Nayak, Kinjal Nayak, Iffat Nayila, Javad Nazari, G. Takop Nchanji, Nini Asfaw Negash, Ionut Negoi, Samata Nepal, Osires de Medeiros Melo Neto, Marie Ng, Cuong Tat Nguyen, Hien Thu Nguyen, Thinh Gia Gia Nguyen, Trang Nguyen, Taxiarchis Konstantinos Nikolouzakis, Peishan Ning, Vikram Niranjan, Richmond Nketia, Chisom Adaobi Nri-Ezedi, Jean Claude Nshimiyimana, Chimezie Igwegbe Nzoputam, Ogochukwu Janet Nzoputam, Bogdan Oancea, Fabio Massimo Oddi, Ismail A Odetokun, Felix Akpojene Ogbo, Abiola Ogunkoya, Olusegun Olatunji Ojedoyin, Osaretin Christabel Okonji, Andrew T Olagunju, Abdulhakeem Abayomi Olorukooba, Oluseye Olalekan Oludoye, Kanyin Liane Ong, Michal Ordak, Hans Orru, Augustus Osborne, Alaa Oteir, Adrian Otoiu, Amel Ouyahia, Mayowa O Owolabi, Tope Oyelade, Kevin Pacheco-Barrios, Jagadish Rao Padubidri, Ashok Pandey, Seithikurippu R Pandi-Perumal, Ilias Papadimopoulos, Evangelos Pappas, Shahina Pardhan, Chulwoo Park, Ava Pashaei, Roberto Passera, Hemal M Patel, Kunal Nitinkumar Patel, Niki Rut Patel, Shankargouda Patil, Mohamed Anas Patni, Dimitrios Patoulias, Uttam Paudel, Shrikant Pawar, Amy E Peden, Veincent Christian Filipino Pepito, Daniela A. Perez-Chadid, Simone Perna, Shahrzad Bazargan-Hejazi, PhD, Anil K Philip, Julian David Pillay, Mapa M Prabhath N Piyasena, Reza Pourbabaki, Sergio I Prada, Harsh Priya, Xiang Qi, Mohammad Qodrati, Nuzul Qur'aniati, Javad Sharifi Rad, Venkatraman Radhakrishnan, Amir Rafe, Pracheth Raghuveer, Sajjad Rahimi, Vafa Rahimi-Movaghar, Mohammad Hifz Ur Rahman, Amir Masoud Rahmani, Amita Rai, Sunil Kumar Raina, Judah Rajendran, Shaman Rajindrajith, Mahmoud Mohammed Ramadan, Kadar Ramadhan, Shakthi Kumaran Ramasamy, Balaji Ramraj, Lokesh Rana, Nemanja Rancic, Asad Gul Rao, Kumuda Rao, Mithun Rao, Sowmya J Rao, Devarajan Rathish, Salman Rawaf, Karthik Rayapureddi, Hassan Magdy Abd El Razek, Mohammed Magdy Abd El Razek, Ateeq Rehman, Rainer Reile, Mina Rezaei, Julio Cesar Campuzano Rincon, Moattar Raza Rizvi, Jefferson Antonio Buendia Rodriguez, Leonardo Roever, Ravi Rohilla, Chanapong Rojanaworarit, Bedanta Roy, Polani Rubeshkumar, Manjula S, Aly M A Saad, Siamak Sabour, Umar Saeed, Mehdi Safari, Amirhossein Sahebkar, Vaibhav Sahni, Zahra Saif, Morteza Saki, Joseph W Sakshaug, Afeez Abolarinwa Salami, Samreen Saleem, Fasineh Shekuba Samura, Abdallah M Samy, Rama Krishna Sanjeev, Milena M Santric-Milicevic, Aswini Saravanan, Yaser Sarikhani, Mohammad Sarmadi, Sachin C Sarode, Michele Sassano, Maheswar Satpathy, Ganesh Kumar Saya, Iman El Sayed, David C Schwebel, John W Scott, Hamidreza Seifmanesh, Siddharthan Selvaraj, Mohammad H

Semreen, Subramanian Senthilkumaran, Francesco Sessa, Yashendra Sethi, Ahmed Nabil Shaaban, Sweni Shah, Shazlin Shaharudin, Amira A Shaheen, Samiah Shahid, Moyad Jamal Shahwan, Muhammad Aaqib Shamim, Amir Hossein Shams, Mehran Shams-Beyranvand, Alfiya Shamsutdinova, Mohd Shanawaz, Fatemeh Saheb Sharif-Askari, Amin Sharifan, Bunty Sharma, Jyoti Sharma, Manoj Sharma, Saurab Sharma, Vishal Sharma, Bereket Beyene Shashamo, Shamee Shastry, Ramzi Shawahna, Nishat Ahmed Sheikh, Jiabin Shen, Samendra P Sherchan, Mahabalesh Shetty, Pavanchand Shetty, Premalatha K Shetty, Suraj S Shetty, Aminu Shittu, Nathan A Shlobin, Sina Shool, Seyed Afshin Shorofi, Kerem Shuval, Md. Kaoser Bin Siddique, Gustavo Correia Basto da Silva, Padam Prasad Simkhada, Akanksha Singh, Harmanjit Singh, Paramdeep Singh, Poornima Suryanath Singh, Surendra Singh, Surjit Singh, Natia Skhvitaridze, Valentin Yurievich Skryabin, Bogdan Socea, Solikhah Solikhah, Lokasani Vijaya Sree, Muhammad Haroon Stanikzai, Paschalis Steiropoulos, Sebastian Straube, Chen-Yang Su, Omer Subasi, Vetriselvan Subramaniyan, Thitiporn Sukaew, Surajo Kamilu Sulaiman, Mark J M Sullman, Katharina S Sunnerhagen, Lukasz Szarpak, Rafael Tabarés-Seisdedos, Celine Tabche, Ramin Tabibi, Iman M Talaat, Mircea Tampa, Anika Tasnim, Emre Tel, Mohamad-Hani Temsah, Reem Temsah, Azimeraw Arega Tesfu, Dejina Thapa, Ismaeel Tharwat, Jeevarathinam Thirumalai, Harshal R Thube, Santo Imanuel Tonapa, Hamidreza Pazoki Toroudi, Arole Darwin Touko, Marcos Roberto Tovani-Palone, Thang Huu Tran, Domenico Trico, Lawrence Sena Tuglo, Samiun Nazrin Bente Kamal Tune, Irfan Ullah, Srikanth Umakanthan, Lawan Umar, Bhaskaran Unnikrishnan, Selamu Amanuel Untiso, Era Upadhyay, Jibrin Sammani Usman, Asokan Govindaraj Vaithinathan, Pascual R Valdez, Tommi Juhani Vasankari, Carlo La Vecchia, Narayanaswamy Venketasubramanian, Akshaya Kumar Verma, Massimiliano Veroux, Georgios-Ioannis Verras, Victor E Villalobos-Daniel, Tuan Vinh, Vasily Vlassov, Yasir Waheed, Wanzhou Wang, Wei Wang, Yanzhong Wang, Apichai Wattanapisit, Anthony Ike Wegbom, Fei-Long Wei, Andrea Werdecker, Taweewat Wiangkham, Dakshitha Praneeth Wickramasinghe, Faustine Williams, Andrew Awuah Wireko, Sree Sudha T Y, Patlolla Sriram Yadav, Vikas Yadav, Musa Yahaya, Saba Yahoo, Eesha Yaqoob, Sanni Yaya, Pengpeng Ye, Kwabena Obiri-Yeboah, Ali Cem Yekdeş, Siyan Yi, Arzu Yiğit, Vahit Yiğit, S Yogeshkumar, Naohiro Yonemoto, Kimia Mozahheb Yousefi, Yong Yu, Mubashir Zafar, Aurora Zanghì, Iman Zare, Michael Zastrozhin, Mohammed G M Zeariya, Zhi-Jiang Zhang, Claire Chenwen Zhong, Hao Zhou, Yawen Zhou, Yossef Teshome Zikarg, Mohammad Ahmmad Mahmoud Al Zoubi, Sa'ed H Zyoud, and Shaher H Zyoud.

Managing the estimation or publications process

Erin B Hamilton, Simon I Hay, Madeline E Moberg, Ali H Mokdad, Christopher J L Murray, Kanyin Liane Ong, and Maja Pasovic.
